# Exploration and characterization of the antimalarial activity of cyclopropyl carboxamides that target the mitochondrial protein, cytochrome *b*

**DOI:** 10.1016/j.ejmech.2024.116921

**Published:** 2024-12-15

**Authors:** Jon Kyle Awalt, Wenyin Su, William Nguyen, Katie Loi, Kate E. Jarman, Jocelyn S. Penington, Saishyam Ramesh, Kate J. Fairhurst, Tomas Yeo, Heekuk Park, Anne-Catrin Uhlemann, Bikash Chandra Maity, Nirupam De, Partha Mukherjee, Arnish Chakraborty, Alisje Churchyard, Mufuliat T. Famodimu, Michael J. Delves, Jake Baum, Nimisha Mittal, Elizabeth A. Winzeler, Anthony T. Papenfuss, Mrittika Chowdury, Tania F. de Koning-Ward, Alexander G. Maier, Giel G. van Dooren, Delphine Baud, Stephen Brand, David A. Fidock, Paul F. Jackson, Alan F. Cowman, Madeline G. Dans, Brad E. Sleebs

**Affiliations:** a The Walter and Eliza Hall Institute of Medical Research, Parkville, 3052, Australia; b Department of Medical Biology, The University of Melbourne, Parkville, 3010, Australia; c Research School of Biology, The Australian National University, Canberra, 2600, Australia; d Department of Microbiology & Immunology, Columbia University Irving Medical Center, New York, NY, USA; e Center for Malaria Therapeutics and Antimicrobial Resistance, Columbia University Irving Medical Center, New York, NY, USA; f Division of Infectious Diseases, Department of Medicine, Columbia University Irving Medical Center, New York, NY, USA; g TCG Lifesciences, Kolkata, West Bengal, 700091, India; h Department of Life Sciences, Imperial College London, South Kensington, SW7 2AZ, UK; i Department of Infection Biology, London School of Hygiene and Tropical Medicine, London, WC1E 7HT, UK; j School of Biomedical Sciences, University of New South Wales, Sydney, 2031, Australia; k School of Medicine, University of California San Diego, La Jolla, CA, 92093, USA; l School of Medicine, Deakin University, Waurn Ponds, Victoria, 3216, Australia; m Institute for Mental and Physical Health and Clinical Translation, Deakin University, Geelong, Victoria, 3216, Australia; n Medicines for Malaria Venture, Geneva, 1215, Switzerland; o Global Public Health, Janssen R&D LLC, La Jolla, 92121, USA

**Keywords:** Malaria, *Plasmodium*, Antimalarial, Mitochondria, Cytochrome *b*

## Abstract

Drug resistance against antimalarials is rendering them increasingly ineffective and so there is a need for the development of new antimalarials. To discover new antimalarial chemotypes a phenotypic screen of the Janssen Jumpstarter library against the *P. falciparum* asexual stage was undertaken, uncovering the cyclopropyl carboxamide structural hit class. Structure-activity analysis revealed that each structural moiety was largely resistant to change, although small changes led to the frontrunner compound, WJM280, which has potent asexual stage activity (EC_50_ 40 nM) and no human cell cytotoxicity. Forward genetics uncovered that cyclopropyl carboxamide resistant parasites have mutations and an amplification in the *cytochrome b* gene. Cytochrome *b* was then verified as the target with profiling against cytochrome *b* drug-resistant parasites and a mitochondrial oxygen consumption assay. Accordingly, the cyclopropyl carboxamide class was shown to have slow-acting asexual stage activity and activity against male gametes and exoerythrocytic forms. Enhancing metabolic stability to attain efficacy in malaria mouse models remains a challenge in the future development of this antimalarial chemotype.

## Highlights

• HT screens against *P. falciparum* identified the cyclopropyl carboxamide chemotype.
• Analogs exhibit potent antiparasitic activity and have high solubility.
• Forward genetics demonstrated the molecular target is cytochrome *b.*
• Analogs are slow-acting and kill liver and transmission stage parasites.

## Graphical abstract

**Figure:**
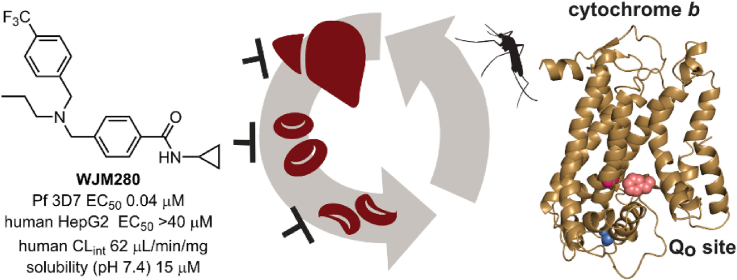

## 1 Introduction

Malaria is a disease that causes significant human morbidity and mortality. Malaria in humans is caused by five species of *Plasmodium* parasites. *P. falciparum* is the most deadly accounting for approximately 70 % of reported cases of malaria and 95 % of deaths globally and >99 % of deaths in sub-Saharan Africa [1]. *P. vivax* infections are most prevalent in South East Asia and the Americas and has a dormant liver stage that is responsible for a delay in symptoms weeks to months after traveling to an endemic area. *P. ovale* and *P. malariae* are known to cause infection and mild symptoms while *P. knowlesi,* a parasite of simian origin, is an emerging pathogen of concern in Southeast Asia.

The malaria parasite is transmitted from one human host to another by way of a bloodmeal from the female *Anopheles* mosquito. When the parasite enters the bloodstream, it travels to the liver to establish the liver stage infection. The parasite replicates and releases hundreds of merozoites into the blood stream to establish the asexual blood stage infection. Multiple rounds of the asexual stage replication cycle leads to exponential growth of the malaria parasite, which destroys many red blood cells leading to the clinical symptoms of the disease. At some point in the asexual stage cycle, sexual gametocytes are generated which are taken up by the mosquito to transmit the disease to another human host. Each stage of the malaria parasite lifecycle represents a potential bottleneck and a point of intervention by vaccines and antimalarials. These are largely aligned with curative (asexual stage), chemoprevention (liver and asexual stages), and transmission blocking (asexual and sexual/mosquito stages) target candidate profiles (TCPs) recommended for new antimalarial therapies [2].

The two current therapeutic approaches to combat malaria infection are preventative and treatment therapies. Prevention strategies to ablate malaria infection include vaccination and chemoprotective therapies. Chemopreventative therapies include suppressive and causal prophylaxis. Suppressive chemoprophylaxis, such as doxycycline, and mefloquine only impacts asexual blood stage parasites. Examples of causal chemoprophylaxis are primaquine, tafenoquine, and Malarone (a combination of atovaquone (ATQ) and pyrimethamine) which can prevent exoerythrocytic (liver stage) development and establishment of symptomatic asexual blood stage parasitemia.

Curative therapies have historically relied on several antimalarial drug classes which consist of quinolines, such as chloroquine and tafenoquine; antifolate and Mannich-based drugs, sulfadoxine and pyronaridine, and aryl amino alcohol-based drugs, such as halofantrine and lumefantrine. The overuse of these drug classes as monotherapies has resulted in widespread resistance rendering them increasingly ineffective. As a result, the frontline therapies to treat malaria are artemisinin combination therapies (ACTs) such as Artemether-lumefantrine. Concerningly, resistance against these frontline therapies has emerged in South-East Asia [3] and more recently in Africa [4].

In the last 20 years, there has been a concerted effort from industry and academia to mass screen compound libraries against the malaria parasite to uncover novel chemotypes for development. This has resulted in many promising new antimalarial chemotypes that are under development, and several have transitioned to human clinical trials. The most advanced include KAE609, a PfATP4 inhibitor, MMV048, a phosphatidylinositol 4-kinase (PI4K) inhibitor and DSM265, a dihydroorotate dehydrogenase (DHODH) inhibitor [5]. Concerningly, mutations have been detected in recrudescent parasites from treated volunteers treated with KAE609 and DMS265 [6,7]. Moreover, there is an ongoing need to identify new chemotypes for development to populate the global antimalarial pipeline.

**Fig. 1:**
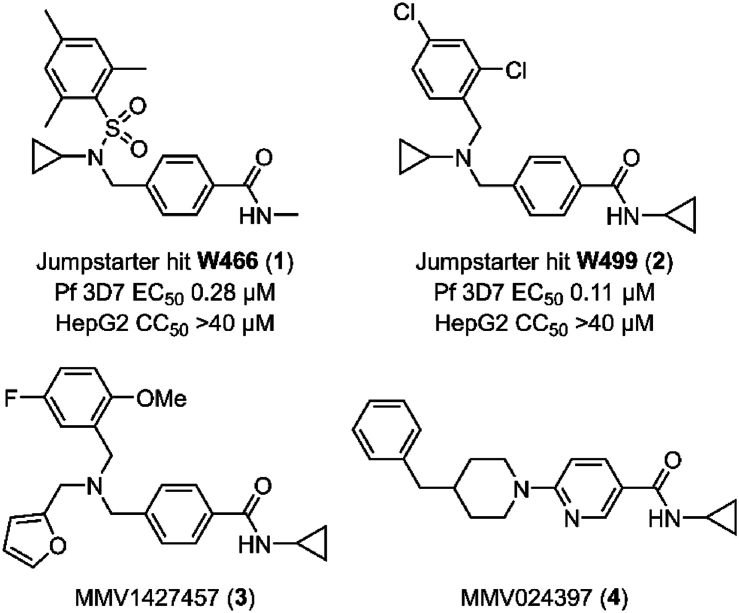
Structures and activity of carboxamide hits from the screen of the Jumpstarter library and structurally related compounds previously reported [16,17].

To discover new antimalarial chemotypes for development, we screened the Janssen Jumpstarter library of 80,000 drug-like compounds against the asexual stage parasite using a lactate dehydrogenase (LDH) assay for analysis of parasite growth [8]. Primary hit compounds were then confirmed using a 72 h asexual stage parasite LDH assay in a dose-response format. Counterscreen assays were also undertaken to ensure hit compounds did not interfere with the LDH assay technology. Hit compounds were further assessed for cytotoxicity in a human HepG2 cell assay. Confirmed hits from the screening cascade included the triazolopyrimidine structural class with an unknown mechanism of action [9], a dihydro-quinazolinone scaffold that targets PfATP4 [[10], [11], [12]], aryl acetamides that target the StAR-related lipid transfer 1 protein [13,14], pyrazolopyridine 4-carboxamide class that has decreased activity against ABCI3 mutant parasites [15], and the 3-oxadiazole quinolone chemotype that targets cytochrome *b* (cyt *b*) [8]. Here we report on the Jumpstarter screening hit compounds W466 (**1**) and W499 (**2**) that have a structure that consists of a methyl and cyclopropyl carboxamide moiety (Fig. 1). The hit carboxamide compounds, W466 (**1**) and W499 (**2**) were shown to exhibit modest potency against the asexual stage parasite (EC_50_ 0.11 and 0.28 μM) and have no human HepG2 cell growth inhibition (CC_50_ > 40 μM) (Fig. 1), and therefore were deemed suitable for further investigation.

At the time of our investigation into the cyclopropyl carboxamide chemotype, Antonova-Koch et al. disclosed their findings on the similar cyclopropyl carboxamide structural class (represented by MMV1427457 (**3**) in Fig. 1) which was identified from a high-throughput screen against the malaria parasite [16]. In this research, it was shown that *P. falciparum* parasites expressing *S. cerevisiae* yeast DHODH show reduced sensitivity to MMV1427457, a signature of compounds impacting the mitochondria electron transport chain (ETC). More recently, Hayward et al. screened the Medicines for Malaria (MMV) Pathogen Box searching for Apicomplexa mitochondrial ETC inhibitors and identified the cyclopropyl carboxamide analog, MMV024397 (**4**) (Fig. 1) [17]. MMV024397 was subsequently found to inhibit cyt b (complex III) in the mitochondria ETC and showed decreased sensitivity to an ATQ resistant strain with a mutation in the Qo site of cyt b. Both MMV1271037 and MMV024397 share structural similarity with the hit compounds W466 (**1**) and W499 (**2**), and therefore it was reasoned that the hit compounds may also target cyt *b* in the mitochondria ETC.

Cyt *b* is a component of the cytochrome *bc*1 complex (complex III) in the mitochondrial electron transport chain (ETC). The ETC consists of five mitochondrial dehydrogenases (NADH:ubiquinone oxidoreductase, succinate-ubiquinone oxidoreductase (complex II), malate:quinone oxidoreductase, DHODH, and glyceraldehyde-3-phosphate dehydrogenase) that reduce coenzyme Q (CoQ). Coenzyme Q (ubiquinone) is then reduced by complex III (cytochrome *bc*_1_) to ubiquinol creating an electron gradient across the inner mitochondrial membrane (ΔΨ_m_). Cytochrome *c* then shuttles electrons to complex IV. This provides the proton motive force for complex V (ATP synthase). Unlike most eukaryotes, complex V does not contribute to the energy supply in *Plasmodium* parasites for which glycolysis is the primary energy source. The ETC however is required as a source of electrons for DHODH which is essential for pyrimidine biosynthesis in asexual stage parasites [18]. DHODH is an established antimalarial drug target, with DSM265 in human clinical trials [19].

Cyt *b* is a component of complex III (cytochrome *bc*_1_) and is a well-established and clinically validated antimalarial drug target. Cyt *b* is highly conserved across *Plasmodium* species but is structurally divergent from the human form and this is the reason cyt *b* inhibitors selectively target the malaria parasite. Cyt *b* is typically targeted by compounds with quinolone or pyridone structures that have structural similarity to the natural substrate ubiquinone, such as ATQ and ELQ300 [20]. ATQ binds to the Q_o_ site of cyt *b* and is typically clinically used in the chemopreventative therapy, Malarone [21,22]. ELQ300 binds to the Q_i_ site of cyt *b* and has advanced to human clinical trials [23]. A significant hurdle for the development of new cyt *b* inhibitors is the fast onset of clinical resistance, which readily occurs because of the point mutations in the mitochondrially encoded *cyt b* gene [24]. Compounding this factor is the decreased sensitivity of compounds in development to parasites with clinically derived mutations in Q_o_ or Q_i_ site of cyt *b* [23,25]. Overcoming these barriers is a key factor in the development of new cyt *b* inhibitors.

Herein, we investigate the suitability of the cyclopropyl carboxamide series for overcoming resistance factors commonly associated with cyt *b* inhibitors and for antimalarial development. We establish the structure-activity relationship (SAR) and use this information to optimize the asexual stage activity of the cyclopropyl carboxamide class, while monitoring physicochemical properties, *in vitro* metabolism, and aqueous solubility. We determine the mechanism of action of the cyclopropyl carboxamide series by performing resistance selection followed by whole genome sequencing. The mechanism of action was subsequently confirmed by profiling against drug-resistant parasite lines and a functional oxygen consumption assay. We then characterize the asexual stage of arrest and rate of kill and assess the liver stage and transmission blocking potential of the cyclopropyl carboxamide class, and finally assess efficacy in a *P. berghei* mouse model.

## 2 Results and discussion

### 2.1 Synthesis

**Scheme 1:**
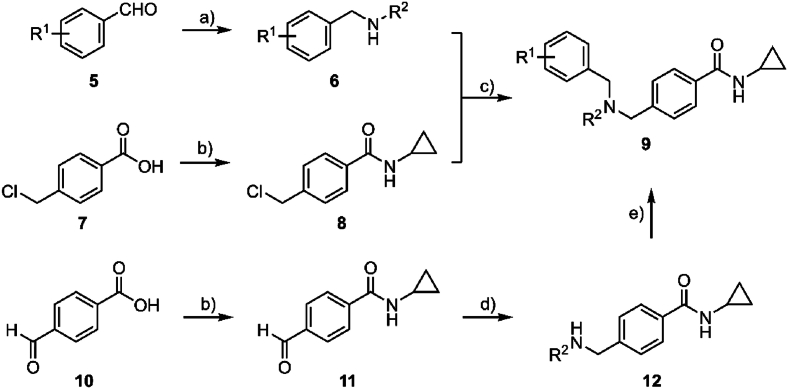
General synthetic pathway to access derivatives. *Reagents and conditions:* (a) R^2^-NH_2_, NaBH_4_, AcOH, MeOH, 0 °C, then rt, 2 h, 60–93 %; (b) i. oxalyl chloride, cat. DMF, DCM, rt, 2 h; ii. cyclopropylamine, Et_3_N, DCM, 0 °C, then rt, 2 h, 87 %; (c) K_2_CO_3_, MeCN, reflux, 16 h, 21–62 %; (d) R^2^-NH_2_, NaBH(OAc)_3_, AcOH, DCE, rt, 16 h, 10–54 %; (e) R^1^-PhCHO, NaBH(OAc)_3_, AcOH, DCE, rt, 2 h, 13–86 %.

Two general synthetic pathways were designed to access cyclopropyl carboxamide analogs with functional group variations on the pendant aryl group and the central tertiary amine (Scheme 1). The first synthesis begins with a reductive amination of an aryl aldehyde **5** and a primary amine using NaBH_4_ to afford the secondary amine **6**. In parallel, 4-chloromethyl aryl carboxylic acid **7** was chlorinated with oxalyl chloride to form the acid chloride in situ, and then subsequently cyclopropylamine was added to form the corresponding carboxamide **8**. The secondary amine **6** was then alkylated with the 4-chloromethyl aryl carboxamide **8** to generate the product **9**. Alternatively, the synthesis began with chlorination of 4-formyl phenyl carboxylic acid **10** using oxalyl chloride. The carboxamide **11** was then formed by the reaction of the acid chloride with cyclopropylamine, and this was followed by reductive amination of the appropriate amine using NaBH(OAc)_3_ to afford the secondary amine **12**. The secondary amine **12** was subjected to another reductive amination using the same conditions with an aryl aldehyde to yield the product **9**.

**Scheme 2:**
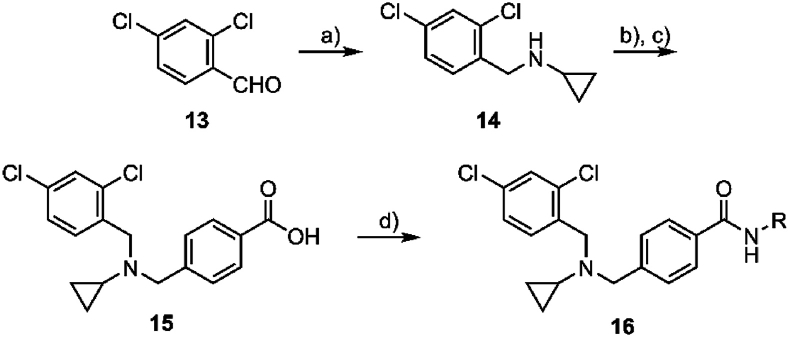
Synthesis of N-substituted carboxamide analogs. *Reagents and conditions:* (a) cyclopropylamine, NaBH_4_, AcOH, MeOH, DCE, 0 °C, then rt, 24 h, 66–70 %; (b) methyl 4-formylbenzoate, NaBH(OAc)_3_, AcOH, DCE, rt, 16 h, 66 %; (c) LiOH·H_2_O, THF, MeOH, H_2_O, 70 °C, 3 h, 91 %; (d) R–NH_2_, EDCI, HOBt, DIPEA, DMF, rt, 15 h, 27–71 %.

Another synthetic pathway was undertaken to produce analogs with variations to the substituent on the carboxamide (Scheme 2). This synthesis started with a reductive amination between the aryl aldehyde **13** and cyclopropylamine using NaBH(OAc)_3_ to give the secondary amine **14**. A second reductive amination was then applied to the secondary amine **14** using methyl 4-formyl benzoate and NaBH(OAc)_3_, followed by hydrolysis of the carboxylate using lithium hydroxide to give the carboxylic acid **15**. Finally, the carboxylic acid **15** was activated with EDCI and HOBt in the presence of DIPEA and reacted with the appropriate amine to give the substituted carboxamide product **16**.

### 2.2 Structure activity relationship

To establish the SAR, synthesized analogs were assessed against the asexual stage parasite (72 h) using LDH as a proxy for parasite growth inhibition. Cytotoxicity was monitored against the human HepG2 cell line using Cell TitreGlo as a metabolic marker for cell growth [26]. Cyt *b* inhibitors typically have high lipophilicity, and so calculated physicochemical properties were determined, while *in vitro* ADME was also collected on representative analogs to gauge their suitability for efficacy studies in a 4-day *P. berghei* model.

The investigation of the SAR began with the exploration of the substitution on the central nitrogen using an unsubstituted aryl variant as a template (**17**) (EC_50_ 0.14 μM) (Table 1) of the hit compound W499 (**2**). It was found that the removal of the *N*-cyclopropyl substituent (**18**) significantly impacted antiparasitic activity (EC_50_ > 10 μM). Replacing the *N*-cyclopropyl group with a methyl or ethyl substituent (**19** and **20**) reduced activity by 20- and 4-fold (EC_50_ 2.6 and 0.70 μM) relative to **17**. Aliphatic groups of a similar steric volume to a cyclopropyl were then incorporated, including isopropyl, cyclobutyl, *t*-butyl, *sec*-butyl, and trifluoroethyl groups (**21**–**25**) which all had similar potency (EC_50_ 0.16–0.63 μM) to the cyclopropyl (**17**), while the *n*-propyl (**26**) was approximately as active (EC_50_ 0.13 μM). A benzyl group in this position (**27**) was tolerated (EC_50_ 0.26 μM), which was unexpected given its steric bulk in comparison to the size of the cyclopropyl group. Compound **28**, featuring the carboxamide group in the meta position relative to the methyl amino functionality was also synthesized and exhibited a 40-fold decrease in activity (EC_50_ 5.6 μM), signifying the para-substitution pattern is optimal. None of the analogs in Table 1 demonstrated adverse cytotoxicity toward the human HepG2 cell line (CC_50_ > 40 μM).

**Table 1:**
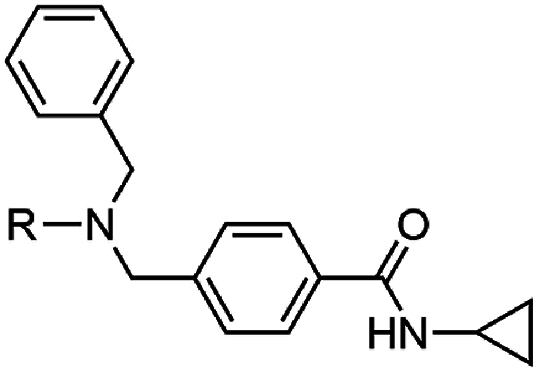
Activities of N-substituted analogs.

compound	R	Pf 3D7 EC_50_ (SD) μM^a^	HepG2 CC_50_ μM^b^
**17**	cyPr	0.14 (0.01)	>40
**18**	H	>10	nd
**19**	Me	2.6 (1.3)	>40
**20**	Et	0.70 (0.1)	>40
**21**	*i-*Pr	0.16 (0.01)	>40
**22**	cyBu	0.16 (0.07)	>40
**23**	*t*-Bu	0.63 (0.3)	>40
**24**	*sec-*Bu	0.21 (0.03)	>40
**25**	CH_2_CF_3_	0.26 (0.01)	>40
**26**	*n*-Pr	0.13 (0.06)	>40
**27**	Bn	0.59 (0.05)	>40
**28**^c^	cyPr	5.6 (0.8)	>40

a EC_50_ data represents means and SDs for three or more independent experiments measuring LDH activity of *P. falciparum* 3D7 parasites over 72 h.

b CC_50_ data represents an average of two or more independent experiments measuring HepG2 cell growth inhibition over 48 h using Cell TitreGlo.

c Carboxamide in the meta-position. Cy = cyclo.

Substitution on the carboxamide was next explored using the hit compound W499 (**2**) scaffold as a template. Deletion of the cyclopropyl group from the carboxamide (**29**) was not tolerated (EC_50_ > 10 μM) (Table 2), indicating the importance of N-substitution for activity. Modifying the cyclopropyl group to aliphatic groups of a similar steric size including methyl, ethyl, *n*-propyl, and isopropyl (**30**–**33**) led to 3- to 7-fold loss in activity (EC_50_ 0.31–1.18 μM), while a trifluoroethyl substituent (**34**) led to a more severe loss of activity (EC_50_ > 10 μM). Replacing the cyclopropyl group with an ethyl group terminating in polar functionalities (**35**–**37**) was not tolerated (EC_50_ 2.4 – >10 μM). Notably, the ethyl-alcohol functionality (**37**) is a possible metabolite of the cyclopropyl group and is approximately 25-fold less active (EC_50_ 2.4 μM). Expanding the cyclopropyl ring to cyclobutyl (**38**) resulted in a 4-fold loss in activity (EC_50_ 0.48 μM) while the cyclopentyl variation (**39)** was 12-fold less active (EC_50_ 1.2 μM). A phenyl ring (**40**) gave similar activity to the cyclobutyl analog (EC_50_ 0.55 μM) and N-methylation of the cyclopropyl carboxamide (**41**) led to a loss in activity (EC_50_ > 10 μM). Collectively, this data suggests that the cyclopropyl carboxamide is optimal for activity. Notably, the cyclopropyl carboxamide moiety is common and assumedly important to the activity of several antimalarial compounds that putatively target cyt *b* (Fig. 1) [16,17].

**Table 2:**
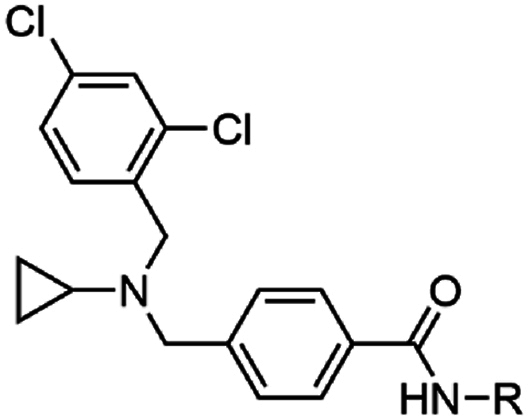
Activities of carboxamide substituted derivatives.

compound	R	Pf 3D7 EC_50_ (SD) μM^a^	HepG2 CC_50_ μM^b^
W466 (**1**)	cyPr	0.11 (0.01)	>40
**29**	H	>10	nd
**30**	Me	1.18 (0.17)	>40
**31**	Et	0.31 (0.02)	>40
**32**	*n-*Pr	0.64 (0.02)	>40
**33**	*i-*Pr	0.69 (0.03)	>40
**34**	CH_2_CF_3_	>10	nd
**35**	(CH_2_)_2_NMe_2_	7.6 (2.3)	9.3
**36**	(CH_2_)_2_NH_2_	>10	nd
**37**	(CH_2_)_2_OH	2.4 (0.2)	>40
**38**	cyBu	0.48 (0.06)	>40
**39**	cyPen	1.8 (0.7)	>40
**40**	Ph	1.2 (0.08)	>40
**41**	cyPr^c^	>10	nd

a EC_50_ data represents means and SDs for three or more independent experiments measuring LDH activity of *P. falciparum* 3D7 parasites over 72 h.

b CC_50_ data represents an average of two or more independent experiments measuring HepG2 cell growth inhibition over 48 h using Cell TitreGlo.

c N-methyl variant. Cy = cyclo.

Substitution on the pendant aryl ring was next explored using the unsubstituted analog **17** as the benchmark (EC_50_ 0.14 μM). The addition of a 2, 3, or 4- fluoro or chloro atom on the aryl ring (**42**–**47**) slightly decreased the antimalarial activity (EC_50_ 0.22–0.34 μM) (Table 3). The 2-methyl and 2-trifluoromethyl substituents (**48** and **49**) also showed slightly decreased activity (EC_50_ 0.20 and 0.22 μM), whereas the 3- and 4- methyl and trifluoromethyl variations (**50**–**53**) were approximately equipotent (EC_50_ 0.11–0.22 μM). Hydrophobic aliphatic groups in the 3- or 4-position including isopropyl or *t*-butyl substituents (**54**–**56**) showed a moderate increase in activity (EC_50_ 0.06–0.11 μM). Polar groups in this position generally decreased activity. For example, methoxy in the 2-, 3- or 4- position (**57**–**59**) gave a modest reduction in activity (EC_50_ 0.23–0.49 μM), while groups with similar polarity and greater steric bulk, including nitrile, dimethylamine, *N*-acetamide, acyl or methyl hydroxy groups in these positions (**60**–**71**) typically exhibited more significant reductions in activity (EC_50_ 0.32 – >10 μM). Collectively, this cohort of analogs suggested that lipophilicity on this moiety was likely the key contributing factor to the activity and that no modification appeared to be taking part in a specific interaction with the molecular target. Nevertheless, the increased lipophilicity of these analogs did not contribute to human HepG2 cell cytotoxicity (CC_50_ > 40 μM).

**Table 3:**
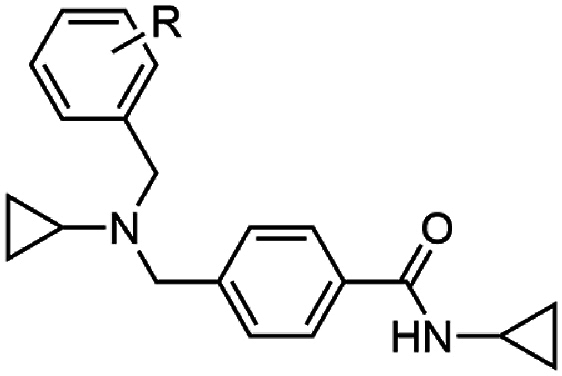
Activities of aryl substituted derivatives.

compound	R	Pf 3D7 EC_50_ (SD) μM^a^	HepG2 CC_50_ μM^b^	PSA (Å^2^)^c^	cLogP^c^
**17**	H	0.14 (0.01)	>40	32	3.4
**42**	2-F	0.25 (0.06)	>40	32	3.9
**43**	3-F	0.24 (0.09)	>40	32	3.9
**44**	4-F	0.22 (0.06)	>40	32	3.9
**45**	2-Cl	0.29 (0.03)	>40	32	3.9
**46**	3-Cl	0.34 (0.05)	>40	32	3.9
**47**	4-Cl	0.21 (0.08)	>40	32	3.9
**48**	2-Me	0.20 (0.04)	>40	32	3.9
**49**	2-CF_3_	0.22 (0.1)	>40	32	4.4
**50**	3-Me	0.17 (0.04)	>40	32	3.9
**51**	4-Me	0.15 (0.02)	>40	32	3.9
**52**	3-CF_3_	0.16 (0.01)	>40	32	4.4
**53**	4-CF_3_	0.11 (0.04)	>40	32	4.4
**54**	3-*i-*Pr	0.08 (0.05)	>40	32	4.7
**55**	4-*i*-Pr	0.11 (0.02)	>40	32	4.7
**56**	4-*t*-Bu	0.06 (0.02)	>40	32	5.2
**57**	2-OMe	0.27 (0.08)	>40	42	3.3
**58**	3-OMe	0.49 (0.07)	>40	42	3.3
**59**	4-OMe	0.23 (0.08)	>40	42	3.3
**60**	2-CN	0.12 (0.05)	>40	56	3.5
**61**	3-CN	0.13 (0.1)	>40	56	3.5
**62**	4-CN	0.63 (0.03)	>40	56	3.5
**63**	4-NMe_2_	0.53 (0.06)	>40	35	3.7
**64**	3-NMe_2_	0.32 (0.06)	>40	35	3.7
**65**	2-NHAc	7.4 (0.1)	>40	61	2.4
**66**	3-NHAc	>10	nd	61	2.4
**67**	4-NHAc	>10	nd	61	2.4
**68**	3-Ac	0.11 (0.1)	>40	49	2.8
**69**	4-Ac	0.90 (0.1)	>40	49	2.8
**70**	3-CH_2_OH	1.1 (0.1)	>40	53	2.9
**71**	4-CH_2_OH	0.17 (0.04)	>40	53	2.9

a EC_50_ data represents means and SDs for three or more independent experiments measuring LDH activity of *P. falciparum* 3D7 parasites over 72 h.

b CC_50_ data represents an average of two or more independent experiments measuring HepG2 cell growth inhibition over 48 h using Cell TitreGlo.

2,4-Dichloro substitution on the pendant aryl ring was present in the screening hit compound W499 (**2**) (EC_50_ 0.11 μM), and therefore di- and tri-substitution on the pendant ring was explored to improve anti-parasitic activity. Different combinations of difluoro and trifluoro substitution (**72**–**75**) did not improve activity (EC_50_ 0.22–0.23 μM) despite the increase in lipophilicity (cLogP 3.8–3.9) (Table 4). 3,4-Dichloro and 3,5-dichloro substitution (**76** and **77**) also did not improve activity (EC_50_ 0.21 and 0.23 μM), while the 2,4,6-trichloro substitution (**78**) showed similar activity (EC_50_ 0.10 μM) as W499 (**2**), signifying the 2,4-combination may be the most ideal substitution pattern. Different substitution patterns of methyl groups (**79**–**81**) also did not improve activity (EC_50_ 0.15–0.19 μM), while the 2,4,6-trimethyl substitution (**82**) exhibited similar activity (EC_50_ 0.08 μM). The same 2,4,6-trimethoxy substitution pattern (**83**) was not tolerated, reinforcing that polarity was not accepted on the pendant aryl ring. Conversely, the lipophilic 3,5-bis(trifluoromethyl) substitution (**84**) was equipotent to W499 (**2**) (EC_50_ 0.10 μM). The intolerance of polar groups was exemplified by the inclusion of an endocyclic nitrogen in the 2-, 3- and 4- positions of the pendant ring system (**85**–**87**), which were all detrimental to activity (EC_50_ 6.6 – >10 μM) (Table 5).

**Table 4:**
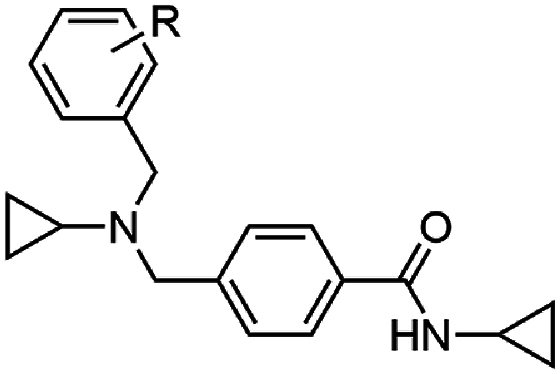
Activities of aryl di- and tri-substituted derivatives.

compound	R	Pf 3D7 EC_50_ (SD) μM^a^	HepG2 CC_50_ μM^b^	PSA (Å^2^)^c^	cLogP^c^
W466 (**1**)	2-Cl, 4-Cl	0.11 (0.01)	>40	32	4.6
**72**	2-F, 4-F	0.23 (0.03)	>40	32	3.8
**73**	3-F, 4-F	0.22 (0.08)	>40	32	3.8
**74**	3-F, 5-F	0.22 (0.07)	>40	32	3.8
**75**	2-F, 4-F, 6-F	0.19 (0.04)	>40	32	3.9
**76**	3-Cl, 4-Cl	0.21 (0.1)	>40	32	4.6
**77**	3-Cl, 5-Cl	0.23 (0.08)	>40	32	4.6
**78**	2-Cl, 4-Cl, 6-Cl	0.10 (0.02)	>40	32	5.1
**79**	2-Me, 3-Me	0.15 (0.00)	>40	32	4.4
**80**	2-Me, 4-Me	0.19 (0.02)	>40	32	4.4
**81**	3-Me, 5-Me	0.19 (0.08)	>40	32	4.4
**82**	2-Me, 4-Me, 6-Me	0.08 (0.01)	>40	32	4.9
**83**	2-OMe, 4-OMe, 6-OMe	2.5 (0.2)	>40	60	3.1
**84**	3-CF_3_, 5-CF_3_	0.10 (0.05)	15	32	5.3

a EC_50_ data represents means and SDs for three or more independent experiments measuring LDH activity of *P. falciparum* 3D7 parasites over 72 h.

b CC_50_ data represents an average of two or more independent experiments measuring HepG2 cell growth inhibition over 48 h using Cell TitreGlo.

**Table 5:**
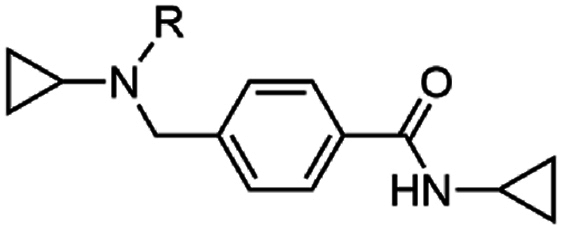
Activities of derivatives with N-substitutions on the central nitrogen.

compound	R	Pf 3D7 EC_50_ (SD) μM^a^	HepG2 CC_50_ (SD) μM^b^	PSA (Å^2^)^c^	cLogP^c^
**17**	CH_2_Ph	0.14 (0.01)	>40	32	3.4
**85**	-(CH_2_)-2-pyridyl	6.6 (1.5)	>40	45	2.5
**86**	-(CH_2_)-3-pyridyl	>10	nd	45	2.5
**87**	-(CH_2_)-4-pyridyl	9.2 (0.7)	>40	45	2.5
**88**	H	>10	nd	41	1.3
**89**	Me	>10	nd	32	1.7
**90**	*n-*Pr	2.5 (0.3)	>40	32	2.5
**91**	*i-*Pr	2.6 (0.5)	>40	32	2.5
**92**	*i-*Bu	0.40 (0.2)	nd	32	2.9
**93**	-(CH_2_)-*t-*Bu	0.46 (0.04)	>40	32	3.5
**94**	-(CH_2_)-cyBu	0.52 (0.1)	>40	32	2.9
**95**	-(CH_2_)-cyPen	0.51 (0.06)	>40	32	3.3
**96**	-(CH_2_)-cyHex	0.24 (0.09)	>40	32	3.7

a EC_50_ data represents means and SDs for three or more independent experiments measuring LDH activity of *P. falciparum* 3D7 parasites over 72 h.

b CC_50_ data represents an average of two or more independent experiments measuring HepG2 cell growth inhibition over 48 h using Cell TitreGlo. Cy = cyclo.

The importance of the pendant aryl ring was investigated. Deletion of this ring (**88** and **89**) was not tolerated (EC_50_ > 10 μM) (Table 5). Replacing the *N*-benzyl substitution with small aliphatic groups (**90** and **91**) was also not tolerated (EC_50_ 2.5 and 2.6 μM) while a larger isobutyl group (**92**) was approximately 4-fold less active (EC_50_ 0.54 μM) than **17**. Incorporating aliphatic groups of increasing size (*t*-butyl, cyclobutyl, cyclopentyl, and cyclohexyl) (**93**–**96**) led to incremental increases in activity (EC_50_ 0.24–0.52 μM), again demonstrating that a combination of steric bulk and lipophilicity in this region were drivers of antiparasitic activity.

Modification of the two carbons adjacent to the central nitrogen was next explored. Methylation of the carbon next to the pendant aryl ring (**97**) maintained antiparasitic activity (EC_50_ 0.17 μM) relative to **17**, while methylation of the carbon adjoining the carboxamide aryl ring (**98**) resulted in a 2-fold decrease in activity (EC_50_ 0.34 μM) (Table 6). Incorporation of a carbonyl group on either alpha carbon (**99** and **100**) was not tolerated (EC_50_ > 10 μM), while a sulfonyl group at either position (**101** and **102**) was also detrimental to activity (EC_50_ 2.3 and 4.8 μM). Notably, sulfonamide functionality was a component of the hit compound W466 (**1**) (Fig. 1) which was approximately 10-fold more active than these analogs due to the contribution from the 2,4,6-trimethyl substitution on the pendant aryl group. This data implied that modification of the alpha carbons was not beneficial to activity, and therefore this derivatization was not further pursued.

**Table 6:**
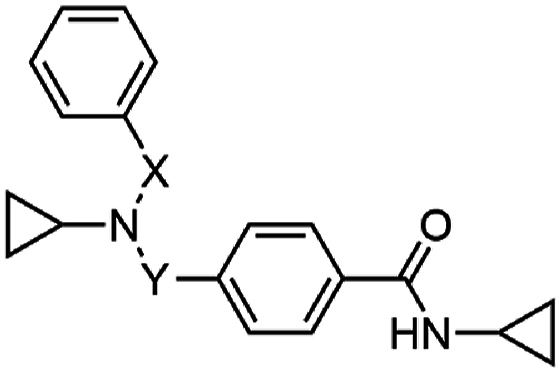
Activities of analogs with modification of the alpha carbons.

compound	X	Y	Pf 3D7 EC_50_ (SD) μM^a^	HepG2 CC_50_ μM^b^	PSA (Å^2^)^c^	cLogP^c^
**17**	CH_2_	CH_2_	0.14 (0.01)	>40	32	3.4
**97**	C(CH_3_)	CH_2_	0.17 (0.02)	>40	32	3.8
**98**	CH_2_	C(CH_3_)	0.34 (0.01)	>40	32	3.8
**99**	C(O)	CH_2_	>10	nd	49	2.9
**100**	CH_2_	C(O)	>10	nd	49	2.9
**101**	SO_2_	CH_2_	2.3 (0.5)	>40	67	2.8
**102**	CH_2_	SO_2_	4.8 (0.2)	>40	67	2.8

a EC_50_ data represents means and SDs for three or more independent experiments measuring LDH activity of *P. falciparum* 3D7 parasites over 72 h.

b CC_50_ data represents an average of two or more independent experiments measuring HepG2 cell growth inhibition over 48 h using Cell TitreGlo.

Lipophilicity appeared to be largely governing the antiparasitic activity of the aryl amino methyl series. In pursuit of increasing polarity, the inclusion of an endocyclic nitrogen was attempted in the carboxamide aryl group. An endocyclic nitrogen in either the 2- or 3-positions relative to the carboxamide aryl ring (**103** and **104**) was detrimental to activity (EC_50_ 7.6 and 3.5 μM) (Table 7). The activity (EC_50_ 2.4 and > 10 μM) was similarly lessened when the aryl ring was replaced with two possible configurations of a thiazole ring (**105** and **106**). Notably, a 2-pyridyl group was found in the structurally related compound MMV024397 (**4**) (Fig. 1), which is reported to have modest parasite activity [17].

**Table 7:**
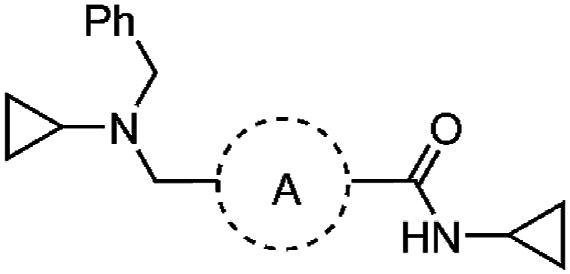
Activities of derivatives with variations to cyclic core.

compound	A	Pf 3D7 EC_50_ (SD) μM^a^	HepG2 CC_50_ μM^b^	PSA (Å^2^)^c^	cLogP^c^
**17**		0.14 (0.01)	>40	32	3.4
**103**		7.6 (1.3)	>40	45	2.5
**104**		3.5 (0.7)	>40	45	2.5
**105**		2.4 (0.08)	>40	45	2.9
**106**		>10	nd	45	2.9

a EC_50_ data represents means and SDs for three or more independent experiments measuring LDH activity of *P. falciparum* 3D7 parasites over 72 h.

b CC_50_ data represents an average of two or more independent experiments measuring HepG2 cell growth inhibition over 48 h using Cell TitreGlo.

We next combined the most active moieties in a set of four compounds. Notably, the *n*-propyl was chosen as the central nitrogen substituent as it (analog **26**) had the most reasonable balance of antimalarial activity, aqueous solubility and metabolic stability (see section on ADME analysis). These compounds incorporated a cyclopropyl carboxamide and *n*-propyl substitution of the central basic nitrogen while varying substitution on the pendant aryl ring. These four compounds (**107**–**110**) exhibited potent asexual stage activity (EC_50_ 0.02–0.08 μM) and did not exert adverse HepG2 cell cytotoxicity (CC_50_ > 40 μM) (Table 8). A defining physicochemical feature of these compounds is the high lipophilicity (cLogP 4.9–5.7) and low PSA (32 Å^2^). The high cLogP and low PSA is a characteristic feature of cyt *b* inhibitors (for example, ELQ300: cLogP 5.7 and PSA 57 Å^2^) and represents the largely hydrophobic Q_i_ and Q_o_ binding sites that accept the lipophilic substrates, ubiquinone or ubiquinol.

**Table 8:**
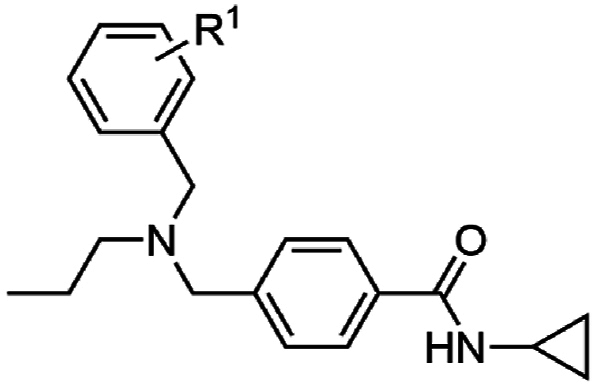
Activity of analogs with a combination of substituents.

compound	R^1^	Pf 3D7 EC_50_ (SD) μM^a^	HepG2 CC_50_ μM^b^	PSA (Å^2^)^c^	cLogP^c^
**107**	4-*t*-Bu	0.02 (0.01)	>40	32	5.7
WJM280 (**108**)	4-CF_3_	0.04 (0.01)	15	32	4.9
**109**	3-CF_3_, 5-CF_3_	0.03 (0.01)	10	32	5.8
**110**	2-Me, 4-Me, 6-Me	0.08 (0.03)	>40	32	5.4

a EC_50_ data represents means and SDs for three or more independent experiments measuring LDH activity of *P. falciparum* 3D7 parasites over 72 h.

b CC_50_ data represents an average of two or more independent experiments measuring HepG2 cell growth inhibition over 48 h using Cell TitreGlo.

### 2.3 *In vitro* ADME analysis

The physicochemical properties of the cyclopropyl carboxamide analogs, high cLogP, low PSA, and tertiary basic nitrogen did not lend favorably to high metabolic stability. This was reflected in the low metabolic stability of the hit compound W499 (**2**) in human liver microsomes and rat hepatocytes (human liver microsomes CL_int_ 177 μL/min/mg and rat hepatocytes CL_int_ > 92 μL/min/10^6^ cells) (Table 9). To determine the functionality responsible for the high metabolic turnover, metabolite identification was performed on W499 (**2**) after incubation in mouse liver microsomes. It was shown that there were three products from oxidative dealkylation of the central tertiary nitrogen, while oxidative addition and dealkylation of both cyclopropyl groups were also detected (Table S1 and Fig. S2). Metabolic turnover of N-cyclopropyl groups by cytochrome P450s has been shown to produce a ring opened hydrated metabolite, through a mechanism putatively causes irreversible inhibition of cytochrome 450 enzymes [27]. Modifying the cyclopropyl substitution on the central nitrogen to a cyclobutyl group (**22**) did not alter the metabolic stability (CL_int_ 151 μL/min/mg), while isopropyl, trifluoroethyl and *n*-propyl, and (**21**, **25** and **26**) appeared to enhance metabolic stability in human liver microsomes (CL_int_ 76–98 μL/min/mg), but not in rat hepatocytes (CL_int_ > 92 μL/min/10^6^ cells). Methyl substitution on the alpha carbon (**97**) which we reasoned could block oxidative metabolism, did not improve metabolic stability in human microsomes (CL_int_ 238 μL/min/mg). A combination of a central *n*-propyl group and a trifluoromethyl functionality on the pendant ring with analogs **108** and **109** gave a modest improvement in metabolic stability (CL_int_ 62 and 65 μL/min/mg) while the analog **107** had significantly lower metabolic stability (CL_int_ 263 μL/min/mg), presumably due to the added lipophilicity and metabolic susceptibility of the *tert*-butyl group. Notably, **109** was the only analog that gave measurable metabolic stability in rat hepatocytes (CL_int_ 60 μL/min/10^6^ cells). The aqueous solubility at pH 7.4 was highly variable (7.4–140 μM) but the lower eLogD values of analogs generally correlated with higher aqueous solubility. Overall, the metabolic stability of the cyclopropyl carboxamide series is not ideal and therefore it would be expected that systemic exposure would be short, lending to low efficacy in a mouse model of malaria. The SAR and the *in vitro* ADME data suggest that it will be challenging to mitigate metabolic stability while maintaining the asexual stage activity, suggesting further development of this antimalarial series may not be feasible.

**Table 9:** Aqueous solubility, *in vitro* metabolism, and eLogD parameters for representative compounds.

compound	aqueous solubility pH 7.4 (μM)^a^	human liver microsomes CL_int_ (μL/min/mg)	rat hepatocytes CL_int_ (μL/min/10^6^ cells)	eLogD^b^
W499 (**2**)	7.4	177	>92	4.1
**17**	44	72	>92	4.0
**21**	140	76	>92	3.5
**22**	92	151	>92	3.8
**25**	11	79	>92	4.8
**26**	83	98	>92	3.7
**97**	22	238	>92	4.2
**107**	10	263	92	4.9
WJM280 (**108**)	15	62	>92	5.2
**109**	2.5	65	60	5.0

a Kinetic in PBS.

b Shake-flask method.

### 2.4 Resistance selection and whole genome sequencing

**Fig. 2:**
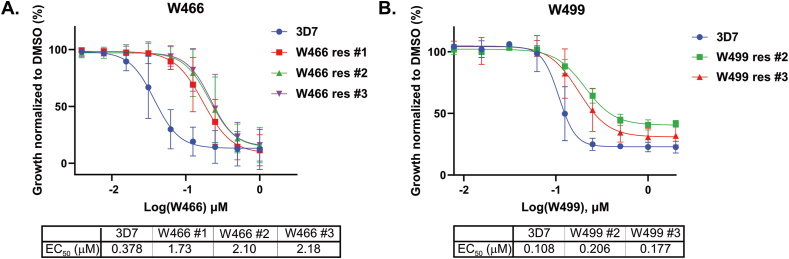
Activity of W466 (**1**) and W499 (**2**) against W466**-** and W499 -resistant populations respectively. W466-resistant population 1 has an A122T cyt *b* Q_o_ site mutation and populations 2 and 3 have an F264L cyt *b* Q_o_ site mutation. W499-resistant populations 2 and 3 have a 2- to 5-fold CNV in region that encodes DHODH. EC_50_ values represent an average of 3 experiments using the LDH assay. Error bars are SD.

To determine the mechanism of action of the cyclopropyl carboxamide series, a forward genetic resistance study was undertaken on the two hit compounds W466 (**1**) and W499 (**2**). To generate resistant parasites, asynchronous asexual 3D7 parasite cultures were treated with compounds starting from approximately two times the EC_50_ (0.50 μM for W466 (**1**) and 0.26 μM for W499 (**2**)). A cycle of compound treatment and removal was repeated with increasing concentrations of compound, which was continued until the compound concentration reached approximately 10 times the EC_50_ for W466 (**1**) (2.5 μM) and W499 (**2**) (1.3 μM). A 72-h growth assay was then performed to determine the EC_50_ of the resistant parasites. This revealed that the three W466 (**1**) selected populations had 4.5–5.7-fold shift in EC_50_ and the two W499 (**2**) selected populations had a 1.6–1.9-fold shift in EC_50_ compared to wildtype parasites, indicating that resistance was generated (Fig. 2).

To identify genetic determinants that are responsible for parasite resistance caused by W466 (**1**) and W499 (**2**), genomic DNA was prepared and whole genome sequencing was performed (Tables S2 and S3). In the W466-resistant populations, SNPs were identified across 5 genes including PF3D7_MIT02300 (*cyt b* from the cyt *bc*1/complex III), *liver stage antigen 1*, PF3D7_1471900, SOC2 and BSD-domain protein, PF3D7_0305500 and PF3D7_1406700 (Table S4). Of these, only *cyt b* had variants across all resistant populations whereby population 1 contained a variant resulting in a A122T mutation and population 2 and 3 contained a variant resulting in a F264L mutation (Table S5). These SNPs were present in 98 % and >95 % of the population in the selected parasites, respectively (Table S4). A copy number variation event was also found in chromosome 02 in W466-resistant populations #1 and #3. All samples, including the parent 3D7 sample, had a deletion compared to the reference from the 5′ end to 106 kb. This deletion continued subclonally in the parent 3D7 and fully in population #2–110 kb but not in populations #1 and #3 (Figs. S3 and 4).

**Fig. 3:**
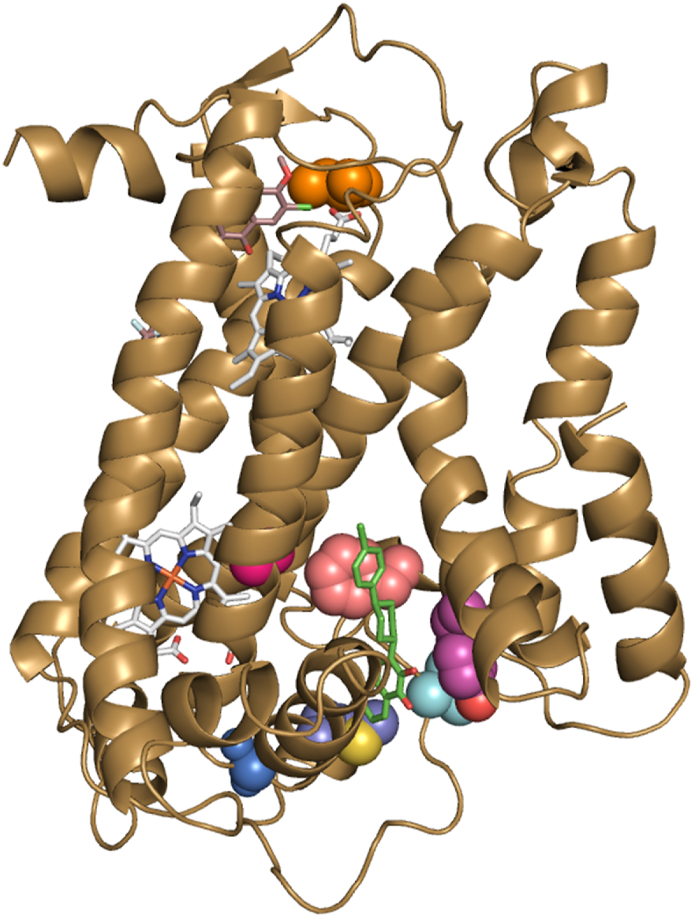
A homology model of *P. falciparum* cyt *b* showing the A122T Q_o_ site mutation (red) found in 3D7 W466-resistant population 1, the F246L Q_o_ site mutation (pink) found in 3D7 W466-resistant populations 2 and 3 and Dd2-Polδ W466-resistant F3 clones (from MIR study), and the G131S Q_o_ site mutation (blue) found in the Dd2 W466-resistant population H3 (from MIR study), relative to the location of ELQ300, 3-oxadiazole quinolone and ATQ *P. falciparum* resistant mutations (used in Table 12). The homology model of *P. falciparum* cyt *b* was created from the X-ray structure of *Gallus gallus* cyt *bc*_1_ (PDB: 3H1I) [28]. TM90-C2B (ATQ resistant) strain cyt *b* Y268S Q_o_ site mutation is shown in magenta; ELQ300 resistant Dd2 strain cyt *b* I22L Q_i_ site mutation (orange); 3-oxadiazole quinolone resistant 3D7 strain cyt *b* V259L Q_o_ site mutation [8] (cyan). Heme molecules (grey), ATQ (green) and ELQ300 (brown) are overlayed using previous structural data [22,32].

The cyt *b* SNPs detected in W466**-**resistant populations were mapped to a homology model of *P. falciparum* cyt *b* created from an X-ray structure of *Gallus gallus* cyt *bc*1 (PDB: 3H1I) [28]. This showed that the A122T and F264L mutations are both located in the Q_o_ site of cyt *b* which is the binding site of ATQ, but distal to the Q_i_ site which is the binding site of ELQ300 (Fig. 3). The A122T mutation has been previously detected in parasites resistant to the quinolone antimalarials, decoquinate, and CK-2-68 [[29], [30]], while the F264L mutation was previously found in parasites resistant to tetracyclic benzothiazepines [31], and the quinolone RYL552 [29]. These compounds have been shown to target cyt *b*, providing supporting evidence that the cyclopropyl carboxamide series also targets cyt *b*.

Sequencing of W499-resistant populations identified a 2 to 5-fold copy number variation (CNV) in the 113 kb–156 kb region of chromosome 6 (Figs. S5 and S6, Tables S6 and S7). Notably, this region of chromosome 6 encodes a putative mitochondrial chaperone BCS1 (PF3D7_0603200) and DHODH (PF3D7_0603300). This amplification event in DHODH has been seen in other inhibitors of cyt *b* and is likely a compensatory mechanism to overcome disruption to the mitochondria electron transport chain [24]. Overall, the evidence from the sequencing the W499**-** and W466-resistant populations supports the mechanism of action of this chemical series in the mitochondrial transport chain function.

### 2.5 Minimum inoculum of resistance

Antimalarials with a high barrier to resistance are desirable from a development and TCP standpoint. The minimum inoculum of resistance (MIR) is an assay commonly used to measure the rate at which parasites acquire resistance to antimalarials under development [33]. We used the MIR assay to determine the rate of resistance for W466 (**1**) or W499 (**2**). For this assay, two selections, both using 2 x 10^5^ Dd2-B2 parasites in each well of a 96-well plate, were treated with either W466 (**1**) or W499 (**2**) at three times their asexual IC_90_ (2.13 μM for W466 (**1**) and 0.67 μM for W499 (**2**)). The selections had consistent drug pressures of 3 × IC_90_ for 60 days and parasite recrudescence was monitored by flow cytometry using SYBR Green and MitoTracker Deep Red FM.

No parasite recrudescence for W499 (**2**) was observed throughout this selection and therefore had a calculated MIR of >2 × 10^7^ (Table S8). Parasite recrudescence was detected in one well (H3) for W466 (**1**), which showed a 4.4-fold shift in the IC_50_ (Fig. S7 and Table S10 and resulted in an MIR of 1.9 × 10^7^ (Table S9). The resistant H3 population was subsequently expanded and then whole genome sequenced to detect the genetic determinants responsible for the recrudescence in the H3 well. Sequencing revealed a single non-synonymous SNP encoding G131S in cyt *b* (Tables S12 and S13). The G131S mutation is located in the Q_o_ site of cyt *b* (Fig. 3) which is consistent with the mutations located in the Q_o_ site uncovered from above forward genetic study on W466 (**1**). The G131S mutation was also detected in Dd2 parasites resistant to tetracyclic benzothiazepine antimalarial class [31], which curiously, along with W466 (**1**), was a compound class that also has reduced activity to parasites with a F264L mutation in cyt *b*.

To further investigate the resistance potential of W466 (**1**) and support cyt *b* as the genetic determinant of resistance for this chemical series, an additional selection was performed using two cultures with 3.3 × 10^8^ Dd2-B2 parasites each, and one culture with 3.3 × 10^8^ Dd2-Polδ parasites, all at a starting concentration of 3 × IC_90_ (2.13 μM). The hypermutable *P. falciparum* line, Dd2-Polδ, contains mutations in the gene encoding the DNA polymerase delta subunit, which impair the protein's proofreading function [34]. Recrudescence was not detected for the two Dd2-B2 parasite cultures, but was observed for the Dd2-Polδ parasite culture (F3 population). The recrudescent F3 population was then profiled against W466 (**1**), revealing a 12 × IC_50_ shift. The F3 bulk culture was then cloned under 3 × IC_90_ drug pressure using a limiting dilution protocol at a concentration of 0.5 infected RBCs per well in a 96-well plate. Three clones (F3_A6, F3_C9, and F3_H4) were picked for phenotyping and revealed IC_50_ fold-shifts ranging from 13.1 to 15.2 (Fig. S8 and Table S11).

The genomic DNA from three W466**-**resistant Dd2-Polδ F3 clones, F3_C9, F3_A6 and F3_H4, were whole genome sequenced. Sequencing revealed a non-synonymous SNP encoding F264L in cyt *b* in all three Dd2-Polδ clones (Tables S14–S16). The data on these three clones corroborates the F264L mutation detected in the Q_o_ site of cyt *b* discovered in the above pulsed forward genetic study on W466 (**1**) (Fig. 2, Fig. 3). In addition to the F264L mutation, several other SNPs were detected in the Dd2-Polδ F3_A6 and F3_H4 clones, but not the F3_C9 clone. These SNPs included missense mutations in PF3D7_0607500 (R82H), PF3D7_0628100 (Q5197K), PF3D7_0905900 (S496F), and PF3D7_1022800 (L674F) (Tables S15–S16). PF3D7_0607500 encodes a putative 4-hydroxybenzoate polyprenyltransferase, PF3D7_0628100 encodes a HECT-domain (ubiquitin-transferase), PF3D7_0905900 encodes a putative coatomer subunit beta, and PF3D7_1022800 encodes 4-hydroxy-3-methylbut-2-en-1-yl diphosphate synthase. Of note, three SNPs were shared between the two clones: PF3D7_0628100 (Q5197K), PF3D7_0905900 (S496F), and a missense mutation in PF3D7_1021200 (S357F), which encodes a conserved *Plasmodium* protein of unknown function. In addition to these three mutations, F3–H4 also harbored a missense mutation in PF3D7_1106500 (F1762Y), which encodes another conserved *Plasmodium* protein of unknown function.

It is not entirely clear whether there is a direct link between the mutations found in the W466**-**resistant Dd2-Polδ F3_A6 and F3_H4 clones and the mutations found in cyt *b*. 4-Hydroxybenzoate polyprenyltransferase is an enzyme putatively involved in the biosynthesis of ubiquinone [35] required for the function of the mitochondrial electron transport chain. 4-Hydroxy-3-methylbut-2-en-1-yl diphosphate synthase is an essential component of the isoprenoid biosynthesis pathway which is necessary for supplying precursors for ubiquinone biosynthesis [36]. The mutations in these proteins may be a compensatory mechanism for the inhibition of cyt *b* by W466 (**1**). The role of these mutations on the function of these enzymes is unknown and requires reverse genetics to delineate. The role or the survival advantage of the mutations found in the other proteins of W466-resistant Dd2-Polδ F3_A6 and F3_H4 clones on the function cyt *b* or the mitochondrial electron transport chain is unclear and therefore further investigation is required. Overall, the whole genome sequencing on the W466 recrudescence parasites from the 10.13039/100023938MIR study supports cyt *b* as the target of the cyclopropyl carboxamide antimalarial series.

### 2.6 Evaluation against W466- and W499-resistant parasites

The structures of W466 (**1**) and W499 (**2**) are slightly different although they appear to belong to the same structural class. To confirm if W466 (**1**) and W499 (**2**) may have the same molecular target, W466 (**1**) and W499 (**2**) were each evaluated for cross resistance against their respective resistant populations from the pulsed forward genetic study (Fig. 2). The results show that W499 (**2**) has a 2-fold reduction in sensitivity (EC_50_ 0.22–0.28 μM) to W466-resistant parasites compared to the wildtype 3D7 parasites (Table 10 and Fig. S9), while W466 (**1**) has a 1.5 to 2-fold reduction in activity (EC_50_ 0.50 and 0.62 μM) against W499-resistant parasites (Table 11 and Fig. S10). This data implies that W466 (**1**) and W499 (**2**) are both likely share the same mechanism of resistance of interfering with cyt *b* function.

**Table 10:** Activity of W499 (**2**), ATQ, ELQ300 and DSM-265 against W466-resistant populations.

compound	Pf 3D7 EC_50_ (SD) nM a	W466-resistant populations EC_50_ (SD) nM a^,^^b^		
		#1	#2	#3
W499 (**2**)	170 (51)	283 (31)	243 (43)	215 (77)
ATQ	1 (<1)	2 (<1)	1 (<1)	1 (<1)
ELQ300	13 (7)	36 (15)	18 (12)	20 (14)
DSM265	53 (57)	19 (19)	13 (16)	13 (16)

a EC_50_ values represent an average of 3 experiments using the LDH assay.

b Population 1 has a A122T cyt *b* Q_o_ site mutation and populations 2 and 3 have a F264L cyt *b* Q_o_ site mutation.

**Table 11:** Activity of W466 (**1**), ATQ, ELQ300 and DSM-265 against W499-resistant populations.

compound	Pf 3D7 EC_50_ (SD) nM^a^	W499-resistant populations EC_50_ (SD) nM^a^^,^^b^^,^^c^	
		#2	#3
W466 (**1**)	321 (77)	620 (81)	500 (98)
ATQ	2 (1)	4 (1)	3 (1)
ELQ300	23 (7)	53 (4)	45 (12)
DSM265	92 (13)	>1000	>1000

a EC_50_ values represent an average of 3 experiments using the LDH assay.

b SYBR-green was used to evaluate DSM265.

c Population #2 and #3 has a 2- and 5-fold copy number variation (CNV) in a region of the genome that encodes DHODH.

Next, the cyt *b* inhibitors ATQ and ELQ300, and the DHODH inhibitor DSM265 were evaluated against W466-resistant and W499-resistant populations. No significant difference in activity (EC_50_ 0.001 and 0.013 μM) was observed with both ATQ and ELQ300 against the W466**-**resistant populations relative to the 3D7 parent strain (Table 10 and Fig. S9). It was expected that ELQ300 would not show reduced sensitivity to the W466-resistant population because the mutations in cyt *b* are in the Q_o_ site (Fig. 3), distal to where ELQ300 is known to bind in the Q_i_ site of cyt *b* [23]. ATQ binds in the Q_o_ site and typically mutations in this site would sensitize the activity of ATQ, but this was not observed for the W466-resistant populations that have either a A122T or a F264L mutation in the Q_o_ site of cyt *b*. This is an intriguing result and suggests the locations of these mutations (Fig. 3) do not impact ATQ binding to the Q_o_ site of cyt *b*. This result was also confirmed by other studies showing that both these mutations do not cause ATQ to have reduced activity [29,30]. Expectedly, DSM265 showed similar activity against the W466-resistant populations compared to the parent 3D7 strain (Table 10).

ATQ and ELQ300 both showed a 2-fold reduction in activity (EC_50_ 0.003 and 0.004; 0.045 and 0.053 μM) against the W499-resistant populations relative to the wildtype 3D7 strain, while DSM265 exhibited a >10-fold decrease in potency (EC_50_ > 1.0 μM) (Table 11 and Fig. S10). This result is consistent with the W499-resistant populations having a 2- to 5-fold amplification in DHODH, which is required for the function of the mitochondrial electron transport chain. Collectively, this data provides evidence supporting cyt *b* as the target of the cyclopropyl carboxamide series.

### 2.7 Evaluation against cyt *b* drug-resistant parasites

To determine whether the cyclopropyl carboxamide series specifically targets the Q_o_ site of cyt *b*, representative analogs W466 (**1**), W499 (**2**), and the focal compound here-on referred to as WJM280 (**108**) were evaluated against drug-resistant parasite strains with mutations in the Q_o_ or Q_i_ site of cyt *b*. These drug-resistant parasite strains included the ATQ and oxadiazole quinolone resistant population with a V259L mutation in the Q_o_ site of cyt *b* [8], and the ELQ300 resistant strain with an I22L mutation in the Q_i_ site of cyt *b* [37]. These mutations are mapped to a model of cyt *b* in Fig. 3.

It was found that all cyclopropyl carboxamide analogs W466 (**1**), W499 (**2**), and WJM280 (**108**) exhibited similar activity (EC_50_ 0.065, 0.049 and 0.002 μM) toward the ELQ300 resistant strain relative to the parent Dd2 parasite line (EC_50_ 0.070, 0.118 and 0.010 μM) (Table 12 and Fig. S11), indicating that the cyclopropyl carboxamide series does not target the Q_i_ site of cyt *b*. However, against the cyt *b*^V259L^ Q_o_ site mutant W466 (**1**) showed a 6-fold reduction in activity (EC_50_ 0.420 μM) while W499 (**2**), and WJM280 (**108**) showed a 1.5- and 2-fold reduction in activity (EC_50_ 0.165 and 0.023 μM) compared to the Dd2 parent strain. This was a similar result to ATQ that had an approximate 10-fold reduction in activity (EC_50_ 0.013 μM) and is known to bind to the Q_o_ site of cyt *b*. This data supports the activity data on the Q_o_ site mutations found in W466-resistant populations and infers that the cyclopropyl carboxamide class targets the Q_o_ site of cyt *b*.

**Table 12:** Evaluation of selected compounds against *P. falciparum* asexual parasites resistant to mitochondria targeted drugs or expressing ScDHODH.

compound	Pf Dd2 EC_50_ (SD) μM^a^	Pf cyt *b* mutant strains EC_50_ (SD) μM^a^		SB1-A6 EC_50_ (SD) μM^b^	Dd2 ScDHODH EC_50_ (SD) μM^b^
		Dd2 ^cyt^^b^^(I22L)^	3D7 ^cyt^^b^^(V259L)^		
W466 (**1**)	0.070 (0.008)	0.065 (0.009)	0.420 (0.013)	>10	>10
W499 (**2**)	0.118 (0.072)	0.049 (0.023)	0.165 (0.082)	>10	>10
WJM280 (**108**)	0.010 (<0.001)	0.002 (<0.001)	0.023 (0.002)	>10	>10
ATQ	0.001	5.4	0.013	>10	>10
ELQ300	0.021	0.176	0.012	–	–
DSM265	0.024	0.008	–	>10	>10

a EC_50_ values represent an average of 3 experiments against the Pf Dd2 parental line, or Pf Dd2 strain with an I22L mutation in the Q_i_ site cyt *b*^37^ or Pf 3D7 3-oxadiazole quinolone resistant strain with a V259L mutation in the Q_o_ site of cyt *b*^8^ over 72 h measuring SYBR green by FACS.

b EC_50_ values represent an average of 3 independent experiments against the Pf SB1-A6 strain and a C276F mutation in DHODH [24] or Pf Dd2 expressing ScDHODH [18] over 72 h measuring SYBR green by FACS. Dose response curves with error values are shown in Fig. S11. Data for known drug controls, ATQ, ELQ300, and DSM265 taken from Nguyen, Dans et al. [8].

### 2.8 Evaluation against DHODH resistant and ScDHODH expressing parasites

DHODH is essential for the function of the mitochondria ETC and pyrimidine biosynthesis. Compounds that inhibit cyt *b* typically have reduced activity against parasite strains with increased DHODH levels. To support evidence that the cyclopropyl carboxamide series impacts the mitochondrial ETC, selected analogs were evaluated against both the SB1-A6 strain that has a 2-fold amplification and a C276F SNP in PfDHODH [24] and a Dd2 strain that expresses *Saccharomyces cerevisiae* DHODH (ScDHODH) [18]. ScDHODH has low sequence homology to PfDHODH and is ubiquinone-independent in that it uses fumarate instead of mitochondrial coenzyme Q as an electron acceptor and therefore compounds that inhibit the *Plasmodium* mitochondrial ETC have reduced activity against the ScDHODH parasite strain. It was shown that the hit compounds W466 (**1**) and W499 (**2**), and WJM280 (**108**) are inactive against both the SB1-A6 strain and ScDHODH Dd2 strain (EC_50_ > 10 μM) in comparison to the parent Dd2 parasite strain (EC_50_ 0.070, 0.018 and 0.010 μM) (Table 12 and Fig. S11). This result reflects the decrease in activity shown by both ATQ and DSM265 against both these strains (EC_50_ > 10 μM). This data supports the cyclopropyl carboxamide series impacting the ETC through inhibition of cyt *b*.

### 2.9 Electron transport chain analysis

**Fig. 4:**
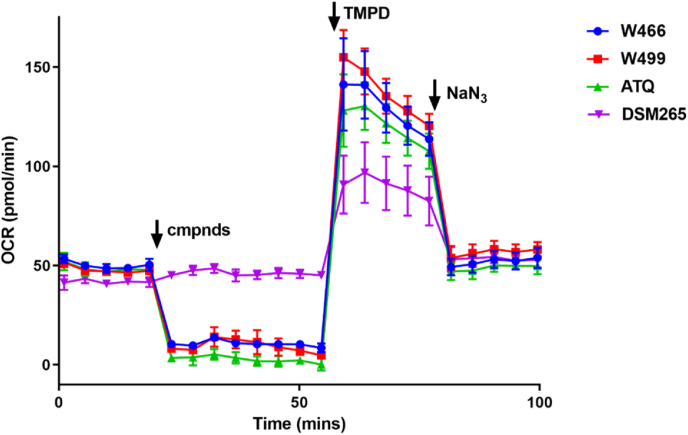
*P. falciparum* oxygen consumption rate (OCR) assay. Data represent the means and SDs of 3 technical replicates. Time points and reagent injections were as follows, 1. First 5 timepoints measured the basal level of malate-dependent OCR; 2. Compounds (5 μM) were injected independently (indicated by arrow), and OCR measured for 8-timepoints; 3. TMPD (a cytochrome *c* electron donor) was injected, and OCR measured for 5 time points; 4. NaN_3_ (a complex IV inhibitor) was then added, and OCR measured for 5 time points. ATQ = atovaquone and DMS265 is a DHODH inhibitor.

To provide further evidence to support the cyclopropyl carboxamide series targeting cyt *b*, an oxygen consumption rate (OCR) assay was performed using a Seahorse XFe96 Flux Analyzer [17]. The primary role of the mitochondria ETC is to produce ATP oxidative phosphorylation, and thus the oxygen consumption directly quantifies mitochondrial ETC activity. In this assay, malate, which is a substrate of malate quinone dehydrogenase (MQD), was added to provide a base line of oxygen consumption at approximately 50 pmol/min (Fig. 4). After adding atovaquone (ATQ) which inhibits cyt *b* (complex III), an OCR reduction was detected while the DHODH inhibitor, DSM265 didn't change the basal OCR. Both W466 (**1**) and W499 (**2**) reduced the OCR to the same level as ATQ. Tetramethylphenylendiamin (TMPD), an electron donor was then added which recovered the OCR, indicating none of the compounds inhibit cyt *c*. Finally, NaN_3_ was added to inhibit complex IV to bring the OCR back to basal levels. The same OCR pattern between ATQ and both W466 (**1**) and W499 (**2**) supports cyt *b* as the target of cyclopropyl carboxamide series. This data also suggests that the amplification of DHODH is a compensatory mechanism of W466- and W499-resistant populations and is not the molecular target of the cyclopropyl carboxamide class.

### 2.10 Asexual stage of arrest and parasite reduction ratio

**Fig. 5:**
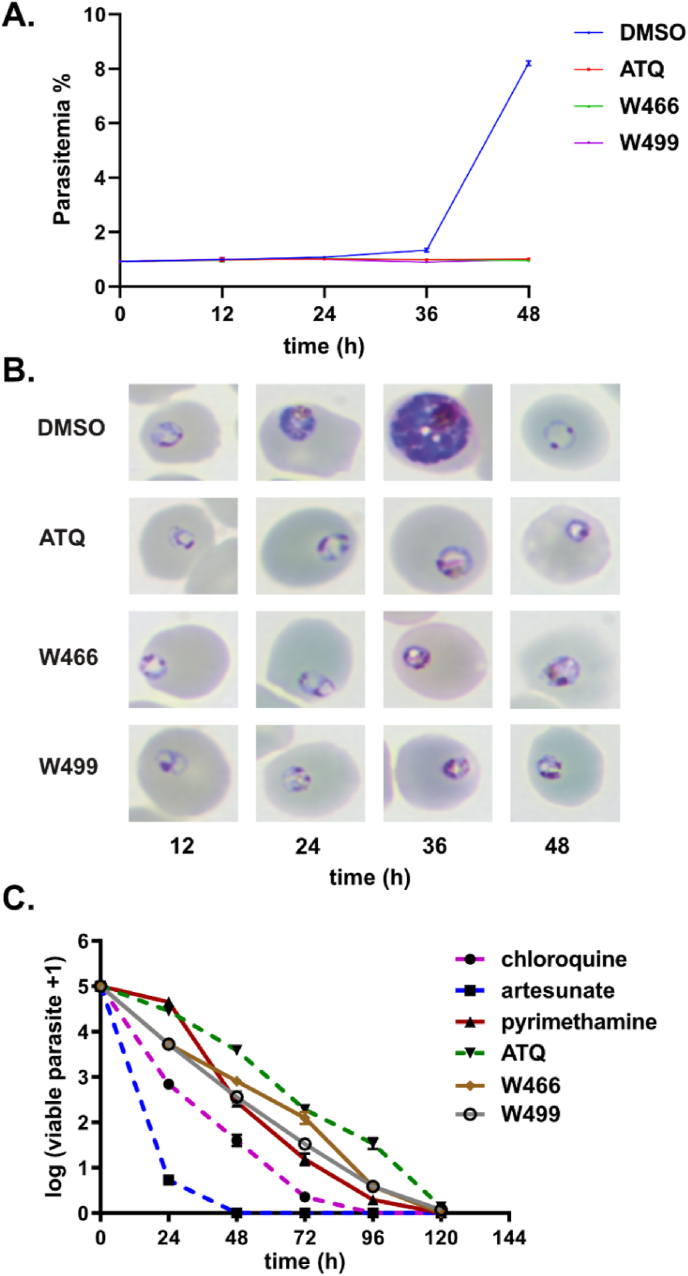
**A.** Representative Giemsa-stained microscopy images showing the asexual stage of arrest on treatment with ATQ, W466 (**1**) and W499 (**2**). Other representative images can be found in Fig. S12. **B.** Flow cytometry of SYBR green-stained infected RBCs. Data points represent the mean of three technical replicates analyzed *via* flow cytometry. Compounds in these experiments were used at a concentration of 10 times the asexual EC_50_ value. **C.** Activity of W466 (**1**) and W499 (**2**) in a parasite reduction ratio assay in comparison to antimalarial drugs. Data represent the means and SDs of 3 replicate experiments using *P. falciparum* 3D7 parasites in a LDH assay. ATQ = atovaquone.

Characterization of asexual stage phenotype and rate of asexual kill is important in determining the downstream application and TCP of an antimalarial series [2]. The asexual stage of arrest assay was performed using highly synchronized ring-stage *P. falciparum* parasites treated with W466 (**1**) and W499 (**2**) at ten times their EC_50_ (1.3 and 2.5 μM). We also included ATQ as a benchmark cyt *b* inhibitor which was used at 10 times its EC_50_ (11.7 nM). Parasites were sampled every 12 h for 48 h, to prepare Giemsa-stained blood smears for analysis by microscopy. Parasitemia of each sample was measured by flow cytometry using SYBR-Green DNA staining. The results show that the DMSO control group advanced through one asexual stage lifecycle in 48 h. Both W466 (**1**) and W499 (**2**) treatment leads to parasite arrest at the start of the trophozoite stage (24 h) in the first lifecycle stage, which is the same phenotype observed for ATQ treated parasites (Fig. 5A). The growth curve over the same time frame shows parasite levels are consistent with the microscopy results, whereby the parasites arrest at the trophozoite stage in the presence of W466 (**1**), W499 (**2**), and atovaquone (Fig. 5B and Fig. S12).

The parasite reduction ratio (PRR) assay is a benchmark assay used to determine the speed of kill of antimalarials at an early stage of development [38]. In the PRR assay, parasites are treated with the compound at ten times the EC_50_ (1.2 μM for W466 (**1**) and 2.8 μM for W499 (**2**)) at 24 h intervals over 120 h period, then the compound is washed out and replenished with uninfected RBCs and media. After 28 days of incubation, parasite growth is measured using an LDH assay. In the PRR assay, both W466 (**1**) and W499 (**2**) were shown to have a moderate to slow rate of kill similar to pyrimethamine and slightly quicker than atovaquone (Fig. 5C). The moderate to slow reduction ratio shown by the cyclopropyl carboxamide series is comparatively consistent with other mitochondrial ETC inhibitors, including ATQ.

### 2.11 Liver and transmission stage activity

Determination of liver stage and transmission stage activity is important for alignment with the TCP and potential application in a prophylaxis or population control therapy. The mitochondrial ETC chain is required for liver stage development and putatively required for transmission stage [[39], [40], [41]]. Like the asexual stage, the role of the mitochondria in liver stage development is the biosynthesis of coenzyme Q, therefore cyt *b* inhibitors, ATQ and ELQ-300 exhibit potent inhibition of exoerythrocytic forms (EEFs) [23,42]. To determine if the cyclopropyl carboxamide series has activity against EEFs, an assay was employed that uses human HepG2 cells that can be infected by *P. berghei* parasites [43]. In this assay protocol, the HepG2 cell line is pre-treated with compound before the addition of *P. berghei* sporozoites expressing luciferase. After a 48 h incubation, EEF viability was determined by measuring bioluminescence [42]. The hit compound, W466 (**1**), exhibited potent inhibition of EEF development (EC_50_ 0.07 μM) (Table 13), corroborating the EFF activity data observed with other cyt *b* inhibitors [8,23,44,45].

**Table 13:** *P. falciparum* liver stage and transmission activity of selected compounds.

compound	Liver stage		Transmission stage	
	Pb EEFs EC_50_ (SD) μM^a^	HepG2 EC_50_ (SD) μM^a^	male gamete EC_50_ μM^b^	female gamete EC_50_ μM^b^
W466 (**1**)	0.07 (0.03)	>50	1.1	>25
W499 (**2**)	nd	nd	0.76	>25
**107**	nd	nd	0.08	21.6
**110**	nd	nd	0.28	>25

a EC_50_ data represents means and SDs for 4 technical replicates following exposure to compounds in 10-point dilution series over 48 h using HepG2 as the host cell line.

b Data represents means from 4 replicate experiments using NF54 parasites in a DGFA. Dose response curves with error values are shown in Fig. S13.

In the transmission stage, cyt *b* inhibitors do not impact gametocytogenesis. In gametogenesis, the mitochondria are maternally acquired, and therefore microgametes (male gametes) do not have mitochondria [46]. Curiously, cyt *b* inhibitors such as atovaquone inhibit male gamete exflagellation but not female gamete formation [8,23,44], supporting evidence that mitochondrial glycolysis provides microgametes (male gametes) with an energy source for flagella formation and motility [47,48]. To determine whether the cyclopropyl carboxamide class has transmission blocking potential we profiled selected analogs in a dual gamete formation assay [41]. In this assay, stage V gametocytes are treated with compound, and gametogenesis is induced by the addition of xanthurenic acid. Male gamete viability is determined by an imaging algorithm measuring motile flagellation, while female viability is measured using an antibody to the female-specific Pfs25 surface marker. It was shown that the hit compounds W466 (**1**) and W499 (**2**) modestly inhibited male gamete viability (EC_50_ 1.1 and 0.76 μM), while the optimized analogs **107** and **110** were more potent (0.08 μM and EC_50_ 0.28), consistent with the activity trend observed with the asexual activity (Table 13 and Fig. S13). Compounds W466 (**1**), W499 (**2**), and **110** did not impact female gamete viability (EC_50_ > 25 μM), while **107** had a slight effect on viability (EC_50_ 21.6 μM), but it is unlikely this modest activity is due to cyt *b* inhibition. Collectively, the activity observed against male gametes is consistent with activity observed with other cyt *b* inhibitors [8,23,44].

### 2.12 Efficacy in a *P. berghei* 4-day mouse model

The 4-day *P. berghei* mouse model is used to determine the preliminary *in vivo* efficacy of antimalarials at an early stage of optimization. To determine if cyclopropyl carboxamide series could reduce asexual blood stage infection, we evaluated representative compounds WJM280 (**108**) and analog **109** in the 4-day *P. berghei* mouse model. In this model, mice were infected *P. berghei* asexual stage parasites, and then 2 h after the infection, WJM280 (**108**) and analog **109** at 50 mg/kg and artesunate at 30 mg/kg were administered by oral gavage. The same compound doses were administered again, 24, 48, and 72 h post infection. Parasitemia was then quantified by preparing blood smears and visualizing by microscopy across days 2, 3, and 4. It was shown that on day 4, WJM280 (**108**) and analog **109** reduced parasitemia on average by 19 % and 9 %, however, this data was not statistically significant relative to the vehicle control (Table 14 and Fig. S14). The known drug control, artesunate, on day 4 reduced parasitemia by 97.7 %. The body weights of mice on average remained unchanged with WJM280 (**108**) and slightly reduced with analog **109** suggesting the compounds using this dosing regimen were tolerated. The inability of these compounds to reduce parasitemia was attributed to their high metabolic turnover, which is a characteristic that needs improving on further development of the cyclopropyl carboxamide class.

**Table 14:** Evaluation of compounds in a Peter's 4 day *P. berghei* mouse model.^a^

compound	WJM280 (108)	109	ART
dose (mg/kg)	50	50	30
% parasitemia^b^	81	91	2.3
% reduction in parasitemia^c^	19	9	97.7

a *P. berghei* ANKA parasites were injected into the tail vein to infect mice on day 0. Compounds WJM280 (**108**) and **109** were administered 50 mg/kg q.d. by oral gavage 2 h after infection (day 0) and then on days 1, 2, and 3. Parasitemia of blood samples was measured by microscopy.

b Average % parasitemia for 4 mice on day 4.

c Average % reduction in parasitemia versus the vehicle control for 4 mice on day 4. Fig. S14 shows average data and error plotted. Data for WJM280 (**108**) and **109** compared to the vehicle was not statistically significant. ART = artesunate.

## 3 Conclusions

The cyclopropyl carboxamide chemotype was identified from a screen of the Jannsen Jumpstarter Library. We performed a systematic SAR investigation of this scaffold, which revealed that the cyclopropyl carboxamide was key to maintaining potency, while the activity contributed by the remainder of the scaffold was largely driven by lipophilicity. The cyclopropyl carboxamide moiety is found in several other structurally similar antimalarial compounds activity that putatively target cyt *b* (Fig. 1), and it is reasoned this motif is important for the activity of these compounds. The lipophilic nature of the cyclopropyl carboxamide class is common to cyt *b* inhibitors such as ATQ and ELQ300, most likely due to the non-polar amino acids that align the pocket that binds the natural substrates ubiquinone or ubiquinol. As a result of the SAR exploration, WJM280 (**108**) was generated as a frontrunner compound with potent asexual stage activity. Cyclopropyl carboxamide analogs were generally found to have relatively high metabolic turnover in rat hepatocytes and human liver microsomes, although WJM280 (**108**) has modestly enhanced stability in human liver microsomes. Aqueous solubility was reasonable at physiological pH, although did vary considerably, and this could be attributed to the lipophilic nature of the analogs. Overall, these findings suggest that improving these ADME properties of the cyclopropyl carboxamide series in the future will be challenging.

Cyt *b* was identified as the molecular target of the cyclopropyl carboxamide series using a pulsed incremental treatment of 3D7 parasites with W466 (**1**) and W499 (**2**). A SNP in the Q_o_ site of cyt *b* was uncovered for W466 (**1**), while a CNV in the genomic region that encoded DHODH was found for W499-resistant populations. No recrudescent parasites were detected for W499 (**2**) in the MIR assay that uses a compound concentration of 3 × the EC_90_, while recrudescent populations were detected for W466 (**1**). Genome sequencing of Dd2 and Dd2-Polδ W466**-**recrudescent parasites from the MIR study also uncovered SNPs in the Q_o_ site of cyt *b* providing further evidence that cyt *b* was the molecular target of the carboxamide series. Overall, the MIR study indicated the cyclopropyl carboxamide class has a relatively low risk of acquiring resistance in clinical development, which is unusual for cyt *b* inhibitors that typically have a fast onset or high risk of resistance.

The Q_o_ site of cyt *b* was confirmed as the binding site and molecular target of the cyclopropyl carboxamide class evaluating the cyclopropyl carboxamide class against ATQ and ELQ300 resistant populations. This data showed that W466 (**1**), W499 (**2**), and WJM280 (**108**) showed decreased sensitivity to ATQ resistant populations with Q_o_ site mutations but not to an ELQ300 resistant population with a Q_i_ site mutation. Curiously, ATQ showed the same level of sensitivity to W466-resistant populations with a Q_o_ site A122T and F264L mutation to wildtype 3D7 parasites, suggesting structural divergence and a different binding mode between ATQ and the cyclopropyl carboxamide class. Expectedly, ATQ, ELQ300, and DSM265 showed decreased sensitivity to W499-resistant populations with an amplification of DHODH. These findings were further supported by the specific inhibition of complex III in the mitochondrial ETC by W466 (**1**) and W499 (**2**) using an oxygen consumption reduction assay.

Phenotypic characterization against the asexual parasite revealed cyclopropyl carboxamide analogs arrest parasites at the trophozoite stage and have a slow rate of kill consistent with the phenotype exhibited by ATQ. Moreover, cyclopropyl carboxamide analogs inhibit EEF and male gamete development comparable to the lifecycle phenotype observed by other cyt *b* inhibitors [8,23,44,45], suggesting a potential role in a chemoprevention therapy, although it is not yet clinically known whether cyt *b* inhibitors will be useful as a partner agent in a transmission blocking therapy. The cyclopropyl carboxamide analogs WJM280 (**108**) and **109** had no impact on parasitemia in a 4-day *P. berghei* mouse efficacy model, which was attributed to low metabolic stability. Enhancing metabolic stability while maintaining or improving antiparasitic potency remains a challenge in the future development of the cyclopropyl carboxamide chemotype.

## 4 Experimental section

### 4.1 General chemistry methods

NMR spectra were recorded on a Bruker Ascend™ 300. Chemical shifts are reported in ppm on the *δ* scale and referenced to the appropriate solvent peak. MeOD, DMSO‑*d*_6_, D_2_O, and CDCl_3_ contain H_2_O. Chromatography was performed with silica gel 60 (particle size 0.040–0.063 μm) using an automated CombiFlash Rf Purification System. LCMS were recorded on an Agilent LCMS system comprised of an Agilent G6120B Mass Detector, 1260 Infinity G1312B Binary pump, 1260 Infinity G1367E HiPALS autosampler and 1260 Infinity G4212B Diode Array Detector. Conditions for LCMS were as follows, column: Luna® Omega 3 μm PS C18 100 Å, LC Column 50 × 2.1 mm at 20 °C, injection volume 2 μL, gradient: 5–100 % B over 3 min (solvent A: H_2_O 0.1 % formic acid; solvent B: ACN 0.1 % formic acid), flow rate: 1.5 mL/min, detection: 254 nm, acquisition time: 4.3 min. Unless otherwise noted, all compounds were found to be >95 % pure by this method. HRMS were acquired through The Bio21 Mass Spectrometry and Proteomics Facility using a Thermo Scientific™ nano-LC Q Exactive™ Plus Mass spectrometer. Compounds **2**, **29**, **30**, **33**, **35**, **38**–**40**, **42**–**44**, **48**–**53**, **56**–**62**, **68**, **69**, **73**, **74**, **76**, **77, 81**, **85**–**87**, **89**, **92**–**96** and **101** were purchased commercially. The integrity and purity of these compounds was confirmed by LCMS to be >95 %.

### 4.2 Chemistry procedures

***4-((Benzyl(cyclopropyl)amino)methyl)-N-cyclopropylbenzamide (17). General Procedure A:*** To a stirred solution of **111** (30 mg, 0.14 mmol) and *N*-benzylcyclopropanamine (0.019 mL, 0.17 mmol) in MeCN (3.0 mL) was added K_2_CO_3_ (59 mg, 0.43 mmol) and the mixture was refluxed under an N_2_ atmosphere for 16 h before being passed through a pad of Celite which was subsequently washed with DCM (10 mL). Volatiles were removed and the resulting crude mixture was purified by column chromatography eluting with 0–5% MeOH/DCM to afford compound **17** as a white solid (10 mg, 33 %). ^1^H NMR (MeOD) *δ* 7.79–7.69 (m, 2H), 7.41–7.34 (m, 2H), 7.34–7.18 (m, 5H), 3.73 (s, 2H), 3.70 (s, 2H), 2.84 (tt, *J* = 7.4, 3.9 Hz, 1H), 1.85 (tt, *J* = 6.8, 3.8 Hz, 1H), 0.80 (td, *J* = 7.2, 4.9 Hz, 2H), 0.72–0.57 (m, 2H), 0.40 (dt, *J* = 6.4, 3.1 Hz, 2H), 0.27 (q, *J* = 3.5 Hz, 2H). ^13^C NMR (DMSO‑*d*_6_) *δ* 167.5, 142.0, 138.4, 133.1, 129.2, 128.9, 128.1, 127.0, 126.9, 58.2, 57.7, 36.3, 23.1, 7.3, 5.8. LCMS *m/z* 321.0 [M+H]. HRMS *m/z*: [M+H] Calcd for C_21_H_24_N_2_O 321.1961; Found 321.1963.

***4-((Benzylamino)methyl)-N-cyclopropylbenzamide (18).*** General Procedure A was followed using phenylmethanamine (0.019 mL, 0.17 mmol) Column chromatography eluting with 0–5% MeOH/DCM afforded compound **18** as a white solid (25 mg, 62 %). ^1^H NMR (CDCl_3_) *δ* 7.73–7.61 (m, 2H), 7.33 (s, 1H), 7.36–7.19 (m, 3H), 7.24–7.14 (m, 1H), 6.41–6.35 (m, 1H), 3.75 (d, *J* = 14.4 Hz, 4H), 2.83 (tq, *J* = 7.1, 3.6 Hz, 1H), 0.87–0.69 (m, 2H), 0.61–0.50 (m, 2H). ^13^C NMR (CDCl_3_) *δ* 168.9, 144.2, 140.2, 133.2, 128.5, 128.2 (2C), 127.2, 127.1, 53.2, 52.8, 23.2, 6.8. LCMS *m/z* 281.2 [M+H]. HRMS *m/z*: [M+H] Calcd for C_18_H_20_N_2_O 281.1648; Found 281.1649.

***4-((Benzyl(methyl)amino)methyl)-N-cyclopropylbenzamide (19)*.** General Procedure A was followed using *N*-methyl-1-phenylmethanamine (0.022 mL, 0.17 mmol). Column chromatography eluting with 30–60 % EtOAc/heptane afforded compound **19** as a white solid (18 mg, 43 %). ^1^H NMR (CDCl_3_) *δ* 7.70–7.60 (m, 2H), 7.42–7.33 (m, 2H), 7.29 (d, *J* = 2.0 Hz, 3H), 7.26–7.16 (m, 2H), 6.26 (s, 1H), 3.48 (d, *J* = 7.7 Hz, 4H), 2.84 (tq, *J* = 7.1, 3.6 Hz, 1H), 2.12 (s, 3H), 0.81 (td, *J* = 7.1, 5.3 Hz, 2H), 0.62–0.50 (m, 2H). ^13^C NMR (CDCl_3_) *δ* 168.9, 143.3, 139.1, 133.3, 129.1, 129.0, 128.4, 127.2, 127.0, 62.0, 61.5, 42.4, 23.2, 6.9. LCMS *m/z* 295.2 [M+H]. HRMS *m/z*: [M+H] Calcd for C_19_H_22_N_2_O 295.1805; Found 295.1804.

***4-((Benzyl(ethyl)amino)methyl)-N-cyclopropylbenzamide (20).*** Compound **114** (49 mg, 0.26 mmol) and *N*-benzylethanamine (0.077 mL, 0.52 mmol) were dissolved in in MeOH (1 mL) and stirred at 50 °C for 2 h. The reaction was then cooled to 0 °C and NaBH_4_ (29 mg, 0.78 mmol) was added. The reaction was allowed to warm to rt and stirred for an additional 1 h. The reaction was then quenched with 10 % citric acid (1 mL) and concentrated. The reaction was then dissolved in DCM (10 mL) and washed successively with sat. aq. NaHCO_3_ (10 mL) and brine (10 mL). The organic fraction was dried over anhydrous Na_2_SO_4_, filtered and concentrated. Column chromatography eluting with 70–100 % EtOAc/DCM afforded compound **20** as a colourless oil (27 mg, 34 %). ^1^H NMR (CDCl_3_): *δ* 7.64–7.72 (m, *J* = 8.3 Hz, 2H), 7.40–7.47 (m, *J* = 8.1 Hz, 2H), 7.28–7.39 (m, 4H), 7.19–7.26 (m, 1H), 6.27 (br. s., 1H), 3.59 (d, *J* = 8.2 Hz, 4H), 2.90 (dt, *J* = 3.4, 7.1 Hz, 1H), 2.50 (q, *J* = 7.1 Hz, 2H), 1.07 (t, *J* = 7.1 Hz, 3H), 0.82–0.94 (m, 2H), 0.57–0.67 (m, 2H). LCMS *m/z* 309.2 [M+H]. HRMS *m/z*: [M+H] Calcd for C_20_H_24_N_2_O 309.1961; Found 309.1962.

***4-((Benzyl(isopropyl)amino)methyl)-N-cyclopropylbenzamide (21).*** Compound **117** (175 mg, 0.754 mmol) and benzaldehyde (80 mg, 0.75 mmol) were dissolved in DCE (3 mL). To this solution was added AcOH (0.040 mL, 0.75 mmol) and the mixture was stirred for 10 min at room temperature. Then, NaBH(OAc)_3_ (320 mg, 1.51 mmol) was added in portions and stirring was continued for another 2 h. On completion, the reaction mixture was diluted with DCM and quenched with sat. aq. NaHCO_3_ solution. The organic layer was separated and the aqueous layer was extracted with DCM (2 × 10 mL). Combined organic fractions were washed with brine, dried over anhydrous Na_2_SO_4_ and concentrated under reduced pressure. Column chromatography eluting with 30–40 % EtOAc/hexane afforded compound **21** as a white solid (100 mg, 41 %). ^1^H NMR (DMSO‑*d*_6_) *δ* 8.34 (d, *J* = 4.2 Hz, 1H), 7.73 (d, *J* = 7.9 Hz, 2H), 7.41 (d, *J* = 7.9 Hz, 2H), 7.38–7.25 (m, 4H), 7.20 (t, *J* = 7.1 Hz, 1H), 3.55 (s, 2H), 3.50 (s, 2H), 2.86–2.73 (m, 2H), 1.03 (d, *J* = 6.6 Hz, 6H), 0.66 (dt, *J* = 6.8, 3.3 Hz, 2H), 0.54 (q, *J* = 3.7 Hz, 2H). ^13^C NMR (DMSO‑*d*_6_) *δ* 167.4, 144.1, 140.4, 133.0, 128.2, 128.1, 127.9, 127.1, 126.6, 52.8, 52.3, 48.2, 23.0, 17.4, 5.8. LCMS *m/z* 323.3 [M+H]. HRMS *m/z*: [M+H] Calcd for C_21_H_26_N_2_O 323.2118; Found 323.2106.

***4-((Benzyl(cyclobutyl)amino)methyl)-N-cyclopropylbenzamide (22)*.** General Procedure A was followed using *N-*benzylcyclobutanamine (0.051 mL, 0.29 mmol). Column chromatography eluting with 10–30 % EtOAc/heptane afforded compound **22** as a white solid (17 mg, 21 %). ^1^H NMR (MeOD) *δ* 7.76–7.68 (m, 2H), 7.38 (d, *J* = 8.1 Hz, 2H), 7.31–7.14 (m, 5H), 3.49 (d, *J* = 10.3 Hz, 4H), 3.23–3.07 (m, 1H), 2.83 (tt, *J* = 7.4, 3.9 Hz, 1H), 2.01–1.79 (m, 4H), 1.70–1.48 (m, 2H), 0.80 (td, *J* = 7.1, 4.8 Hz, 2H), 0.74–0.57 (m, 2H). ^13^C NMR (MeOD) *δ* 170.9, 143.9, 139.1, 133.2, 129.5, 129.3, 128.3, 127.2, 127.2, 59.0, 54.9, 54.4, 47.3, 28.3, 23.1, 14.4, 5.7. LCMS *m/z* 335.4 [M+H]. HRMS *m/z*: [M+H] Calcd for C_22_H_26_N_2_O 335.2118; Found 335.2120.

***4-((Benzyl(tert-butyl)amino)methyl)-N-cyclopropylbenzamide (23).*** General Procedure A was followed using *N*-benzyl-2-methylpropan-2-amine (0.052 mL, 0.29 mmol). Column chromatography eluting with 10–30 % EtOAc/heptane afforded compound **23** as a white solid (47 mg, 59 %). ^1^H NMR (CDCl_3_) *δ* 7.57–7.47 (m, 2H), 7.33–7.24 (m, 4H), 7.27–7.04 (m, 3H), 6.12 (s, 1H), 3.71 (d, *J* = 7.6 Hz, 4H), 2.86 (tq, *J* = 7.1, 3.7 Hz, 1H), 1.13 (s, 9H), 0.95–0.77 (m, 2H), 0.57 (ddd, *J* = 6.9, 5.2, 3.9 Hz, 2H). ^13^C NMR (CDCl_3_) *δ* 169.0, 147.2, 142.3, 132.3, 128.5, 128.2, 128.0, 126.5, 126.4, 55.8, 54.8, 54.1, 27.6, 23.1, 6.9. LCMS *m/z* 337.4 [M+H]. HRMS *m/z*: [M+H] Calcd for C_22_H_28_N_2_O 337.2274; Found 337.2277.

***4-((Benzyl(sec-butyl)amino)methyl)-N-cyclopropylbenzamide (24). General Procedure B:*** To a solution of **114** (90 mg, 0.48 mmol) and **121** (118 mg, 0.719 mmol) in DCE (5 mL) was added AcOH (0.051 mL, 0.96 mmol) and the mixture was stirred for 1–4 h at rt under N_2_ atmosphere. NaBH(OAc)_3_ (203 mg, 0.958 mmol) was then added in portions and stirring was continued for another 6 h. On completion, the reaction mixture was diluted with DCM (10 mL) and quenched with sat. aq. NaHCO_3_. The organic fraction was separated and the aqueous fraction was extracted with DCM (2 × 10 mL). Combined organic fractions were washed with brine, dried over anhydrous Na_2_SO_4_ and concentrated under reduced pressure. Column chromatography eluting with 30–50 % EtOAc/hexane afforded compound **24** as a white solid (75 mg, 47 %). ^1^H NMR (DMSO‑*d*_6_) *δ* 8.34 (d, *J* = 4.3 Hz, 1H), 7.74 (d, *J* = 8.1 Hz, 2H), 7.41 (d, *J* = 8.1 Hz, 2H), 7.38–7.26 (m, 4H), 7.25–7.16 (m, 1H), 3.62 (t, *J* = 14.8 Hz, 2H), 3.43 (dd, *J* = 19.6, 14.1 Hz, 2H), 2.82 (tq, *J* = 7.8, 4.0 Hz, 1H), 2.51–2.39 (m, 1H), 1.60 (dp, *J* = 14.7, 7.3 Hz, 1H), 1.27 (dp, *J* = 14.2, 7.2 Hz, 1H), 0.98 (d, *J* = 6.5 Hz, 3H), 0.83 (t, *J* = 7.4 Hz, 3H), 0.67 (dt, *J* = 6.9, 3.3 Hz, 2H), 0.54 (dt, *J* = 6.9, 4.3 Hz, 2H). ^13^C NMR (DMSO‑*d*_6_) *δ* 167.4, 143.9, 140.3, 133.0, 128.3, 128.1, 128.0, 127.1, 126.7, 54.3, 52.8, 52.4, 26.0, 23.0, 13.1, 11.6, 5.8, 5.7. LCMS *m/z* 337.4 [M+H]. HRMS *m/z*: [M+H] Calcd for C_22_H_28_N_2_O 337.2274; Found 337.2264.

***4-((Benzyl(2,2,2-trifluoroethyl)amino)methyl)-N-cyclopropylbenzamide (25).*** General Procedure B was followed using **114** (80 mg, 0.42 mmol) and **122** (120 mg, 0.634 mmol). Column chromatography eluting with 30–50 % EtOAc/hexane compound **25** as a white solid (14 mg, 10 %). ^1^H NMR (DMSO‑*d*_6_) *δ* 8.39 (d, *J* = 4.2 Hz, 1H), 7.78 (d, *J* = 8.1 Hz, 2H), 7.42–7.30 (m, 6H), 7.30–7.23 (m, 1H), 3.79 (s, 2H), 3.74 (s, 2H), 3.26 (t, *J* = 10.0 Hz, 2H), 2.82 (tt, *J* = 7.8, 3.9 Hz, 1H), 0.68 (td, *J* = 7.1, 4.6 Hz, 2H), 0.55 (p, *J* = 4.3 Hz, 2H). ^13^C NMR (DMSO‑*d*_6_) *δ* 167.3, 141.6, 138.1, 133.5, 128.6, 128.4, 128.3, 127.3, 127.3, 126.2 (q, *J* = 282 Hz), 57.5 (q, *J* = 26 Hz), 52.8 (q, *J* = 30 Hz), 23.0, 5.7. ^19^F NMR (DMSO‑*d*_6,_ 376 MHz) *δ* −67.87 (s, 3F). LCMS *m/z* 363.3 [M+H]. HRMS *m/z*: [M+H] Calcd for C_20_H_21_F_3_N_2_O 363.1679; Found 363.1668.

***4-((Benzyl(propyl)amino)methyl)-N-cyclopropylbenzamide (26).*** General Procedure A was followed using *N*-benzylpropan-1-amine (0.047 mL, 0.29 mmol). Column chromatography eluting with 10–30 % EtOAc/heptane afforded compound **26** as a white solid (40 mg, 52 %). ^1^H NMR (CDCl_3_) *δ* 7.72–7.62 (m, 2H), 7.46–7.17 (m, 7H), 6.22 (s, 1H), 3.56 (d, *J* = 6.3 Hz, 4H), 2.96–2.82 (m, 1H), 2.37 (t, *J* = 7.3 Hz, 2H), 1.52 (h, *J* = 7.4 Hz, 2H), 0.92–0.77 (m, 5H), 0.66–0.55 (m, 2H). ^13^C NMR (CDCl_3_) *δ* 169.0, 144.2, 139.8, 133.0, 128.8, 128.8, 128.3, 126.9, 126.9, 58.4, 58.1, 55.6, 23.2, 20.3, 11.9, 6.8. LCMS *m/z* 323.4 [M+H]. HRMS *m/z*: [M+H] Calcd for C_21_H_26_N_2_O 323.2118; Found 323.2119.

***N-Cyclopropyl-4-((dibenzylamino)methyl)benzamide (27).*** General Procedure A was followed using *N*-benzyl-2-methylpropan-2-amine (0.10 mL, 0.52 mmol). Column chromatography eluting with 0–10 % EtOAc/DCM afforded compound **27** as a colourless semi-solid (73 mg, 46 %). ^1^H NMR (MeOD) *δ* 7.79–7.70 (m, 2H), 7.46 (d, *J* = 8.1 Hz, 2H), 7.41–7.15 (m, 11H), 3.53 (s, 2H), 3.49 (s, 4H), 2.83 (tt, *J* = 7.4, 3.9 Hz, 1H), 0.78 (dt, *J* = 7.1, 3.5 Hz, 2H), 0.68–0.56 (m, 2H). ^13^C NMR (MeOD) *δ* 170.9, 144.3, 139.8, 133.4, 129.0, 129.0, 128.5, 127.4, 127.2, 58.1, 57.5, 23.1, 5.7. LCMS *m/z* 371.2 [M+H]. HRMS *m/z*: [M+H] Calcd for C_25_H_26_N_2_O 371.2118; Found 371.2109.

***3-((Benzyl(cyclopropyl)amino)methyl)-N-cyclopropylbenzamide (28).*** General Procedure B was followed using **123** and *N*-benzylcyclopropanamine (0.50 mg, 0.34 mmol). Column chromatography eluting with 15–30 % EtOAc/hexane afforded compound **28** as a white solid (42 mg, 39 %). ^1^H NMR (DMSO‑*d*_6_) *δ* 8.40 (d, *J* = 4.2 Hz, 1H), 7.73–7.64 (m, 2H), 7.44–7.20 (m, 6H), 3.63 (d, *J* = 14.2 Hz, 4H), 2.83 (tq, *J* = 7.8, 4.0 Hz, 1H), 1.78 (tt, *J* = 6.7, 3.7 Hz, 1H), 0.68 (td, *J* = 7.1, 4.6 Hz, 2H), 0.64–0.52 (m, 2H), 0.35 (td, *J* = 6.5, 4.3 Hz, 2H), 0.23–0.14 (m, 2H). ^13^C NMR (DMSO‑*d*_6_) *δ* 167.6, 138.8, 138.3, 134.2, 131.8, 129.1, 128.0, 128.0, 127.8, 126.8, 125.6, 58.1, 57.9, 36.2, 23.1, 7.3, 5.8. LCMS *m/z* 321.3 [M+H]. HRMS *m/z*: [M+H] Calcd for C_21_H_24_N_2_O 321.1961; Found 321.1953.

***4-((Cyclopropyl(2,4-dichlorobenzyl)amino)methyl)-N-ethylbenzamide (31). General Procedure C:*** To a stirred solution of **120** (100 mg, 0.286 mmol) in DMF (3 mL) were added DIPEA (0.25 mL, 1.4 mmol), EDCI (82 mg, 0.43 mmol) and HOBt (46.3 mg, 0.343 mmol) at 0 °C under Ar. The mixture was stirred for 10 min, and then ethylamine hydrochloride (35 mg, 0.43 mmol) was added. The ice-bath was removed and the reaction mixture was stirred at rt for approximately 16 h. On completion, the reaction mixture was diluted with EtOAc and washed with water (3 × 10 mL) and brine (5 mL). The organic fraction was dried over Na_2_SO_4_, filtered, and concentrated under reduced pressure. Column chromatography eluting with 15–30 % EtOAc/hexane followed by trituration with MeOH/DCM/pentane (1:3:6) afforded compound **31** as a white solid (40 mg, 37 %). ^1^H NMR (DMSO‑*d*_6_) *δ* 8.41 (t, *J* = 5.4 Hz, 1H), 7.76 (d, *J* = 7.8 Hz, 2H), 7.54 (d, *J* = 2.2 Hz, 1H), 7.45 (d, *J* = 8.3 Hz, 1H), 7.41–7.28 (m, 3H), 3.73 (d, *J* = 4.1 Hz, 4H), 3.29–3.22 (m, 2H), 1.89 (tt, *J* = 6.7, 3.7 Hz, 1H), 1.11 (t, *J* = 7.2 Hz, 3H), 0.33 (dt, *J* = 6.5, 3.2 Hz, 2H), 0.14 (p, *J* = 4.2 Hz, 2H). ^13^C NMR (DMSO‑*d*_6_) *δ* 165.8, 141.4, 135.6, 134.2, 133.3, 132.9, 132.2, 128.9, 128.5, 127.0, 126.8, 58.8, 55.2, 36.7, 34.0, 14.8, 7.1. LCMS *m/z* 377.3 [M+H]. HRMS *m/z*: [M+H] Calcd for C_20_H_22_C_l2_N_2_O 377.1182; Found 377.1172.

***4-((Cyclopropyl(2,4-dichlorobenzyl)amino)methyl)-N-propylbenzamide (32).*** General Procedure C was followed using *n*-propylamine (0.035 mL, 0.43). Column chromatography eluting with 15–25 % EtOAc/hexane followed by trituration with MeOH-DCM-pentane (1:3:6) to afford compound **32** as white solid (30 mg, 27 %). ^1^H NMR (DMSO‑*d*_6_) *δ* 8.40 (s, 1H), 7.77 (d, *J* = 7.8 Hz, 2H), 7.54 (d, *J* = 2.3 Hz, 1H), 7.45 (d, *J* = 8.3 Hz, 1H), 7.41–7.30 (m, 3H), 3.73 (d, *J* = 4.3 Hz, 4H), 3.20 (q, *J* = 6.5 Hz, 2H), 1.89 (d, *J* = 7.3 Hz, 1H), 1.52 (h, *J* = 7.4 Hz, 2H), 0.88 (t, *J* = 7.4 Hz, 3H), 0.34 (d, *J* = 6.4 Hz, 2H), 0.14 (s, 2H). ^13^C NMR (DMSO‑*d*_6_) *δ* 166.0, 141.4, 135.6, 134.2, 133.3, 132.9, 132.2, 128.9, 128.5, 127.0, 126.9, 58.8, 55.2, 41.0, 36.7, 22.4, 11.5, 7.1. LCMS *m/z* 391.3 [M+H]. HRMS *m/z*: [M+H] Calcd for C_21_H_24_C_l2_N_2_O 391.1338; Found 391.1329.

***4-((Cyclopropyl(2,4-dichlorobenzyl)amino)methyl)-N-(2,2,2-trifluoroethyl)benzamide (34).*** General Procedure C was followed using 2,2,2-trifluoroethan-1-amine (0.092 mL, 0.771 mmol). Column chromatography eluting with 15–25 % EtOAc/hexane followed by trituration with MeOH-DCM-pentane (1:3:6) afforded compound **34** as a white solid (117 mg, 53 %). ^1^H NMR (DMSO‑*d*_6_) *δ* 9.04 (t, *J* = 6.3 Hz, 1H), 7.82 (d, *J* = 8.0 Hz, 2H), 7.54 (d, *J* = 2.1 Hz, 1H), 7.46 (d, *J* = 8.3 Hz, 1H), 7.42–7.33 (m, 3H), 4.06 (td, *J* = 9.8, 6.3 Hz, 2H), 3.74 (d, *J* = 8.7 Hz, 4H), 3.31 (s, 2H), 1.90 (tt, *J* = 6.5, 3.6 Hz, 1H), 0.39–0.30 (m, 2H), 0.18–0.10 (m, 2H). ^13^C NMR (DMSO‑*d*_6_) *δ* 166.7, 142.5, 135.5, 134.3, 133.0, 132.2, 131.8, 129.1, 128.5, 127.2, 127.0, 124.9 (d, *J* = 279.4 Hz), 58.8, 55.3, 40.0, 36.8, 7.1. ^19^F NMR (DMSO‑*d*_6,_ 376 MHz) *δ* −70.41 (s, 3F). LCMS *m/z* 431.3 [M+H]. HRMS *m/z*: [M+H] Calcd for C_20_H_19_C_l2_F_3_N_2_O 431.0899; Found 431.089.

***2-(4-((Cyclopropyl(2,4-dichlorobenzyl)amino)methyl)benzamido)ethan-1-aminium 2,2,2-trifluoroacetate (36).*** General Procedure C was followed using *tert*-butyl (2-aminoethyl)carbamate (138 mg, 0.861 mmol). Column chromatography eluting with 15–25 % EtOAc in hexane afforded an off-white solid (120 mg) which was dissolved in EtOH (1 mL). To the solution was then added 1 M HCl in diethyl ether (3 mL) under Ar at 0 °C. The ice-bath was removed and the mixture stirred at rt for 30 min. On completion, reaction mixture was concentrated under reduced pressure. The crude residue was purified by reverse phase preparative HPLC eluting with 20–100 % MeCN/water (including 0.1 % TFA) to afford compound **36** as a light yellow gum (40 mg, 49 %). ^1^H NMR (DMSO‑*d*_6_) *δ* 8.60 (s, 1H), 7.86–7.77 (m, 5H), 7.66–7.25 (m, 4H), 3.84 (s, 4H), 3.49 (q, *J* = 6.1 Hz, 2H), 2.99 (q, *J* = 6.0 Hz, 2H), 2.00 (s, 1H), 0.39 (s, 2H), 0.21 (s, 2H). ^13^C NMR (DMSO‑*d*_6_) *δ* 166.7, 158.5 (q, *J* = 34.9 Hz), 138.0, 135.2, 134.2, 133.7, 131.8, 130.3, 128.9, 127.3, 116.1 (q, *J* = 293.3 Hz), 58.9, 55.2, 38.7, 37.2, 36.7, 6.1. ^19^F NMR (DMSO‑*d*_6,_ 376 MHz) *δ* −73.78 (s, 3F). LCMS *m/z* 392.3 [M+H]. HRMS *m/z*: [M+H] Calcd for C_20_H_23_C_l2_N_3_O 392.1291; Found 392.1281.

***4-((Cyclopropyl(2,4-dichlorobenzyl)amino)methyl)-N-(2-hydroxyethyl)benzamide (37).*** General Procedure C was followed using 2-aminoethan-1-ol (0.070 mL, 1.14 mmol). Trituration with MeOH-DCM-pentane (1:3:6) afforded compound **37** as a white solid (160 mg, 71 %). ^1^H NMR (MeOD) *δ* 7.76 (d, *J* = 8.1 Hz, 2H), 7.45–7.33 (m, 4H), 7.24 (dd, *J* = 8.3, 2.1 Hz, 1H), 3.79 (s, 4H), 3.71 (t, *J* = 5.8 Hz, 2H), 3.50 (t, *J* = 5.8 Hz, 2H), 1.92 (tt, *J* = 6.7, 3.6 Hz, 1H), 0.38 (dt, *J* = 6.4, 3.1 Hz, 2H), 0.22 (p, *J* = 4.2 Hz, 2H). ^13^C NMR (DMSO‑*d*_6_) *δ* 166.2, 141.5, 135.6, 134.2, 133.2, 132.9, 132.2, 128.9, 128.5, 127.0, 126.9, 59.8, 58.8, 55.2, 42.1, 36.7, 7.1. LCMS *m/z* 393.3 [M+H]. HRMS *m/z*: [M+H] Calcd for C_20_H_22_C_l2_N_2_O_2_ 393.1131; Found 393.1122.

***4-((Benzyl(cyclopropyl)amino)methyl)-N-cyclopropyl-N-methylbenzamide (41).*** To a stirred solution of **17** (30 mg, 0.094 mmol) in anhydrous DMF (1 mL) cooled to 0 °C on an ice bath and under an N_2_ atmosphere was added a 60 % dispersion of sodium hydride in mineral oil (11 mg, 0.28 mmol). The mixture was allowed to stir for 15 min before iodomethane (0.018 mL, 0.28 mmol) was added and the ice bath removed. The mixture was then stirred at rt an additional 16 h before being diluted with DCM (5 mL), washed with water (5 mL), saturated aqueous NaHCO_3_ (5 mL) and brine (5 mL). The organic fraction was then dried over MgSO_4_, filtered and concentrated to afford compound **41** as a beige solid (25 mg, 80 %). ^1^H NMR (CDCl_3_) *δ* 7.41 (d, *J* = 8.1 Hz, 2H), 7.35–7.15 (m, 8H), 3.65 (s, 4H), 3.05 (s, 3H), 2.78 (tt, *J* = 7.0, 4.1 Hz, 1H), 1.79 (tt, *J* = 6.5, 3.7 Hz, 1H), 0.67–0.17 (m, 8H). ^13^C NMR (DMSO‑*d*_6_) *δ* 171.3, 140.0, 138.5, 136.1, 129.1, 128.6, 126.9, 126.9, 58.4, 57.8, 36.3, 34.7, 32.4, 9.0, 7.2. LCMS *m/z* 335.4 [M+H]. HRMS *m/z*: [M+H] Calcd for C_22_H_26_N_2_O 335.2118; Found 335.2120.

***4-(((2-Chlorobenzyl) (cyclopropyl)amino)methyl)-N-cyclopropylbenzamide (45).*** General Procedure B was followed using 2-chlorobenzaldehyde (100 mg, 0.711 mmol) and **88** (196 mg, 0.854 mmol). Column chromatography eluting with 10–20 % EtOAc/hexane afforded compound **45** as a white solid (120 mg, 47 %). ^1^H NMR (DMSO‑*d*_6_) *δ* 8.37 (d, *J* = 4.2 Hz, 1H), 7.79–7.69 (m, 2H), 7.44 (dd, *J* = 7.2, 2.2 Hz, 1H), 7.38 (dd, *J* = 7.1, 2.0 Hz, 1H), 7.36–7.22 (m, 4H), 3.74 (d, *J* = 7.8 Hz, 4H), 2.83 (tq, *J* = 7.8, 4.0 Hz, 1H), 1.88 (tt, *J* = 6.7, 3.6 Hz, 1H), 0.67 (dt, *J* = 6.9, 3.3 Hz, 2H), 0.61–0.48 (m, 2H), 0.40–0.23 (m, 2H), 0.22–0.07 (m, 2H). ^13^C NMR (DMSO‑*d*_6_) *δ* 167.3, 141.7, 136.3, 133.4, 133.0, 131.6, 129.1, 128.8, 128.6, 126.9, 126.8, 58.7, 55.7, 36.7, 23.0, 7.0, 5.7. LCMS *m/z* 355.3 [M+H]. HRMS *m/z*: [M+H] Calcd for C_21_H_23_ClN_2_O 355.1572; Found 355.1574.

***4-(((3-Chlorobenzyl) (cyclopropyl)amino)methyl)-N-cyclopropylbenzamide (46).*** General Procedure A was followed using **112** (42 mg, 0.23 mmol). Column chromatography eluting with 0–20 % EtOAc/DCM afforded compound **46** as a white solid (17 mg, 25 %). ^1^H NMR (CDCl_3_) *δ* 7.68 (d, *J* = 7.8 Hz, 2H), 7.31 (d, *J* = 7.7 Hz, 3H), 7.22 (s, 2H), 7.13 (s, 1H), 6.23 (s, 1H), 3.70–3.58 (m, 4H), 2.91 (tq, *J* = 7.1, 3.6 Hz, 1H), 1.83 (s, 1H), 0.87 (td, *J* = 7.0, 5.2 Hz, 2H), 0.62 (qd, *J* = 5.1, 3.7 Hz, 2H), 0.51–0.17 (m, 4H). ^13^C NMR (DMSO‑*d*_6_) *δ* 167.3, 141.6, 141.3, 133.1, 132.8, 129.9, 128.8, 128.6, 127.6, 127.0, 126.8, 57.9, 57.6, 36.4, 23.0, 7.2, 5.7. LCMS *m/z* 355.2 [M+H]. HRMS *m/z*: [M+H] Calcd for C_21_H_23_ClN_2_O 355.1572; Found 355.1574.

***4-(((4-Chlorobenzyl) (cyclopropyl)amino)methyl)-N-cyclopropylbenzamide (47).*** General Procedure A was followed using **113** (59 mg, 0.33 mmol). Column chromatography eluting with 0–20 % EtOAc/DCM afforded compound **47** as a white solid (17 mg, 25 %). ^1^H NMR (CDCl_3_) *δ* 7.68 (d, *J* = 7.8 Hz, 2H), 7.31 (d, *J* = 7.7 Hz, 3H), 7.22 (s, 2H), 7.13 (s, 1H), 6.23 (s, 1H), 3.70–3.58 (m, 4H), 2.91 (tq, *J* = 7.1, 3.6 Hz, 1H), 1.83 (s, 1H), 0.87 (td, *J* = 7.0, 5.2 Hz, 2H), 0.62 (qd, *J* = 5.1, 3.7 Hz, 2H), 0.51–0.17 (m, 4H). ^13^C NMR (DMSO‑*d*_6_) *δ* 167.3, 141.7, 137.5, 133.0, 131.4, 130.8, 128.7, 127.9, 126.9, 57.8, 57.4, 36.3, 23.0, 7.2, 5.7. LCMS *m/z* 355.2 [M+H]. HRMS *m/z*: [M+H] Calcd for C_21_H_23_ClN_2_O 355.1572; Found 355.1571.

***N-Cyclopropyl-4-((cyclopropyl(3-isopropylbenzyl)amino)methyl)benzamide (54).*** General Procedure B was followed using 3-isopropylbenzaldehyde (100 mg, 0.675 mmol) and **88** (196 mg, 0.854 mmol). Column chromatography eluting with 10–20 % EtOAc/hexane afforded compound **54** as a white solid (120 mg, 47 %). ^1^H NMR (DMSO‑*d*_6_) *δ* 8.36 (d, *J* = 4.0 Hz, 1H), 7.76 (d, *J* = 7.8 Hz, 2H), 7.31 (d, *J* = 7.9 Hz, 2H), 7.23 (t, *J* = 7.6 Hz, 1H), 7.12 (d, *J* = 6.6 Hz, 2H), 7.06 (d, *J* = 7.5 Hz, 1H), 3.62 (d, *J* = 12.8 Hz, 4H), 2.85 (td, *J* = 11.2, 6.0 Hz, 2H), 1.80 (s, 1H), 1.20 (d, *J* = 6.9 Hz, 6H), 0.75–0.64 (m, 2H), 0.57 (q, *J* = 3.7 Hz, 2H), 0.37 (d, *J* = 5.7 Hz, 2H), 0.22 (q, *J* = 3.5 Hz, 2H). ^13^C NMR (DMSO‑*d*_6_) *δ* 167.3, 148.0, 142.0, 138.0, 133.0, 128.7, 127.9, 127.1, 126.9, 126.6, 124.8, 58.2, 57.6, 36.3, 33.3, 23.9, 23.0, 7.2, 5.7. LCMS *m/z* 363.4 [M+H]. HRMS *m/z*: [M+H] Calcd for C_24_H_30_N_2_O 363.2431; Found 363.243.

***N-Cyclopropyl-4-((cyclopropyl(4-isopropylbenzyl)amino)methyl)benzamide (55).*** General Procedure B was followed using 4-isopropylbenzaldehyde (68 mg, 0.456 mmol) and **88** (70 mg, 0.304 mmol). Column chromatography eluting with 10–20 % EtOAc/hexane afforded compound **55** as a white solid (43 mg, 39 %). ^1^H NMR (DMSO‑*d*_6_) *δ* 8.37 (d, *J* = 4.2 Hz, 1H), 7.75 (d, *J* = 8.0 Hz, 2H), 7.31 (d, *J* = 7.8 Hz, 2H), 7.18 (s, 4H), 3.63 (s, 2H), 3.58 (s, 2H), 2.92–2.78 (m, 2H), 1.82–1.75 (m, 1H), 1.19 (d, *J* = 6.9 Hz, 6H), 0.68 (td, *J* = 7.1, 4.6 Hz, 2H), 0.56 (q, *J* = 3.7 Hz, 2H), 0.37 (dt, *J* = 6.3, 3.0 Hz, 2H), 0.25–0.19 (m, 2H). ^13^C NMR (DMSO‑*d*_6_) *δ* 167.4, 146.8, 142.0, 135.5, 133.0, 129.0, 128.7, 126.9, 125.9, 57.7, 57.4, 36.2, 33.1, 23.9, 23.0, 7.3, 5.7. LCMS *m/z* 363.3 [M+H]. HRMS *m/z*: [M+H] Calcd for C_24_H_30_N_2_O 363.2431; Found 363.2429.

***N-Cyclopropyl-4-((cyclopropyl(4-(dimethylamino)benzyl)amino)methyl)benzamide (63).*** General Procedure B was followed using and 4-(dimethylamino)benzaldehyde (100 mg, 0.667 mmol) and **88** (185 mg, 0.806 mmol). Column chromatography eluting with 15–30 % EtOAc/hexane afforded compound **63** as a white solid (105 mg, 43 %). ^1^H NMR (DMSO‑*d*_6_) *δ* 8.36 (d, *J* = 4.2 Hz, 1H), 7.75 (d, *J* = 7.8 Hz, 2H), 7.30 (d, *J* = 7.9 Hz, 2H), 7.06 (d, *J* = 8.2 Hz, 2H), 6.71–6.64 (m, 2H), 3.60 (s, 2H), 3.50 (s, 2H), 2.92–2.79 (m, 6H), 1.75 (dd, *J* = 7.1, 3.7 Hz, 1H), 0.68 (dt, *J* = 6.9, 3.2 Hz, 2H), 0.57 (q, *J* = 3.7 Hz, 2H), 0.40–0.32 (m, 2H), 0.23–0.17 (m, 2H). ^13^C NMR (DMSO‑*d*_6_) *δ* 167.4, 149.5, 142.3, 132.9, 129.9, 128.7, 126.9, 125.3, 112.0, 57.3, 57.2, 40.2, 36.0, 23.0, 7.2, 5.8. LCMS *m/z* 364.3 [M+H]. HRMS *m/z*: [M+H] Calcd for C_23_H_29_N_3_O 364.2383; Found 364.2380.

***N-Cyclopropyl-4-((cyclopropyl(3-(dimethylamino)benzyl)amino)methyl)benzamide (64).*** General Procedure B was followed using **88** (70 mg, 0.30 mmol) and 3-(dimethylamino)benzaldehyde (68 mg, 0.46 mmol). Column chromatography eluting with 15–30 % EtOAc/hexane followed by trituration with diethyl ether and *n*-pentane (7:3) afforded compound **64** as a white solid (23 mg, 21 %). ^1^H NMR (DMSO‑*d*_6_) *δ* 8.37 (s, 1H), 7.76 (d, *J* = 7.8 Hz, 2H), 7.32 (d, *J* = 8.0 Hz, 2H), 7.12 (t, *J* = 7.8 Hz, 1H), 6.64–6.55 (m, 3H), 3.64 (s, 2H), 3.56 (s, 2H), 2.88 (s, 6H), 2.82 (s, 1H), 1.83–1.75 (m, 1H), 0.68 (q, *J* = 4.5 Hz, 2H), 0.57 (t, *J* = 4.2 Hz, 2H), 0.37 (d, *J* = 6.0 Hz, 2H), 0.24 (q, *J* = 3.7 Hz, 2H). ^13^C NMR (DMSO‑*d*_6_) *δ* 167.4, 150.3, 142.2, 138.6, 132.9, 128.7, 128.5, 126.9, 117.2, 113.2, 111.1, 58.5, 57.4, 40.2, 36.3, 23.0, 7.3, 5.7. LCMS *m/z* 364.4 [M+H]. HRMS *m/z*: [M+H] Calcd for C_23_H_29_N_3_O 364.2383; Found 364.2382.

***4-(((2-Acetamidobenzyl) (cyclopropyl)amino)methyl)-N-cyclopropylbenzamide (65). General Procedure D:*** To a stirred solution of **125** (500 mg, 1.49 mmol) in DCM (10 mL) at 0 °C was added DIPEA (0.78 mL, 4.5 mmol) followed by acetyl chloride (0.21 mL, 3.0 mmol) dropwise and the mixture was stirred at the same temperature for 30 min. On completion, water was added to the reaction mixture which was then extracted with EtOAc (3 × 10 mL). Combined organic fractions was washed with brine, dried over anhydrous Na_2_SO_4_ and concentrated under reduced pressure. Column chromatography eluting with 20–35 % EtOAc/hexane followed by reverse phase preparative HPLC eluting with 20–100 % MeCN/water (including 20 mM ammonium bicarbonate) afforded compound **65** as a white solid (90 mg, 16 %). ^1^H NMR (MeOD) *δ* 7.97–7.87 (m, 1H), 7.79–7.69 (m, 2H), 7.37–7.21 (m, 4H), 7.13–7.03 (m, 1H), 3.82 (s, 2H), 3.70 (s, 2H), 2.84 (tt, *J* = 7.4, 3.9 Hz, 1H), 2.01 (s, 3H), 1.87 (tt, *J* = 6.8, 3.8 Hz, 1H), 0.80 (dt, *J* = 7.1, 3.4 Hz, 2H), 0.69–0.58 (m, 2H), 0.46 (dt, *J* = 6.6, 3.2 Hz, 2H), 0.35–0.22 (m, 2H). ^13^C NMR (DMSO‑*d*_6_) *δ* 167.6, 167.3, 140.6, 137.9, 133.2, 130.1, 129.4, 127.9, 127.6, 127.0, 123.7, 121.7, 57.9, 57.2, 36.5, 24.0, 23.0, 6.7, 5.8. LCMS *m/z* 378.4 [M+H]. HRMS *m/z*: [M+H] Calcd for C_23_H_27_N_3_O_2_ 378.2176; Found 378.2173.

***4-(((3-Acetamidobenzyl) (cyclopropyl)amino)methyl)-N-cyclopropylbenzamide (66).*** General Procedure D was followed using **127** (100 mg, 0.298 mmol). Column chromatography eluting with 20–35 % EtOAc/hexane followed by treatment with 2 M HCl in diethyl ether afforded the mono hydrochloride salt of compound **66** as a white solid (28 mg, 23 %). ^1^H NMR (DMSO‑*d*_6_) *δ* 10.78 (s, 1H), 10.15 (s, 1H), 8.54 (d, *J* = 4.2 Hz, 1H), 7.87 (d, *J* = 7.5 Hz, 3H), 7.69 (d, *J* = 7.9 Hz, 2H), 7.58 (d, *J* = 8.0 Hz, 1H), 7.37 (t, *J* = 7.8 Hz, 1H), 7.30 (d, *J* = 7.6 Hz, 1H), 4.47 (d, *J* = 13.0 Hz, 1H), 4.33 (s, 4H), 2.85 (dq, *J* = 7.3, 3.7 Hz, 1H), 2.64–2.59 (m, 1H), 2.06 (s, 3H), 0.74–0.51 (m, 8H). ^13^C NMR (DMSO‑*d*_6_) *δ* 168.5, 166.9, 139.4, 134.9, 131.7, 128.8, 127.2, 126.5, 122.4, 120.0, 58.6, 57.7, 36.1, 23.9, 23.1, 5.7, 4.8. LCMS *m/z* 378.3 [M+H]. HRMS *m/z*: [M+H] Calcd for C_23_H_27_N_3_O_2_ 378.2176; Found 378.2173.

***4-(((4-Acetamidobenzyl) (cyclopropyl)amino)methyl)-N-cyclopropylbenzamide (67).*** To a stirred solution of **128** (120 mg, 0.328 mmol) in EtOH (2 mL) and water (0.67 mL) was added ammonium chloride (17 mg, 2.7 mmol) followed by iron powder (18 mg, 2.7 mmol) and the mixture was refluxed for 2 h. On completion, the reaction mixture was cooled to rt, diluted with EtOH (10 mL) and filtered through a pad of Celite. The filtrate was concentrated under reduced pressure, water (10 mL) was added to the residue and it was then extracted with DCM (3 × 10 mL). Combined organic fractions were washed with brine, dried over anhydrous Na_2_SO_4_ and concentrated under reduced pressure to afford 4-(((4-aminobenzyl) (cyclopropyl)amino)methyl)-*N*-cyclopropylbenzamide as a brown gum which was unstable to storage and used in the next step immediately by dissolving in DCM (8 mL) and following General Procedure D. Purification by reverse phase preparative HPLC eluting with 20–100 % MeCN/water (including 20 mM ammonium bicarbonate) afforded compound **67** as a white solid (21 mg, 17 %). ^1^H NMR (DMSO‑*d*_6_) *δ* 9.90 (s, 1H), 8.38 (d, *J* = 4.2 Hz, 1H), 7.75 (d, *J* = 7.9 Hz, 2H), 7.51 (d, *J* = 8.1 Hz, 2H), 7.32 (d, *J* = 7.9 Hz, 2H), 7.17 (d, *J* = 8.1 Hz, 2H), 3.62 (s, 2H), 3.54 (s, 2H), 2.83 (td, *J* = 7.3, 3.7 Hz, 1H), 2.02 (s, 3H), 1.76 (dq, *J* = 6.8, 3.5 Hz, 1H), 0.68 (dt, *J* = 6.8, 3.3 Hz, 2H), 0.56 (p, *J* = 4.5 Hz, 2H), 0.35 (dt, *J* = 6.5, 3.2 Hz, 2H), 0.19 (p, *J* = 4.1 Hz, 2H). ^13^C NMR (DMSO‑*d*_6_) *δ* 168.2, 167.4, 142.0, 138.1, 133.0, 132.7, 129.4, 128.8, 126.9, 118.6, 57.6, 57.5, 36.2, 24.0, 23.0, 7.2, 5.7. LCMS *m/z* 378.4 [M+H]. HRMS *m/z*: [M+H] Calcd for C_23_H_27_N_3_O_2_ 378.2176; Found 378.2172.

***N-Cyclopropyl-4-((cyclopropyl(3-(hydroxymethyl)benzyl)amino)methyl)benzamide (70).*** Compound **129** was neutralized by dissolving the compound in DCM followed by addition of triethylamine at 0 °C. After neutralization, the mixture was concentrated under vacuo and the crude product (140 mg, 0.373 mmol) dissolved in THF (8 mL). 1 M LiAlH_4_ in THF solution (0.56 mL, 0.56 mmol) was added at 0 °C and the reaction mixture was stirred at the same temperature for 1 h. On completion, the reaction mixture was diluted with EtOAc and quenched slowly with 15 % aq. NaOH solution at 0 °C. The mixture was filtered through a pad of Celite which was then washed with EtOAc (3 × 10 mL). Combined filtrate was concentrated under reduced pressure and the crude residue was triturated with EtOAc and *n*-pentane (4:6) to afford compound **70** as a white solid (70 mg, 54 % yield). ^1^H NMR (DMSO‑*d*_6_) *δ* 8.37 (d, *J* = 4.2 Hz, 1H), 7.76 (d, *J* = 7.9 Hz, 2H), 7.35–7.16 (m, 5H), 7.13 (d, *J* = 7.4 Hz, 1H), 5.15 (t, *J* = 5.7 Hz, 1H), 4.49 (d, *J* = 5.9 Hz, 2H), 3.63 (s, 2H), 3.61 (s, 2H), 2.83 (tq, *J* = 7.8, 4.1 Hz, 1H), 1.79 (tt, *J* = 6.8, 3.6 Hz, 1H), 0.68 (dt, *J* = 6.9, 3.3 Hz, 2H), 0.56 (p, *J* = 4.5 Hz, 2H), 0.36 (dt, *J* = 6.3, 3.1 Hz, 2H), 0.21 (p, *J* = 4.1 Hz, 2H). ^13^C NMR (DMSO‑*d*_6_) *δ* 167.4, 142.3, 141.9, 138.0, 133.0, 128.8, 127.7, 127.5, 127.2, 126.9, 125.0, 62.9, 58.1, 57.5, 36.3, 23.0, 7.3, 5.7. LCMS *m/z* 351.3 [M+H]. HRMS *m/z*: [M+H] Calcd for C_22_H_26_N_2_O_2_ 351.2067; Found 351.2063.

***N-Cyclopropyl-4-((cyclopropyl(4-(hydroxymethyl)benzyl)amino)methyl)benzamide (71).*** To a stirred solution of **130** (130 mg, 0.344 mmol) in THF (5 mL) was added 1 M LiAlH_4_ in THF solution (0.52 mL, 0.52 mmol) at 0 °C and the reaction mixture was stirred at the same temperature for 1 h. On completion, the reaction mixture was diluted with EtOAc and quenched slowly with 15 % aq. NaOH solution at 0 °C. The mixture was filtered through a pad of Celite which was then extracted with EtOAc (3 × 5 mL). Organic fractions were combined and concentrated under reduced pressure and the crude residue was triturated with EtOAc and *n*-pentane (4:6) to afford compound **71** as an off-white solid (54 mg, 45 % yield). ^1^H NMR (DMSO‑*d*_6_) *δ* 8.36 (d, *J* = 4.2 Hz, 1H), 7.76 (d, *J* = 7.9 Hz, 2H), 7.35–7.18 (m, 6H), 5.12 (t, *J* = 5.6 Hz, 1H), 4.48 (d, *J* = 5.8 Hz, 2H), 3.63 (s, 2H), 3.59 (s, 2H), 2.82 (tt, *J* = 7.6, 3.9 Hz, 1H), 1.78 (tt, *J* = 6.7, 3.7 Hz, 1H), 0.68 (dt, *J* = 6.9, 3.3 Hz, 2H), 0.56 (p, *J* = 4.5 Hz, 2H), 0.36 (td, *J* = 6.4, 4.2 Hz, 2H), 0.21 (p, *J* = 4.1 Hz, 2H). ^13^C NMR (DMSO‑*d*_6_) *δ* 167.4, 142.0, 141.1, 136.5, 133.0, 128.9, 128.7, 126.9, 126.2, 62.8, 57.8, 57.5, 36.2, 23.0, 7.3, 5.7. LCMS *m/z* 351.3 [M+H]. HRMS *m/z*: [M+H] Calcd for C_22_H_26_N_2_O_2_ 351.2067; Found 351.2062.

***N-Cyclopropyl-4-((cyclopropyl(2,4-difluorobenzyl)amino)methyl)benzamide (72).*** General Procedure B was followed using **88** (70 mg, 0.30 mmol) and 2,4-difluorobenzaldehyde (65 mg, 0.46 mmol). Column chromatography eluting with 15–30 % EtOAc/hexane followed by trituration with diethyl ether and *n*-pentane (7:3) afforded compound **72** as a white solid (75 mg, 69 %). ^1^H NMR (DMSO‑*d*_6_) *δ* 8.38 (d, *J* = 4.2 Hz, 1H), 7.75 (d, *J* = 7.8 Hz, 2H), 7.42–7.30 (m, 3H), 7.16 (td, *J* = 9.9, 2.6 Hz, 1H), 7.03 (td, *J* = 8.5, 2.6 Hz, 1H), 3.69 (s, 2H), 3.63 (s, 2H), 2.83 (dq, *J* = 7.4, 3.8 Hz, 1H), 1.79 (dt, *J* = 6.7, 3.3 Hz, 1H), 0.67 (dt, *J* = 6.8, 3.3 Hz, 2H), 0.56 (p, *J* = 4.4 Hz, 2H), 0.35 (dt, *J* = 6.3, 3.1 Hz, 2H), 0.15 (p, *J* = 4.1 Hz, 2H). ^13^C NMR (DMSO‑*d*_6_) *δ* 167.4, 162.8 (dd, *J* = 44.0, 12.3 Hz), 159.5 (dd, *J* = 46.0, 12.3 Hz), 141.8, 133.3–132.8 (m), 133.0, 128.6, 126.9, 121.4 (dd, *J* = 15.3, 3.6 Hz), 111.0 (dd, *J* = 20.9, 3.7 Hz), 103.8–103.0 (m), 58.3, 50.6, 36.2, 23.0, 7.1, 5.7. ^19^F NMR (DMSO‑*d*_6,_ 376 MHz) *δ* −111.94 (d, *J* = 7.1 Hz, 1F), −113.41 (d, *J* = 7.7 Hz, 1F). LCMS *m/z* 357.3 [M+H]. HRMS *m/z*: [M+H] Calcd for C_21_H_22_F_2_N_2_O 357.1773; Found 357.1767.

***N-Cyclopropyl-4-((cyclopropyl(2,4,6-trifluorobenzyl)amino)methyl)benzamide (75).*** General Procedure B was followed using **88** (25 mg, 0.11 mmol) and 2,4,6-trifluorobenzaldehyde (19 mg, 0.12 mmol). Column chromatography eluting with 0–10 % EtOAc/DCM afforded compound **75** as a white solid (35 mg, 86 %). ^1^H NMR (CDCl_3_) *δ* 7.62–7.53 (m, 2H), 7.27 (d, *J* = 7.9 Hz, 2H), 6.62–6.44 (m, 2H), 6.19–6.12 (m, 1H), 3.70 (s, 2H), 3.62 (s, 2H), 2.83 (tq, *J* = 7.1, 3.7 Hz, 1H), 1.81–1.68 (m, 1H), 0.79 (td, *J* = 7.0, 5.1 Hz, 2H), 0.60–0.48 (m, 2H), 0.31 (dt, *J* = 6.4, 3.1 Hz, 2H), 0.16 (p, *J* = 4.2 Hz, 2H). ^13^C NMR (DMSO‑*d*_6_) *δ* 167.3, 163.5–162.7 (m), 160.6–159.3 (m), 141.9, 133.0, 128.3, 126.8, 111.0–110.1 (m), 101.1–99.3 (m), 59.1, 44.9, 36.1, 23.0, 6.9, 5.7. LCMS *m/z* 375.2 [M+H]. HRMS *m/z*: [M+H] Calcd for C_21_H_21_F_3_N_2_O 375.1679; Found 375.1672.

***N-Cyclopropyl-4-((cyclopropyl(2,4,6-trichlorobenzyl)amino)methyl)benzamide (78).*** General Procedure B was followed using **88** (25 mg, 0.11 mmol) and 2,4,6-trichlorobenzaldehyde (25 mg, 0.12 mmol). Column chromatography eluting with 0–10 % EtOAc/DCM afforded compound **78** as a white solid (22 mg, 48 %). ^1^H NMR (CDCl_3_) *δ* 7.61 (d, *J* = 8.0 Hz, 2H), 7.32 (d, *J* = 7.8 Hz, 2H), 7.22 (s, 2H), 6.20 (s, 1H), 3.88 (s, 2H), 3.81 (s, 2H), 2.89 (tq, *J* = 7.1, 3.6 Hz, 1H), 1.98 (d, *J* = 16.3 Hz, 1H), 0.87 (td, *J* = 7.0, 5.1 Hz, 2H), 0.67–0.56 (m, 2H), 0.29 (h, *J* = 4.5 Hz, 2H), 0.12 (s, 2H). ^13^C NMR (DMSO‑*d*_6_) *δ* 167.3, 141.5, 136.8, 133.5, 133.0, 132.8, 128.7, 127.9, 126.7, 60.2, 53.4, 36.3, 23.0, 6.6, 5.7. LCMS *m/z* 423.2 [M+H]. HRMS *m/z*: [M+H] Calcd for C_21_H_21_C_l3_N_2_O 423.0792; Found 423.0788.

***N-Cyclopropyl-4-((cyclopropyl(3,4-dimethylbenzyl)amino)methyl)benzamide (79).*** General Procedure B was followed using 3,4-dimethylbenzaldehyde (100 mg, 0.746 mmol) and **88** (206 mg, 0.896 mmol). Column chromatography eluting with 10–20 % EtOAc/hexane afforded compound **79** as a white solid (160 mg, 61 %). ^1^H NMR (DMSO‑*d*_6_) *δ* 8.37 (d, *J* = 4.2 Hz, 1H), 7.75 (d, *J* = 8.0 Hz, 2H), 7.30 (d, *J* = 8.0 Hz, 2H), 7.07 (d, *J* = 7.6 Hz, 1H), 7.01 (s, 1H), 6.97 (dd, *J* = 7.4, 1.7 Hz, 1H), 3.61 (s, 2H), 3.54 (s, 2H), 2.83 (tq, *J* = 7.8, 4.0 Hz, 1H), 2.20 (d, *J* = 4.7 Hz, 6H), 1.77 (tt, *J* = 6.7, 3.6 Hz, 1H), 0.68 (dt, *J* = 6.9, 3.3 Hz, 2H), 0.56 (p, *J* = 4.4 Hz, 2H), 0.36 (dt, *J* = 6.5, 3.2 Hz, 2H), 0.21 (p, *J* = 3.9 Hz, 2H). ^13^C NMR (DMSO‑*d*_6_) *δ* 167.4, 142.0, 135.6, 135.4, 134.5, 133.0, 130.3, 129.1, 128.7, 126.9, 126.5, 57.8, 57.4, 36.2, 23.0, 19.4, 19.0, 7.2, 5.7. LCMS *m/z* 349.3 [M+H]. HRMS *m/z*: [M+H] Calcd for C_23_H_28_N_2_O 349.2274; Found 349.227.

***N-Cyclopropyl-4-((cyclopropyl(2,4-dimethylbenzyl)amino)methyl)benzamide (80).*** General Procedure B was followed using **88** (70 mg, 0.30 mmol) and 2,4-dimethylbenzaldehyde (61 mg, 0.46 mmol). Column chromatography eluting with 10–20 % EtOAc/hexane followed by trituration with 2 M HCl in diethyl ether afforded the hydrochloride salt of compound **80** as a white solid (29 mg, 25 %). ^1^H NMR (DMSO‑*d*_6_) *δ* 10.36 (s, 1H), 8.55 (d, *J* = 4.0 Hz, 1H), 7.89 (d, *J* = 8.0 Hz, 2H), 7.74 (d, *J* = 7.9 Hz, 2H), 7.48 (d, *J* = 7.8 Hz, 1H), 7.10–7.02 (m, 2H), 4.48 (d, *J* = 5.5 Hz, 2H), 4.33 (s, 2H), 2.85 (dq, *J* = 7.3, 3.7 Hz, 1H), 2.81–2.71 (m, 1H), 2.30 (d, *J* = 10.8 Hz, 6H), 0.76–0.62 (m, 3H), 0.57 (dq, *J* = 19.4, 4.1 Hz, 5H). ^13^C NMR (DMSO‑*d*_6_) *δ* 166.9, 138.8, 138.4, 135.0, 132.9, 131.9, 131.2, 127.2, 126.4, 125.5, 58.3, 55.5, 36.5, 23.1, 20.7, 19.4, 5.7, 5.0, 4.1. LCMS *m/z* 349.3 [M+H]. HRMS *m/z*: [M+H] Calcd for C_23_H_28_N_2_O 349.2274; Found 349.2269.

***N-Cyclopropyl-4-((cyclopropyl(2,4,6-trimethylbenzyl)amino)methyl)benzamide (82).*** General Procedure B was followed using **88** (17 mg, 0.0.074 mmol) and 2,4,6-trimethylbenzaldehyde (11 mg, 0.074 mmol). Column chromatography eluting with 0–20 % EtOAc/DCM afforded compound **82** as a white solid (13 mg, 49 %). ^1^H NMR (CDCl_3_) *δ* 7.61 (d, *J* = 8.1 Hz, 2H), 7.24 (d, *J* = 8.0 Hz, 2H), 6.78 (s, 2H), 6.25 (s, 1H), 3.65 (s, 2H), 3.63 (s, 2H), 2.89 (tq, *J* = 7.1, 3.6 Hz, 1H), 2.27 (s, 6H), 2.23 (s, 3H), 1.74 (tt, *J* = 6.8, 3.7 Hz, 1H), 0.86 (td, *J* = 7.0, 5.2 Hz, 2H), 0.67–0.55 (m, 2H), 0.19 (td, *J* = 6.6, 4.5 Hz, 2H), 0.07 to −0.09 (m, 2H). ^13^C NMR (DMSO‑*d*_6_) *δ* 167.3, 142.6, 137.4, 135.4, 132.8, 132.1, 129.0, 128.5, 126.7, 59.6, 53.1, 36.7, 23.0, 20.5, 19.8, 6.1, 5.7. LCMS *m/z* 363.4 [M+H]. HRMS *m/z*: [M+H] Calcd for C_24_H_30_N_2_O 363.2431; Found 363.2425.

***N-Cyclopropyl-4-((cyclopropyl(2,4,6-trimethoxybenzyl)amino)methyl)benzamide (83).*** General Procedure B was followed using **88** (30 mg, 0.13 mmol) and 2,4,6-trimethoxybenzaldehyde (28 mg, 0.14 mmol). Column chromatography eluting with 10–30 % EtOAc/DCM afforded compound **83** as a beige solid (15 mg, 25 %). ^1^H NMR (CDCl_3_) *δ* 7.63 (d, *J* = 7.9 Hz, 2H), 7.36 (d, *J* = 7.8 Hz, 2H), 6.28 (s, 1H), 6.07 (s, 2H), 3.85–3.66 (m, 13H), 2.89 (tq, *J* = 7.1, 3.6 Hz, 1H), 1.89–1.74 (m, 1H), 0.85 (td, *J* = 7.0, 5.1 Hz, 2H), 0.61 (qd, *J* = 5.1, 3.7 Hz, 2H), 0.38–0.16 (m, 4H). ^13^C NMR (DMSO‑*d*_6_) *δ* 167.5, 160.1, 159.6, 143.5, 132.5, 128.1, 126.6, 106.8, 90.5, 58.4, 55.4, 55.1, 45.5, 36.5, 23.0, 6.9, 5.7. LCMS *m/z* 411.3 [M+H]. HRMS *m/z*: [M+H] Calcd for C_24_H_30_N_2_O_4_ 411.2278; Found 411.2272.

***4-(((3,5-Bis(trifluoromethyl)benzyl) (cyclopropyl)amino)methyl)-N-cyclopropylbenzamide (84).*** General Procedure B was followed using 3,5-bis(trifluoromethyl)benzaldehyde (30 mg, 0.12 mmol) and **88** (29 mg, 0.12 mmol). Column chromatography eluting with 10–30 % EtOAc/DCM followed by reverse phase preparative HPLC eluting with a gradient of 5–75 % MeCN/water (including 0.1 % formic acid) afforded compound **84** as a colourless semi-solid (9 mg, 16 %). ^1^H NMR (CDCl_3_) *δ* 7.63 (d, *J* = 7.9 Hz, 2H), 7.36 (d, *J* = 7.8 Hz, 2H), 6.28 (s, 1H), 6.07 (s, 2H), 3.85–3.66 (m, 13H), 2.89 (tq, *J* = 7.1, 3.6 Hz, 1H), 1.89–1.74 (m, 1H), 0.85 (td, *J* = 7.0, 5.1 Hz, 2H), 0.61 (qd, *J* = 5.1, 3.7 Hz, 2H), 0.38–0.16 (m, 4H). LCMS *m/z* 457.2 [M+H]. HRMS *m/z*: [M+H] Calcd for C_23_H_22_F_6_N_2_O 457.1709; Found 457.1702.

***N-cyclopropyl-4-((cyclopropylamino)methyl)benzamide* (88).** General Procedure B was followed using **114** (300 mg, 1.6 mmol) and cyclopropylamine (1.6 mL, 2.4 mmol) to afford compound **88** as a beige solid (197 mg, 54 %). ^1^H NMR (DMSO‑*d*_6_) *δ* 8.34 (d, *J* = 4.3 Hz, 1H), 7.75 (d, *J* = 7.8 Hz, 2H), 7.37 (d, *J* = 7.8 Hz, 2H), 3.74 (s, 2H), 2.83 (tq, *J* = 7.8, 4.1 Hz, 1H), 2.73 (s, 1H), 2.01 (tt, *J* = 6.7, 3.5 Hz, 1H), 0.68 (td, *J* = 7.0, 4.5 Hz, 2H), 0.56 (q, *J* = 3.7 Hz, 2H), 0.37–0.27 (m, 2H), 0.30–0.19 (m, 2H). ^13^C NMR (DMSO‑*d*_6_) *δ* 167.4, 144.4, 132.6, 127.6, 127.0, 52.5, 29.9, 23.0, 6.1, 5.7. LCMS *m/z* 232.2 [M+H]. HRMS *m/z*: [M+H] Calcd for C_14_H_18_N_2_O 231.1492; Found 231.1488.

***N-Cyclopropyl-4-((cyclopropyl(propyl)amino)methyl)benzamide (90).*** General Procedure B was followed using **88** (515 mg, 2.24 mmol) and propionaldehyde (130 mg, 2.24 mmol). Column chromatography eluting with 10–25 % EtOAc/hexane afforded compound **90** as a white solid (85 mg, 14 %). ^1^H NMR (DMSO‑*d*_6_) *δ* 8.36 (d, *J* = 4.2 Hz, 1H), 7.74 (d, *J* = 7.8 Hz, 2H), 7.31 (d, *J* = 7.8 Hz, 2H), 3.70 (s, 2H), 2.83 (tq, *J* = 7.9, 4.1 Hz, 1H), 2.39 (dd, *J* = 8.4, 6.4 Hz, 2H), 1.76 (tt, *J* = 6.9, 3.8 Hz, 1H), 1.46 (h, *J* = 7.4 Hz, 2H), 0.76 (t, *J* = 7.3 Hz, 3H), 0.67 (dd, *J* = 7.5, 5.0 Hz, 2H), 0.56 (q, *J* = 3.8 Hz, 2H), 0.42 (dd, *J* = 6.8, 4.5 Hz, 2H), 0.29 (p, *J* = 4.2 Hz, 2H). ^13^C NMR (DMSO‑*d*_6_) *δ* 167.4, 142.3, 132.9, 128.6, 126.9, 58.7, 56.5, 36.5, 23.0, 19.5, 11.8, 7.0, 5.7. LCMS *m/z* 273.2 [M+H]. HRMS *m/z*: [M+H] Calcd for C_17_H_24_N_2_O 273.1961; Found 273.1957.

***N-Cyclopropyl-4-((cyclopropyl(isopropyl)amino)methyl)benzamide (91).*** General Procedure B was followed using **88** (150 mg, 0.651 mmol) and acetone (0.150 mL, 1.95 mmol). Column chromatography eluting with 10–20 % EtOAc/hexane afforded compound **91** as a white solid (120 mg, 67 %). ^1^H NMR (DMSO‑*d*_6_) *δ* 8.33 (d, *J* = 4.2 Hz, 1H), 7.71 (d, *J* = 7.9 Hz, 2H), 7.31 (d, *J* = 7.9 Hz, 2H), 3.69 (s, 2H), 2.96–2.76 (m, 2H), 1.93 (tt, *J* = 6.8, 3.7 Hz, 1H), 1.02 (d, *J* = 6.6 Hz, 6H), 0.67 (dt, *J* = 6.7, 3.2 Hz, 2H), 0.55 (p, *J* = 4.4 Hz, 2H), 0.35 (dt, *J* = 6.4, 3.1 Hz, 2H), 0.16 (p, *J* = 3.9 Hz, 2H). ^13^C NMR (DMSO‑*d*_6_) *δ* 167.5, 144.5, 132.7, 128.1, 126.8, 54.7, 51.5, 33.0, 23.0, 18.6, 6.9, 5.7. LCMS *m/z* 273.3 [M+H]. HRMS *m/z*: [M+H] Calcd for C_17_H_24_N_2_O 273.1961; Found 273.1956.

***N-Cyclopropyl-4-((cyclopropyl(1-phenylethyl)amino)methyl)benzamide (97).*** To a stirred solution of **88** (150 mg, 0.651 mmol) in DMF (3 mL) at 0 °C was added K_2_CO_3_ (270 mg, 1.954 mmol) followed by (1-bromoethyl)benzene (0.050 mL, 0.88 mmol) and the mixture was allowed to warm to room temperature over 15 min and stirred for 6 h. On completion, the reaction mixture was diluted with EtOAc, washed with cold water (2 × 10 mL) and brine. The organic phase was dried over anhydrous Na_2_SO_4_ and concentrated under reduced pressure. Column chromatography eluting with 10–25 % EtOAc/hexane afforded compound **97** as a white solid (30 mg, 21 %). ^1^H NMR (DMSO‑*d*_6_) *δ* 8.33 (d, *J* = 4.2 Hz, 1H), 7.71 (d, *J* = 7.9 Hz, 2H), 7.35–7.33 (m, 4H), 7.30–7.19 (m, 3H), 3.88 (q, *J* = 6.9 Hz, 1H), 3.74 (d, *J* = 14.0 Hz, 1H), 3.41 (d, *J* = 14.0 Hz, 1H), 2.82 (dt, *J* = 7.3, 4.2 Hz, 1H), 1.84–1.73 (m, 1H), 1.41 (d, *J* = 6.9 Hz, 3H), 0.67 (dt, *J* = 6.9, 3.3 Hz, 2H), 0.56 (q, *J* = 3.8 Hz, 2H), 0.35 (d, *J* = 6.6 Hz, 2H), 0.16 (d, *J* = 27.5 Hz, 2H). ^13^C NMR (DMSO‑*d*_6_) *δ* 167.4, 143.5, 142.5, 132.8, 128.4, 127.9, 127.8, 126.8, 126.8, 60.3, 55.1, 33.9, 23.0, 17.0, 7.6, 7.0, 5.7. LCMS *m/z* 335.3 [M+H]. HRMS *m/z*: [M+H] Calcd for C_22_H_26_N_2_O 335.2118; Found 335.211.

***4-(1-(Benzyl(cyclopropyl)amino)ethyl)-N-cyclopropylbenzamide (98).*** General Procedure C was followed using **133** (230 mg, 0.770 mmol) and cyclopropylamine (64 μL, 0.92 mmol). Column chromatography eluting with 20–30 % EtOAc/hexane followed by trituration with pentane/DCM (7:3) afforded compound **98** as a white solid (51 mg, 20 %). ^1^H NMR (DMSO‑*d*_6_) *δ* 8.38 (d, *J* = 4.2 Hz, 1H), 7.78 (d, *J* = 8.0 Hz, 2H), 7.40 (d, *J* = 7.9 Hz, 2H), 7.31–7.24 (m, 2H), 7.24–7.17 (m, 3H), 3.92 (q, *J* = 6.9 Hz, 1H), 3.70 (d, *J* = 13.8 Hz, 1H), 3.37–3.31 (m, 1H), 2.84 (tt, *J* = 7.3, 3.9 Hz, 1H), 1.76 (dq, *J* = 6.9, 3.4 Hz, 1H), 1.41 (d, *J* = 6.9 Hz, 3H), 0.68 (dt, *J* = 6.9, 3.3 Hz, 2H), 0.56 (p, *J* = 4.5 Hz, 2H), 0.39–0.30 (m, 2H), 0.24–0.16 (m, 1H), 0.11 (dt, *J* = 9.3, 4.3 Hz, 1H). ^13^C NMR (DMSO‑*d*_6_) *δ* 167.5, 146.1, 139.8, 132.9, 128.8, 127.9, 127.6, 126.9, 126.6, 59.9, 55.5, 33.7, 23.0, 16.9, 7.9, 6.8, 5.8. LCMS *m/z* 335.3 [M+H]. HRMS *m/z*: [M+H] Calcd for C_22_H_26_N_2_O 335.2118; Found 335.2112.

***N-Cyclopropyl-N-(4-(cyclopropylcarbamoyl)benzyl)benzamide (99).*** To a stirred solution of **88** (50 mg, 0.22 mmol) and triethylamine 0.061 mL, 0.43 mmol) in DCM (3.0 mL) at rt under an N_2_ atmosphere, benzoyl chloride (0.030 mL, 0.26 mmol) was added dropwise. The solution was then allowed to stir for 1 h before being diluted with DCM (10 mL) and washed with saturated aqueous NaHCO_3_ (10 mL) and brine (10 mL). The organic fraction was then dried over MgSO_4_, filtered and concentrated. Column chromatography eluting with 0–30 % EtOAc/DCM afforded compound **99** as a colourless oil (36 mg, 50 %). ^1^H NMR (CDCl_3_) *δ* 7.78–7.68 (m, 2H), 7.53–7.31 (m, 7H), 6.38 (s, 1H), 4.76 (s, 2H), 2.89 (tq, *J* = 7.0, 3.6 Hz, 1H), 2.58 (tt, *J* = 6.9, 4.1 Hz, 1H), 0.86 (td, *J* = 7.0, 5.2 Hz, 2H), 0.71–0.37 (m, 6H). ^13^C NMR (DMSO‑*d*_6_) *δ* 171.6, 167.2, 141.4, 137.3, 133.2, 129.4, 128.0, 127.4, 127.0, 126.9, 66.3, 49.8, 31.4, 23.0, 9.4, 5.7. LCMS *m/z* 335.2 [M+H]. HRMS *m/z*: [M+H] Calcd for C_21_H_22_N_2_O_2_ 335.1754; Found 335.1755.

***N*** [4]***-Benzyl-N***^***1***^***,N*** [4]***-dicyclopropyl-terephthalamide (100).*** Compound **147** (34 mg, 0.17 mmol), *N*-benzylcyclopropanamine (29 mg, 0.20 mmol), DIPEA (0.088 mL, 0.503 mmol) and HATU (94 mg, 0.25 mmol) were dissolved in DCM (2 mL) and stirred for 2 h at rt. The reaction was quenched with 10 % aq. Citric acid (5 mL) and diluted with additional DCM (10 mL). The organic fraction was separated and washed with sat. aq. NaHCO_3_ (10 mL) and brine (10 mL), dried with Na_2_SO_4_, filtered and concentrated. Column chromatography eluting with 70–100 % EtOAc/DCM afforded compound **100** as a white solid (24 mg, 43 %). ^1^H NMR (CDCl_3_): *δ* 7.70–7.80 (m, *J =* 8.3 Hz, 2H), 7.45–7.57 (m, *J =* 8.2 Hz, 2H), 7.29–7.42 (m, 5H), 6.45 (br. s., 1H), 4.75 (br. s., 2H), 2.92 (dt, *J* = 3.4, 7.1 Hz, 1H), 2.49–2.64 (m, 1H), 0.82–0.93 (m, 2H), 0.61–0.69 (m, 2H), 0.58–0.35 (4H). LCMS *m/z* 335.2 [M+H]. HRMS *m/z*: [M+H] Calcd for C_21_H_22_N_2_O_2_ 335.1754; Found 335.1755.

***4-(N-Benzyl-N-cyclopropylsulfamoyl)-N-cyclopropylbenzamide (102).*** General Procedure C was followed using **134** (100 mg, 0.357 mmol) and cyclopropylamine (36 mL, 0.428 mmol). The crude product was triturated with diethyl ether/*n*-pentane (1:3) to afford compound **102** as a white solid (83 mg, 63 %). ^1^H NMR (DMSO‑*d*_6_) *δ* 8.71 (d, *J* = 4.3 Hz, 1H), 8.01 (d, *J* = 8.4 Hz, 2H), 7.90 (d, *J* = 8.4 Hz, 2H), 7.39–7.24 (m, 5H), 4.34 (s, 2H), 2.87 (tq, *J* = 7.9, 4.0 Hz, 1H), 2.05 (ddd, *J* = 8.9, 6.9, 3.8 Hz, 1H), 0.71 (dt, *J* = 6.9, 3.3 Hz, 2H), 0.63–0.46 (m, 6H). ^13^C NMR (DMSO‑*d*_6_) *δ* 166.4, 140.3, 138.4, 137.2, 128.4, 128.3, 128.2, 127.5, 127.4, 53.8, 30.7, 23.3, 6.8, 5.8. LCMS *m/z* 371.3 [M+H]. HRMS *m/z*: [M+H] Calcd for C_20_H_22_N_2_O_3_S 371.1424; Found 371.1418.

***5-((Benzyl(cyclopropyl)amino)methyl)-N-cyclopropylpicolinamide (103). General Procedure E:*** A small microwave vessel was charged with **144** (50 mg, 0.17 mmol), cyclopropylamine (0.018 mL, 0.25 mmol), bis(trimethylaluminum)-1,4-diazabicyclo[2.2.2]octane adduct (65 mg, 0.25 mmol) and anhydrous toluene (2 mL). The mixture was stirred and heated to 130 °C at 296 W in a microwave reactor for 8 min. The reaction did not go to completion and the vessel was recharged with bis(trimethylaluminum)-1,4-diazabicyclo[2.2.2]octane adduct (65 mg, 0.25 mmol) and reacted again under the same conditions for an additional 8 m. The resulting mixture was then quenched by the addition of water (10 mL) and extracted with DCM (3 × 5 mL). Organic fractions were then combined and washed with saturated aqueous NaHCO_3_ (5 mL) and brine (5 mL), dried over MgSO_4_, filtered and concentrated. Column chromatography eluting with 0–10 % EtOAc/DCM afforded compound **103** as a beige solid (7 mg, 13 %). ^1^H NMR (CDCl_3_) *δ* 7.72–7.62 (m, 2H), 7.39–7.15 (m, 4H), 7.13–6.94 (m, 2H), 6.25 (s, 1H), 3.76–3.69 (m, 4H), 2.90 (tq, *J* = 7.1, 3.6 Hz, 1H), 1.92–1.75 (m, 1H), 0.95–0.78 (m, 2H), 0.72–0.55 (m, 2H), 0.48–0.21 (m, 4H). LCMS *m/z* 322.2 [M+H]. HRMS *m/z*: [M+H] Calcd for C_20_H_23_N_3_O 322.1914; Found 322.1916.

***6-((Benzyl(cyclopropyl)amino)methyl)-N-cyclopropylnicotinamide (104).*** General Procedure E was followed using **145** (100 mg, 0.34 mmol). Column chromatography eluting with 30–60 % EtOAc/DCM afforded compound **104** as a white solid (51 mg, 47 %). ^1^H NMR (CDCl_3_) *δ* 8.82 (dd, *J* = 2.4, 0.8 Hz, 1H), 7.99 (dd, *J* = 8.1, 2.3 Hz, 1H), 7.40 (dd, *J* = 8.2, 0.8 Hz, 1H), 7.34–7.15 (m, 5H), 6.46 (s, 1H), 3.85 (s, 2H), 3.74 (s, 2H), 2.89 (tq, *J* = 7.1, 3.6 Hz, 1H), 1.91 (tt, *J* = 6.5, 3.8 Hz, 1H), 0.94–0.77 (m, 2H), 0.71–0.57 (m, 2H), 0.45–0.23 (m, 4H). ^13^C NMR (DMSO‑*d*_6_) *δ* 166.0, 161.9, 147.4, 138.0, 134.9, 129.2, 128.1, 127.9, 126.8, 122.7, 59.4, 58.3, 36.5, 22.9, 7.3, 5.7. LCMS *m/z* 322.2 [M+H]. HRMS *m/z*: [M+H] Calcd for C_20_H_23_N_3_O 322.1914; Found 322.1917.

***2-((Benzyl(cyclopropyl)amino)methyl)-N-cyclopropylthiazole-5-carboxamide (105).*** General Procedure B was followed using **139** (120 mg, 0.506 mmol) and benzaldehyde (0.100 mL, 1.01 mmol). Column chromatography eluting with 25–40 % EtOAc/hexane followed by reverse phase preparative HPLC eluting with 10–60 % MeCN/water (including 0.1 % formic acid) afforded compound **105** as a white solid (37 mg, 23 %). ^1^H NMR (DMSO‑*d*_6_) *δ* 8.57 (d, *J* = 3.8 Hz, 1H), 8.19 (s, 1H), 7.38–7.22 (m, 5H), 3.93 (s, 2H), 3.76 (s, 2H), 2.78 (dq, *J* = 7.5, 3.8 Hz, 1H), 2.00 (dq, *J* = 7.1, 3.5 Hz, 1H), 0.70 (td, *J* = 7.1, 4.7 Hz, 2H), 0.55 (p, *J* = 4.6 Hz, 2H), 0.42 (dd, *J* = 6.6, 4.5 Hz, 2H), 0.33 (q, *J* = 3.4 Hz, 2H). ^13^C NMR (DMSO‑*d*_6_) *δ* 174.1, 161.1, 142.6, 137.5, 135.2, 129.3, 128.0, 127.1, 58.2, 55.1, 36.5, 22.8, 7.2, 5.7. LCMS *m/z* 328.2 [M+H]. HRMS *m/z*: [M+H] Calcd for C_18_H_21_N_3_OS 328.1478; Found 328.1472.

***5-((Benzyl(cyclopropyl)amino)methyl)-N-cyclopropylthiazole-2-carboxamide (106).*** To a stirred solution of **143** (500 mg, 3.33 mmol) in DMSO (25 mL) in a sealed tube was added cyclopropylamine (209 mg, 3.66 mmol). The vessel was closed, and the mixture was heated at 70 °C for approximately 16 h. On completion, the mixture was cooled to room temperature, diluted with EtOAc (25 mL), washed with water (2 × 25 mL) and brine (10 mL). The organic fraction was dried over Na_2_SO_4_ and concentrated under reduced pressure. Crude residue was purified by column chromatography eluting with 30–40 % EtOAc/hexane followed by reverse phase preparative HPLC eluting 20–100 % MeCN/water (including 20 mM ammonium bicarbonate) in to afford compound **106** as a brownish gum (18 mg, 9 %). ^1^H NMR (DMSO‑*d*_6_) *δ* 8.68 (s, 1H), 7.71 (s, 1H), 7.32–7.19 (m, 5H), 3.80 (s, 2H), 3.73 (s, 2H), 2.89–2.81 (m, 1H), 1.92–1.84 (m, 1H), 0.69 (d, *J* = 5.6 Hz, 4H), 0.37 (d, *J* = 5.6 Hz, 2H), 0.23 (d, *J* = 3.0 Hz, 2H).^13^C NMR (DMSO‑*d*_6_) *δ* 162.9, 160.5, 155.5, 138.3, 129.3, 128.0, 126.9, 122.5, 58.4, 53.4, 36.1, 23.0, 7.3, 5.8. LCMS *m/z* 328.3 [M+H]. HRMS *m/z*: [M+H] Calcd for C_18_H_21_N_3_OS 328.1478; Found 328.1472.

***4-(((4-(tert-Butyl)benzyl) (propyl)amino)methyl)-N-cyclopropylbenzamide (107).*** General Procedure B was followed using **115** (45 mg, 0.19 mmol) and 2,4,6-trichlorobenzaldehyde (31 mg, 0.19 mmol). Column chromatography eluting with 0–10 % EtOAc/DCM afforded a beige solid. This material was further purified by reverse phase preparatory HPLC eluting with 5–100 % MeCN/water (including 0.1 % formic acid) to which afforded compound **107** as a white solid (19 mg, 73 %). ^1^H NMR (DMSO‑*d*_6_) *δ* 8.37 (d, *J* = 4.2 Hz, 1H), 7.75 (d, *J* = 8.1 Hz, 2H), 7.46–7.18 (m, 6H), 3.50 (d, *J* = 18.0 Hz, 4H), 2.82 (tt, *J* = 7.8, 3.9 Hz, 1H), 2.29 (t, *J* = 7.2 Hz, 2H), 1.47 (h, *J* = 7.3 Hz, 2H), 1.25 (s, 9H), 0.78 (t, *J* = 7.3 Hz, 3H), 0.75–0.48 (m, 4H). ^13^C NMR (CDCl_3_) *δ* 169.0, 149.8, 144.0, 136.4, 133.0, 128.8, 128.4, 126.9, 125.1, 57.9, 57.9, 55.5, 34.5, 31.5, 23.2, 20.1, 11.8, 6.7. LCMS *m/z* 379.4 [M+H]. HRMS *m/z*: [M+H] Calcd for C_25_H_34_N_2_O 379.2744; Found 379.2738.

***N-Cyclopropyl-4-((propyl(4-(trifluoromethyl)benzyl)amino)methyl)benzamide (108).*** General Procedure B was followed using **115** (40 mg, 0.17 mmol) and 4-(trifluoromethyl)benzaldehyde (30 mg, 0.19 mmol). Column chromatography eluting with 0–10 % EtOAc/DCM afforded compound **108** as a colourless semi-solid (30 mg, 45 %). ^1^H NMR (CDCl_3_) *δ* 7.72–7.65 (m, 2H), 7.54 (d, *J* = 8.1 Hz, 2H), 7.45 (d, *J* = 8.1 Hz, 2H), 7.42–7.34 (m, 2H), 6.33 (s, 1H), 3.56 (s, 4H), 2.87 (tt, *J* = 7.1, 3.5 Hz, 1H), 2.36 (dd, *J* = 8.1, 6.4 Hz, 2H), 1.51 (h, *J* = 7.4 Hz, 2H), 0.91–0.78 (m, 5H), 0.61 (pd, *J* = 5.5, 3.0 Hz, 2H). ^13^C NMR (CDCl_3_) *δ* 168.9, 144.3, 143.7, 133.2, 129.2 (q, *J* = 32.2 Hz), 128.9, 128.8, 127.0, 125.2 (q, *J* = 3.8 Hz), 124.4 (q, *J* = 270 Hz), 58.2, 58.1, 55.8, 23.2, 20.3, 11.9, 6.9. LCMS *m/z* 391.2 [M+H]. HRMS *m/z*: [M+H] Calcd for C_22_H_25_F_3_N_2_O 391.1992; Found 391.1984.

***4-(((3,5-Bis(trifluoromethyl)benzyl) (propyl)amino)methyl)-N-cyclopropylbenzamide (109).*** To a stirred solution of **111** (30 mg, 0.14 mmol) and **116** (41 mg, 0.14 mmol) in DMF (2 mL) was added K_2_CO_3_ (39 mg, 0.28 mmol) and the mixture was heated to 90 °C in a sealed screw-top vial for 24 h. Volatiles were removed under reduced pressure and the residue transferred to a separatory funnel by EtOAc (10 mL) and washed with water (2 × 10 mL) and brine (5 mL) before being dried over MgSO_4_, filtered and concentrated. Column chromatography eluting with 0–10 % EtOAc/DCM followed by reverse phase preparatory HPLC eluting with 5–50 % MeCN/water (including 0.1 % formic acid) afforded compound **109** as a white solid (21 mg, 32 %). ^1^H NMR (CDCl_3_) *δ* 7.80 (s, 2H), 7.73 (s, 1H), 7.71–7.65 (m, 2H), 7.41–7.33 (m, 2H), 6.27 (s, 1H), 3.61 (d, *J* = 11.9 Hz, 4H), 2.89 (tq, *J* = 7.1, 3.6 Hz, 1H), 2.39 (dd, *J* = 8.1, 6.3 Hz, 2H), 1.53 (h, *J* = 7.3 Hz, 2H), 0.85 (td, *J* = 7.1, 4.7 Hz, 5H), 0.66–0.56 (m, 2H). ^13^C NMR (CDCl_3_) *δ* 168.8, 143.1, 143.0, 133.5, 131.6 (q, *J* = 33.1 Hz), 128.9, 128.6, 127.1, 123.6 (q, *J* = 272.6 Hz), 121.2–120.9 (m), 77.4, 58.3, 57.8, 56.0, 23.2, 20.3, 11.8, 6.9. LCMS *m/z* 459.2 [M+H]. HRMS *m/z*: [M+H] Calcd for C_23_H_24_F_6_N_2_O 459.1866; Found 459.1857.

***N-Cyclopropyl-4-((propyl(2,4,6-trimethylbenzyl)amino)methyl)benzamide (110).*** General Procedure B was followed using 2,4,6-trimethylbenzaldehyde (0.029 mL, 0.196 mmol) and **115** (0.063 mL, 0.196 mmol). Column chromatography eluting with 0–5% MeOH in DCM followed by reverse phase preparatory HPLC eluting with 5–50 % MeCN/water (including 0.1 % formic acid) afforded compound **110** as a white solid (9 mg, 13 %). ^1^H NMR (CDCl_3_) *δ* 7.81 (d, *J* = 7.2 Hz, 2H), 7.58 (d, *J* = 7.5 Hz, 2H), 6.88 (s, 2H), 6.58 (s, 1H), 4.30 (s, 2H), 4.20 (s, 2H), 2.91 (t, *J* = 8.4 Hz, 3H), 2.24 (d, *J* = 8.0 Hz, 9H), 0.89 (t, *J* = 7.0 Hz, 5H), 0.66 (t, *J* = 2.9 Hz, 2H). ^13^C NMR (DMSO‑*d*_6_) *δ* 167.3, 143.2, 137.4, 135.5, 132.8, 132.1, 128.7, 128.3, 126.9, 57.0, 55.3, 52.1, 23.0, 20.5, 19.8, 19.5, 11.8, 5.7. LCMS *m/z* 365.4 [M+H]. HRMS *m/z*: [M+H] Calcd for C_24_H_32_N_2_O 365.2587; Found 365.258.

***4-(Chloromethyl)-N-cyclopropylbenzamide (111). General Procedure F:*** To a stirred solution of 4-(chloromethyl)benzoic acid (2.0 g, 12 mmol) and DMF (2 drops) in DCM (15 mL), oxalyl chloride (2.0 mL, 23 mmol) was added slowly. The solution was allowed to stir at rt for 2 h before volatiles were removed *via* rotary evaporation and the crude resin redissolved in DCM (5 mL) and cooled to 0 °C on an ice bath. Separately, a solution of cyclopropylamine (0.97 mL, 14 mmol) and triethylamine (2.5 mL, 18 mmol) was prepared in DCM (15 mL) and also cooled to 0 °C on an ice bath before the crude acid chloride solution was added dropwise *via* syringe. The final solution was allowed to warm to rt and stirred for 2 h before aqueous 1 M HCl (5 mL) was added and the organics separated and sequentially washed with brine (5 mL), saturated aqueous NaHCO_3_ (5 mL), and brine (5 mL). The organics were then dried over MgSO_4_, filtered and concentrated to afford compound **111** as an off-white solid (2.13 g, 87 %). ^1^H NMR (CDCl_3_) *δ* 7.80–7.67 (m, 2H), 7.48–7.38 (m, 2H), 6.26 (s, 1H), 4.60 (s, 2H), 2.90 (tq, *J* = 7.1, 3.6 Hz, 1H), 0.87 (td, *J* = 7.0, 5.2 Hz, 2H), 0.68–0.56 (m, 2H). LCMS *m/z* 210.2 [M+H].

***N-(3-Chlorobenzyl)cyclopropanamine (112). General Procedure G:*** To a stirred solution of 3-chlorobenzaldehyde (80 mg, 0.57 mmol) and cyclopropylamine (0.59 mL, 0.85 mmol) in MeOH (2 mL), cooled to 0 °C on an ice bath and under an N_2_ atmosphere, was added AcOH (0.049 mL, 0.85 mmol). The solution was allowed to stir for 15 m before NaBH_4_ (32 mg, 085 mmol) was added portion-wise. The mixture was allowed to warm to rt and stirred for 2 h before being quenched by saturated aqueous NaHCO_3_ (5 mL) and extracted by DCM (4 × 5 mL). The organic fractions were then combined and washed with brine (5 mL), dried over MgSO_4_, filtered and concentrated. Column chromatography eluting with 0–5% MeOH/DCM afforded compound **112** as a colourless oil (91 mg, 88 %). ^1^H NMR (CDCl_3_) *δ* 7.34–7.30 (m, 1H), 7.28–7.16 (m, 4H), 3.82 (s, 2H), 2.14 (tt, *J* = 6.5, 3.7 Hz, 1H), 0.54–0.33 (m, 4H). LCMS *m/z* 182.2 [M+H].

***N-(4-Chlorobenzyl)cyclopropanamine (113).*** General Procedure G was followed using 4-chlorobenzaldehyde (100 mg, 0.71 mmol). Column chromatography eluting with 0–5% MeOH/DCM afforded compound **113** as a colourless oil (78 mg, 60 %).

***N-Cyclopropyl-4-formylbenzamide (114).*** General Procedure F was followed using 4-formylbenzoic acid (0.5 g, 3.3 mmol) to afford compound **114** as a beige solid (0.55 g, 87 %). ^1^H NMR (CDCl_3_) *δ* 10.05 (s, 1H), 7.89 (s, 4H), 6.56 (s, 1H), 2.91 (tq, *J* = 7.2, 3.6 Hz, 1H), 0.87 (td, *J* = 7.0, 5.2 Hz, 2H), 0.75–0.58 (m, 2H). LCMS *m/z* 190.2 [M+H].

***N-Cyclopropyl-4-((propylamino)methyl)benzamide (115).*** To a stirred solution of **111** (137 mg, 0.653 mmol) in MeCN (3 mL) in a small screw-top vial was added *N*-propylamine (0.269 mL, 3.37 mmol) and K_2_CO_3_ (271 mg, 1.96 mmol). The vial was sealed and heated to 80 °C for 16 h at which point the mixture was diluted with EtOAc (10 mL) and transferred to a separatory funnel. The organics were washed with water (10 mL) and brine (10 mL), dried over MgSO_4_, filtered and concentrated. Column chromatography eluting with 30–60 % EtOAc/heptane afforded compound **115** as a colourless semi-solid (18 mg, 43 %). ^1^H NMR (CDCl_3_) *δ* 7.70–7.60 (m, 2H), 7.42–7.33 (m, 2H), 7.29 (d, *J* = 2.0 Hz, 3H), 7.26–7.16 (m, 2H), 6.26 (s, 1H), 3.48 (d, *J* = 7.7 Hz, 4H), 2.84 (tq, *J* = 7.1, 3.6 Hz, 1H), 2.12 (s, 3H), 0.81 (td, *J* = 7.1, 5.3 Hz, 2H), 0.62–0.50 (m, 2H). ^13^C NMR (CDCl_3_) *δ* 168.9, 143.3, 139.1, 133.3, 129.1, 129.0, 128.4, 127.2, 127.0, 62.0, 61.5, 42.4, 23.2, 6.9. LCMS *m/z* 295.2 [M+H].

***N-(3,5-Bis(trifluoromethyl)benzyl)propan-1-amine (116).*** General Procedure G was followed using 3,5-bis(trifluoromethyl)benzaldehyde (100 mg, 0.42 mmol) and propylamine (34 μL, 0.42 mmol). The colourless oil (**116**) afforded by the workup was used without further purification (110 mg, 93 %). LCMS *m/z* 286.2 [M+H].

***N-Cyclopropyl-4-((isopropylamino)methyl)benzamide (117).*** General Procedure G was followed using **114** (170 mg, 0.898 mmol) and isopropylamine (0.230 mL, 2.70 mmol) to afford compound **117** as a light-yellow oil which was used for the next step without further purification (150 mg, 72 %). ^1^H NMR (DMSO‑*d*_6_) *δ* 8.36 (d, *J* = 3.8 Hz, 1H), 7.74 (d, *J* = 8.1 Hz, 2H), 7.38 (d, *J* = 8.1 Hz, 2H), 3.72 (s, 2H), 2.85–2.80 (m, 1H), 2.70–2.64 (m, 1H), 0.99 (d, *J* = 6.2 Hz, 6H), 0.70–0.65 (m, 2H), 0.57–0.53 (m, 2H). LCMS *m/z* 233.0 [M+H].

***N-(2,4-Dichlorobenzyl)cyclopropanamine (118).*** General Procedure G was followed using 2,4-dichlorobenzaldehyde (2.00 g, 11.4 mmol) and cyclopropylamine (1.36 mL, 17.1 mmol) to afford compound **118** as a brown gum which was used immediately in the next step without further purification (1.73 g, 70 %). LCMS *m/z* 216.0 [M+H].

***Methyl 4-((cyclopropyl(2,4-dichlorobenzyl)amino)methyl)benzoate (119).*** General Procedure B was followed using **118** (1.71 g, 7.92 mmol) and methyl 4-formylbenzoate (1.3 g, 7.92 mmol). Column chromatography eluting with 15–20 % EtOAc/hexane afforded compound **119** as an off-white solid (1.9 g, 66 %). ^1^H NMR (DMSO‑*d*_6_): *δ* 7.88 (d, *J* = 8.0 Hz, 2H), 7.52 (d, *J* = 2.0 Hz, 1H), 7.46–7.34 (m, 4H), 3.84 (s, 3H), 3.76 (s, 2H), 3.74 (s, 2H), 1.94–1.86 (m, 1H), 0.37–0.31 (m, 2H), 0.17–0.10 (m, 2H). LCMS *m/z* 364.2 [M+H].

***4-((Cyclopropyl(2,4-dichlorobenzyl)amino)methyl)benzoic acid (120).*** To a stirred solution of **119** (1.90 g, 5.22 mmol) in THF (8 mL) and MeOH (8 mL) was added LiOH·H_2_O (1.09 g, 26.1 mmol, dissolved in 4 mL deionized water) at 0 °C. The reaction mixture was warmed to rt, heated to 70 °C and stirred for another 3 h. On completion, the reaction mixture was cooled to rt and concentrated. Water was added to the residue and it was acidified with 10 % aq. Citric acid solution (pH < 5) was added and extracted with EtOAc (4 × 10 mL). Organic extracts were combined, washed with brine, dried over anhydrous Na_2_SO_4_ and concentrated to afford compound **120** as an off-white solid (1.66 g, 91 %). ^1^H NMR (DMSO‑*d*_6_) *δ* 12.86 (br s, 1H), 7.86 (d, *J* = 8.0 Hz, 2H), 7.53 (s, 1H), 7.45 (d, *J* = 8.0 Hz, 1H), 7.42–7.35 (m, 3H), 3.75 (s, 2H), 3.73 (s, 2H), 1.96–1.88 (m, 1H), 0.35–0.30 (m, 2H), 0.18–0.12 (m, 2H). LCMS *m/z* 350.3 [M+H].

***N-Benzylbutan-2-amine (121).*** General Procedure G was followed using butan-2-amine (0.150 mL, 1.41 mmol) to afford compound **121** as a colourless oil which was used for the next step without further purification (130 mg, 84 %). ^1^H NMR (DMSO‑*d*_6_): *δ* 7.34–7.17 (m, 5H), 3.72 (d, *J* = 13.2 Hz, 1H), 3.65 (d, *J* = 13.2 Hz, 1H), 2.51–2.42 (m, 1H), 1.84 (br s, 1H), 1.50–1.38 (m, 1H), 1.31–1.21 (m, 1H), 0.96 (d, *J* = 6.2 Hz, 3H), 0.83 (t, *J* = 7.6 Hz, 3H). LCMS *m/z* 164.0 [M+H].

***N-Benzyl-2,2,2-trifluoroethan-1-amine (122).*** General Procedure G was followed using 2,2,2-trifluoroethan-1-amine (0.110 mL, 1.41 mmol) to afford compound **122** as a colourless oil which was used in the next step without further purification (90 mg, 50 %). ^1^H NMR (DMSO‑*d*_6_): *δ* 7.34–7.19 (m, 5H), 3.77 (d, *J* = 6.0 Hz, 2H), 3.23–3.10 (m, 2H), 2.89 (br s, 1H). LCMS *m/z* 190.1 [M+H].

***N-Cyclopropyl-3-formylbenzamide (123).*** General Procedure C was followed using 3-formylbenzoic acid (500 mg, 3.33 mmol) and cyclopropylamine (0.35 mL, 5.0 mmol). Column chromatography eluting with 15–20 % EtOAc/hexane afforded compound **123** as a white solid (320 mg, 51 %). ^1^H NMR (DMSO‑*d*_6_): *δ* 10.06 (s, 1H), 8.70–8.63 (m, 1H), 8.34 (s, 1H), 8.12 (d, *J* = 8.0 Hz, 1H), 8.04 (d, *J* = 8.0 Hz, 1H), 7.69 (t, *J* = 8.0 Hz, 1H), 2.91–2.82 (m, 1H), 0.78–0.68 (m, 2H), 0.66–0.52 (m, 2H). LCMS *m/z* 190.3 [M+H].

***N-Cyclopropyl-4-((cyclopropyl(2-nitrobenzyl)amino)methyl)benzamide (124).*** General Procedure B was followed using **88** (90 mg, 0.39 mmol) and 2-nitrobenzaldehyde (89 mg, 0.59 mmol). Column chromatography eluting with 15–25 % EtOAc/hexane followed by trituration with diethyl ether and *n*-pentane (7:3) afforded compound **124** as a white solid (33 mg, 23 %). ^1^H NMR (DMSO‑*d*_6_): *δ* 8.37 (d, *J* = 4.0 Hz, 1H), 7.80 (d, *J* = 8.0 Hz, 1H), 7.72 (d, *J* = 8.0 Hz, 2H), 7.64–7.56 (m, 2H), 7.54–7.47 (m, 1H), 7.23 (d, *J* = 8.0 Hz, 2H), 3.92 (s, 2H), 3.60 (s, 2H), 2.85–2.79 (m, 1H), 1.79–1.72 (m, 1H), 0.70–0.65 (m, 2H), 0.57–0.54 (m, 2H), 0.25–0.22 (m, 2H), 0.05–0.00 (m, 2H). LCMS *m/z* 366.3 [M+H].

***4-(((2-Aminobenzyl) (cyclopropyl)amino)methyl)-N-cyclopropylbenzamide (125). General Procedure H:*** To a stirred solution of **124** (100 mg, 0.274 mmol) in EtOH (6 mL) and water (2 mL) was added ammonium chloride (146 mg, 2.74 mmol) followed by iron powder (150 mg, 2.74 mmol) and the mixture was refluxed for 2 h. On completion, the reaction mixture was cooled to rt, diluted with EtOH (10 mL) and filtered through a pad of Celite. The filtrate was concentrated under vacuo, water (10 mL) was added to the residue and it was then extracted with DCM (3 × 10 mL). Combined organic fractions were washed with brine, dried over anhydrous Na_2_SO_4_ and concentrated under reduced pressure to afford compound **125** as a brown gum (55 mg, 60 %). The next step was performed without further purification as decomposition occurred upon storage. LCMS *m/z* 336.2 [M+H].

***N-Cyclopropyl-4-((cyclopropyl(3-nitrobenzyl)amino)methyl)benzamide (126).*** General Procedure B was followed using **88** (200 mg, 0.868 mmol) and 3-nitrobenzaldehyde (197 mg, 1.30 mmol). Column chromatography eluting with 15–30 % EtOAc/hexane followed by trituration with diethyl ether and *n*-pentane (7:3) afforded compound **126** as an off-white solid (238 mg, 75 %). ^1^H NMR (DMSO‑*d*_6_): *δ* 8.37 (d, *J* = 3.2 Hz, 1H), 8.19–8.06 (m, 2H), 7.79–7.65 (m, 3H), 7.60 (t, *J* = 8.0 Hz, 1H), 7.33 (d, *J* = 8.0 Hz, 2H), 3.76 (s, 2H), 3.70 (s, 2H), 2.90–2.74 (m, 1H), 1.93–1.79 (m, 1H), 0.75–0.47 (m, 4H), 0.45–0.11 (m, 4H). LCMS *m/z* 366.3 [M+H].

***4-(((3-Aminobenzyl) (cyclopropyl)amino)methyl)-N-cyclopropylbenzamide (127).*** General Procedure H was followed using **126** (220 mg, 4.11 mmol). Compound **127** was afforded as a brown gum (59 mg, 43 %). The next step was performed without further purification as decomposition occurred upon storage. ^1^H NMR (DMSO‑*d*_6_): *δ* 8.37 (d, *J* = 3.2 Hz, 1H), 7.75 (d, *J* = 8.0 Hz, 2H), 7.31 (d, *J* = 8.0 Hz, 2H), 6.97–6.92 (m, 1H), 6.52 (s, 1H), 6.47–6.40 (m, 2H), 4.98 (br s, 2H), 3.62 (s, 2H), 3.46 (s, 2H), 2.90–2.77 (m, 1H), 1.81–1.77 (m, 1H), 0.75–0.66 (m, 2H), 0.63–0.57 (m, 2H), 0.43–0.35 (m, 2H), 0.27–0.19 (m, 2H). LCMS *m/z* 336.3 [M+H].

***N-Cyclopropyl-4-((cyclopropyl(4-nitrobenzyl)amino)methyl)benzamide (128).*** General Procedure B was followed using **88** (100 mg, 0.434 mmol, 1.0 equiv) and 3-nitrobenzaldehyde (183 mg, 0.661 mmol, 1.5 equiv). Column chromatography eluting with 15–30 % EtOAc/hexane afforded compound **128** as a light-yellow solid (65 mg, 27 %). ^1^H NMR (DMSO‑*d*_6_): *δ* 8.38 (d, *J* = 4.0 Hz, 1H), 8.17 (d, *J* = 8.4 Hz, 2H), 7.76 (d, *J* = 8.0 Hz, 2H), 7.54 (d, *J* = 8.4 Hz, 2H), 7.33 (d, *J* = 8.0 Hz, 2H), 3.74 (s, 2H), 3.69 (s, 2H), 2.87–2.78 (m, 1H), 1.90–1.81 (m, 1H), 0.71–0.64 (m, 2H), 0.61–0.53 (m, 2H), 0.42–0.35 (m, 2H), 0.27–0.18 (m, 2H). LCMS *m/z* 366.3 [M+H].

***Methyl 3-((cyclopropyl(4-(cyclopropylcarbamoyl)benzyl)amino)methyl)benzoate (129).*** General Procedure B was followed using **88** (150 mg, 0.651 mmol) and methyl 3-formylbenzoate (160 mg, 0.977 mmol). Column chromatography eluting with 15–25 % EtOAc/hexane followed by trituration with 2 N HCl in diethyl ether afforded the mono hydrochloride salt of compound **129** as a white solid (219 mg, 81 %). ^1^H NMR (DMSO‑*d*_6_): *δ* 10.89 (br s, 1H), 8.53 (s, 1H), 8.18 (s, 1H), 8.02 (s, 1H), 7.94–7.72 (m, 3H), 7.70–7.49 (m, 3H), 4.55–4.29 (m, 4H), 3.86 (s, 3H), 2.90–2.79 (m, 1H), 2.77–2.61 (m, 1H), 0.73–0.41 (m, 8H). LCMS *m/z* 379.3 [M+H].

***Methyl 4-((cyclopropyl(4-(cyclopropylcarbamoyl)benzyl)amino)methyl)benzoate (130).*** General Procedure B was followed using methyl 4-formylbenzoate (100 mg, 0.609 mmol) and **88** (168 mg, 0.731 mmol). Column chromatography eluting with 15–25 % EtOAc/hexane followed by trituration with diethyl ether and *n*-pentane (7:3) afforded compound **130** as a white solid (80 mg, 35 %). ^1^H NMR (DMSO‑*d*_6_): *δ* 8.38 (s, 1H), 7.91 (d, *J* = 8.0 Hz, 2H), 7.76 (d, *J* = 8.0 Hz, 2H), 7.41 (d, *J* = 8.0 Hz, 2H), 7.33 (d, *J* = 8.0 Hz, 2H), 3.84 (s, 3H), 3.69–3.65 (m, 4H), 2.86–2.81 (m, 1H), 1.84–1.80 (m, 1H), 0.74–0.67 (m, 2H), 0.63–0.56 (m, 2H), 0.40–0.32 (m, 2H), 0.21–0.17 (m, 2H). LCMS *m/z* 379.3 [M+H].

***Methyl 4-(1-(cyclopropylamino)ethyl)benzoate (131).*** A solution of methyl 4-acetylbenzoate (1.00 g, 5.61 mmol), cyclopropylamine (1.95 mL, 28.1 mmol), and AcOH (0.620 mL, 11.2 mmol) in toluene (25 mL) was heated to reflux for 6 h. The mixture was cooled to rt and NaBH_4_ (425 mg, 11.22 mmol) and MeOH (2.0 mL) were added and stirring was continued at rt for approximately 16 h. On completion the reaction mixture was diluted with aq. 1.0 N HCl (pH < 4) and washed with EtOAc (3 × 10 mL). The aqueous layer was neutralized with portion-wise addition of NaHCO_3_ (solid) at 0 °C and then extracted with EtOAc (3 × 10 mL). Combined organic fractions were dried over anhydrous Na_2_SO_4_ and concentrated under reduced pressure. The crude residue was triturated with diethyl ether and *n*-pentane (3:7) to afford compound **131** as an off-white solid (880 mg, 71 %). ^1^H NMR (DMSO‑*d*_6_): *δ* 7.90 (d, *J* = 8.0 Hz, 2H), 7.49 (d, *J* = 8.0 Hz, 2H), 3.85–3.74 (m, 4H), 1.83–1.75 (m, 1H), 1.22 (d, *J* = 6.6 Hz, 3H), 0.26–0.10 (m, 4H). LCMS *m/z* 220.2 [M+H].

***Methyl 4-(1-(benzyl(cyclopropyl)amino)ethyl)benzoate (132).*** To a stirred solution of **131** (450 mg, 2.05 mmol) in DMF (5 mL) at 0 °C was added K_2_CO_3_ (567 mg, 4.10 mmol) followed by benzyl bromide (0.268 mL, 2.26 mmol) and the mixture was allowed to warm to rt over 15 min and then stirred for 6 h. On completion the reaction mixture was diluted with EtOAc (10 mL), washed with cold water (2 × 5 mL) and brine (5 mL). The organic phase was dried over anhydrous Na_2_SO_4_ and concentrated under reduced pressure to afford compound **132** as a light brown gum which was used in the next step without further purification (530 mg, 83 %). ^1^H NMR (DMSO‑*d*_6_): *δ* 7.94 (d, *J* = 8.0 Hz, 2H), 7.49 (d, *J* = 8.0 Hz, 2H), 7.40–7.17 (m, 5H), 3.97–3.92 (m, 1H), 3.84 (s, 3H), 3.69 (d, *J* = 13.6 Hz, 1H), 3.38 (d, *J* = 13.6 Hz, 1H), 1.84–1.76 (m, 1H), 1.42 (d, *J* = 6.8 Hz, 3H), 0.45–0.29 (m, 2H), 0.25–0.17 (m, 1H), 0.13–0.06 (m, 1H). LCMS *m/z* 310.2 [M+H].

***4-(1-(Benzyl(cyclopropyl)amino)ethyl)benzoic acid (133).*** To a stirred solution of **132** (530 mg, 1.71 mmol) in THF (5 mL), MeOH (5 mL), and H_2_O (1 mL) was added lithium hydroxide monohydrate (360 mg, 8.58 mmol) at room temperature. The reaction mixture was then heated to 70 °C and stirred for another 3 h. On completion the reaction mixture was cooled to room temperature and concentrated under vacuo. Water (10 mL) followed by 10 % aq. Citric acid solution (10 mL) were added to the residue which was then extracted with EtOAc (4 × 10 mL). Organic fractions were combined, washed with brine (5 mL), dried over anhydrous Na_2_SO_4_ and concentrated to afford compound **133** as a white solid (506 mg, 91 %). ^1^H NMR (DMSO‑*d*_6_): *δ* 7.91 (d, *J* = 8.0 Hz, 2H), 7.46 (d, *J* = 8.0 Hz, 2H), 7.33–7.17 (m, 5H), 3.94 (q, *J* = 6.8 Hz, 1H), 3.70 (d, *J* = 13.6 Hz, 1H), 3.39 (d, *J* = 13.6 Hz, 1H), 1.81–1.76 (m, 1H), 1.42 (d, *J* = 6.8 Hz, 3H), 0.41–0.28 (m, 2H), 0.26–0.09 (m, 2H). LCMS *m/z* 296.3 [M+H].

***N-Cyclopropyl-4-(N-cyclopropylsulfamoyl)benzamide (134).*** General Procedure C was followed using 4-(chlorosulfonyl)benzoic acid (500 mg, 2.25 mmol) and cyclopropylamine (0.700 mL, 10.1 mmol). The crude residue was triturated with MeOH/DCM/*n*-pentane (1:3:6) to afford compound **134** as an off-white solid (300 g, 32 %). ^1^H NMR (DMSO‑*d*_6_): *δ* 8.66 (d, *J* = 4.0 Hz, 1H), 8.03–7.97 (m, 3H), 7.86 (d, *J* = 8.0 Hz, 2H), 2.91–2.83 (m, 1H), 2.15–2.07 (m, 1H), 0.76–0.68 (m, 2H), 0.63–0.56 (m, 2H), 0.51–0.42 (m, 2H), 0.38–0.32 (m, 2H)]. LCMS *m/z* 281.2 [M+H].

***2-(1,3-Dioxolan-*2-yl*)thiazole (135).*** To a solution of thiazole-2-carbaldehyde (1.00 g, 8.84 mmol) in toluene (50 mL) was added ethylene glycol (1.46 mL, 26.5 mmol) followed by *p*-toluenesulfonic acid (460 mg, 2.65 mmol) and the mixture was refluxed in a Dean-Stark apparatus for 16 h. On completion, the reaction mixture was cooled to room temperature and concentrated under reduced pressure. Water (25 mL) was added to the residue, and the aqueous layer extracted by EtOAc (3 × 10 mL). Combined organic fractions were dried over Na_2_SO_4_ and concentrated under reduced pressure. Column chromatography eluting with 10–15 % EtOAc/hexane afforded compound **135** as colourless oil (870 mg, 63 %). ^1^H NMR (CDCl_3_): *δ* 7.81 (d, *J* = 3.2 Hz, 1H), 7.36 (d, *J* = 3.2 Hz, 1H), 6.15 (s, 1H), 4.29–3.97 (m, 4H).

***2-(1,3-Dioxolan-*2-yl*)thiazole-5-carboxylic acid (136).*** To a stirred solution of **135** (500 mg, 3.18 mmol) in anhydrous THF (10 mL), was added a solution of 2.5 M *n*-BuLi in hexane (3.82 mL, 9.54 mmol) under N_2_ at −78 °C and the mixture was stirred for 1 h. The mixture was then added dropwise into a separate vessel containing dry ice under N_2_ and the resulting mixture was allowed to warm slowly to room temperature and stirred overnight. The reaction mixture was quenched by slow addition of aq. 2 N HCl (pH 4) and then extracted with diethyl ether (3 × 10 mL). Combined organic fractions were dried over Na_2_SO_4_ and concentrated under reduced pressure. The crude residue was triturated with *n*-pentane to afford compound **136** as white solid (397 mg, 62 %). ^1^H NMR (DMSO‑*d*_6_): *δ* 13.68 (brs, 1H), 8.35 (s, 1H), 6.09 (s, 1H), 4.11–4.00 (m, 4H). LCMS *m/z* 202.1 [M+H].

***N-Cyclopropyl-2-(1,3-dioxolan-*2-yl*)thiazole-5-carboxamide (137).*** General Procedure C was followed using **136** (250 mg, 1.24 mmol) and cyclopropylamine (0.130 mL, 1.86 mmol) The crude residue was triturated with MeOH/DCM/*n*-pentane (1:4:6) to afford compound **137** as an off-white solid which was used in the next step without further purification (222 mg, 74 %). ^1^H NMR (DMSO‑*d*_6_): *δ* 8.71 (brs, 1H), 8.30 (s, 1H), 6.04 (s, 1H), 4.13–3.98 (m, 4H), 2.83–2.75 (m, 1H), 0.75–0.66 (m, 2H), 0.64–0.48 (m, 2H). LCMS *m/z* 240.9 [M+H].

***N-Cyclopropyl-2-formylthiazole-5-carboxamide (138).*** To a stirred solution of **137** (200 mg, 0.832 mmol) in acetone and water (8 mL, 3:1), was added *p*-toluenesulfonic acid (158 mg, 0.832 mmol) and the mixture was refluxed for 4 h. On completion, the reaction mixture was concentrated under reduced pressure and the residue was redissolved in EtOAc (20 mL), washed with sat. aq. NaHCO_3_ (10 mL), water (10 mL) and brine (5 mL). The organic fraction was dried over Na_2_SO_4_ and concentrated under reduced pressure to afford compound **138** as a colourless semi-solid which was used in the next step without further purification (138 mg, 85 %). ^1^H NMR (DMSO‑*d*_6_): *δ* 9.92 (s, 1H), 8.96 (brs, 1H), 8.64 (s, 1H), 2.87–2.60 (m, 1H), 0.79–0.66 (m, 2H), 0.65–0.49 (m, 2H). LCMS *m/z* 197.2 [M+H].

***N-Cyclopropyl-2-((cyclopropylamino)methyl)thiazole-5-carboxamide (139).*** To a stirred solution of **138** (100 mg, 0.510 mmol) in DCE (5 mL) were added cyclopropylamine (0.070 mL, 1.0 mmol) and AcOH (0.060 mL, 1.0 mmol) and the mixture was stirred at rt for 3 h NaBH_4_ (58 mg, 1.5 mmol) was then added followed by MeOH (0.1 ml) and stirring was continued at rt for approximately 16 h. On completion, aq. 1 N HCl (5 mL) and DCM (5 mL) were added to the reaction mixture which was transferred to a separatory funnel. The organic fraction was discarded, and the aqueous fraction was neutralized by sat. aq. NaHCO_3_ and extracted with EtOAc (3 × 10 mL). Combined organic fractions were washed with brine (5 mL), dried over anhydrous Na_2_SO_4_ and concentrated under reduced pressure. The crude residue was triturated with diethyl ether/*n*-pentane (3:7) to afford compound **139** as an off-white solid (40 mg, 33 %). ^1^H NMR (DMSO‑*d*_6_): *δ* 8.54 (d, *J* = 3.2 Hz, 1H), 8.16 (s, 1H), 3.98 (s, 2H), 2.82–2.73 (m, 1H), 2.23–2.15 (m, 1H), 0.74–0.65 (m, 2H), 0.58–0.52 (m, 2H), 0.41–0.35 (m, 2H), 0.32–0.26 (m, 2H). LCMS *m/z* 238.1 [M+H].

***2-(Ethoxycarbonyl)thiazole-5-carboxylic acid (140).*** To a solution of ethyl 2-amino-2-thioxoacetate (1.00 g, 8.84 mmol) in 1,4-dioxane (50 mL) was added 3-bromo-2-oxopropanoic acid (1.46 mL, 26.5 mmol) and the mixture was refluxed for 5 h. The reaction mixture was then cooled to rt and poured into water (50 mL). The solution was basified with sat. aq. NaHCO_3_ (pH > 9) and washed with EtOAc (3 × 10 mL). The aqueous fraction was separated, acidified with aq. 2 N HCl (pH < 4) and extracted with EtOAc (3 × 10 mL). Combined organic fractions were dried over Na_2_SO_4_ and concentrated under reduced pressure to afford compound **140** which was used in the next step without further purification (720 mg, 95 %). ^1^H NMR (DMSO‑*d*_6_): *δ* 8.77 (s, 1H), 4.10 (q, *J* = 7.0 Hz, 2H), 1.34 (t, *J* = 7.0 Hz, 3H). LCMS *m/z* 202.0 [M+H].

***Ethyl 5-(hydroxymethyl)thiazole-2-carboxylate (141).*** To a stirred solution of **140** (800 mg, 3.98 mmol) in THF (10 mL) at 0 °C was added BH_3_-DMS (1.13 mL, 11.9 mmol) dropwise and the mixture was allowed to warm to rt slowly while stirring was continued for approximately 16 h. Upon completion the mixture was quenched with a few drops of MeOH and aq. 1 N HCl before being extracted with EtOAc (3 × 10 mL). Combined organic fractions were dried over Na_2_SO_4_ and concentrated under reduced pressure to afford compound **141** as light-yellow oil which was used in the next step without further purification (260 mg, 35 %). LCMS *m/z* 187.9 [M+H].

***Ethyl 5-formylthiazole-2-carboxylate (142).*** To a solution of **141** (260 mg, 1.39 mmol) in DCM (10 mL) was added Dess-Martin periodinane (707 mg, 1.67 mmol) at 0 °C. The mixture was allowed to warm to rt over 15 min and stirring was continued for another 4 h. On completion, the reaction mixture was quenched by sat. aq. NaHCO_3_ and the resulting precipitate was removed by filtration. The filtrate was concentrated under reduced pressure to compound **142** (also containing periodinane impurities) which was used for the next step without further purification (240 mg, 93 %). ^1^H NMR (DMSO‑*d*_6_): *δ* 10.02 (s, 1H), 8.99 (s, 1H), 4.42 (q, *J* = 7.0 Hz, 2H), 1.33 (t, *J* = 7.0 Hz, 3H). LCMS *m/z* 186.2 [M+H].

***Ethyl 5-((benzyl(cyclopropyl)amino)methyl)thiazole-2-carboxylate (143).*** General Procedure B was followed using **142** (300 mg, 1.62 mmol) and *N*-benzylcyclopropanamine (238 mg, 1.62 mmol). Column chromatography eluting with 15–30 % EtOAc/hexane afforded compound as **143** an off-white gum (201 mg, 39 %) which was 70 % pure by LCMS and used without further purification. LCMS *m/z* 317.1 [M+H].

***Methyl 5-((benzyl(cyclopropyl)amino)methyl)picolinate (144). General Procedure I:*** To a stirred solution of methyl 5-formylpyridine-2-carboxylate (200 mg, 1.21 mmol) in MeOH (10 mL) was added *N*-benzylcyclopropanamine (267 mg, 1.82 mmol) and the solution cooled to 0 °C. NaBH(OAc)_3_ (642 mg, 3.03 mmol) was then added portion-wise and the mixture was allowed to warm to rt and stirred for 18 h. LCMS revealed incomplete conversion of aldehyde to the desired product and additional NaBH(OAc)_3_ was added (770 mg, 3.64 mmol). The mixture was stirred at rt for an additional 16 h at which point complete conversion was observed. Water (20 mL) was added, and the mixture was extracted with DCM (3 × 10 mL). Organic fractions were combined, washed with brine (5 mL), dried over MgSO_4_, filtered and concentrated. The resulting crude material was purified by column chromatography eluting with 0–50 % EtOAc/heptane to afford compound **144** as a colourless oil (78 mg, 22 %). ^1^H NMR (CDCl_3_) *δ* 8.59 (d, *J* = 2.1 Hz, 1H), 8.04 (dd, *J* = 8.0, 0.8 Hz, 1H), 7.70 (dd, *J* = 7.9, 2.1 Hz, 1H), 7.35–7.17 (m, 5H), 3.99 (s, 3H), 3.70 (d, *J* = 3.6 Hz, 4H), 1.83 (tt, *J* = 6.5, 3.7 Hz, 1H), 0.48–0.32 (m, 2H), 0.27 (p, *J* = 4.1 Hz, 2H). LCMS *m/z* 207.2 [M+H].

***Methyl 6-((benzyl(cyclopropyl)amino)methyl)nicotinate (145).*** General Procedure I was followed using methyl 6-formylnicotinate (200 mg, 1.21 mmol, 1.0 equiv). Column chromatography eluting with 0–10 % EtOAc/DCM afforded compound **145** as a yellow oil (130 mg, 36 %). ^1^H NMR (CDCl_3_) *δ* 9.13–9.06 (m, 1H), 8.17 (dd, *J* = 8.1, 2.2 Hz, 1H), 7.41 (d, *J* = 8.1 Hz, 1H), 7.30–7.17 (m, 5H), 3.92 (s, 3H), 3.87 (s, 2H), 3.74 (s, 2H), 1.93 (s, 1H), 0.43–0.35 (m, 2H), 0.28 (s, 2H). LCMS *m/z* 207.2 [M+H].

***Methyl 4-(cyclopropylcarbamoyl)benzoate (146).*** 4-Methoxycarbonylbenzoic acid (250 mg, 1.39 mmol), cyclopropanamine (0.240 mL, 3.47 mmol) and HATU (630 mg, 1.7 mmol) were dissolved in DCM (25 mL) and stirred at 20 °C for 2 h. The reaction was then washed with 2 M HCl (15 mL), saturated NaHCO_3_ (15 mL) and brine (10 mL). The organics were separated and dried with anhydrous Na_2_SO_4_, filtered and concentrated to obtain compound **146** as an off-white solid (250 mg, 81 %). ^1^H NMR (CDCl_3_): *δ* 8.04–8.16 (m, 2H), 7.75–7.85 (m, 2H), 6.26 (s, 1H), 3.95 (s, 3H), 2.85–3.00 (m, 1H), 0.80–0.96 (m, 2H), 0.60–0.74 (m, 2H).

***4-(Cyclopropylcarbamoyl)benzoic acid (147)***. Compound **146** (246 mg, 1.12 mmol) and LiOH (130 mg, 5.6 mmol) were dissolved in a 1:1 solution of MeOH and H_2_O (10 mL) and stirred at 50 °C for 4 h. The reaction was then concentrated to remove MeOH and the solution made acidic (pH 4) with aq. 1 M HCl. The aqueous mixture was then extracted with EtOAc (3 × 10 mL). The organics were separated and dried with anhydrous Na_2_SO_4_, filtered and concentrated to obtain compound **147** as a white solid (157 mg, 68 %). ^1^H NMR (DMSO‑*d*_6_) *δ* 8.59 (d, *J* = 4.3 Hz, 1H), 7.95–8.02 (m, 2H), 7.86–7.93 (m, 2H), 2.86 (dt, *J* = 4.0, 7.3 Hz, 1H), 0.67–0.75 (m, 2H), 0.53–0.63 (m, 2H).

***P. falciparum* 3D7 Asexual Stage LDH Assay.** This is a platform assay is routinely undertaken at the Walter and Eliza Hall Institute and details on the protocol has been previously published [8,49]. Briefly, 10-point dilution series of the compounds were prepared in 384 well assay plates using an Echo555 (Labcyte). Appropriate volumes of 10 mM compound stocks were transferred into the assay plates such that the starting concentration was 1 μM, with a 1 in 3-fold dilution series. All wells were backfilled with DMSO to 40 nL such that this remained constant (0.1 % DMSO). The positive growth control was 0.1 % DMSO and the negative growth control was 100 nM chloroquine. The parasite inoculum (40 μL) was dispensed into plates containing compounds using a Multidrop Combi dispenser (Thermo Scientific). Plates were incubated at 37 °C for 72 h in an atmosphere of 5 % CO_2_, 5 % O_2_, 95 % N_2_. Following 72 h growth, plates were sealed with parafilm and frozen at −80 °C overnight. Plates were thawed at room temperature for at least 4 h prior to LDH activity being measured. 45 μL of fresh LDH reaction mix (174 mM sodium l-lactate, 214 μM 3-acetyl pyridine adenine dinucleotide (APAD), 270 μM Nitro Blue tetrazolium chloride (NBT), 4.35 U/mL diaphorase, 0.7 % Tween 20, 100 mM Tris-HCl pH 7.5) was dispensed into each well using a Multidrop Combi dispenser. Plates were shaken to ensure mixing and absorbance at 650 nm was measured in an EnVision (PerkinElmer) plate reader after 30 min of incubation at room temperature. EC_50_ values were calculated by Dotmatics 5.3 and Spotfire 7.11.1 software using a nonlinear regression four-parameter fit analysis. The equation used is sigmoidal dose response (variable slope), Y = bottom + (top − bottom)/(1 + 10((logEC_50_ − X) × Hill Slope)).

**Human HepG2 Growth Inhibition Assay.** This is a routine assay undertaken at the Walter and Eliza Hall Institute and the method has been previously published [8,50]. HepG2 cells were cultured in Dulbeccos modified eagles medium (DMEM) supplemented with 10 % fetal calf serum (FCS), in a humidified incubator at 37 °C and 5 % CO_2_. Assay plates (Greiner #781098, 384 well, white, tissue culture treated) were prepared by transferring appropriate volumes of 10 mM compound stocks using an Echo555 (Labcyte) such that the starting concentration was 50 μM, with a 1:2-fold dilution series. All wells were backfilled with DMSO to 100 nL such that this remained constant (0.5 % DMSO). The positive growth control was 0.5 % DMSO and the negative growth control was 10 μM Bortezomib. HepG2 cells were seeded with 1000 cells in 50 μL DMEM with 10 % FCS into each well of the assay plates using a Multidrop Combi dispenser (Thermo Scientific). Plates were incubated at 37 °C and 5 % CO_2_ for 48 h. Cytotoxicity was determined using Cell Titer Glo (Promega) and calculated as a percentage using DMSO as the positive growth control and 10 μM Bortezomib as a negative growth control. EC_50_ values were calculated by Dotmatics 5.3 and Spotfire 7.11.1 software using a nonlinear regression four-parameter fit analysis. The equation used is sigmoidal dose response (variable slope), Y = bottom + (top − bottom)/(1 + 10((logEC_50_ − X) × Hill Slope)).

***In vitro* ADME.** These routine assays are conducted by TCG Life Sciences and methods are previously disclosed [8,51] and briefly outlined below.

**Human Liver Microsome Stability.** A solution of the test compounds in phosphate buffer solution (1 μM) was incubated in pooled human liver microsomes (0.5 mg/mL) for 0, 5, 20, 30, 45 and 60 min at 37 °C in the presence and absence of NADPH regeneration system. The reaction was terminated with the addition of ice-cold acetonitrile containing system suitability standard at designated time points. The sample was centrifuged (4200 rpm) for 20 min at 20 °C and the supernatant was half diluted in water and then analyzed by means of LC-MS/MS.% Parent compound remaining, half-life (T_1/2_) and clearance (CL_int_) were calculated using standard methodology. The experiment was carried out in duplicate. Verapamil, diltiazem, phenacetin, and imipramine were used as reference standards.

**Rat Hepatocyte Stability.** A solution of the test compound in Krebs-Henseliet buffer solution (1 μM) was incubated in pooled rat hepatocytes (1 × 10^6^ cells/mL) for 0, 15,30,45, 60, 75 and 90 min at 37 °C (5 % CO_2_, 95 % relative humidity). The reaction was terminated with the addition of ice-cold acetonitrile containing system suitability standard at designated time points. The sample was centrifuged (4200 rpm) for 20 min at 20 °C and the supernatant was half diluted in water and then analyzed by means of LC-MS/MS. % Parent compound remaining, half-life (T_1/2_) and clearance (CL_int_) were calculated using standard methodology. The experiment was carried out in duplicate. Diltiazem, 7-ethoxy coumarin, propranolol, and midazolam were used as reference standards.

**Solubility.** The solubility assay was performed using a miniaturized shake flask method. A solution of phosphate buffered saline (PBS) and the test compound (200 μM) was incubated at 25 °C with constant shaking (600 rpm) for 2 h. The samples were filtered using a multiscreen solubility filter plate. The filtrate was half diluted in acetonitrile. A five-point linearity curve was prepared in PBS:acetonitrile (1:1, v/v) at 200, 150, 75, 25 and 2.5 μM. Blank, linearity and test samples (n = 2) were transferred to a UV readable plate and the plate was scanned for absorbance. Best fit calibration curves were constructed using the calibration standards and used to determine the test sample solubility. The experiment was carried out in duplicate. Diethylstilbestrol, haloperidol, and diclofenac sodium were used as reference standards.

**eLogD.** The LogD (pH 7.4) assay was performed using a miniaturized shake flask method. A solution of a pre-saturated mixture of 1-octanol and phosphate buffered saline (PBS) (1:1, v/v) and the test compound (75 μM) was incubated at 25 °C with constant shaking (850 rpm) for 2 h. After incubation the organic and aqueous phases were separated, and samples of each phase transferred to plate for dilution. The organic phase was diluted to 1000-fold and the aqueous phase was diluted 20-fold. The samples were quantitated using LC-MS/MS. The experiment was carried out in duplicate. Propranolol, amitriptyline, and midazolam were used as reference standards.

**Resistance Selection.** This was performed as previously described [8], with 3D7 wildtype parasites exposed to 2 x EC_50_ of W466 (**1**) (0.5 μM) or W499 (**2**) (0.26 μM) for first round of selection.

**Whole Genome Sequencing of W466- and W499- resistant strains.** Methods for whole genome sequencing were followed according to prior literature [8]. The data for this study have been deposited in the European Nucleotide Archive (ENA) at EMBL-EBI under accession number PRJEB75780 (https://www.ebi.ac.uk/ena/browser/view/PRJEB75780). The sequencing for the parental 3D7 sample has accession ERS14309311.

**MIR assay.***Plasmodium falciparum* asexual blood stage (ABS) parasites were cultured at 3 % hematocrit in human O^+^ RBCs in RPMI-1640 media, supplemented with 25 mM HEPES, 50 mg/L hypoxanthine, 2 mM l-glutamine, 0.21 % sodium bicarbonate, 0.5 % (wt/vol) AlbuMAXII (Invitrogen) and 10 μg/mL gentamycin, in modular incubator chambers (Billups- Rothenberg) at 5 % O_2_, 5 % CO_2_ and 90 % N_2_ at 37 °C. Dd2 parasites were obtained from T. Wellems (NIAID, NIH). Dd2-B2 is a genetically homogenous line that was cloned from Dd2 by limiting dilution in the Fidock lab. Dd2-Polδ is a line edited in the Fidock Lab to have a hypermutable polymerase [34]. To define the EC_50_ of ABS parasites, we exposed Dd2-B2 and Dd2-Polδ ring-stage cultures at 0.3 % parasitemia and 1 % hematocrit for 72 h to a range of ten drug concentrations that were 2-fold serially diluted in duplicates along with drug-free controls. Parasite survival was assessed by flow cytometry on an iQue flow cytometer (Sartorius) using SYBR Green and MitoTracker Deep Red FM (Life Technologies) as nuclear stain and vital dyes respectively.

For W499 (**2**), one single-step selection was set up using 2 × 10^5^ Dd2-B2 parasites in each well of a 96 well plate at a starting concentration of 3 × EC_90_ (671 nM). We observed parasite clearance over the first 4 days. This selection had a consistent drug pressure of 3 × EC_90_ over 60 days, and cultures were screened three times weekly by flow cytometry and smearing. Wells are considered positive for recrudescence when the overall parasitemia reaches 0.3 % and parasites are seen on a blood smear. No recrudescence was observed over the course of this selection.

For W466 (**1**), one single-step selection was set up using 2 × 10^5^ Dd2-B2 parasites in each well of a 96-well plate at a starting concentration of 3 × EC_90_ (2134 nM). We observed parasite clearance over the first 4 days. This selection had a consistent drug pressure of 3 × IC_90_ for up to 60 days, and cultures were screened three times weekly by flow cytometry and smearing. Wells are considered positive for recrudescence when the overall parasitemia reaches 0.3 % and parasites are seen on a blood smear. Well H3 became positive on day 30 and was picked for MIR determination and for IC_50_ and IC_90_ values as described above.

W466 (**1**) was also further investigated using an additional selection set-up using two flasks with 3.3 × 10^8^ Dd2-B2 parasites each, and one flask with 3.3 × 10^8^ Dd2-Polδ parasites, again at a starting concentration of 3 × EC_90_ (2134 nM). We observed parasite clearance over the first 5 days. Recrudescence was only observed in the flask containing the Dd2-Polδ parasites (F3). The F3 bulk culture was then cloned under 3 × EC_90_ drug pressure using a limiting dilution protocol at a concentration of 0.5 % infected RBCs/well in a 96-well plate. Three F3 clones were selected for IC_50_ and IC_90_ determination as described above and genotyping.

**Whole Genome Sequencing for MIR recrudescent populations.** Whole-genome sequencing was performed using an Illumina DNA prep kit with Nextera DNA CD Indexes (Illumina, San Diego, CA) and multiplexed on a MiSeq (MiSeq Reagent Kit V3 600, Illumina, San Diego, CA) to generate 300 bp paired-end reads. Sequences were aligned to the Pf 3D7 reference genome (PlasmoDB-48_Pfalciparum3D7; https://plasmodb.org/plasmo/app/downloads/release-48/Pfalciparum3D7/fasta/) using the Burrows-Wheeler Alignment (BWA version 0.7.17). PCR duplicates and unmapped reads were filtered using Samtools (version 1.13) and Picard MarkDuplicates (GATK version 4.2.2). Base quality scores were recalibrated using the GATK BaseRecalibrator (GATK version 4.2.2). The GATK HaplotypeCaller (GATK version 4.2.2) was used to identify all possible single-nucleotide variants (SNVs) in test parasite lines filtered based on quality scores (variant quality as a function of depth QD > 1.5, mapping quality >40, min base quality score >18, read depth >5) to obtain high-quality single-nucleotide polymorphisms (SNPs) that were annotated using SnpEff version 4.3t. BIC-Seq version 1.1.2 was used to discover copy number variants (CNVs) against the parental strain using the Bayesian statistical model. SNPs and CNVs were visually inspected and confirmed using the Integrative Genome Viewer (IGV). The data for this study have been deposited in the European Nucleotide Archive (ENA) at EMBL-EBI under accession number PRJEB75806 (https://www.ebi.ac.uk/ena/browser/view/PRJEB75806).

**Model of *P. falciparum* cyt *b*.** The homology model of *P. falciparum* cyt *b* was created as previously described [8]. Briefly, SWISS-MODEL software [52] was used to identify *Gallus gallus* cyt *bc*1 (PDB: 3H1I) [28] as the highest homologous X-ray structure based on the protein sequence of *P. falciparum* cyt *b*. The pdb for the model of *P. falciparum* cyt *b* was transformed and visualized using PyMol Molecular Graphics System software (version 2.5.0). ATQ and ELQ300 were positioned in the *P. falciparum* cyt *b homology* model relative to the X-ray structures previously discolsed [22,32].

**Cyt *b* Resistant and ScDHODH Asexual Stage Assays.** These assays were conducted as previously described [8]. Briefly, asexual parasite strains indicated in Table 12 were incubated with compound in a dose response format for 72 h. Parasitemia was determined using SYBR-green fluorescence measured by flow cytometry (Thermo Fisher Attune). EC_50_ values were determined as described for the LDH assay.

**Parasite Reduction Ratio Assay.** This is a platform assay conducted by TGC Lifesciences using a previously described protocol [38]. 3D7 *P. falciparum* strain was treated with compounds W466 (**1**) and W499 (**2**) separately at a concentration corresponding to 10 × IC_50_ (IC_50_ was previously determined using *in vitro* *P.* *falciparum* LDH assay after 72 h of drug treatment). Parasites were treated up to 120 h. Drug was renewed every 24 h over the entire treatment period (as directed). Samples of parasites were taken from the treated culture every 24 h (24, 48, 72, 96 and 120 h time points), drug was washed out and drug-free parasites were cultured in 96 well plates. To quantify number of viable parasites after treatment, serial dilutions were performed with the above-mentioned compounds after washing out the compound. Parasites were cultured in microtiter plates to allow all wells with viable parasites, to render detectable parasitaemia. After 22 days of culturing, cultured plates were checked for parasite growth. Final sampling was done after 28 days to confirm growth/no growth. The parasite levels were determined using a LDH assay as follows. A culture of parasitized red blood cells (RBC) of strain 3D7 with 0.25 % parasitaemia and 2 % haematocrit in RPMI-1640, 0.5 % albumax and 150 μM hypoxanthine is exposed to 3-fold serial dilutions of the compound. Plates are incubated for 72 h at 37 °C, 5 % CO_2_, 5 % O_2_, 90 % N_2_. After 72 h incubation, plates were frozen at −80 °C for 24 h and then thawed at 21 °C for 5 h. To evaluate Pf-LDH activity, 70 μL of freshly prepared reaction mix containing: 143 mM sodium l-lactate, 143 μM 3-acetyl pyridine adenine dinucleotide (APAD), 179 μM nitro blue tetrazolium chloride (NBT), diaphorase (2.83 U/mL), 0.7 % Tween 20, 100 mM Tris-HCl pH 8.0 was added into each well of the incubation plate mentioned above. Plates were shaken to ensure mixing. Plates are placed in an incubator at 21 °C for 20 min. Once a colour reaction is observed from yellow to purple, absorbance at 650 nm was recorded in a plate reader (Spectramax M5) after 20 min of incubation.

**Asexual Stage of Arrest Assay.** This assay was conducted as previously described [8].

**ETC Biosensor Assay.** This assay was performed as previously described [17]. Briefly, 3 **×** 10^8^ trophozoite infected RBCs were purified using the SuperMACS II separator (Miltenyi Biotec, Germany). The trophozoite-stage parasites were released from erythrocytes by treating them with 0.05 % (w/v) pre-warmed saponin in phosphate-buffered saline (PBS) at 37 °C for 3 min. The cells were washed with pre-warmed PBS, three times. The parasite count was adjusted to 5 × 10^7^ parasites/mL in mitochondrial assay solution (MAS) buffer (mannitol (220 mM), sucrose (70 mM), KH_2_PO_4_ (10 mM), MgCl_2_ (5 mM), HEPES (2 mM), EGTA (1 mM), BSA 0.2 % (w/v)), supplemented with 25 mM malate and 0.002 % (w/v) digitonin. Parasites were seeded at a density of 5 × 10^6^ cells per well in a Cell-Tak-coated XFe96 cell culture plate and centrifuged (800×*g*, 10 min, room temperature) with low braking to adhere the parasites to the bottom of the wells. 75 μL MAS buffer was added to the wells, without detaching the parasite. The plate was visualized under the microscope to ensure cell monolayer. The inhibitor/controls were prepared in MAS buffer (final concentration after injection given in brackets) and loaded into ports A-C of the XFe96 sensor cartridge: Port A, inhibitors/atovaquone (5 μM); Port B, N,N,N',N'-tetramethyl-*p*-phenylenediamine dihydrochloride (TMPD - 0.2 mM) and ascorbic acid (2 mM); Port C, sodium azide (NaN_3_; 10 mM). The cells were measured for oxygen consumption rate (OCR) using Seahorse XFe96 flux analyzer (Agilent). The OCR was assessed for five cycles of 20 s mixing, 1 min waiting, 2.5 min measuring to establish baseline OCR before any substrate injections, for eight cycles of 20 s mixing, 1 min waiting followed by 2.5 min measuring after inhibitor injection from port A and for five cycles of 20 s mixing 1 min waiting, 2.5 min measuring after TMPD, and NaN_3_, injections.

**Exoerythrocytic Stage Assay.** This is a platform assay conducted at the University of California, San Diego and follows a protocol previously described [42]. *P. berghei* luciferase sporozoites were obtained by dissection of infected *A. stephensi* mosquito salivary glands supplied by the New York University Insectary. Dissected salivary glands were homogenized in a glass tissue grinder and filtered twice through a 20 μm nylon net filter (Steriflip, Millipore) and counted using a hemocytometer. The sporozoites were kept on ice until needed. Host HepG2-A16-CD81EGFP cells [53] were stably transformed to express a GFP-CD81 fusion protein [54], were cultured at 37 °C in 5 % CO_2_ in DMEM (Invitrogen, Carlsbad, USA) supplemented with 10 % FBS, 0.29 mg/mL glutamine, 100 units penicillin and 100 μg/mL streptomycin. For the sporozoite invasion assay the rodent parasite, *P. berghei* was used. *P. berghei* infects human hepatocarcinoma HepG2 cells expressing the tetraspanin CD81 receptor. 3 × 10^3^ HepG2-A16-CD81EGFP cells in 5 μL of medium (2 × 10^5^ cells/mL, 5 % FBS, 5 × Pen/Strep/Glu) were seeded in 1536-well plates (Greiner BioOne white solid bottom custom GNF mold) 20–26 h prior to the actual infection. 18 h prior to infection, 50 nL of compound in DMSO (0.5 % final DMSO concentration per well) were transferred using the Acoustic Transfer System (ATS) (Biosero) into the assay plates (10 μM final concentration). In cases when acoustic compatible source plates were not available, the 50 nL of compounds to test were transferred with PinTool (GNF Systems). Atovaquone (5 μM) and 0.5 % DMSO were used as positive and negative controls, respectively. *P. berghei* luciferase sporozoites were freshly dissected from infected *A. stephensi* mosquito salivary glands and filtered twice through a through a 20 μm nylon net filter (Steriflip, Millipore). The sporozoites were re-suspended in media, counted in a hemocytometer and their final concentration adjusted to 200 sporozoites per μL. Also, penicillin and streptomycin were added at 5-fold increased concentration for a final 5-fold increased concentration in the well. The HepG2-A16-CD81EGFP cells were then infected with 1 × 10^3^ sporozoites per well (5 μL) with a single tip Bottle Valve liquid handler (WDII, GNF), and the plates spun down at 37 °C for 3 min in an Eppendorf 5810 R centrifuge with a centrifugal force of 330 g on normal acceleration and brake setting. After incubation at 37 °C for 48 h the EEF growth was quantified by a bioluminescence measurement. Atovaquone and naïve wells were used as controls on each plate. The compounds were screened in a 12-point serial dilution to determine their exact EC_50_ values. For bioluminescence quantification of exo-erythrocytic forms (EEFs), the media was removed by spinning the inverted plates at 150 g for 30 sec. 2 μL BrightGlo (Promega) were being dispensed with the MicroFlo (BioTek) liquid handler. Immediately after addition of the luminescence reagent, the plates were read by the Envision Multilabel Reader (PerkinElmer). EC_50_ values were obtained using measured bioluminescence intensity and a non-linear variable slope four parameter regression curve fitting model in Prism 6 (GraphPad Software Inc).

**Dual Gamete Formation Assay.** Compounds were evaluated as previously described [41,55] as a platform run at both Imperial College London and the London School of Hygiene and Tropical Medicine. Briefly, mature stage V gametocytes were exposed to compounds for 48 h at 37 °C in 384 well plates in gametocyte culture medium (RPMI 1640 supplemented with 25 mM HEPES, 50 μg/mL hypoxanthine, 4.8 g/L NaHCO_3_, 2 mM l-glutamine, 5 % pooled type AB serum, 0.5 % Albumax II (Gibco)) under a 1 % O_2_/3 % CO_2_/96 % N_2_ environment. Gametogenesis was then triggered by the addition of 10 μL ookinete medium (gametocyte culture medium supplemented with 100 μM xanthurenic acid and 0.27 μg/mL Cy3-labelled anti-Pfs25 antibody) to each well at room temperature. Plates were then cooled on a metal block at 4 °C for 4 min to ensure even cooling and then stabilized for a further 4 min at 28 °C. At 20 min post-induction, male gametogenesis was recorded in each well by automated brightfield microscopy using a × 4 objective lens and 1.5 × magnifier ( × 6 effective magnification). Afterward, plates were incubated in the dark at room temperature for 24 h and then female gametogenesis recorded in each well by automated fluorescence microscopy (anti-Pfs25-positive cells). All experiments were performed in quadruplicate with DMSO and cabamiquine as negative and positive controls respectively. All data was evaluated in comparison to the positive and negative controls to calculate percentage inhibition of male and female gametocytes, and dose response analysis and EC_50_ calculation performed using GraphPad Prism.

**Animal Ethics and Maintenance.** Ethics statement: Experiments involving rodents were performed in strict accordance with the recommendations of the Australian Government and National Health and Medical Research Council Australian code of practice for the care and use of animals for scientific purposes. The protocols were approved by the Animal Welfare Committee at Deakin University (approval no. G11/2023).

***P. berghei* 4-day Mouse Model.** This model was conducted as previously described [14].

## CRediT authorship contribution statement

**Jon Kyle Awalt:** Writing – review & editing, Writing – original draft, Methodology, Investigation, Data curation. **Wenyin Su:** Writing – original draft, Methodology, Investigation, Data curation. **William Nguyen:** Supervision, Methodology, Data curation. **Katie Loi:** Methodology, Investigation, Data curation. **Kate E. Jarman:** Methodology, Formal analysis, Data curation. **Jocelyn S. Penington:** Methodology, Investigation, Formal analysis, Data curation. **Saishyam Ramesh:** Methodology, Investigation, Formal analysis, Data curation. **Kate J. Fairhurst:** Methodology, Investigation, Formal analysis, Data curation. **Tomas Yeo:** Methodology, Formal analysis, Data curation. **Heekuk Park:** Methodology, Investigation, Formal analysis, Data curation. **Anne-Catrin Uhlemann:** Methodology, Investigation, Formal analysis, Data curation. **Bikash Chandra Maity:** Methodology, Data curation. **Nirupam De:** Methodology, Formal analysis, Data curation. **Partha Mukherjee:** Methodology, Formal analysis, Data curation. **Arnish Chakraborty:** Methodology, Formal analysis, Data curation. **Alisje Churchyard:** Methodology, Formal analysis, Data curation. **Mufuliat T. Famodimu:** Methodology, Formal analysis, Data curation. **Michael J. Delves:** Supervision, Funding acquisition, Formal analysis, Data curation. **Jake Baum:** Supervision, Funding acquisition, Formal analysis, Data curation. **Nimisha Mittal:** Methodology, Formal analysis, Data curation. **Elizabeth A. Winzeler:** Supervision, Funding acquisition, Formal analysis, Data curation. **Anthony T. Papenfuss:** Supervision, Funding acquisition, Formal analysis. **Mrittika Chowdury:** Methodology, Investigation, Data curation. **Tania F. de Koning-Ward:** Supervision, Funding acquisition, Formal analysis. **Alexander G. Maier:** Writing – review & editing, Supervision, Funding acquisition, Formal analysis. **Giel G. van Dooren:** Supervision, Funding acquisition, Formal analysis. **Delphine Baud:** Supervision, Resources, Formal analysis. **Stephen Brand:** Supervision, Resources, Formal analysis. **David A. Fidock:** Supervision, Funding acquisition, Formal analysis. **Paul F. Jackson:** Supervision, Project administration, Funding acquisition, Conceptualization. **Alan F. Cowman:** Supervision, Funding acquisition, Formal analysis. **Madeline G. Dans:** Writing – review & editing, Writing – original draft, Supervision, Methodology, Investigation, Formal analysis, Data curation. **Brad E. Sleebs:** Writing – review & editing, Writing – original draft, Supervision, Project administration, Investigation, Funding acquisition, Formal analysis, Data curation.

## Declaration of competing interest

The authors declare that they have no known competing financial interests or personal relationships that could have appeared to influence the work reported in this paper.

## Data Availability

Data will be made available on request.

## Abbreviations

ACT, artemisinin combination therapy; ART, artesunate; ATQ, atovaquone; CoQ, coenzyme Q; CQ, chloroquine; cyt *b*, cytochrome *b*; cyt *bc*1, cytochrome *b* complex 1; DCM, dichloromethane; DGFA, dual gamete formation assay; DHODH, dihydroorotate dehydrogenase; DIPEA, *N*,*N*-diisopropylethylamine; DMF, dimethylformamide; DMSO, dimethyl sulfoxide; EDCI, 1-ethyl-3-(3-dimethylaminopropyl)carbodiimide hydrochloride; EEF, exoerythrocytic form; ETC, electron transport chain; GFP, green fluorescent protein; HATU, 1-[Bis(dimethylamino)methylene]-1H-1,2,3-triazolo[4,5-*b*]pyridinium 3-oxide hexafluorophosphate; HOBt, hydroxybenzotriazole; HRMS, high resolution mass spectrometry; HT, high throughput; LCMS, liquid chromatography mass spectrometry; LDH, lactate dehydrogenase; MIR, minimum inoculum of resistance; NDH, NADH dehydrogenase; NMR, nuclear magnetic resonance; OCR, oxygen consumption rate; Pb, *Plasmodium berghei*; Pf, *Plasmodium falciparum*; PI4K, phosphatidylinositol 4-kinase; PRR, parasite reduction ratio; SAR, structure activity relationship, Sc, *Saccharomyces cerevisiae*; SNP, single-nucleotide polymorphism; SNV, single nucleotide variant; TCP, target candidate profile; TMPD, *N,N,N′,N*′-tetramethyl-*p*-phenylenediamine dihydrochloride; WJM, WEHI Janssen MMV.

## References

[1] (2022). World Malaria Report.

[2] Burrows J.N., Duparc S., Gutteridge W.E., Hooft van Huijsduijnen R., Kaszubska W., Macintyre F., Mazzuri S., Möhrle J.J., Wells T.N.C. (2017). New developments in anti-malarial target candidate and product profiles. Malar. J..

[3] Ashley E.A., Dhorda M., Fairhurst R.M., Amaratunga C., Lim P., Suon S., Sreng S., Anderson J.M., Mao S., Sam B., Sopha C., Chuor C.M., Nguon C., Sovannaroth S., Pukrittayakamee S., Jittamala P., Chotivanich K., Chutasmit K., Suchatsoonthorn C., Runcharoen R., Hien T.T., Thuy-Nhien N.T., Thanh N.V., Phu N.H., Htut Y., Han K.T., Aye K.H., Mokuolu O.A., Olaosebikan R.R., Folaranmi O.O., Mayxay M., Khanthavong M., Hongvanthong B., Newton P.N., Onyamboko M.A., Fanello C.I., Tshefu A.K., Mishra N., Valecha N., Phyo A.P., Nosten F., Yi P., Tripura R., Borrmann S., Bashraheil M., Peshu J., Faiz M.A., Ghose A., Hossain M.A., Samad R., Rahman M.R., Hasan M.M., Islam A., Miotto O., Amato R., MacInnis B., Stalker J., Kwiatkowski D.P., Bozdech Z., Jeeyapant A., Cheah P.Y., Sakulthaew T., Chalk J., Intharabut B., Silamut K., Lee S.J., Vihokhern B., Kunasol C., Imwong M., Tarning J., Taylor W.J., Yeung S., Woodrow C.J., Flegg J.A., Das D., Smith J., Venkatesan M., Plowe C.V., Stepniewska K., Guerin P.J., Dondorp A.M., Day N.P., White N.J. (2014). Spread of artemisinin resistance in *Plasmodium falciparum* malaria. N. Engl. J. Med..

[4] Uwimana A., Legrand E., Stokes B.H., Ndikumana J.-L.M., Warsame M., Umulisa N., Ngamije D., Munyaneza T., Mazarati J.-B., Munguti K., Campagne P., Criscuolo A., Ariey F., Murindahabi M., Ringwald P., Fidock D.A., Mbituyumuremyi A., Menard D. (2020). Emergence and clonal expansion of in vitro artemisinin-resistant *Plasmodium falciparum* kelch13 R561H mutant parasites in Rwanda. Nat. Med..

[5] Ashton T.D., Devine S.M., Möhrle J.J., Laleu B., Burrows J.N., Charman S.A., Creek D.J., Sleebs B.E. (2019). The development process for discovery and clinical advancement of modern antimalarials. J. Med. Chem..

[6] McCarthy J.S., Lotharius J., Rückle T., Chalon S., Phillips M.A., Elliott S., Sekuloski S., Griffin P., Ng C.L., Fidock D.A., Marquart L., Williams N.S., Gobeau N., Bebrevska L., Rosario M., Marsh K., Möhrle J.J. (2017). Safety, tolerability, pharmacokinetics, and activity of the novel long-acting antimalarial DSM265: a two-part first-in-human phase 1a/1b randomised study. Lancet Infect. Dis..

[7] Schmitt E.K., Ndayisaba G., Yeka A., Asante K.P., Grobusch M.P., Karita E., Mugerwa H., Asiimwe S., Oduro A., Fofana B., Doumbia S., Su G., Csermak Renner K., Venishetty V.K., Sayyed S., Straimer J., Demin I., Barsainya S., Boulton C., Gandhi P. (2022). Efficacy of cipargamin (KAE609) in a randomized, phase II dose-escalation study in adults in sub-Saharan Africa with uncomplicated *Plasmodium falciparum* malaria. Clin. Infect. Dis..

[8] Nguyen W., Dans M.G., Currie I., Awalt J.K., Bailey B.L., Lumb C., Ngo A., Favuzza P., Palandri J., Ramesh S., Penington J., Jarman K.E., Mukherjee P., Chakraborty A., Maier A.G., van Dooren G.G., Papenfuss T., Wittlin S., Churchyard A., Baum J., Winzeler E.A., Baud D., Brand S., Jackson P.F., Cowman A.F., Sleebs B.E. (2023). 7-N-Substituted-3-oxadiazole quinolones with potent antimalarial activity target the cytochrome bc(1) complex. ACS Infect. Dis..

[9] Bailey B.L., Nguyen W., Ngo A., Goodman C.D., Gancheva M.R., Favuzza P., Sanz L.M., Gamo F.-J., Lowes K.N., McFadden G.I., Wilson D.W., Laleu B., Brand S., Jackson P.F., Cowman A.F., Sleebs B.E. (2021). Optimisation of 2-(N-phenyl carboxamide) triazolopyrimidine antimalarials with moderate to slow acting erythrocytic stage activity. Bioorg. Chem..

[10] Ashton T.D., Dans M.G., Favuzza P., Ngo A., Lehane A.M., Zhang X., Qiu D., Chandra Maity B., De N., Schindler K.A., Yeo T., Park H., Uhlemann A.-C., Churchyard A., Baum J., Fidock D.A., Jarman K.E., Lowes K.N., Baud D., Brand S., Jackson P.F., Cowman A.F., Sleebs B.E. (2023). Optimization of 2,3-dihydroquinazolinone-3-carboxamides as antimalarials targeting PfATP4. J. Med. Chem..

[11] Ashton T.D., Calic P.P.S., Dans M.G., Ooi Z.K., Zhou Q., Palandri J., Loi K., Jarman K.E., Qiu D., Lehane A.M., Maity B.C., De N., Giannangelo C., MacRaild C.A., Creek D.J., Mao E.Y., Gancheva M.R., Wilson D.W., Chowdury M., de Koning-Ward T.F., Famodimu M.T., Delves M.J., Pollard H., Sutherland C.J., Baud D., Brand S., Jackson P.F., Cowman A.F., Sleebs B.E. (2024). Property and activity refinement of dihydroquinazolinone-3-carboxamides as orally efficacious antimalarials that target PfATP4. J. Med. Chem..

[12] Ashton T.D., Calic P.P.S., Dans M.G., Ooi Z.K., Zhou Q., Loi K., Jarman K.E., Palandri J., Qiu D., Lehane A.M., Maity B., De N., Famodimu M.T., Delves M.J., Mao E.Y., Gancheva M.R., Wilson D.W., Chowdury M., de Koning-Ward T.F., Baud D., Brand S., Jackson P.F., Cowman A.F., Sleebs B.E. (2024). Lactam truncation yields a dihydroquinazolinone scaffold with potent antimalarial activity that targets PfATP4. ChemMedChem.

[13] Dans M.G., Boulet C., Watson G.M., Nguyen W., Dziekan J.M., Evelyn C., Reaksudsan K., Mehra S., Razook Z., Geoghegan N.D., Mlodzianoski M.J., Goodman C.D., Ling D.B., Jonsdottir T.K., Tong J., Famodimu M.T., Kouskousis B., Delves M.J., McFadden G.I., Barry A.E., Crabb B.S., de Koning-Ward T.F., Rogers K.L., Cowman A.F., Tham W.-H., Sleebs B.E., Gilson P.R. (2024). Aryl amino acetamides prevent the development of *Plasmodium falciparum* rings via targeting the lipid transfer protein PfSTART1. Nat. Commun..

[14] Nguyen W., Boulet C., Dans M.G., Loi K., Jarman K.E., Watson G.M., Tham W.-H., Fairhurst K.J., Yeo T., Fidock D.A., Wittlin S., Chowdury M., de Koning-Ward T.F., Chen G., Yan D., Charman S.A., Baud D., Brand S., Jackson P.F., Cowman A.F., Gilson P.R., Sleebs B.E. (2024). Activity refinement of aryl amino acetamides that target the *P. falciparum* STAR-related lipid transfer 1 protein. Eur. J. Med. Chem..

[15] Calic P.S., Ashton T.D., Mansouri M., Loi K., Jarman K.E., Qiu D., Lehane A.M., Roy S., Rao G.P., Maity B.C., Wittlin S., Crespo B., Gamo F.J., Deni I., Fidock D.A., Chowdury M., de Koning-Ward T.F., Cowman A.F., Jackson P.F., Baud D., Brand S., Laleu B., Sleebs B.E. (2024). Optimization of pyrazolopyridine 4-carboxamides with potent antimalarial activity that is resistant to the *P. falciparum* transporter ABCI3. Eur. J. Med. Chem..

[16] Antonova-Koch Y., Meister S., Abraham M., Luth M.R., Ottilie S., Lukens A.K., Sakata-Kato T., Vanaerschot M., Owen E., Jado J.C., Maher S.P., Calla J., Plouffe D., Zhong Y., Chen K., Chaumeau V., Conway A.J., McNamara C.W., Ibanez M., Gagaring K., Serrano F.N., Eribez K., Taggard C.M., Cheung A.L., Lincoln C., Ambachew B., Rouillier M., Siegel D., Nosten F., Kyle D.E., Gamo F.-J., Zhou Y., Llinás M., Fidock D.A., Wirth D.F., Burrows J., Campo B., Winzeler E.A. (2018). Open-source discovery of chemical leads for next-generation chemoprotective antimalarials. Science.

[17] Hayward J.A., Makota F.V., Cihalova D., Leonard R.A., Rajendran E., Zwahlen S.M., Shuttleworth L., Wiedemann U., Spry C., Saliba K.J., Maier A.G., van Dooren G.G. (2023). A screen of drug-like molecules identifies chemically diverse electron transport chain inhibitors in apicomplexan parasites. PLoS Pathog..

[18] Painter H.J., Morrisey J.M., Mather M.W., Vaidya A.B. (2007). Specific role of mitochondrial electron transport in blood-stage *Plasmodium falciparum*. Nature.

[19] Phillips M.A., Lotharius J., Marsh K., White J., Dayan A., White K.L., Njoroge J.W., El Mazouni F., Lao Y., Kokkonda S., Tomchick D.R., Deng X., Laird T., Bhatia S.N., March S., Ng C.L., Fidock D.A., Wittlin S., Lafuente-Monasterio M., Benito F.J., Alonso L.M., Martinez M.S., Jimenez-Diaz M.B., Bazaga S.F., Angulo-Barturen I., Haselden J.N., Louttit J., Cui Y., Sridhar A., Zeeman A.M., Kocken C., Sauerwein R., Dechering K., Avery V.M., Duffy S., Delves M., Sinden R., Ruecker A., Wickham K.S., Rochford R., Gahagen J., Iyer L., Riccio E., Mirsalis J., Bathhurst I., Rueckle T., Ding X., Campo B., Leroy D., Rogers M.J., Rathod P.K., Burrows J.N., Charman S.A. (2015). A long-duration dihydroorotate dehydrogenase inhibitor (DSM265) for prevention and treatment of malaria. Sci. Transl. Med..

[20] Nixon G.L., Pidathala C., Shone A.E., Antoine T., Fisher N., O'Neill P.M., Ward S.A., Biagini G.A. (2013). Targeting the mitochondrial electron transport chain of *Plasmodium falciparum*: new strategies towards the development of improved antimalarials for the elimination era. Future Med. Chem..

[21] McKeage K., Scott L. (2003). Atovaquone/proguanil: a review of its use for the prophylaxis of *Plasmodium falciparum* malaria. Drugs.

[22] Birth D., Kao W.-C., Hunte C. (2014). Structural analysis of atovaquone-inhibited cytochrome bc1 complex reveals the molecular basis of antimalarial drug action. Nat. Commun..

[23] Nilsen A., LaCrue A.N., White K.L., Forquer I.P., Cross R.M., Marfurt J., Mather M.W., Delves M.J., Shackleford D.M., Saenz F.E., Morrisey J.M., Steuten J., Mutka T., Li Y., Wirjanata G., Ryan E., Duffy S., Kelly J.X., Sebayang B.F., Zeeman A.M., Noviyanti R., Sinden R.E., Kocken C.H.M., Price R.N., Avery V.M., Angulo-Barturen I., Jiménez-Díaz M.B., Ferrer S., Herreros E., Sanz L.M., Gamo F.J., Bathurst I., Burrows J.N., Siegl P., Guy R.K., Winter R.W., Vaidya A.B., Charman S.A., Kyle D.E., Manetsch R., Riscoe M.K. (2013). Quinolone-3-diarylethers: a new class of antimalarial drug. Sci. Transl. Med..

[24] Painter H.J., Morrisey J.M., Mather M.W., Orchard L.M., Luck C., Smilkstein M.J., Riscoe M.K., Vaidya A.B., Llinás M. (2021). Atypical molecular basis for drug resistance to mitochondrial function inhibitors in *Plasmodium falciparum*. Antimicrob. Agents Chemother..

[25] Siegel S., Rivero A., Kyle D. (2014). Molecular basis of extreme resistance in *Plasmodium falciparum* to atovaquone and other mitochondrial inhibitors. Malar. J..

[26] Gilson P.R., Tan C., Jarman K.E., Lowes K.N., Curtis J.M., Nguyen W., Di Rago A.E., Bullen H.E., Prinz B., Duffy S., Baell J.B., Hutton C.A., Jousset Subroux H., Crabb B.S., Avery V.M., Cowman A.F., Sleebs B.E. (2017). Optimization of 2-anilino 4-amino substituted quinazolines into potent antimalarial agents with oral in vivo activity. J. Med. Chem..

[27] Cerny M.A., Hanzlik R.P. (2006). Cytochrome P450-catalyzed oxidation of N-benzyl-N-cyclopropylamine generates both cyclopropanone hydrate and 3-hydroxypropionaldehyde via hydrogen Abstraction, not single electron transfer. J. Am. Chem. Soc..

[28] Zhang Z., Huang L., Shulmeister V.M., Chi Y.I., Kim K.K., Hung L.W., Crofts A.R., Berry E.A., Kim S.H. (1998). Electron transfer by domain movement in cytochrome bc1. Nature.

[29] Lane K.D., Mu J., Lu J., Windle S.T., Liu A., Sun P.D., Wellems T.E. (2018). Selection of *Plasmodium falciparum* cytochrome B mutants by putative PfNDH2 inhibitors. Proc. Natl. Acad. Sci. U.S.A..

[30] Nam T.G., McNamara C.W., Bopp S., Dharia N.V., Meister S., Bonamy G.M., Plouffe D.M., Kato N., McCormack S., Bursulaya B., Ke H., Vaidya A.B., Schultz P.G., Winzeler E.A. (2011). A chemical genomic analysis of decoquinate, a *Plasmodium falciparum* cytochrome b inhibitor. ACS Chem. Biol..

[31] Dong C.K., Urgaonkar S., Cortese J.F., Gamo F.J., Garcia-Bustos J.F., Lafuente M.J., Patel V., Ross L., Coleman B.I., Derbyshire E.R., Clish C.B., Serrano A.E., Cromwell M., Barker R.H., Dvorin J.D., Duraisingh M.T., Wirth D.F., Clardy J., Mazitschek R. (2011). Identification and validation of tetracyclic benzothiazepines as Plasmodium falciparum cytochrome bc1 inhibitors. Chem. Biol..

[32] Capper M.J., O'Neill P.M., Fisher N., Strange R.W., Moss D., Ward S.A., Berry N.G., Lawrenson A.S., Hasnain S.S., Biagini G.A., Antonyuk S.V. (2015). Antimalarial 4(1H)-pyridones bind to the Qi site of cytochrome bc1. Proc. Natl. Acad. Sci. U.S.A..

[33] Ding X.C., Ubben D., Wells T.N.C. (2012). A framework for assessing the risk of resistance for anti-malarials in development. Malar. J..

[34] Kümpornsin K., Kochakarn T., Yeo T., Okombo J., Luth M.R., Hoshizaki J., Rawat M., Pearson R.D., Schindler K.A., Mok S., Park H., Uhlemann A.C., Jana G.P., Maity B.C., Laleu B., Chenu E., Duffy J., Moliner Cubel S., Franco V., Gomez-Lorenzo M.G., Gamo F.J., Winzeler E.A., Fidock D.A., Chookajorn T., Lee M.C.S. (2023). Generation of a mutator parasite to drive resistome discovery in *Plasmodium falciparum*. Nat. Commun..

[35] Zafra C.A., Crispim M., Verdaguer I.B., Ríos A.G., Moura G.C., Katzin A.M., Hernández A. (2023). *Plasmodium falciparum* COQ2 gene encodes a functional 4-hydroxybenzoate polyprenyltransferase. FEMS Microbiol. Lett..

[36] Saggu G.S., Garg S., Pala Z.R., Yadav S.K., Kochar S.K., Kochar D.K., Saxena V. (2017). Characterization of 4-hydroxy-3-methylbut-2-en-1-yl diphosphate synthase (IspG) from *Plasmodium vivax* and it's potential as an antimalarial drug target. Int. J. Biol. Macromol..

[37] Stickles A.M., de Almeida M.J., Morrisey J.M., Sheridan K.A., Forquer I.P., Nilsen A., Winter R.W., Burrows J.N., Fidock D.A., Vaidya A.B., Riscoe M.K. (2015). Subtle changes in endochin-like quinolone structure alter the site of inhibition within the cytochrome bc1 complex of *Plasmodium falciparum*. Antimicrob. Agents Chemother..

[38] Sanz L.M., Crespo B., De-Cózar C., Ding X.C., Llergo J.L., Burrows J.N., García-Bustos J.F., Gamo F.J. (2012). *P. falciparum* in vitro killing rates allow to discriminate between different antimalarial mode-of-action. PLoS One.

[39] Barata L., Houzé P., Boutbibe K., Zanghi G., Franetich J.-F., Mazier D., Clain J. (2016). In vitro analysis of the interaction between atovaquone and proguanil against liver stage malaria parasites. Antimicrob. Agents Chemother..

[40] Fowler R.E., Sinden R.E., Pudney M. (1995). Inhibitory activity of the anti-malarial atovaquone (566C80) against ookinetes, oocysts, and sporozoites of *Plasmodium berghei*. J. Parasitol..

[41] Ruecker A., Mathias D.K., Straschil U., Churcher T.S., Dinglasan R.R., Leroy D., Sinden R.E., Delves M.J. (2014). A male and female gametocyte functional viability assay to identify biologically relevant malaria transmission-blocking drugs. Antimicrob. Agents Chemother..

[42] Swann J., Corey V., Scherer C.A., Kato N., Comer E., Maetani M., Antonova-Koch Y., Reimer C., Gagaring K., Ibanez M., Plouffe D., Zeeman A.-M., Kocken C.H.M., McNamara C.W., Schreiber S.L., Campo B., Winzeler E.A., Meister S. (2016). High-throughput luciferase-based assay for the discovery of therapeutics that prevent malaria. ACS Infect. Dis..

[43] Franke-Fayard B., Trueman H., Ramesar J., Mendoza J., van der Keur M., van der Linden R., Sinden R.E., Waters A.P., Janse C.J. (2004). A *Plasmodium berghei* reference line that constitutively expresses GFP at a high level throughout the complete life cycle. Mol. Biochem. Parasitol..

[44] Monastyrskyi A., Kyle D.E., Manetsch R. (2014). 4(1H)-pyridone and 4(1H)-quinolone derivatives as antimalarials with erythrocytic, exoerythrocytic, and transmission blocking activities. Curr. Top. Med. Chem..

[45] LaCrue Alexis N., Sáenz Fabián E., Cross R.M., Udenze Kenneth O., Monastyrskyi A., Stein S., Mutka Tina S., Manetsch R., Kyle Dennis E. (2013). 4(1H)-Quinolones with liver stage activity against *Plasmodium berghei*. Antimicrob. Agents Chemother..

[46] Okamoto N., Spurck T.P., Goodman C.D., McFadden G.I. (2009). Apicoplast and mitochondrion in gametocytogenesis of *Plasmodium falciparum*. Eukaryot. Cell.

[47] Ke H., Lewis I.A., Morrisey J.M., McLean K.J., Ganesan S.M., Painter H.J., Mather M.W., Jacobs-Lorena M., Llinás M., Vaidya A.B. (2015). Genetic investigation of tricarboxylic acid metabolism during the *Plasmodium falciparum* life cycle. Cell Rep..

[48] Talman A.M., Prieto J.H., Marques S., Ubaida-Mohien C., Lawniczak M., Wass M.N., Xu T., Frank R., Ecker A., Stanway R.S., Krishna S., Sternberg M.J.E., Christophides G.K., Graham D.R., Dinglasan R.R., Yates J.R., Sinden R.E. (2014). Proteomic analysis of the *Plasmodium* male gamete reveals the key role for glycolysis in flagellar motility. Malar. J..

[49] Gilson P.R., Nguyen W., Poole W.A., Teixeira J.E., Thompson J.K., Guo K., Stewart R.J., Ashton T.D., White K.L., Sanz L.M., Gamo F.J., Charman S.A., Wittlin S., Duffy J., Tonkin C.J., Tham W.H., Crabb B.S., Cooke B.M., Huston C.D., Cowman A.F., Sleebs B.E. (2019). Evaluation of 4-amino 2-anilinoquinazolines against *Plasmodium* and Other Apicomplexan parasites in vitro and in a *P. falciparum* humanized NOD-scid IL2Rγ(null) mouse model of malaria. Antimicrob. Agents Chemother..

[50] Nguyen W., Hodder A.N., de Lezongard R.B., Czabotar P.E., Jarman K.E., O'Neill M.T., Thompson J.K., Jousset Sabroux H., Cowman A.F., Boddey J.A., Sleebs B.E. (2018). Enhanced antimalarial activity of plasmepsin V inhibitors by modification of the P(2) position of PEXEL peptidomimetics. Eur. J. Med. Chem..

[51] Ashton T.D., Ngo A., Favuzza P., Bullen H.E., Gancheva M.R., Romeo O., Parkyn Schneider M., Nguyen N., Steel R.W.J., Duffy S., Lowes K.N., Sabroux H.J., Avery V.M., Boddey J.A., Wilson D.W., Cowman A.F., Gilson P.R., Sleebs B.E. (2021). Property activity refinement of 2-anilino 4-amino substituted quinazolines as antimalarials with fast acting asexual parasite activity. Bioorg. Chem..

[52] Waterhouse A., Bertoni M., Bienert S., Studer G., Tauriello G., Gumienny R., Heer F.T., de Beer T.A.P., Rempfer C., Bordoli L., Lepore R., Schwede T. (2018). SWISS-MODEL: homology modelling of protein structures and complexes. Nucleic Acids Res..

[53] da Cruz F.P., Martin C., Buchholz K., Lafuente-Monasterio M.J., Rodrigues T., Sönnichsen B., Moreira R., Gamo F.J., Marti M., Mota M.M., Hannus M., Prudêncio M. (2012). Drug screen targeted at *Plasmodium* liver stages identifies a potent multistage antimalarial drug. J. Infect. Dis..

[54] Yalaoui S., Zougbédé S., Charrin S., Silvie O., Arduise C., Farhati K., Boucheix C., Mazier D., Rubinstein E., Froissard P. (2008). Hepatocyte permissiveness to *Plasmodium* infection is conveyed by a short and structurally conserved region of the CD81 large extracellular domain. PLoS Pathog..

[55] Delves M.J., Straschil U., Ruecker A., Miguel-Blanco C., Marques S., Dufour A.C., Baum J., Sinden R.E. (2016). Routine in vitro culture of *P. falciparum* gametocytes to evaluate novel transmission-blocking interventions. Nat. Protoc..

