# Supplementary material for: Exploration and characterization of the antimalarial activity of cyclopropyl carboxamides that target the mitochondrial protein, cytochrome b
Source: Eur J Med Chem. 2024 Dec 15;280:116921. doi: 10.1016/j.ejmech.2024.116921 (PMC11609934; doi:10.1016/j.ejmech.2024.116921)
Supplement: Multimedia component 1 [file mmc1.docx]

*SUPPORTING INFORMATION FOR*

**Exploration and Characterization of the Antimalarial Activity of Cyclopropyl Carboxamides that Target the Mitochondrial Protein, Cytochrome b**

Jon Kyle Awalt,^a,b,#^ Wenyin Su,^a,b,#^ William Nguyen,^a,b^ Katie Loi,^a^ Kate E. Jarman,^a,b^ Jocelyn S. Penington,^a,b^ Saishyam Ramesh,^c^ Kate J. Fairhurst,^d,e^ Tomas Yeo,^d,e^ Heekuk Park,^e,f^ Anne-Catrin Uhlemann,^e,f^ Bikash Chandra Maity,^g^ Nirupam De,^g^ Partha Mukherjee,^g^ Arnish Chakraborty,^g^ Alisje Churchyard,^h^ Mufuliat T. Famodimu,^i^ Michael J. Delves,^i^ Jake Baum,^h,j^ Nimisha Mittal,^k^ Elizabeth A. Winzeler,^k^ Anthony T. Papenfuss,^a,b^ Mrittika Chowdury,^l,m^ Tania F. de Koning-Ward,^l,m^ Alexander G. Maier,^c^ Giel G. van Dooren,^c^ Delphine Baud,^n^ Stephen Brand,^n^ David A. Fidock,^d,e,f^ Paul F. Jackson,^o^ Alan F. Cowman,^a,b^ and Madeline G. Dans,^a,b,*^ Brad E. Sleebs.^a,b,*^

^a^ The Walter and Eliza Hall Institute of Medical Research, Parkville 3052, Australia.

^b^ Department of Medical Biology, The University of Melbourne, Parkville 3010, Australia.

^c^ Research School of Biology, The Australian National University, Canberra 2600, Australia.

^d^ Department of Microbiology & Immunology, Columbia University Irving Medical Center, New York, NY, USA

^e^ Center for Malaria Therapeutics and Antimicrobial Resistance, Columbia University Irving Medical Center, New York, NY, USA

^f^ Division of Infectious Diseases, Department of Medicine, Columbia University Irving Medical Center, New York, NY, USA

^g^ TCG Lifesciences, Kolkata, West Bengal, 700091, India.

^h^ Department of Life Sciences, Imperial College London, South Kensington, SW7 2AZ UK.

^i^ Department of Infection Biology, London School of Hygiene and Tropical Medicine, London, WC1E 7HT, UK.

^j^ School of Biomedical Sciences, University of New South Wales, Sydney, 2031, Australia.

^k^ School of Medicine, University of California San Diego, La Jolla, CA, 92093, USA.

^l^ School of Medicine, Deakin University, Waurn Ponds, Victoria 3216, Australia

^m^ Institute for Mental and Physical Health and Clinical Translation, Deakin University, Geelong, Victoria, 3216, Australia

^n^ Medicines for Malaria Venture, Geneva 1215, Switzerland.

^o^ Global Public Health, Janssen R&D LLC, La Jolla 92121, USA.

^*^ Correspondence to:

Brad E. Sleebs and Madeline G. Dans

The Walter and Eliza Hall Institute of Medical Research

1G Royal Parade, Parkville 3052, Victoria, Australia

Email: [sleebs@wehi.edu.au](mailto:sleebs@wehi.edu.au) ; [dans.m@wehi.edu.au](mailto:dans.m@wehi.edu.au)

**Index**

Page

S4 Schemes S1-3 Synthetic pathways for analogs

S5 Schemes S4-6 Synthetic pathways for analogs

S6 Figure S1 *P. falciparum* 3D7 asexual dose response curves

S7 Table S1 Mouse liver microsomal W499 (**2**) metabolite identification

S7 Figure S2 Mouse liver microsomal W499 (**2**) metabolite identification

S8 Table S2 Sequencing depth for W466-resistant parasites

S8 Table S3 Sequences received for W466-resistant parasites

S9 Table S4 SNPs identified from W466-resistant genomes

S9 Table S5 Prevalence of SNPs from W466-resistant genomes

S10 Figure S3 CNV events from W466-resistant parasites

S11 Figure S4 Detailed CNV events for W466-resistant parasites

S12 Table S6 Structural variant events for W499-resistant parasites

S13 Table S7 CNVs for W499-resistant parasites

S14 Figure S5 CNV events from W499-resistant parasites

S15 Figure S6 Detailed CNV events from W499**-**resistant parasites

S16 Table S8 Summary of the MIR on W499 (**2**)

S16 Table S9 Summary of the MIR on W466 (**1**)

S17 Figure S7 IC_50_ and IC_90_ shifts of W466-recrudescent Dd2 parasites from MIR

S17 Table S10 IC_50_ and IC_90_ shifts of W466-recrudescent Dd2 parasites from MIR

S18 Figure S8 IC_50_ and IC_90_ shifts of W466-recrudescent Dd2_Polδ parasites from MIR

S18 Table S11 IC_50_ and IC_90_ shifts of W466-recrudescent Dd2_Polδ parasites from MIR

S19 Table S12 WGS metrics for Dd2 W466-resistant H3 population from MIR

S19 Table S13 SNPs observed for Dd2 W466-resistant H3 population from MIR

S20 Table S14 WGS metrics for Dd2_Polδ W466-resistant F3 clones

S21 Table S15 SNPs observed for Dd2-Polδ W466-resistant F3 clones

S22 Table S16 SNP prevalence for Dd2-Polδ W466-resistant F3 clones

S23 Figure S9 W466-resistant population dose response curves

S24 Figure S10 W499-resistant population dose response curves

S25 Figure S11 ScDHODH and SB1-A6 dose response curves

S26 Figure S12 Biological repeats for asexual stage of arrest microscopy

S27 Figure S13 NF54 gamete dose response curves

S28 Figure S14 Mouse model efficacy data

S29 NMR spectra for final compounds

**
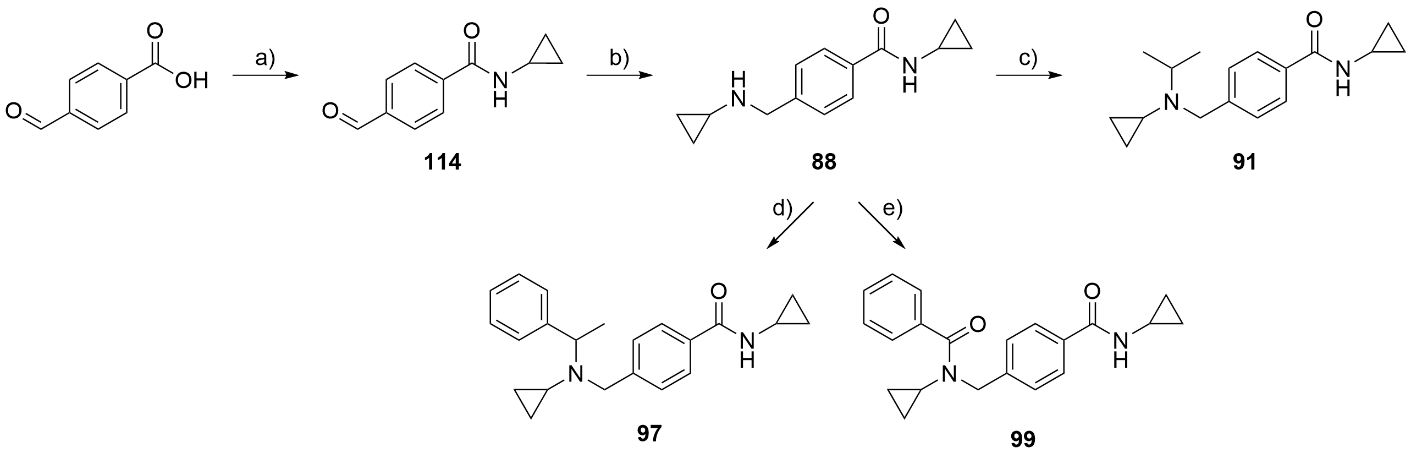
**

**Scheme S1.** Synthetic pathways to afford compounds **91**, **97** and **99**. *Reagents and conditions:* (a) i. oxalyl chloride, cat. DMF, DCM, 0 °C..rt, 2 h; ii. cyclopropylamine, Et_3_N, DCM, 0 °C - rt, 2 h, 87%; (b) cyclopropylamine, AcOH, cat. MeOH, NaBH(OAc)_3_, DCE, rt, 6 h, 54%; (c) acetone, AcOH, NaBH(OAc)_3_, DCE, rt, 6 h, 67%; (d) (1-bromoethyl)benzene, K_2_CO_3_, DMF, 0 °C..rt, 6 h, 21%; (e) BzCl, Et_3_N, DCM, rt, 1 h, 50%.

**
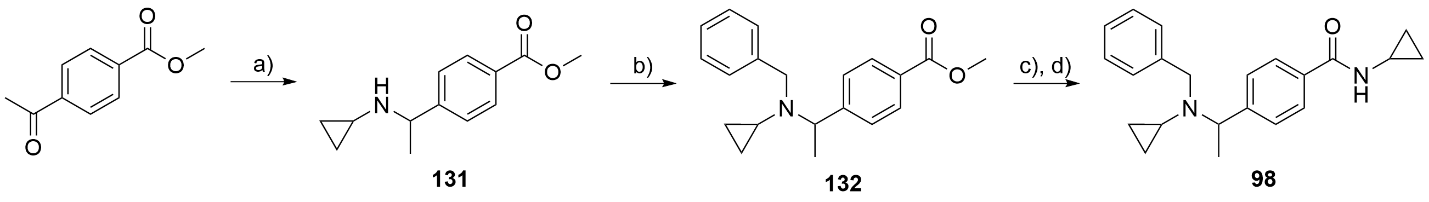
**

**Scheme S2.** Synthetic pathway to afford compound **98**. *Reagents and conditions:* (a) i. cyclopropylamine, PhMe, 110 °C, 6 h; ii. NaBH_4_, MeOH, rt, 16 h, 71%; (b) BnBr, K_2_CO_3_, DMF, 0 °C - rt, 6 h, 83%; (c) LiOH, THF, MeOH, H_2_O, 60 °C, 6 h, 91%; (d) cyclopropylamine, EDCI, HOBt, DIPEA, DMF, rt, 15 h, 20%.


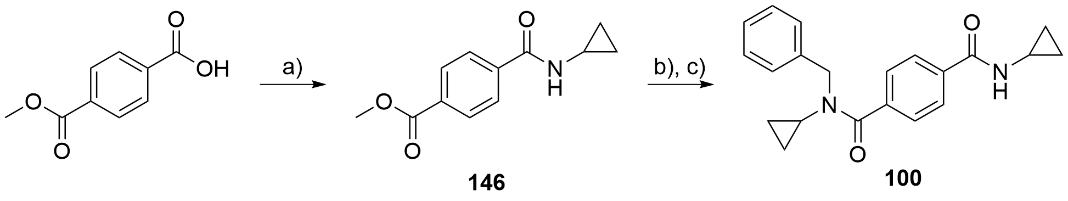


**Scheme S3**. Synthetic pathway to afford compound **100**. (a) cyclopropylamine, HATU, DIPEA, DCM, rt, 2 h, 81%; (b) LiOH, THF, H_2_O, rt, 16 h, 68%; (c) *N*-benzylcyclopropanamine, HATU, DIPEA, DCM, rt, 2 h, 43%.


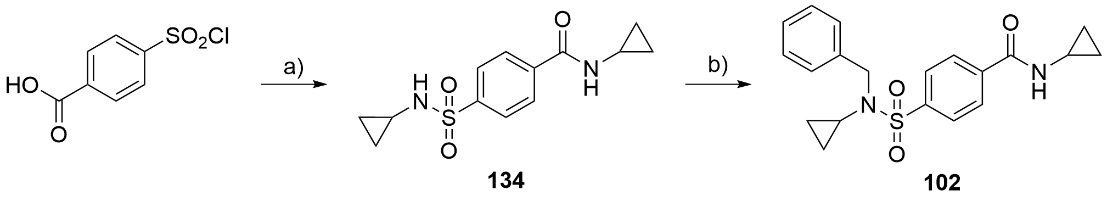


**Scheme S4**. Synthetic pathway to afford compound **102**. (a) i. cyclopropylamine, DIPEA, DMF, 0 °C, 1 h; ii. EDCI, HOBt, rt, 16 h, 32%; (b) BnBr, K_2_CO_3_, DMF, 0 °C - rt, 16 h, 63%.

**
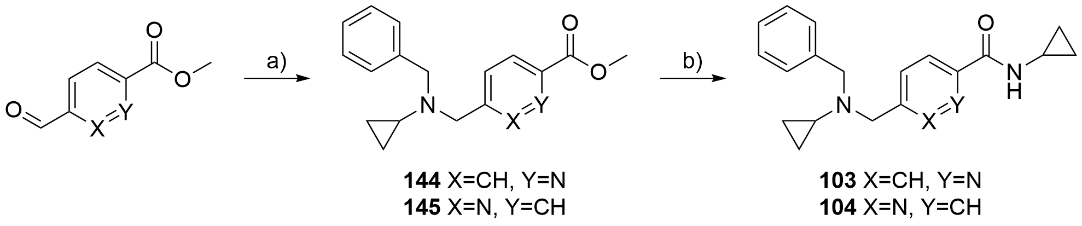
**

**Scheme S5.** Synthetic pathways to afford compounds **103** and **104.** *Reagents and conditions:* (a) *N*-benzylcyclopropanamine, NaBH(OAc)_3_, MeOH, 0 °C - rt, 34 h, 22-36%; (b) cyclopropylamine, DABAL-Me_3_, PhMe, μW, 130 °C, 296 W, 16 min, 13-47%.

**
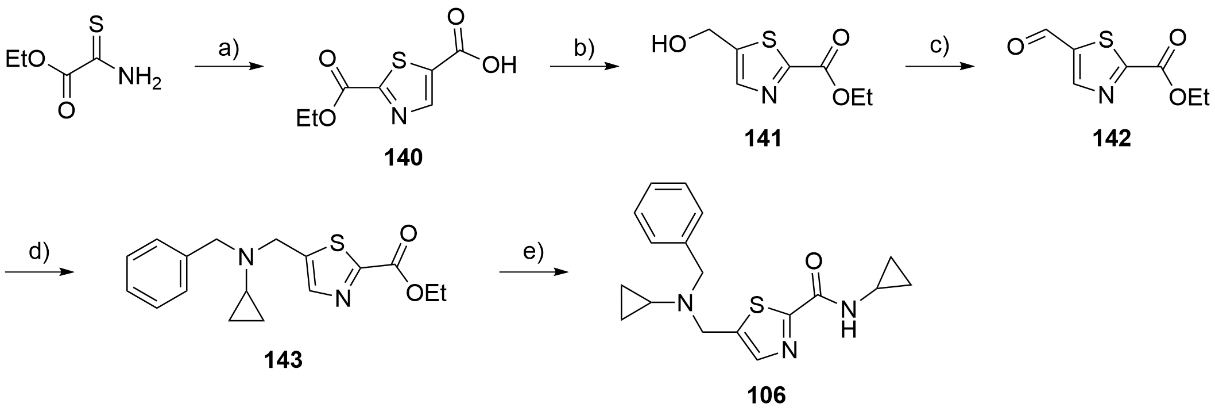
**

**Scheme S6.** Synthetic pathway to afford compound 106**.** *Reagents and conditions:* (a) 3-bromo-2-oxopropanoic acid, dioxane, reflux, 5 h. 95%; (b) BH_3_-DMS, THF, rt, 16 h, 35%; (c) Dess–Martin periodinane, DCM, 0 °C - rt, 4 h, 93%; (d) *N*-benzylcyclopropanamine, AcOH, cat. MeOH, NaBH(OAc)_3_, DCE, rt, 6 h, 39%; (e) cyclopropylamine, DMSO, 70 °C, 16 h, 9%.

Compound **2**

**
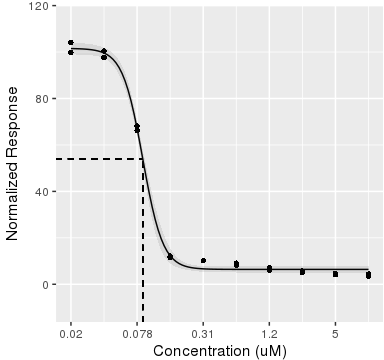

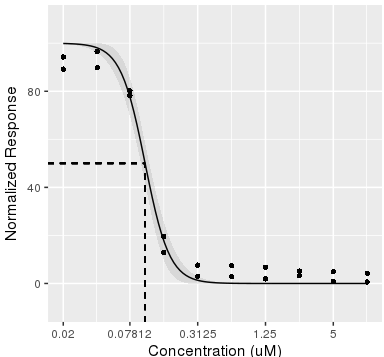

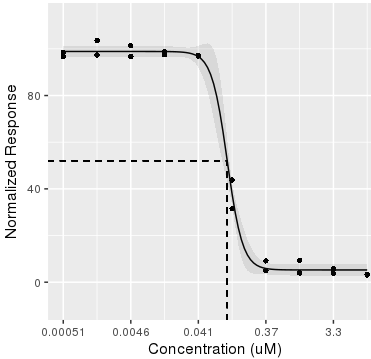
**

Compound **17**


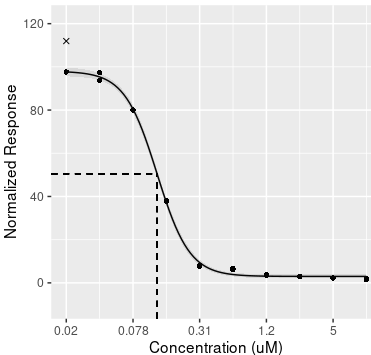

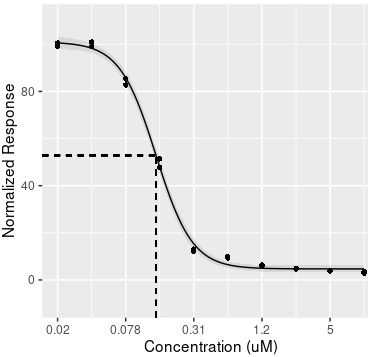

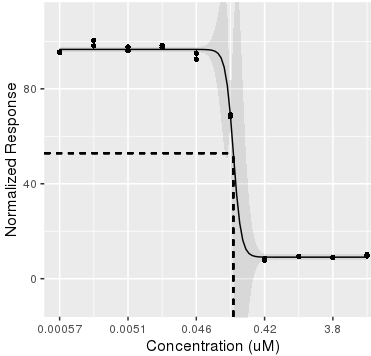


Compound WJM280 (**108**)


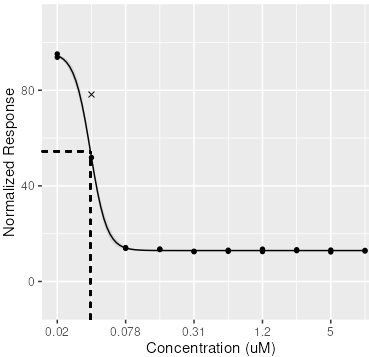

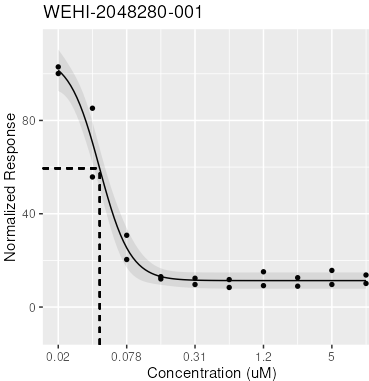

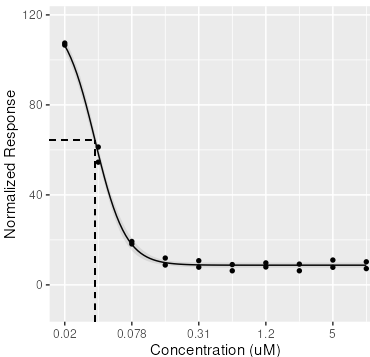


**Figure S1.** Dose response curves of selected compounds against *P. falciparum* 3D7. EC_50_ data represent means and SDs for 3 experiments measuring the LDH activity of *P. falciparum* 3D7 parasites following exposure to compounds for 72 h.

**Table S1.** Mouse liver microsomal metabolite identification for compound W499 (**2**).

| **metabolite** | **RT (min)** | **m/z** | **% metabolite abundance** | **molecular biotransformation** | **predicted**  **biotransformation** |
| --- | --- | --- | --- | --- | --- |
| M1 | 2.9 | 231 | 38 | P-158 | N-dealkylation adjacent to the chlorinated aryl ring |
| M2 | 4.2 | 206 | 3 | P-183 +2 x O | oxidative N-dealkylation adjacent to the benzene ring, resulting in a carboxylic acid |
| M3 | 4.7 | 216 | 7 | P - 40 | byproduct of M2 biotransformation |
| M4 | 5.3 | 349 | 18 | - cyPr | N-dealkylation of the terminal cyPr |
| M5 | 7.0 | 421 | 3 | +2 x O | bis-oxidation of the central cyPr |
| parent | 7.7 | 389 | - | - | - |


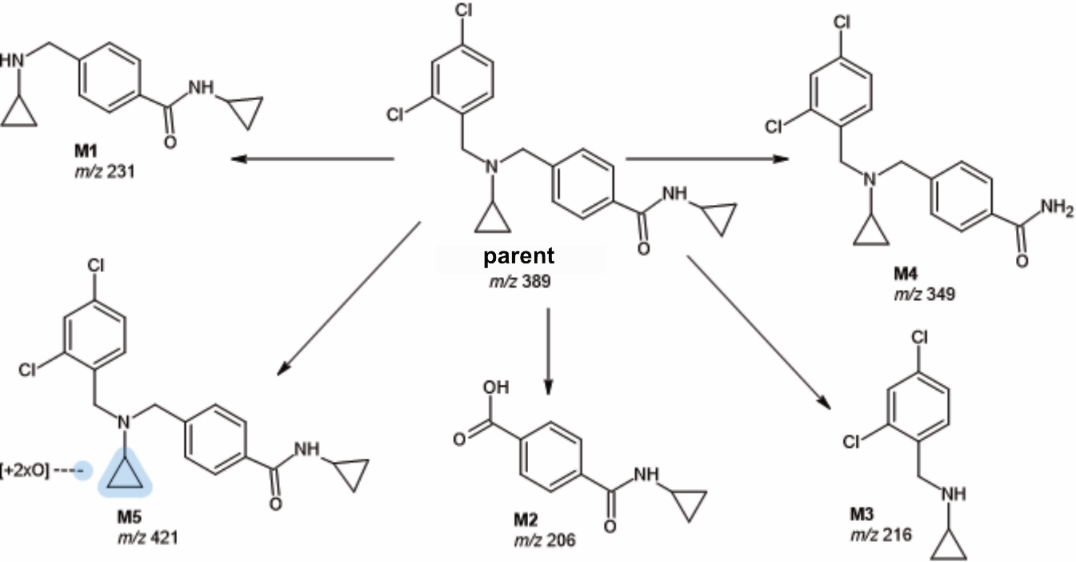


**Figure S2.** Metabolites generated from incubation of W499 (**2**) with mouse liver microsomes.

**Table S2.** Depth of sequencing for W466-resistant parasite populations 1, 2 and 3 (in duplicate), and comparison sample after aligning and filtering. Sequences were aligned to the reference PlasmoDB-52 or 54_Pfalciparum3D7 using bwa-mem with default settings. Sequences were filtered using Picard Mark Duplicates. Quality was confirmed using fastQC program. The comparison sample, 3D7_uncloned, was sequenced to a depth of 60.7 million aligned, deduplicated, paired-end sequences.

| Sample name | W466_1.1 | W466_1.2 | W466_2.1 | W466_2.2 | W466_3.1 | W466_3.2 |
| --- | --- | --- | --- | --- | --- | --- |
| Total sequences | 27.7 mil | 14.6 mil | 23.4 mil | 18.7 mil | 64.7 mil | 30.7 mil |

**Table S3.** Sequences received for four W499-resistant samples were aligned to the reference PlasmoDB-52_Pfalciparum3D7 using bwa-mem with default settings. Sequences were filtered using Picard MarkDuplicates and quality was confirmed using fastQC program.

| Sample name | W499_2.1 | W499_2.2 | W499_3.1 | W499_3.2 |
| --- | --- | --- | --- | --- |
| Total sequences | 160.2 mil | 152.3 mil | 88.7 mil | 54.1 mil |

**Table S4.** Non-synonymous single nucleotide polymorphisms identified from W466-resistant genomes.

| Chrom | Position | Base change | Amino acid change | gene ID | Gene description | Population (Samples)/  Comment |
| --- | --- | --- | --- | --- | --- | --- |
| MIT | 3855 | G->A | A122T | PF3D7_MIT02300 | CYTB | R1(1&2) |
| MIT | 4283 | T->A | F264L | PF3D7_MIT02300 | CYTB | R2(1&2)  R3(1&2) |
| 03 | 268357 | C->A | V1644L | PF3D7_0305500 | protein dopey homolog, putative | R1 (1&2) |
| 04 | 1050893 | C->G | N110K | PF3D7_0423200 | BSD-domain protein, putative | R2(1&2), R3(1&2) |
| 10 | 1437030 | A->G | T239A | PF3D7_1036400 | LSA1 | Low-to-no coverage. Present in parent sample. |
| 10 | 1437032 | T->A |  |  |  |  |
| 10 | 1437307 | A->G | E331G | PF3D7_1036400 | LSA1 | Low-to-no coverage. |
| 10 | 1437308 | A->G |  |  |  |  |
| 12 | 1116878 | T->G | N1406H | PF3D7_1227500 | SOC2 | R2(1&2), R3(1&2) |
| 14 | 2936817 | T->A | K911I | PF3D7_1471900 | Conserved unknown | Error due to highly repetitive region |
| 14 | 245161 | G->C | C85S | PF3D7_1406700 | VPS29 | R1(1&2) |

Variants were called using bcftools mpileup and call. Results were filtered to QUAL > 50, not called in parent, in “core genome” as defined by Miles et al. (Genome Res. 2016, 26(9), 1288) in a gene region excluding PfEMP1 var, rifin, stevor and pseudo genes, and in at least 2 out of the 6 samples.

**Table S5.** Allele frequencies of notable single nucleotide polymorphisms observed from whole-genome sequencing of W466-resistant genomes.

| Gene name | Amino acid change | Proportions for populations | | | | | | |
| --- | --- | --- | --- | --- | --- | --- | --- | --- |
|  |  | W466_R1-1 | W466_R1-2 | W466_R2-1 | W466_R2-2 | W466_R3-1 | W466_R3-2 | 3D7 |
| CYTB | A122T | 98% | 98% | 0.6% | 0.9% | 2.0% | 3.0% | 0.0% |
| CYTB | F264L | 0.6% | 0.6% | 99% | 95% | 98% | 97% | 0.3% |
| SOC2 | N1406H | 0.0% | 0.0% | 97% | 99% | 98% | 99% | 6.0% |
| BSD | N110K | 98% | 99% | 0.6% | 0.0% | 99% | 99% | 1.0% |


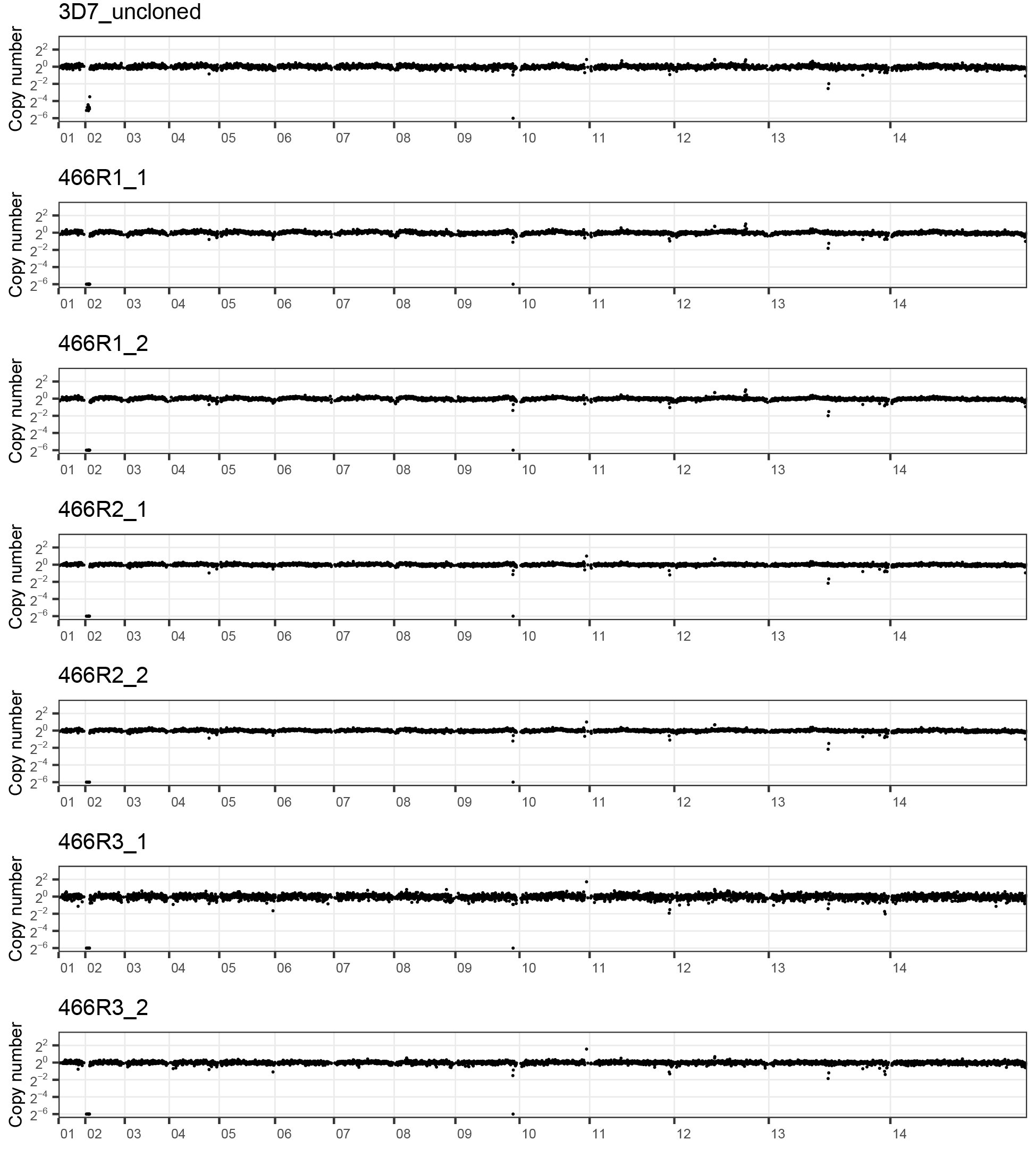


**Figure S3**. Copy numbers for W466-resistant parasite populations 1, 2 and 3 (in duplicate) with comparison sample, in 5 kb bins.

**
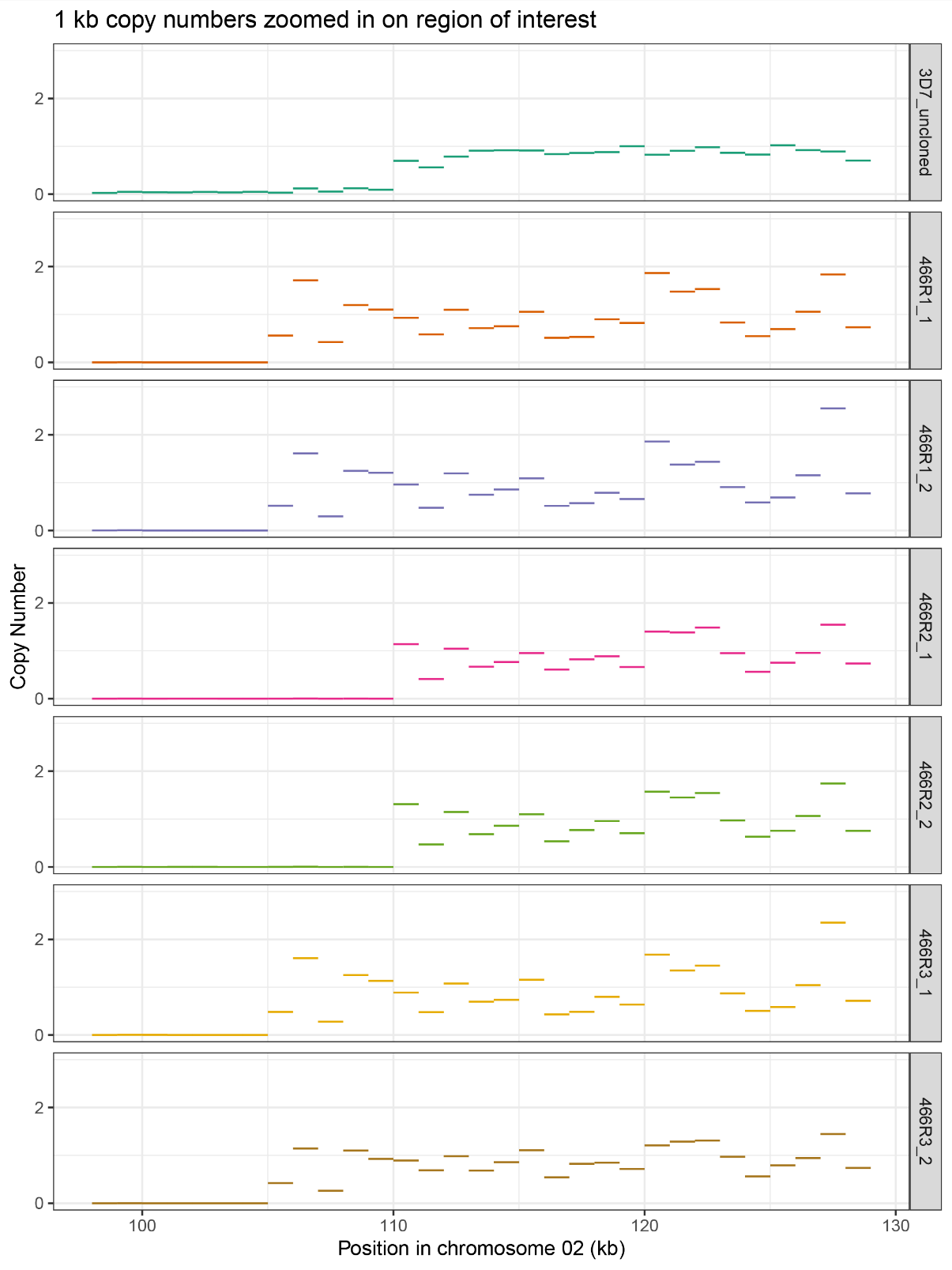
**

**Figure S4**. Detail of copy number variant events in chromosome 02 from W466-resistant parasite populations 1, 2 and 3 (in duplicate), with comparison sample. There is a deletion in all samples from the start of the chromosome to 106kb. This deletion continues to 110kb in samples W466_2.1 and W466_2.2, and sub-clonally in the parent 3D7 (deletion in LSAP2 and KAHRP) but not in the population 1 and 3 samples (deletion only in LSAP).

**Table S6.** Structural variant events in chromosome 06 supporting the amplification from W499-resistant parasite populations 2 and 3 (in duplicate). Sample allele frequencies, with 2 values where break-ends have different values.

| GRIDSS ID | Position 1 | Position 2 | W499_2.1 | W499_2.2 | W499_3.1 | W499_3.2 |
| --- | --- | --- | --- | --- | --- | --- |
| gridss0bf_62726 | 90,403 | 160,729 | 0.4 | 0.4 | 0 | 0 |
| gridss0bb_52286 | 113,044 | 120,346 | 0.2, 0.1 | 0.2, 0.1 | 0 | 0 |
| gridss0ff_52895 | 152,978 | 180,231 | 0.1, 0.3 | 0.1, 0.3 | 0 | 0 |
| gridss0bf_62951 | 104,687 | 156,425 | 0 | 0 | 0.4 | 0.4 |

| Range | Approximate copy number | sample |
| --- | --- | --- |
| Pf3D7_06_v3: 90,403..113,044 | 3 | W499_2.1 and W499_2.2 |
| Pf3D7_06_v3: 113,044..120,346 | 4 |  |
| Pf3D7_06_v3: 120,346..152,978 | 5 |  |
| Pf3D7_06_v3:152,978..160,729 | 4 |  |
| Pf3D7_06_v3:160,729..180,231 | 2 |  |
| Pf3D7_06_v3:104,687..156,425 | 4 | W499_3.1 and W499_3.2 |

**Table S7.** Genes in the amplified region (chromosome 6: 113kb – 156kb) from W499-resistant parasite populations 2 and 3.

| Gene ID | Genomic location (strand) | Product description | Gene name |
| --- | --- | --- | --- |
| PF3D7_0602700 | Pf3D7_06_v3: 113,592 - 115,478 (-) | conserved Plasmodium protein, unknown function | N/A |
| PF3D7_0602800 | Pf3D7_06_v3: 115,789 - 118,379 (+) | JmjC domain-containing protein 2, putative | JmjC2 |
| PF3D7_0602900 | Pf3D7_06_v3: 118,239 - 120,929 (-) | conserved Plasmodium protein, unknown function | N/A |
| PF3D7_0603000 | Pf3D7_06_v3: 121,248 - 124,846 (+) | SDE2 domain-containing protein, putative | N/A |
| PF3D7_0603100 | Pf3D7_06_v3: 125,002 - 127,010 (-) | RNA-binding protein, putative | N/A |
| PF3D7_0603200 | Pf3D7_06_v3: 128,292 - 130,385 (+) | mitochondrial chaperone BCS1, putative | BCS1 |
| PF3D7_0603300 | Pf3D7_06_v3: 130,191 - 132,792 (-) | dihydroorotate dehydrogenase | DHODH |
| PF3D7_0603400 | Pf3D7_06_v3: 132,888 - 138,199 (-) | trophozoite exported protein 1 | TEX1 |
| PF3D7_0603500 | Pf3D7_06_v3: 138,667 - 141,013 (+) | cation/H+ antiporter | CAX |
| PF3D7_0603600 | Pf3D7_06_v3: 140,777 - 149,585 (-) | AT-rich interactive domain-containing protein, putative | N/A |
| PF3D7_0603700 | Pf3D7_06_v3: 150,279 - 152,471 (+) | phenylalanine--tRNA ligase | mFRS |


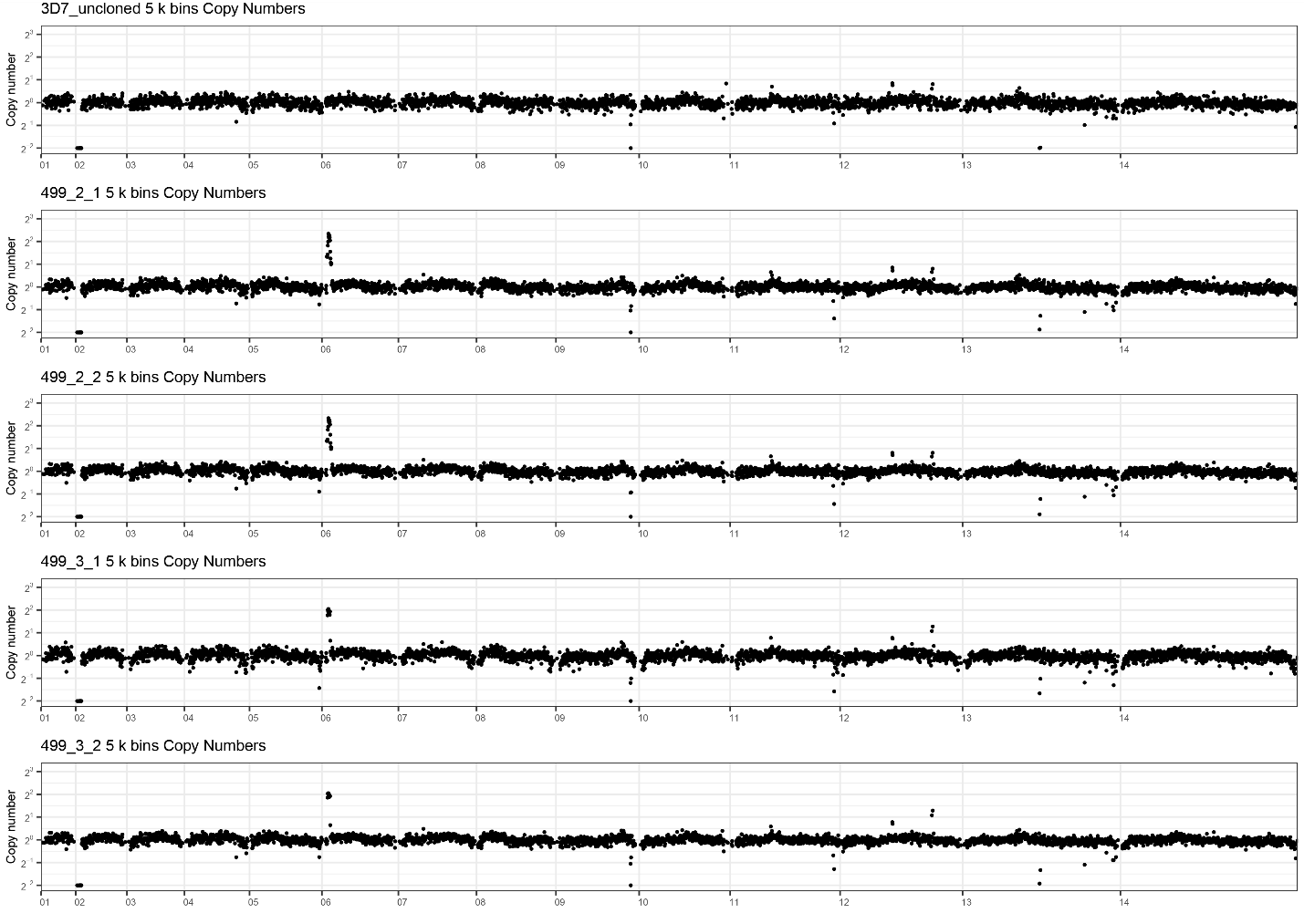


**Figure S5**. Copy numbers for W499-resistant parasite populations 2 and 3 (in duplicate), with parent 3D7 sample.


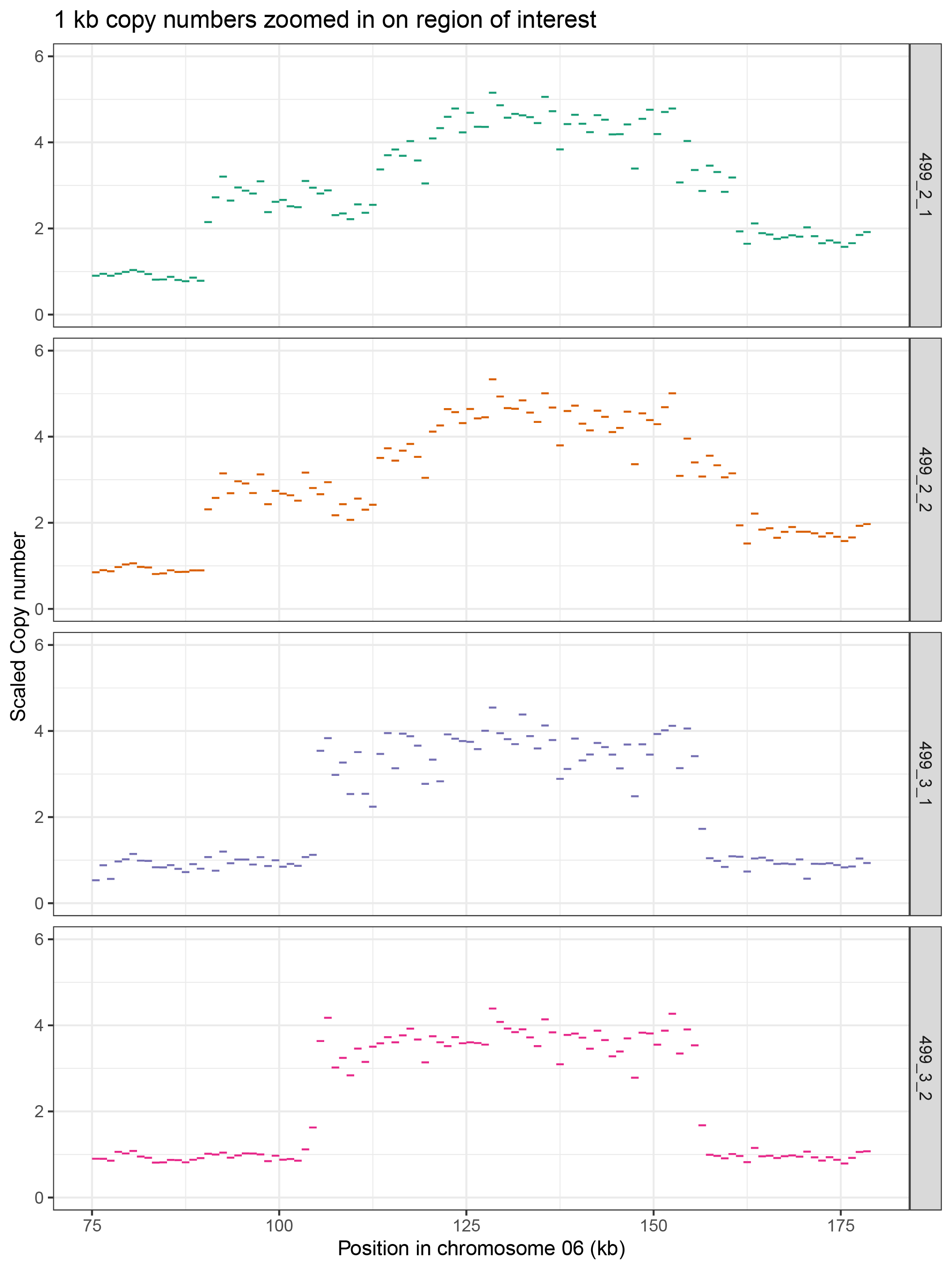


**Figure S6**. Detail of copy number variant events in chromosome 06 W499-resistant parasite populations 2 and 3 (in duplicate).

**Table S8.** Summary of the minimum inoculum of resistance (MIR) study on W499 (**2**).^a^

| **Compound:** W499 (**2**) | | |  |
| --- | --- | --- | --- |
| **Parasite Line:** Dd2-B2 | | |  |
| **IC_50_/IC_90_:** 112/224 nM | | |  |
| **Selection Pressure:** 3 x IC_90_ (671 nM) | | |  |
| **Selection:** | | 2 x 10^5^ |  |
| **Day of recrudescence:** | | N/A |  |
|  |  |  |  |
| **IC_50_ shift:** N/A | | |  |
| **MIR:** >2 x 10^7^ | **Log10 MIR:** 7.3 | |  |

**Table S9.** Summary of the minimum inoculum of resistance (MIR) study on W466 (**1**).^a^

| **Compound:** W466 (**1**). | |  |
| --- | --- | --- |
| **Parasite Line:** Dd2-B2 | |  |
| **IC_50_/IC_90_:** 226.7/711.2 nM | |  |
| **Selection Pressure:** 3 x IC_90_ (2134 nM) | |  |
| **Selection:** 2 x 10^5^ | |  |
| **Day of recrudescence:** 30 | |  |
|  |  |  |
| **IC_50_ shift:** 4.4 | |  |
| **MIR:** 1.9 x 10^7^ | **Log10 MIR:** 7.3 |  |


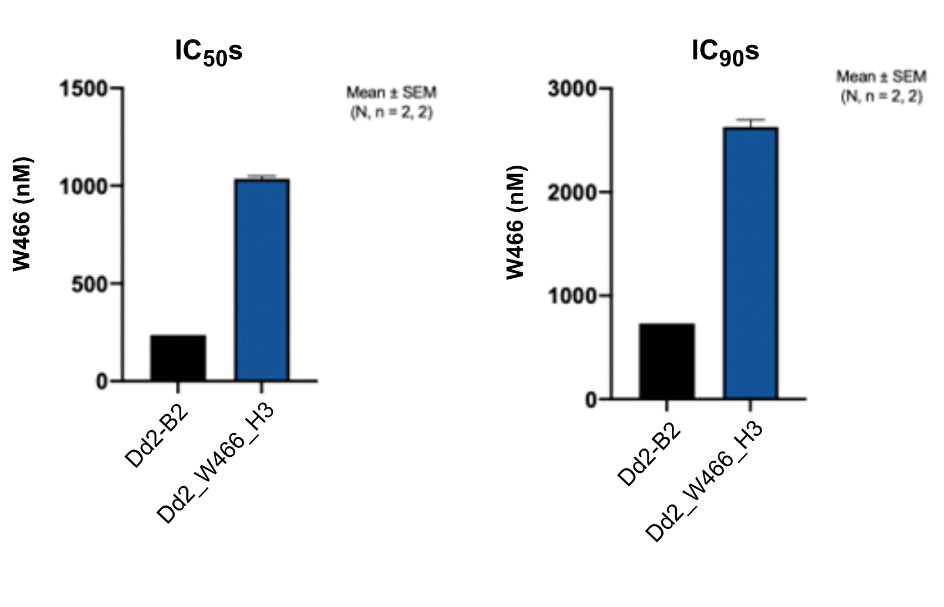


**Figure S7**. IC_50_ and IC_90_ shifts for Dd2-B2 and W466**_**H3-resistant populations obtained from the MIR study. Data shown are mean ±SEM (N, n= 2, 2).

**Table S10.** IC_50_ and IC_90_ shifts for Dd2-B2 and H3_recrudescent parasites after selection with 3x IC_90_ of compound W466 (**1**) obtained from the MIR study.

|  | Dd2-B2 | W466_H3 |
| --- | --- | --- |
| Average IC_50_ (±SEM) nM | 237 (<0.1) | 1036 (13) |
| IC_50_ fold shift | n/a | 4.4 |
| Average IC_90_ (±SEM) nM | 730 (0.3) | 2629 (69) |
| IC_90_ fold shift | n/a | 3.6 |

^a^ IC_50_ and IC_90_ values represent an average of n=2 biological replicates of a 72 h SYBR Green I and MitoTracker Deep Red FM flow cytometry assay, with technical duplicates


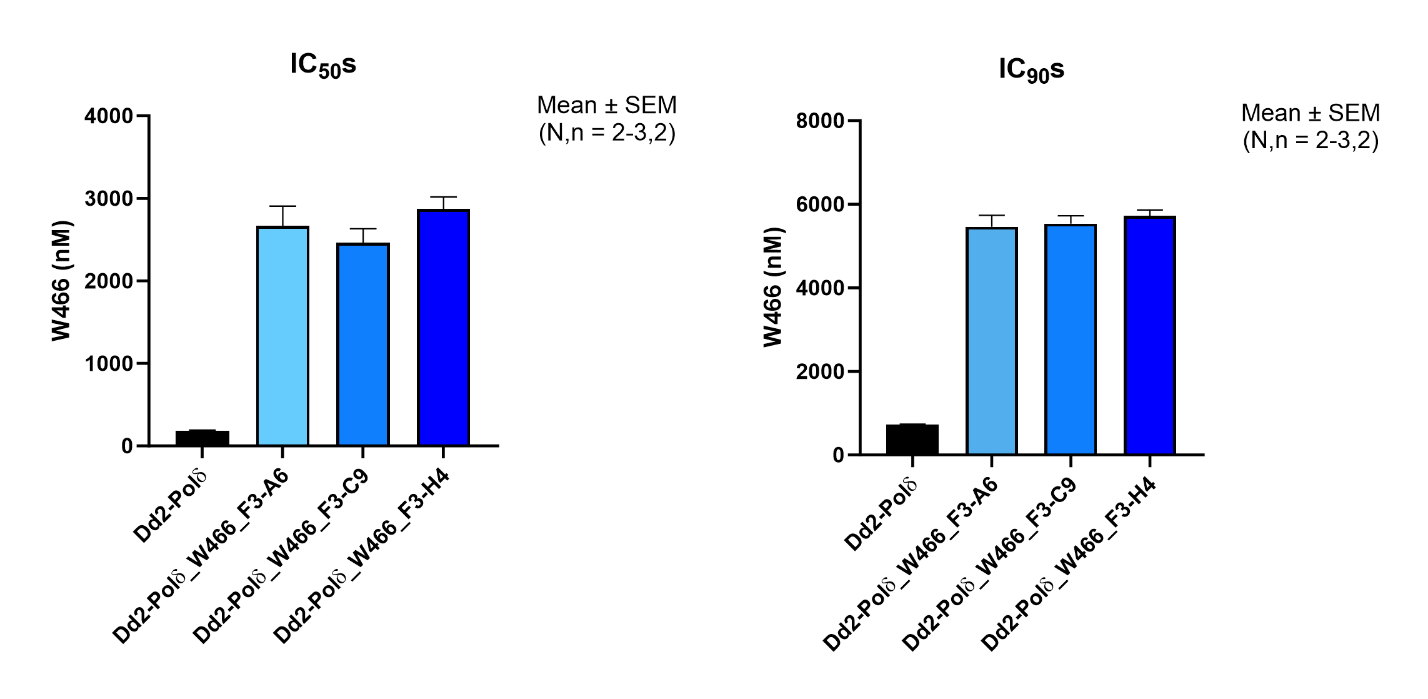


**Figure S8**. IC_50_ and IC_90_ values for Dd2-Polδ W466_F3-resistant parasite lines obtained from the MIR study. Data shown are mean ± SEM (N, n= 2-3, 2).

**Table S11.** IC_50_ and IC_90_ fold-shifts for Dd2-Polδ and W466_F3-resistant parasite lines obtained from the MIR study.

|  | Dd2-Polδ | W466_F3-C9 | W466_F3-A6 | W466_F3-H4 |
| --- | --- | --- | --- | --- |
| IC_50_ (±SEM) nM | 188 (5) | 2461 (13) | 2663 (241) | 2874 (143) |
| IC_50_ fold shift | n/a | 13.1 | 14.2 | 15.2 |
| IC_90_ (±SEM) nM | 732 (2) | 5333 (193) | 5458 (280) | 5726 (137) |
| IC_90_ fold shift | n/a | 7.6 | 7.5 | 30.5 |

^a^ IC_50_ and IC_90_ values represent an average of n=2-3 biological replicates of a 72 h SYBR Green I and MitoTracker Deep Red FM flow cytometry assay.

**Table S12.** Whole genome sequencing metrics for Dd2 W466-resistant H3 population from the MIR study.

|  |  | **Dd2-B2 bulk** | **Parent** |
| --- | --- | --- | --- |
| **Sample names** | | **H3** | **Dd2_B2** |
| **Total reads** | | 4,611,720 | 4,680,824 |
| **# Mapped reads** | | 4,448,707 | 4,342,594 |
| **Duplication rate** | | 48.57% | 31.51% |
| **General error rate** | | 1.56% | 1.71% |
| **Mean mapping quality (Phred)** | | 56.95 | 56.74 |
| **Depth of coverage** | **mean** | 32.739 | 40.85 |
|  | **SD** | 31.6639 | 37.53 |
| **% of PF genome with > x no. reads** | **1X** | 95.79% | 96.07% |
|  | **5X** | 93.88% | 94.20% |
|  | **10X** | 91.31% | 91.81% |
|  | **20X** | 80.51% | 83.57% |
|  | **30X** | 59.23% | 71.42% |

**Table S13.** Homozygous non-synonymous single nucleotide polymorphism identified from the Dd2 W466-resistant H3 genome from the MIR study.

| Chrom | position | Base change | Amino acid change | Codon change | Gene name | Gene description |
| --- | --- | --- | --- | --- | --- | --- |
| MIT | 3882 | G->A | G131S | Ggt/Agt | PF3D7_MIT02300 | cyt *b* |

**Table S14.** Whole genome sequencing metrics for Dd2-Polδ W466-resistant F3 clones from the MIR study.

|  | | **Dd2-Polδ clones** | | | **Parent** |
| --- | --- | --- | --- | --- | --- |
| **Sample names** | | **F3-A6** | **F3-H4** | **F3-C9** | **Dd2-Polδ** |
| **Total reads** | | 3,684,204 | 3,269,997 | 4,689,367 | 4,271,890 |
| **# Mapped reads** | | 3,205,348 | 2,950,618 | 4,331,963 | 3,963,861 |
| **Duplication rate** | | 29.46% | 29.58% | 28.25% | 28.71% |
| **General error rate** | | 1.37% | 1.30% | 1.78% | 1.89% |
| **Mean mapping quality (Phred)** | | 56.63 | 56.67 | 56.55 | 56.54 |
| **Depth of coverage** | **mean** | 29.98 | 27.86 | 38.2554 | 36.1 |
|  | **SD** | 58.48 | 60.62 | 30.7309 | 30.49 |
| **% of PF genome with > x no. reads** | **1X** | 94.85% | 94.18% | 96.14% | 96.07% |
|  | **5X** | 88.34% | 86.64% | 94.31% | 94.29% |
|  | **10X** | 79.98% | 77.44% | 92.08% | 92.43% |
|  | **20X** | 64.37% | 61.36% | 83.55% | 84.94% |
|  | **30X** | 49.46% | 45.66% | 69.47% | 68.06% |

**Table S15.** Non-synonymous single nucleotide polymorphism identified from the Dd2-Polδ W466-resistant F3 clones from the MIR study.

| **Chrom** | **Position** | **Ref** | **Amino acid change** | **Codon change** | **Gene name** |
| --- | --- | --- | --- | --- | --- |
| Pf_M76611 | 4281 | T->C | F264L | Ttt/Ctt | mal_mito_3 (cytochrome b) |
| Pf3D7_03_v3 | 956042 | G->A | S2109F | tCt/tTt | PF3D7_0322700 (conserved Plasmodium protein, unknown function) |
| Pf3D7_05_v3 | 416423 | G->T | R20I | aGa/aTa | PF3D7_0509900 (conserved protein, unknown function) |
| Pf3D7_06_v3 | 311794 | G->A | R82H | cGt/cAt | PF3D7_0607500 (4-hydroxybenzoate polyprenyltransferase, putative) |
| Pf3D7_06_v3 | 1138076 | G->T | Q5197K | Caa/Aaa | PF3D7_0628100 (HECT-domain (ubiquitin-transferase), putative) |
| Pf3D7_09_v3 | 295131 | G->A | S496F | tCt/tTt | PF3D7_0905900 (coatomer subunit beta, putative) |
| Pf3D7_10_v3 | 513984 | G->T | M967I | atG/atT | PF3D7_1013200 (conserved Plasmodium protein, unknown function) |
| Pf3D7_10_v3 | 858673 | G->A | S357F | tCt/tTt | PF3D7_1021200 (conserved Plasmodium protein, unknown function) |
| Pf3D7_10_v3 | 957902 | C->T | L674F | Ctt/Ttt | PF3D7_1022800 (4-hydroxy-3-methylbut-2-en-1-yl diphosphate synthase (ferredoxin), putative) |
| Pf3D7_11_v3 | 269138 | A->T | F1762Y | tTt/tAt | PF3D7_1106500 (conserved Plasmodium protein, unknown function) |

**Table S16.** Summary of homozygous SNPs observed from whole-genome sequencing of respective Dd2-Polδ W466-resistant F3_A6, F3_H4 and F3-C9 clones from the MIR study.

| **Gene name** | **Amino acid change** | **Codon change** | **Dd2-Polδ clones** | | |
| --- | --- | --- | --- | --- | --- |
|  |  |  | **F3-C9** | **F3-A6** | **F3-H4** |
| PF3D7_MIT02300 | F264L | Ttt/Ctt | 100% | 100% | 100% |
| PF3D7_0322700 | S2109F | tCt/tTt | 0% | 100% | 0% |
| PF3D7_0509900 | R20I | aGa/aTa | 100% | 0% | 0% |
| PF3D7_0607500 | R82H | cGt/cAt | 0% | 100% | 0% |
| PF3D7_0628100 | Q5197K | Caa/Aaa | 0% | 100% | 100% |
| PF3D7_0905900 | S496F | tCt/tTt | 100% | 100% | 100% |
| PF3D7_1013200 | M967I | atG/atT | 100% | 0% | 0% |
| PF3D7_1021200 | S357F | tCt/tTt | 100% | 100% | 100% |
| PF3D7_1022800 | L674F | Ctt/Ttt | 0% | 100% | 0% |
| PF3D7_1106500 | F1762Y | tTt/tAt | 0% | 0% | 100% |


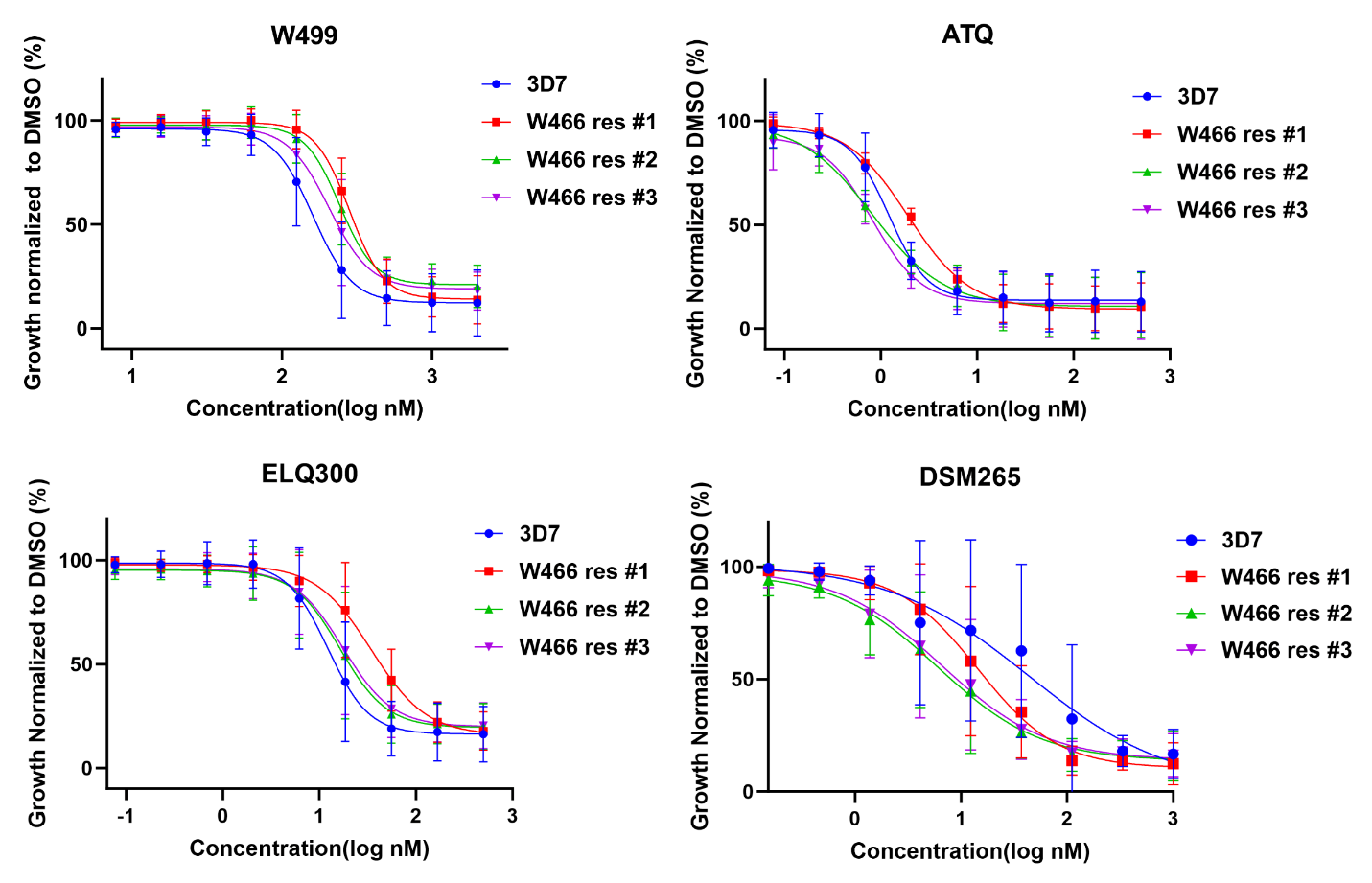


**Figure S9.** Dose response curves of W499 (**2**), DSM-265, ATQ and ELQ300 against W466**-** resistant populations (population 1 has a A122T cyt *b* Q_o_ site mutation and populations 2 and 3 have a F264L cyt *b* Q_o_ site mutation). EC_50_ values represent an average of 3 experiments using the LDH assay. SYBR-green was used for analysis of DSM265. Error bars are SD.


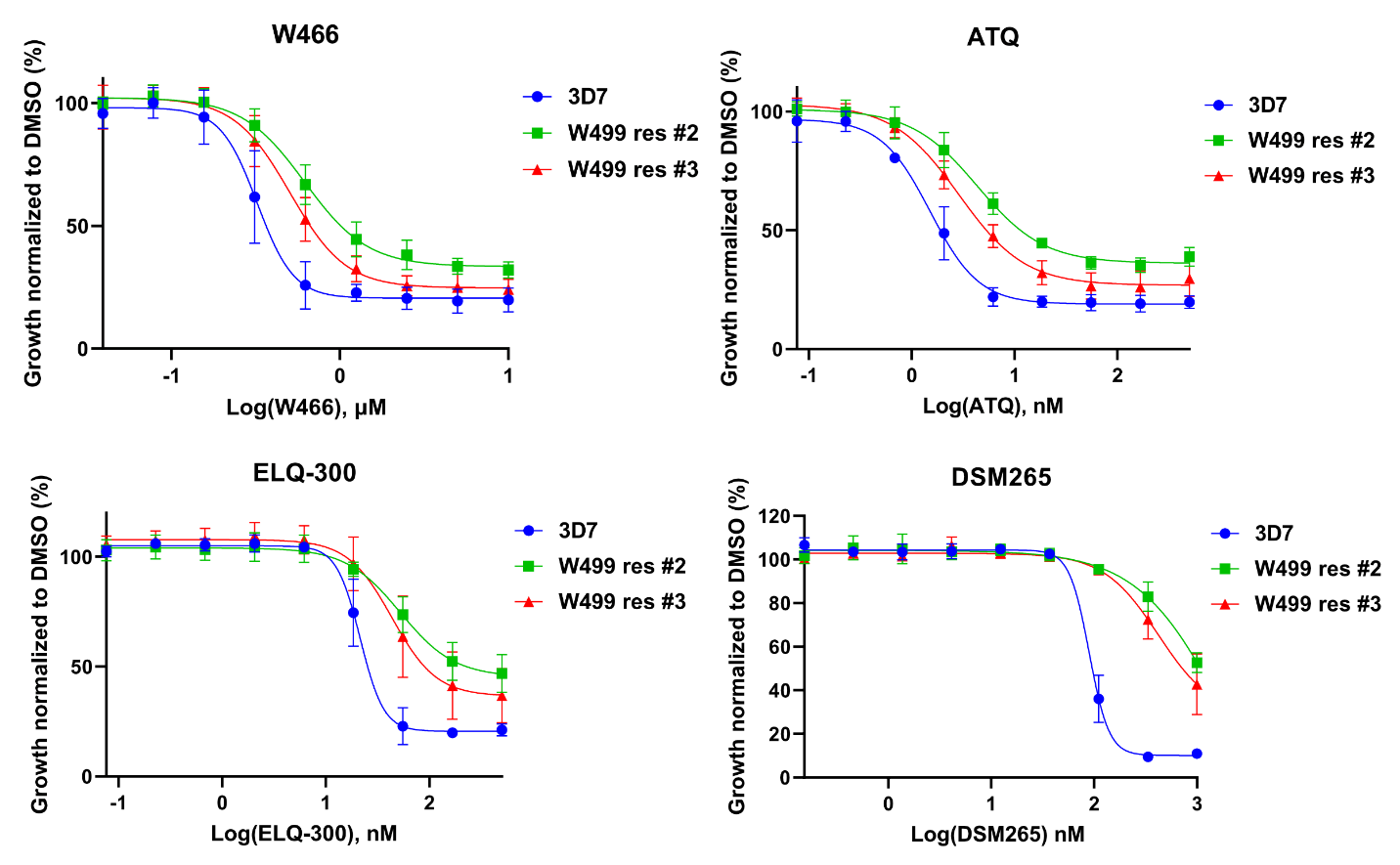


**Figure S10.** Dose response curves of W466 (**1**), DSM-265, ATQ and ELQ300 against W499**-** resistant populations that have a 2 to 5-fold CNV in region of the genome that encodes DHODH. EC_50_ values represent an average of 3 experiments using the LDH assay. SYBR-green was used for analysis of DSM265. Error bars are SD.

**
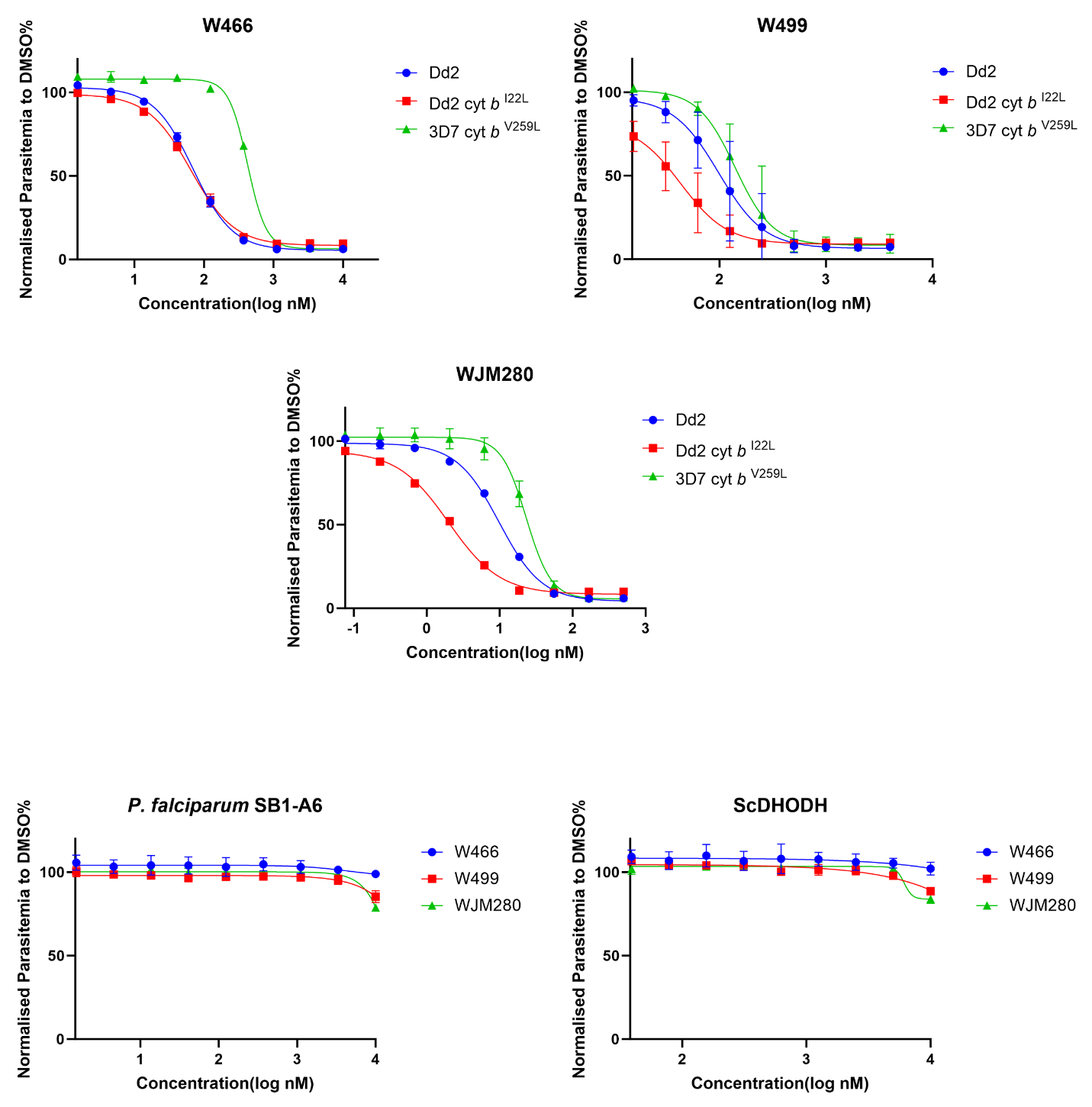
**

**Figure S11**. Dose response curves represent averages and SDs of 3 independent experiments against the Pf SB1-A6 strain with a CNV (~2-fold) and a C276F mutation in DHODH or Pf Dd2 expressing ScDHODH over 72 h measuring SYBR green by flow cytometry.

**
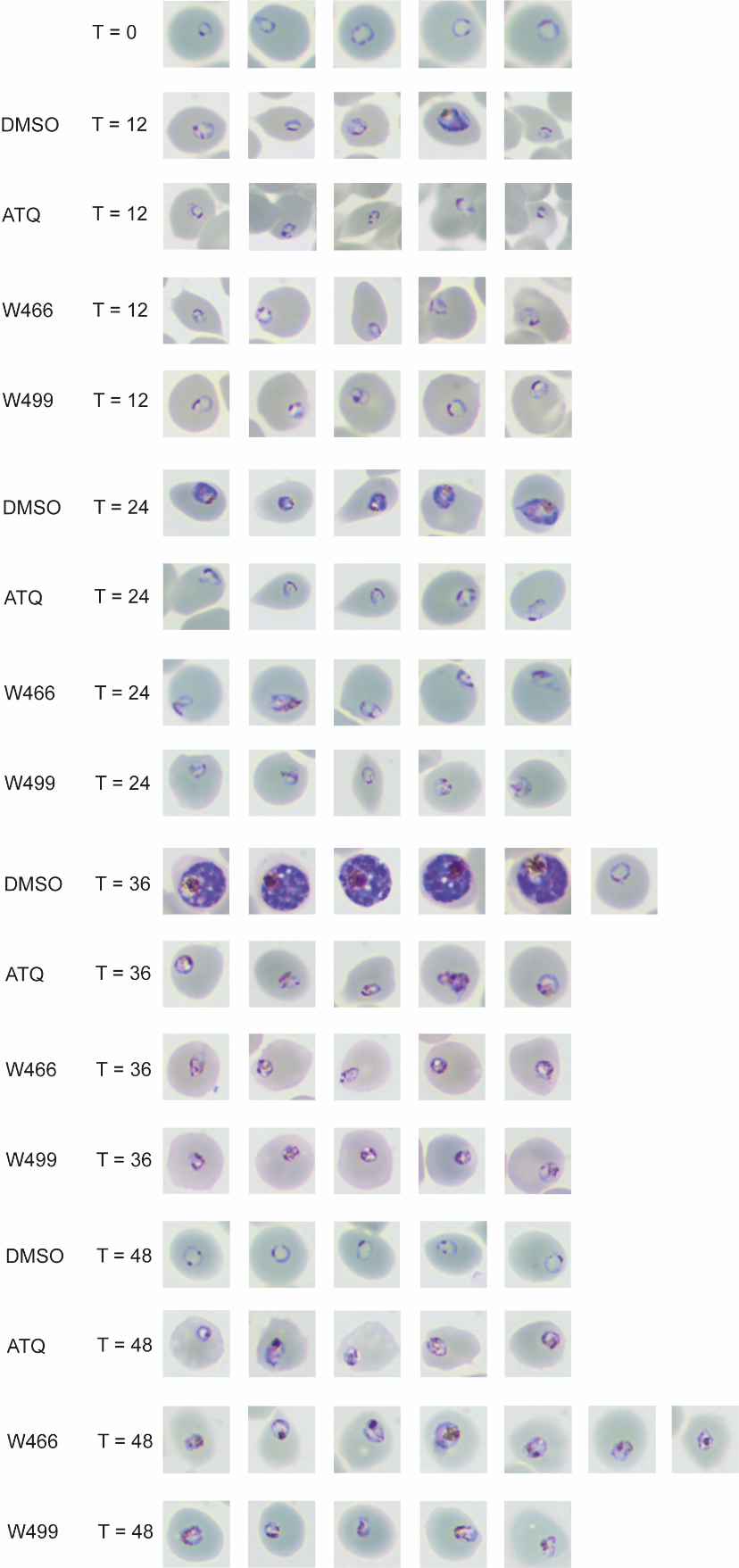
**

**Figure S12.** Stage of asexual arrest of W466 (**1**), W499 (**2**) and ATQ. Microscopy images are representative of parasite morphology determined by Giemsa-stained blood smears at 10 x EC_50_ of each compound after treatment of ring-stage parasites.


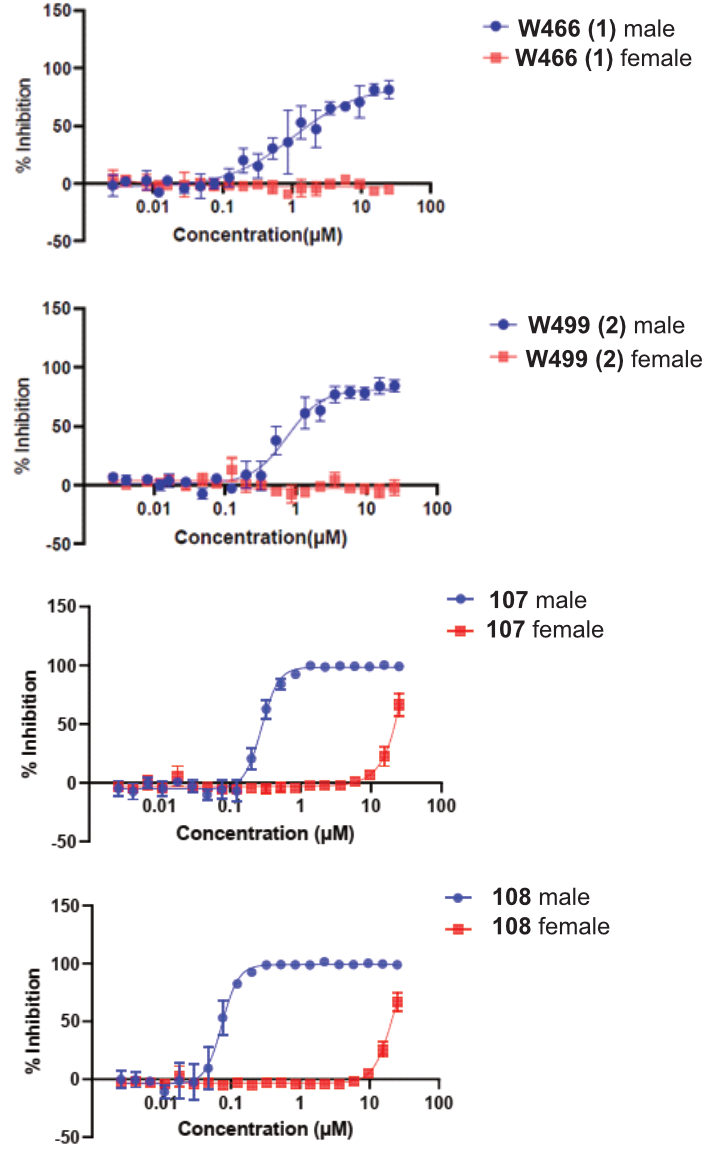


**Figure S13.** Dose response curves of selected compounds against P. falciparum NF54 male and female gametocytes. EC_50_ data represent means and SDs for four replicate experiments. Gametocytes were exposed to the compounds for 48 h before gametogenesis was triggered. The ability to form male gametocytes was assessed 30 min later and female gametes 24 h later. The male gametocyte viability was quantified by automated microscopy measuring exflagellation and for female gametocyte viability measured by fluorescence microscopy using a Cy3-conjugated αPfs25 antibody.

**
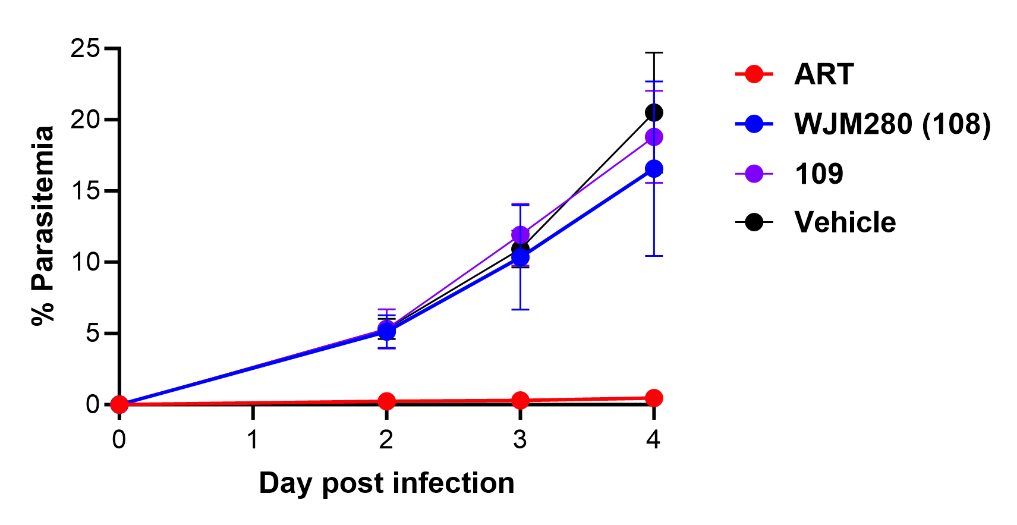
**

**
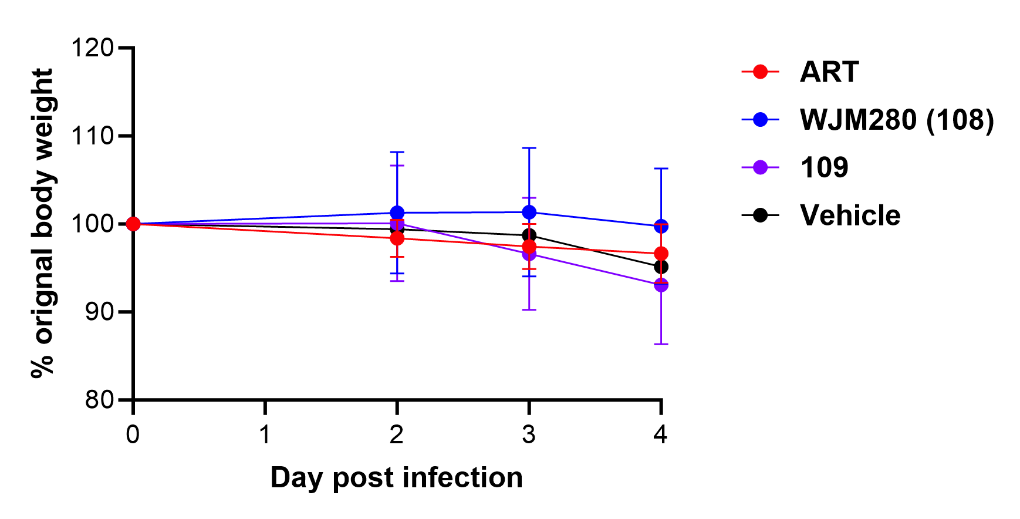
**

**Figure S14.** Evaluation of **17** in a *P. berghei* 4 day mouse model. *P. berghei* ANKA parasites were injected into the tail vein to infect mice on day 0. Compounds WJM280 (**108**) and **109** were administered 50 mg/kg q.d. by oral gavage 2 h after infection (day 0) and then on days 1, 2, and 3. ART = artemisinin; was dosed at 30 mg/kg. Parasitemia of blood samples was measured by microscopy on days 2, 3 and 4 (top panel). Data for WJM280 (**108**) and **109** compared to the vehicle was not statistically significant. Body weight of mice in the *P. berghei* mouse model (bottom panel).

**LC chromatograms, ^1^H-NMR and ^13^C-NMR Spectra of Final Compounds**

Compound **17**

**
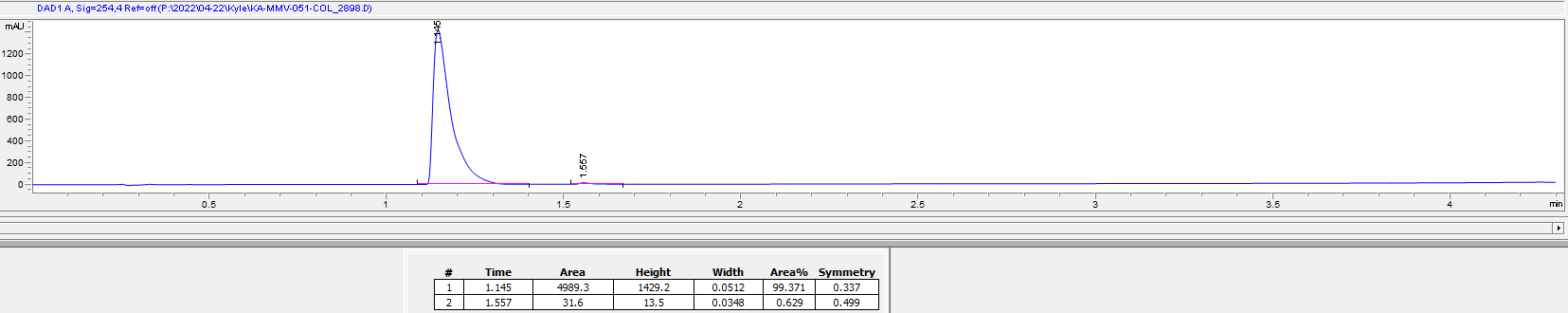
**

**
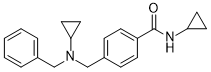

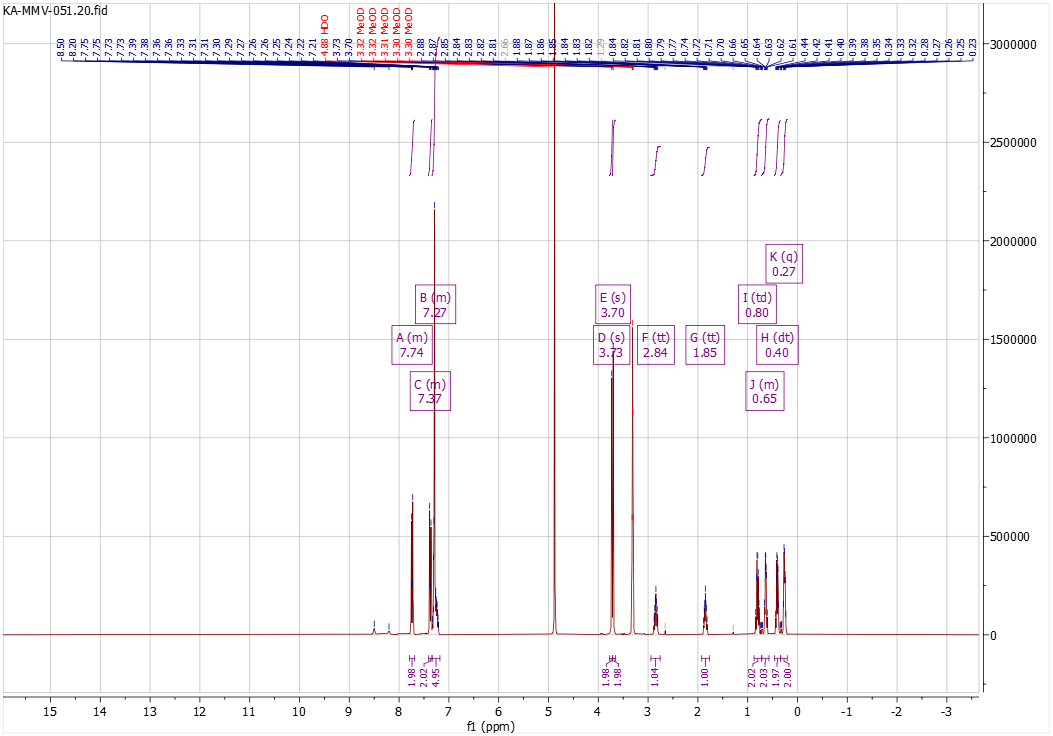
**


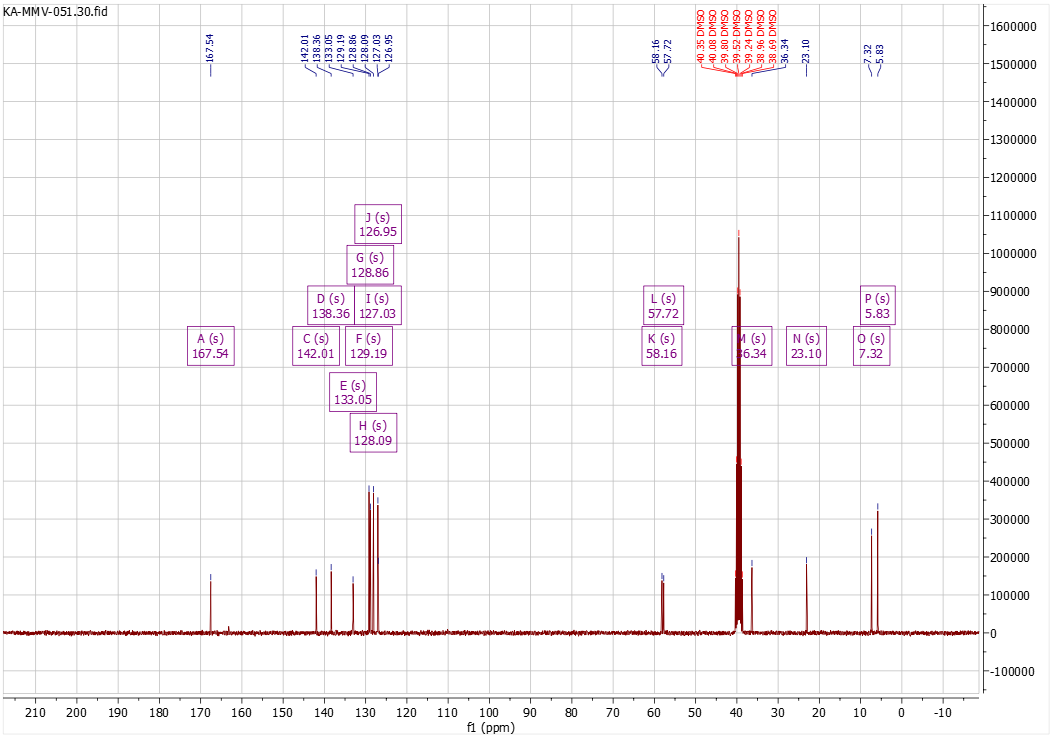


Compound **18**

**
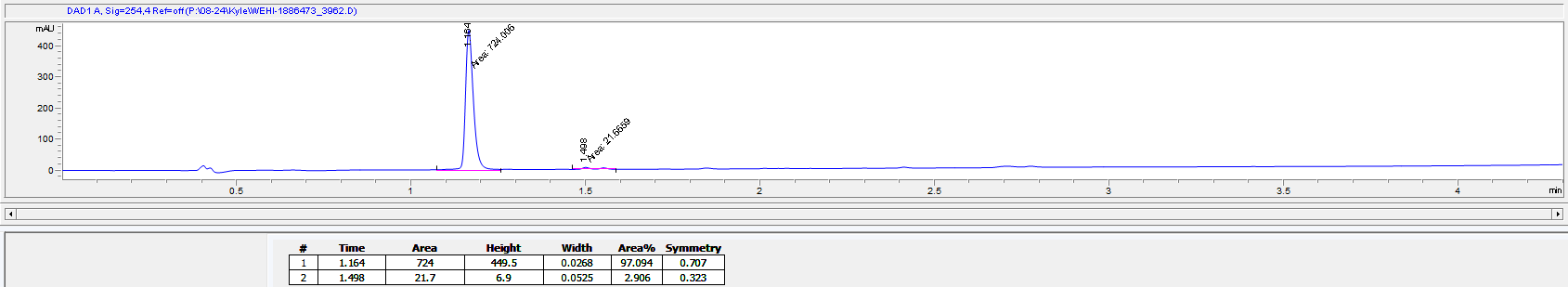
**


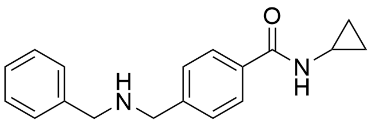

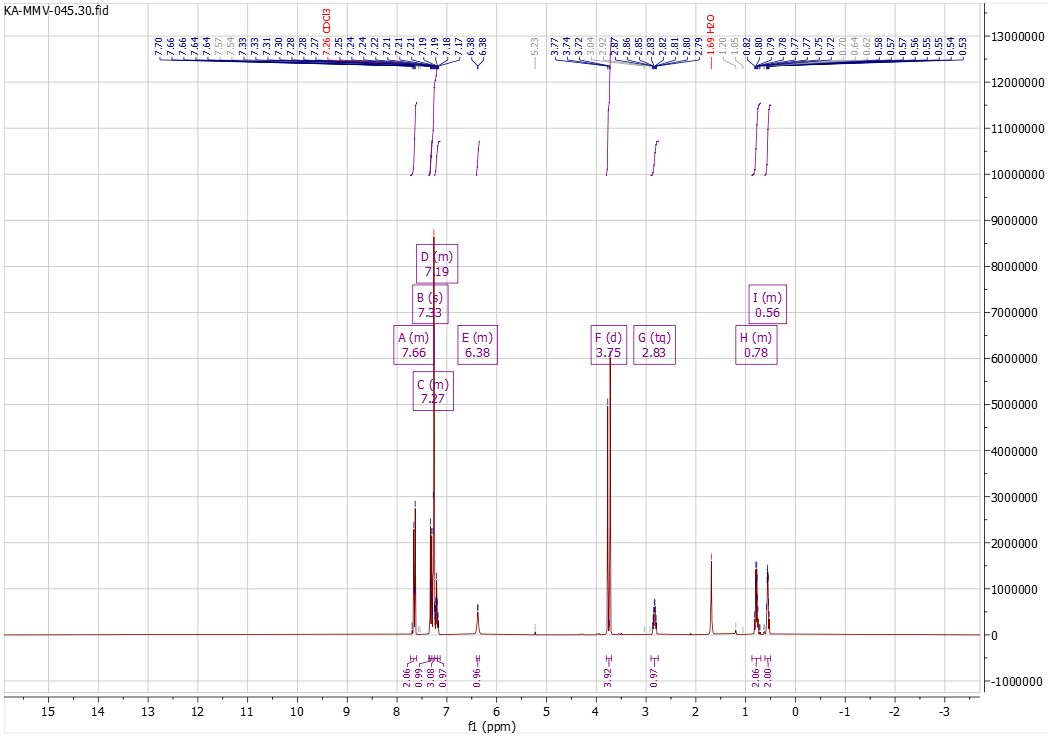


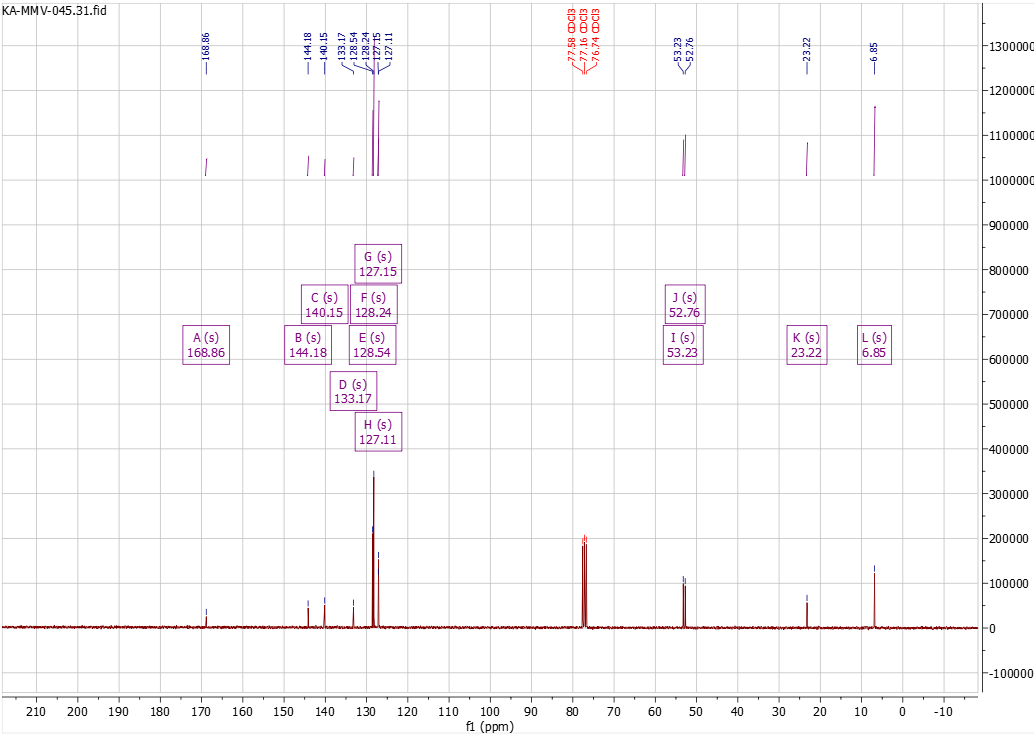


Compound **19**

**
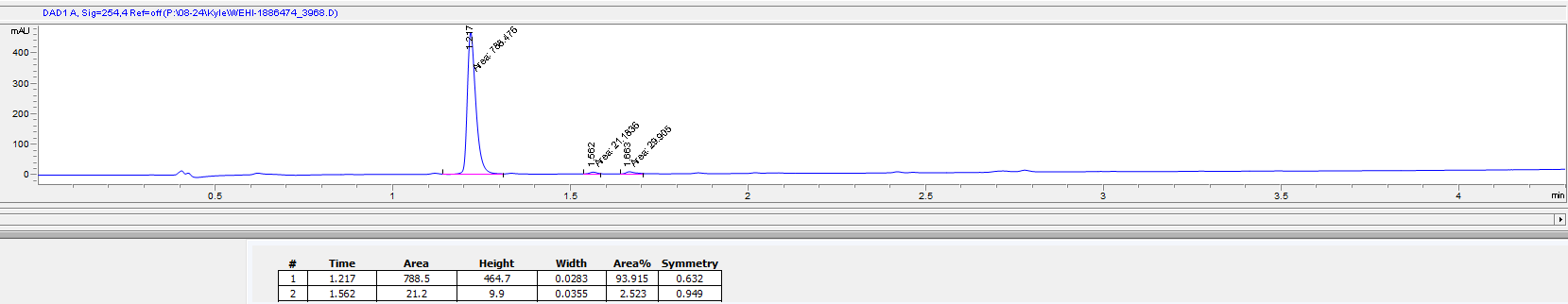
**


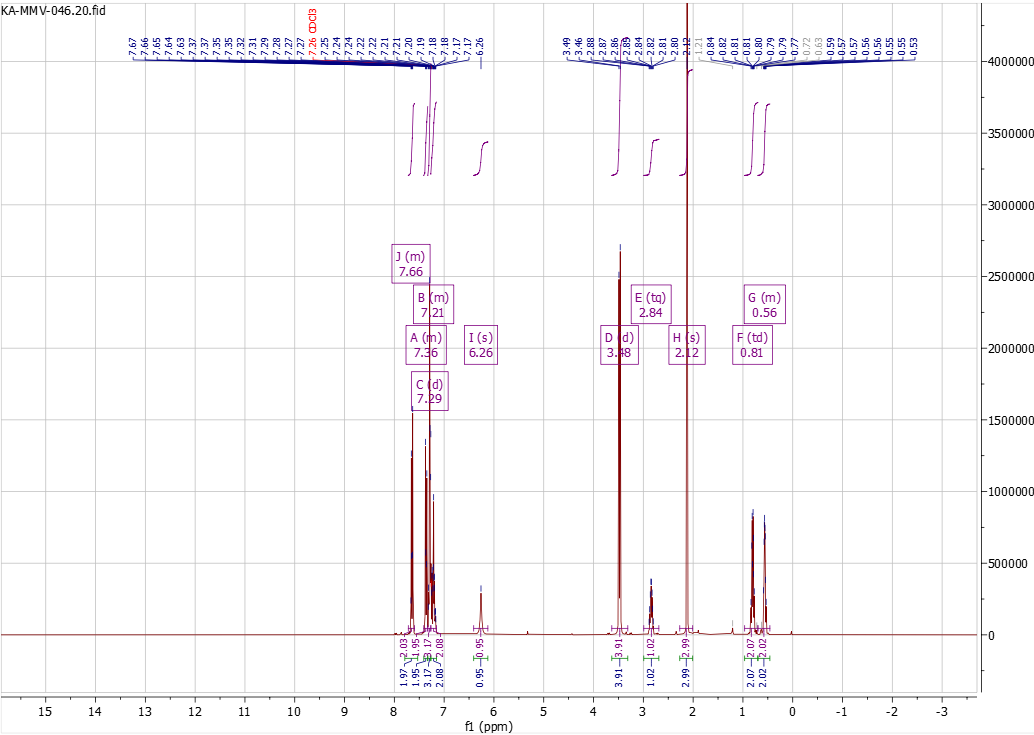

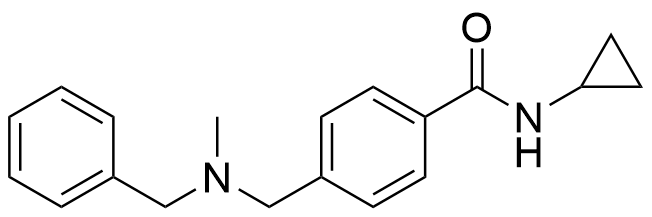


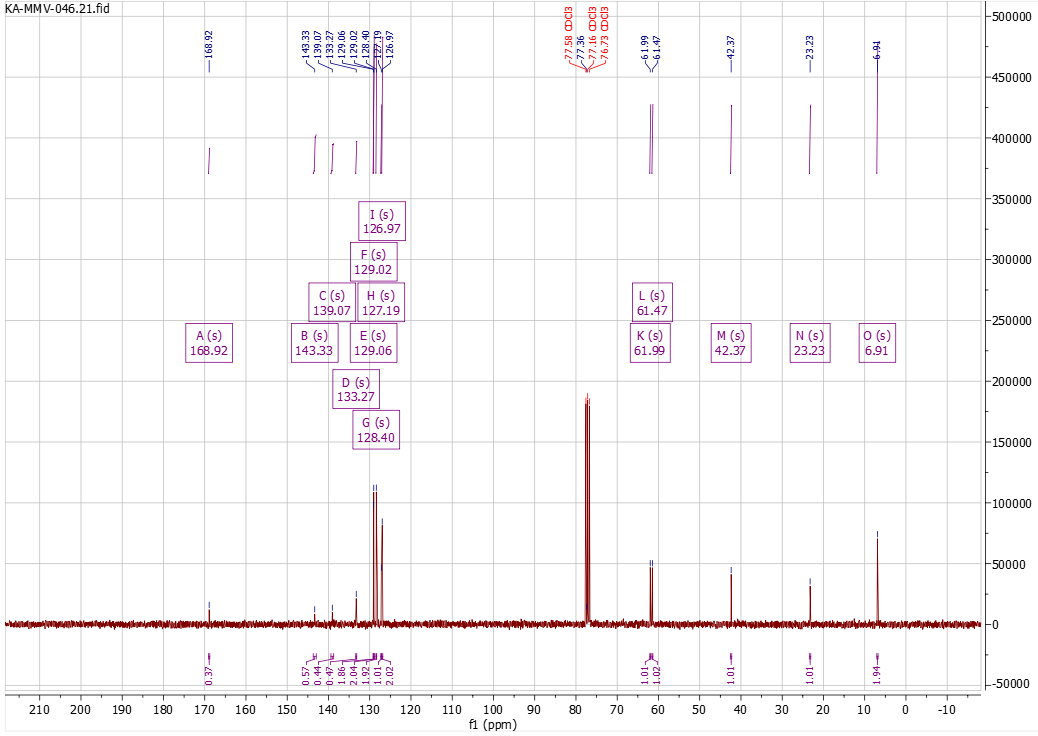
Compound **21**


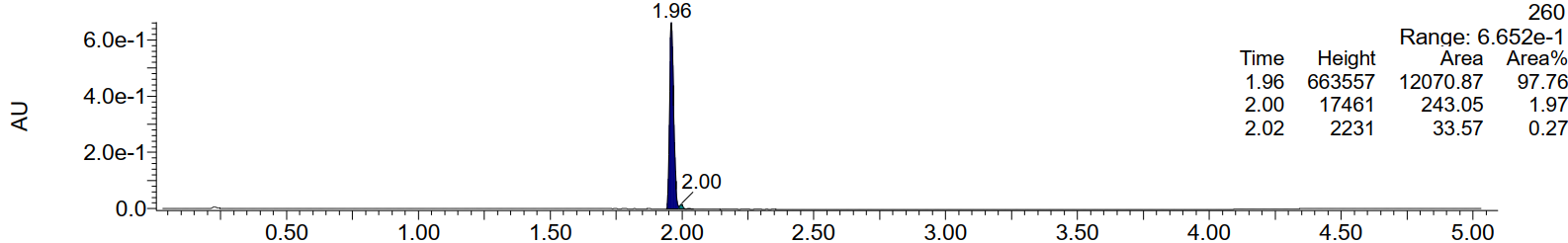


**
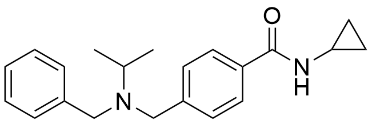
**
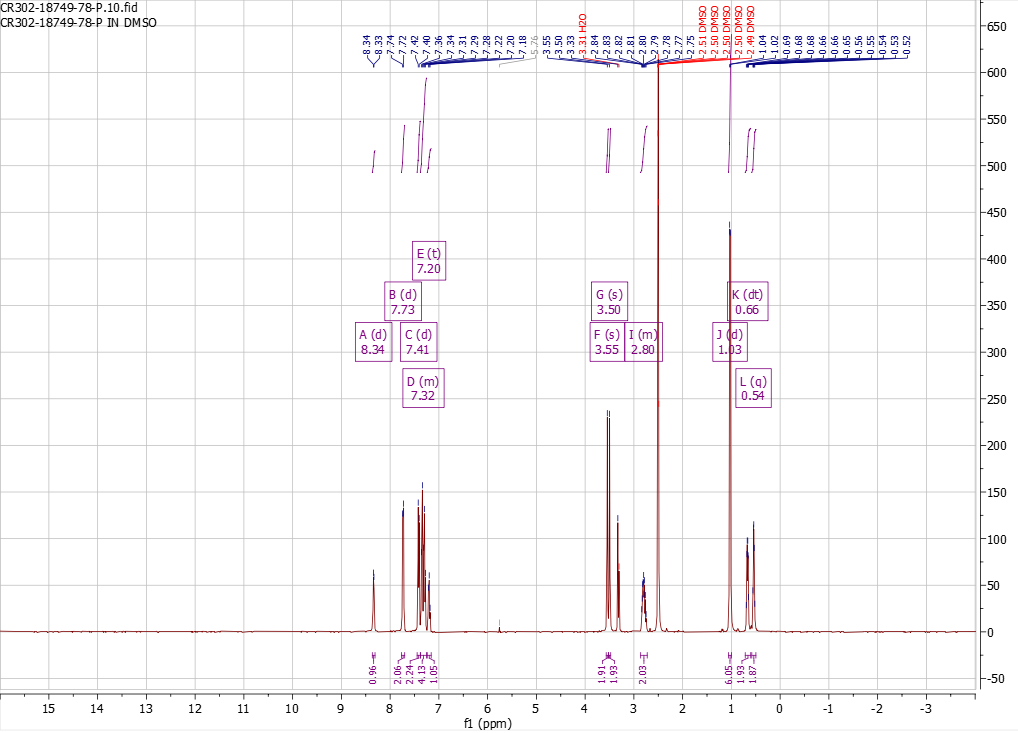


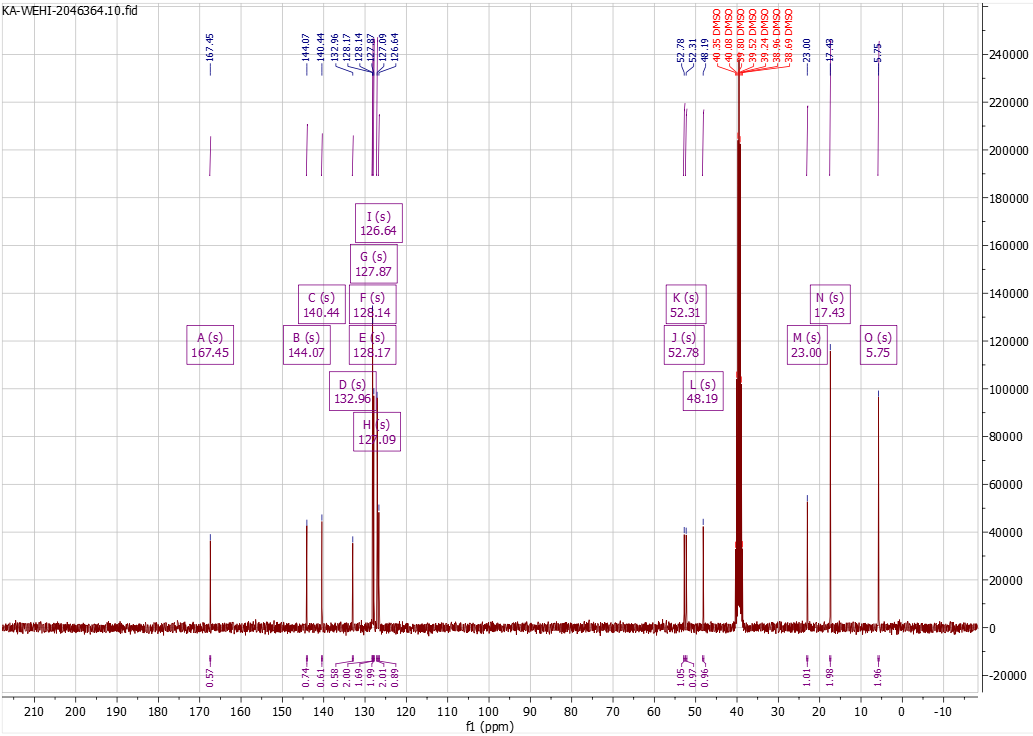


Compound **22**

**
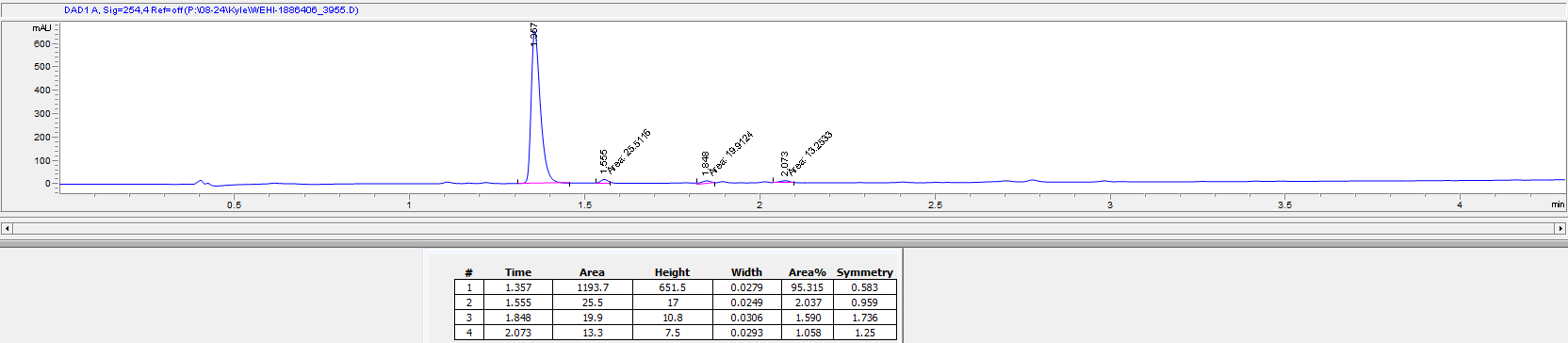
**

**
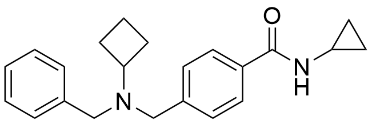
**
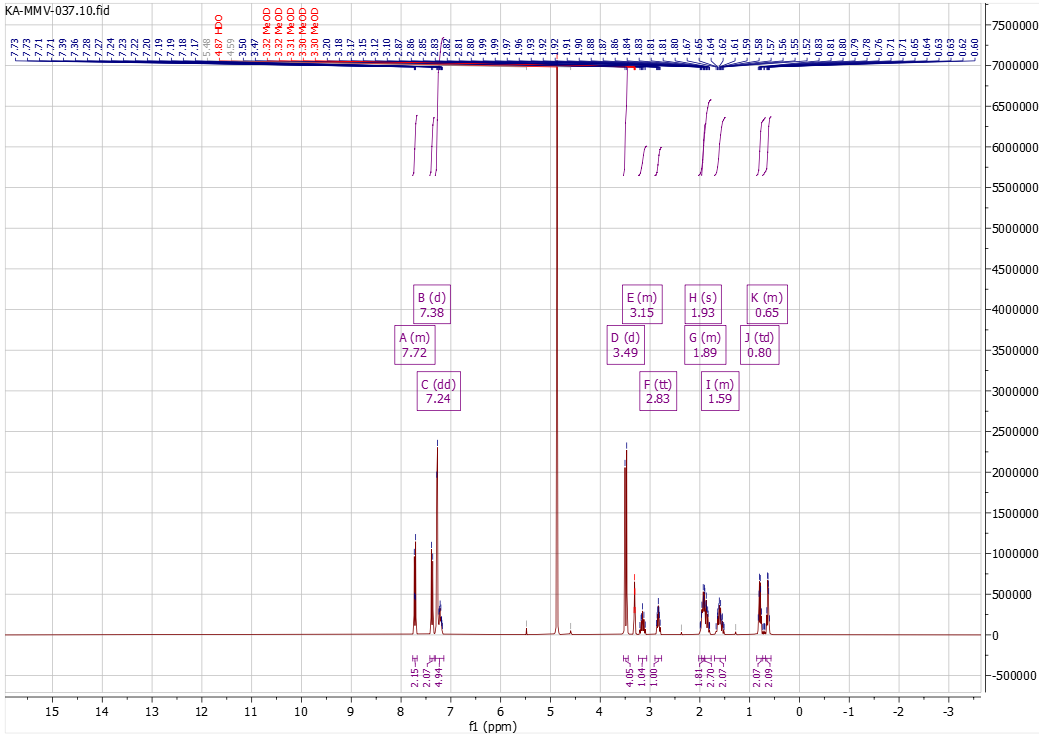


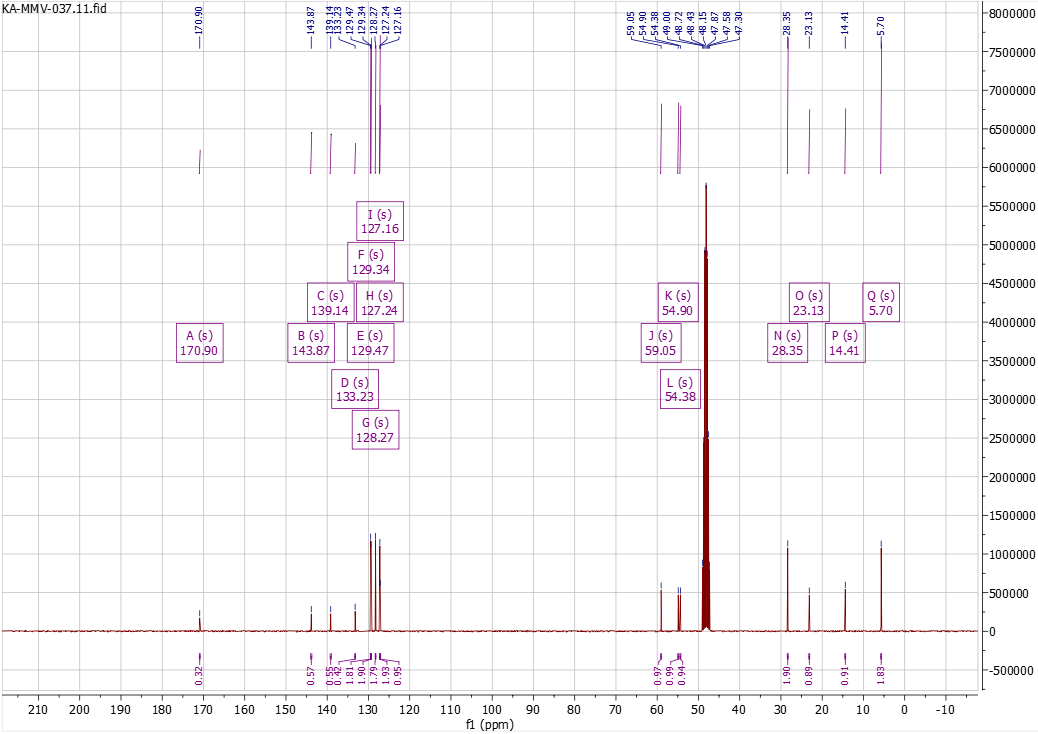


Compound **23**

**
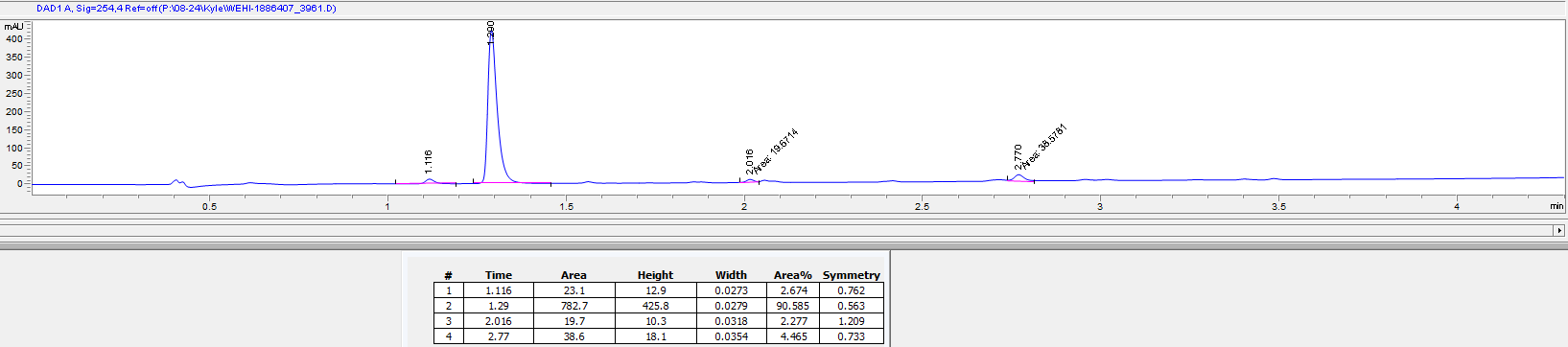
**

**
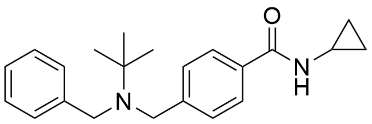
**
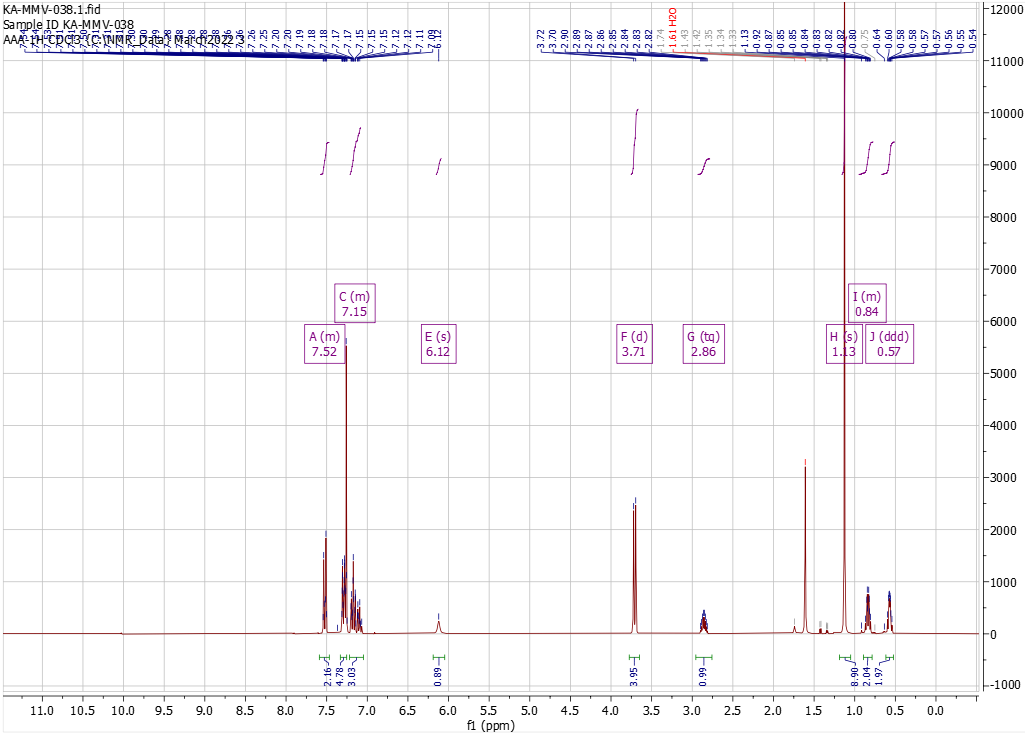


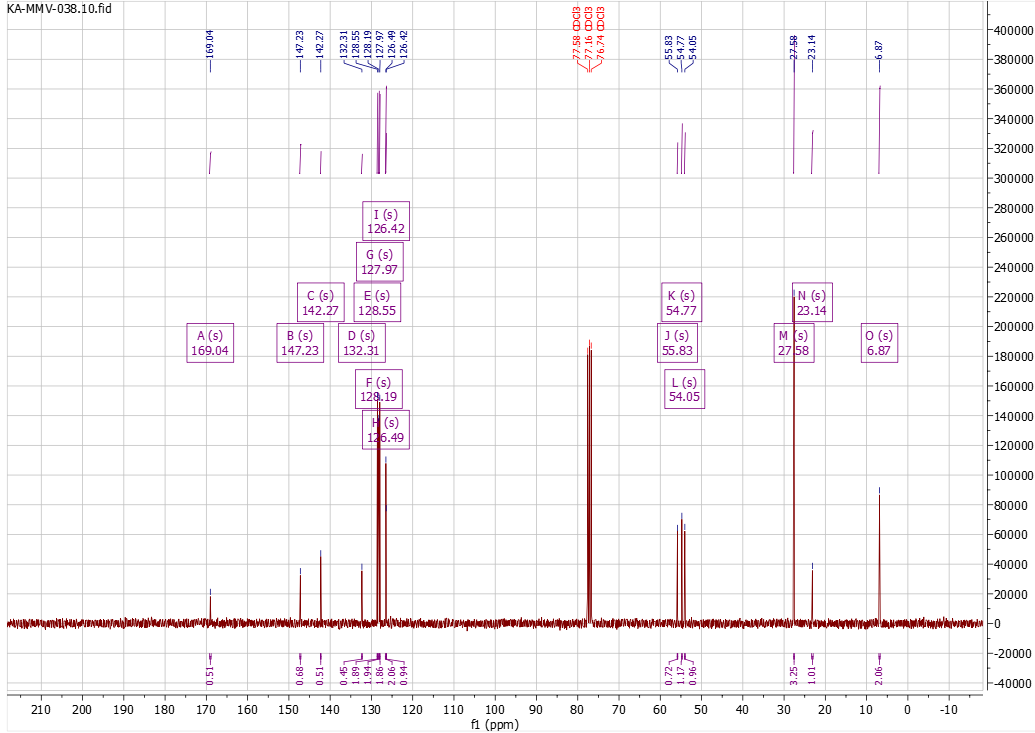


Compound **24**

**
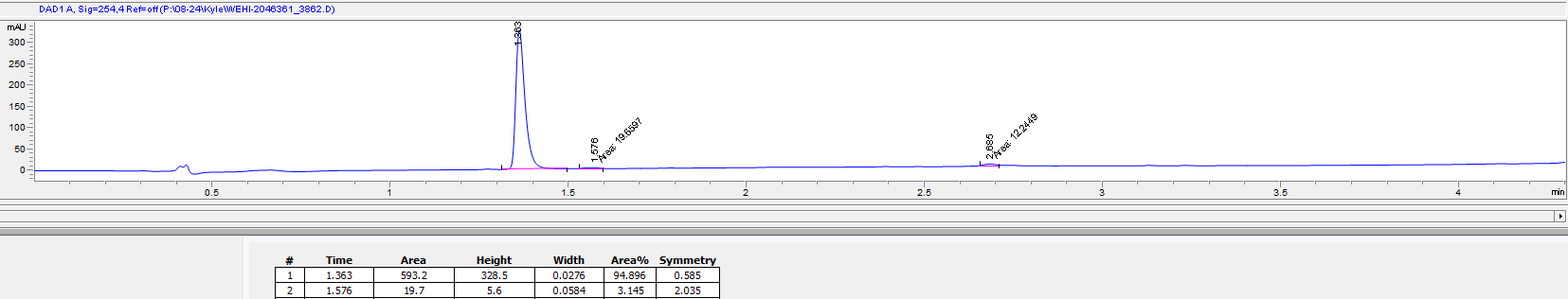
**

**
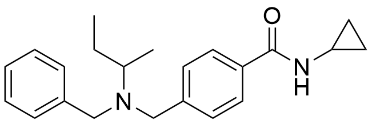
**
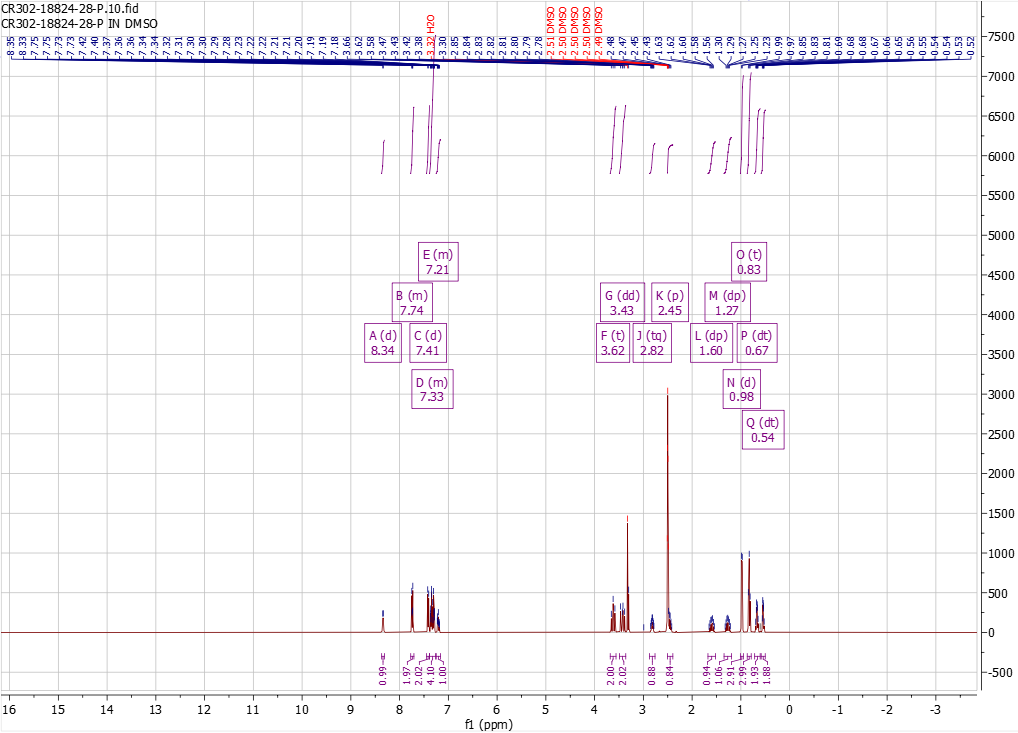


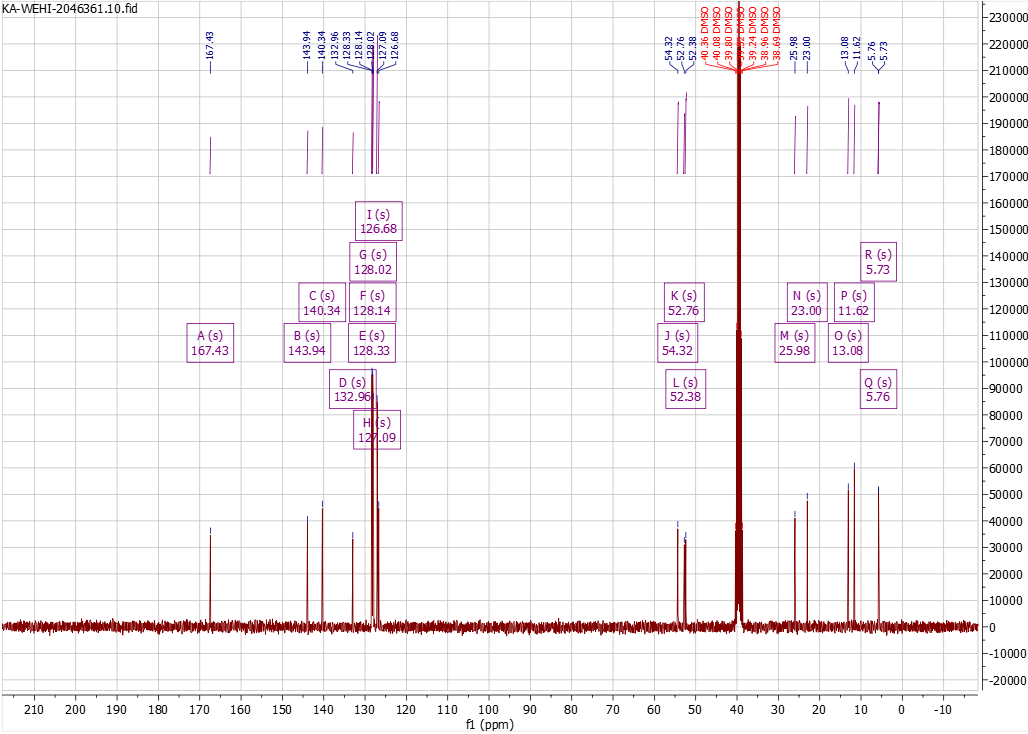


Compound **25**

**
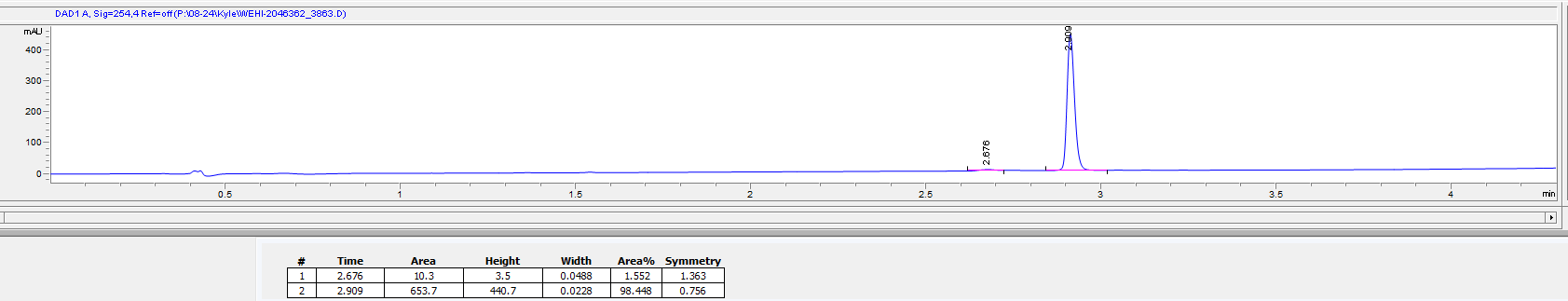
**

**
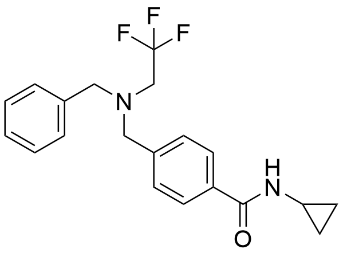
**
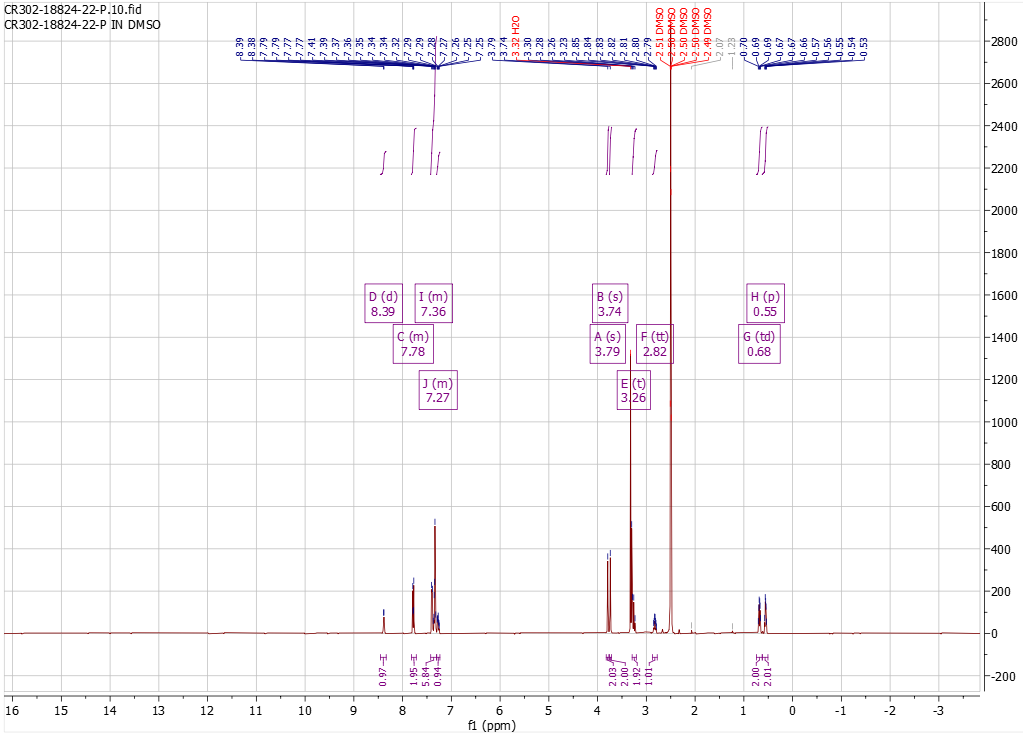


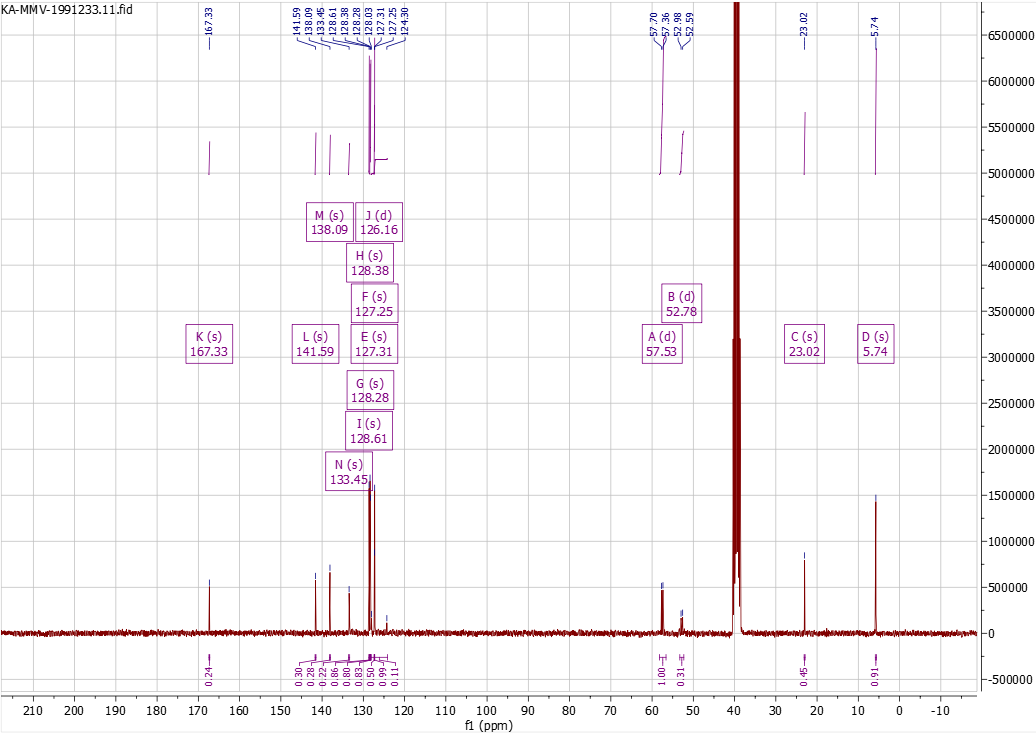


Compound **26**

**
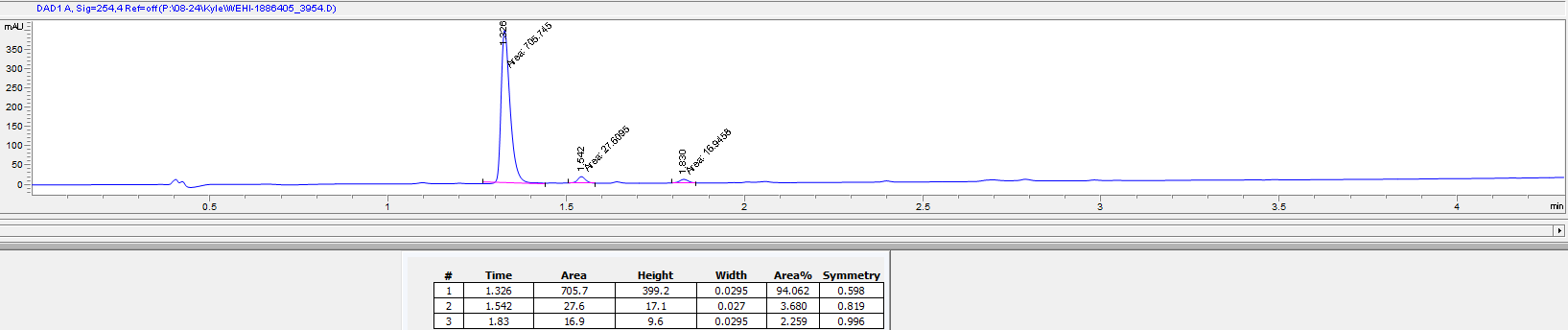
**

**
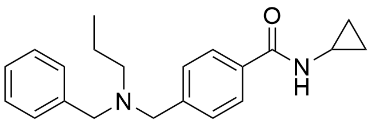
**
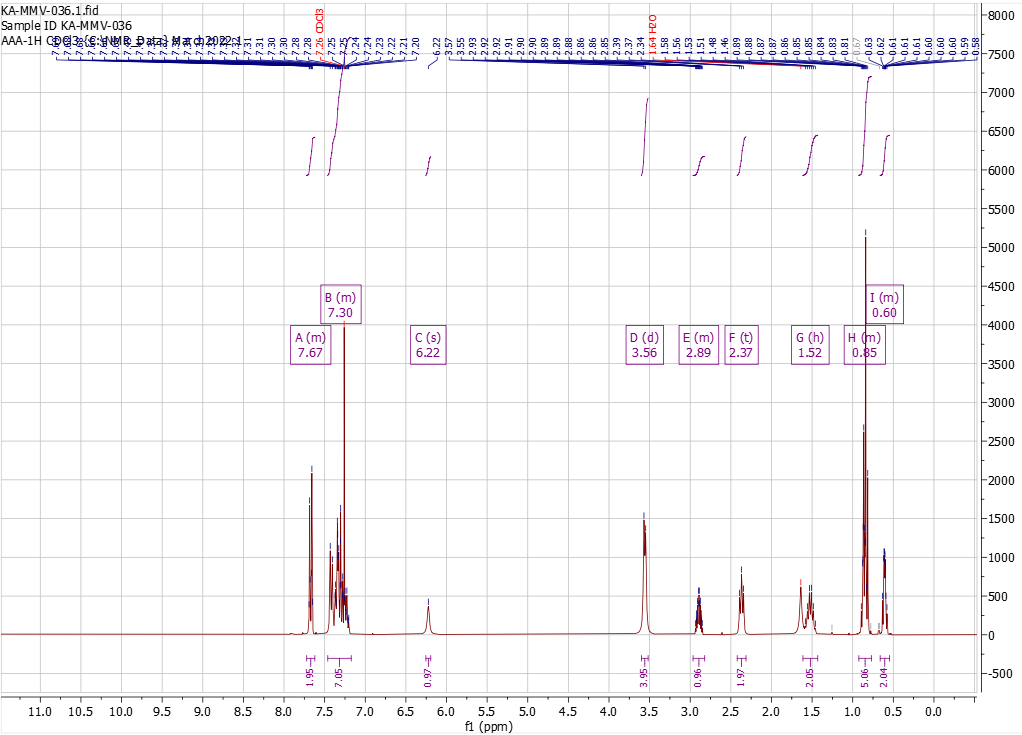


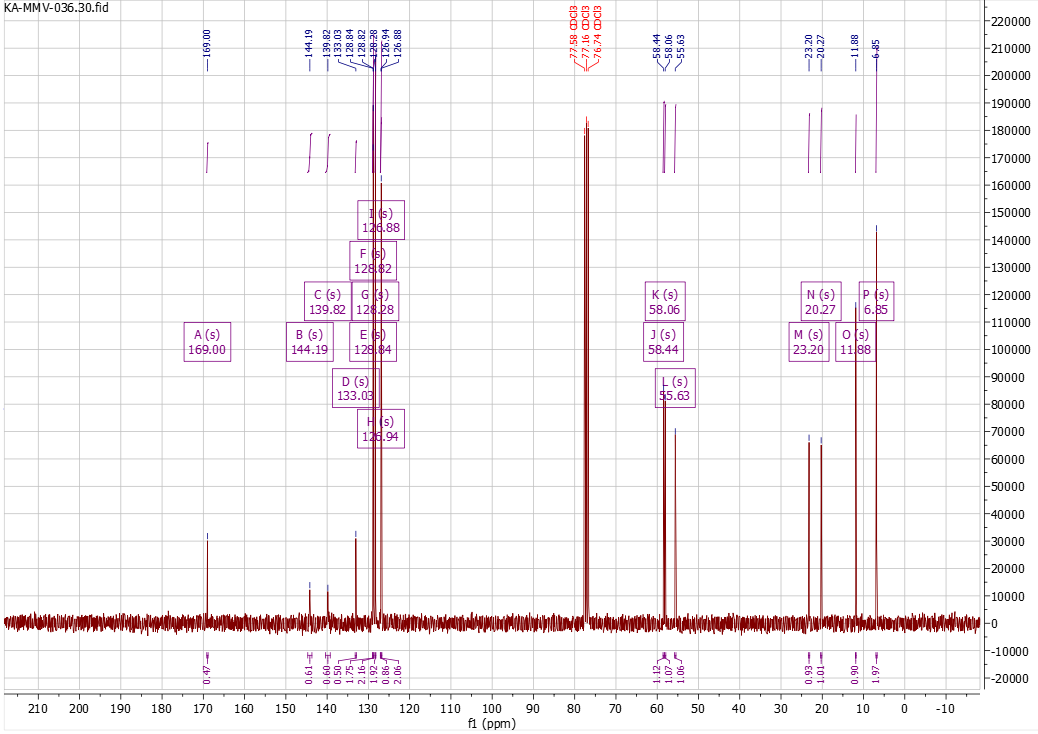


Compound **27**


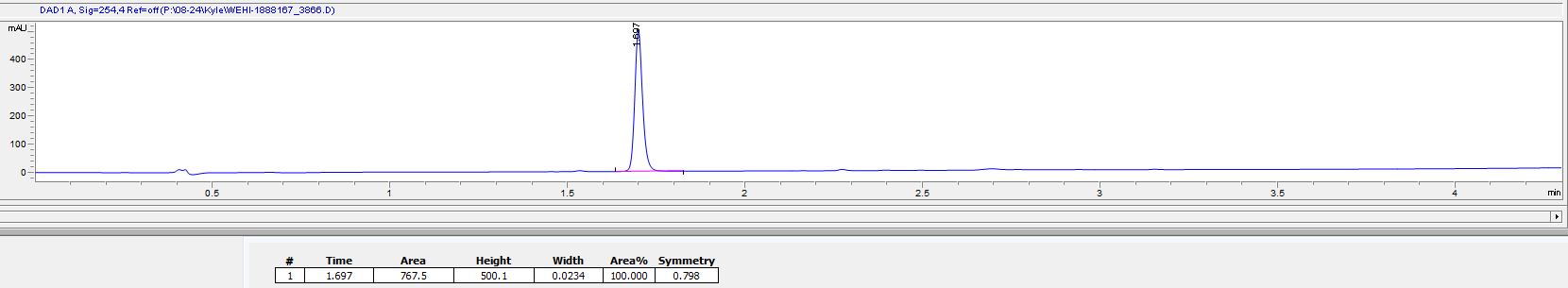


**
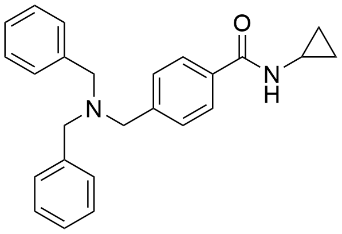
**
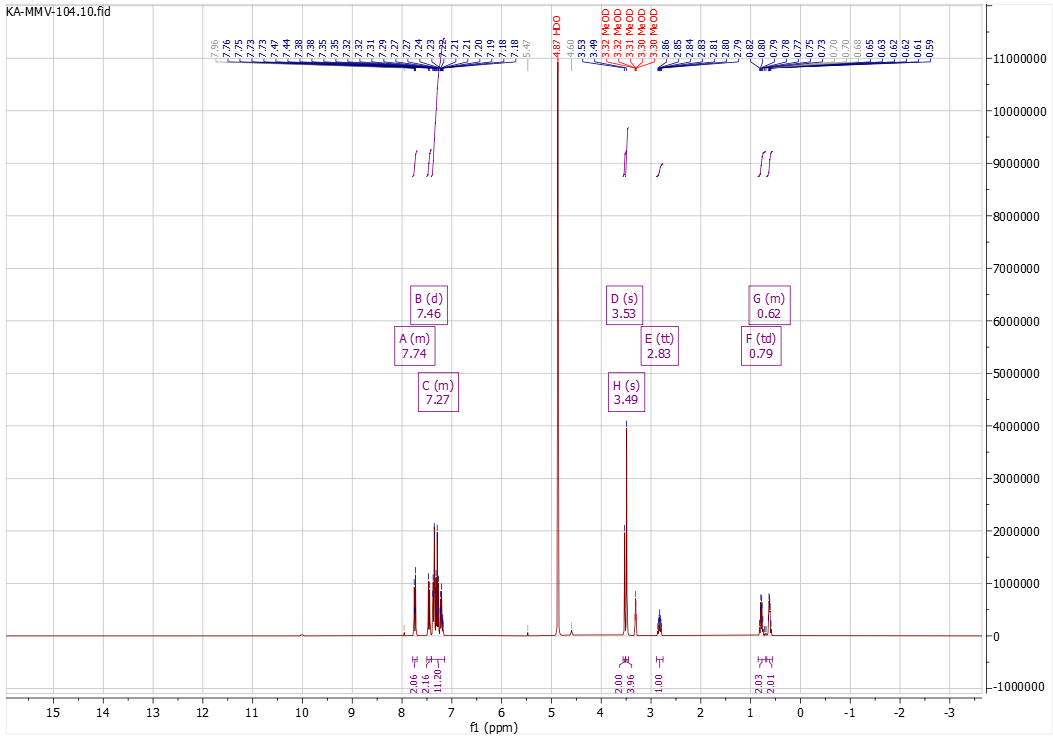


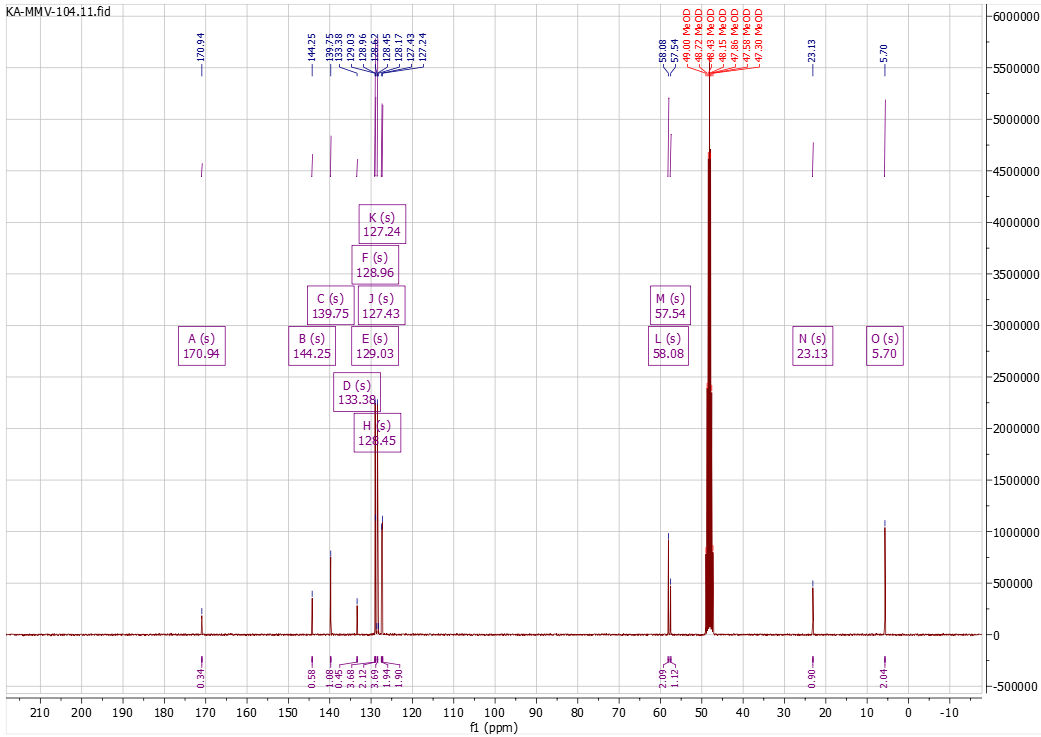


Compound **28**


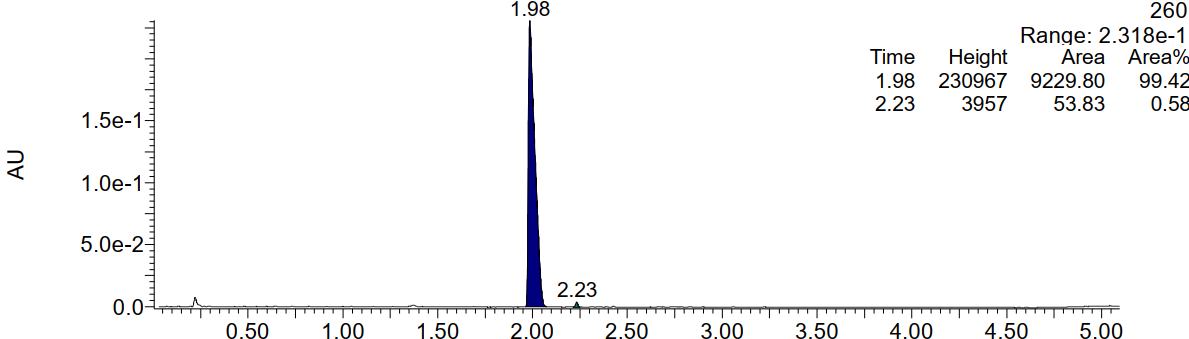


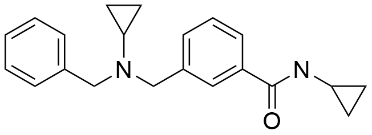

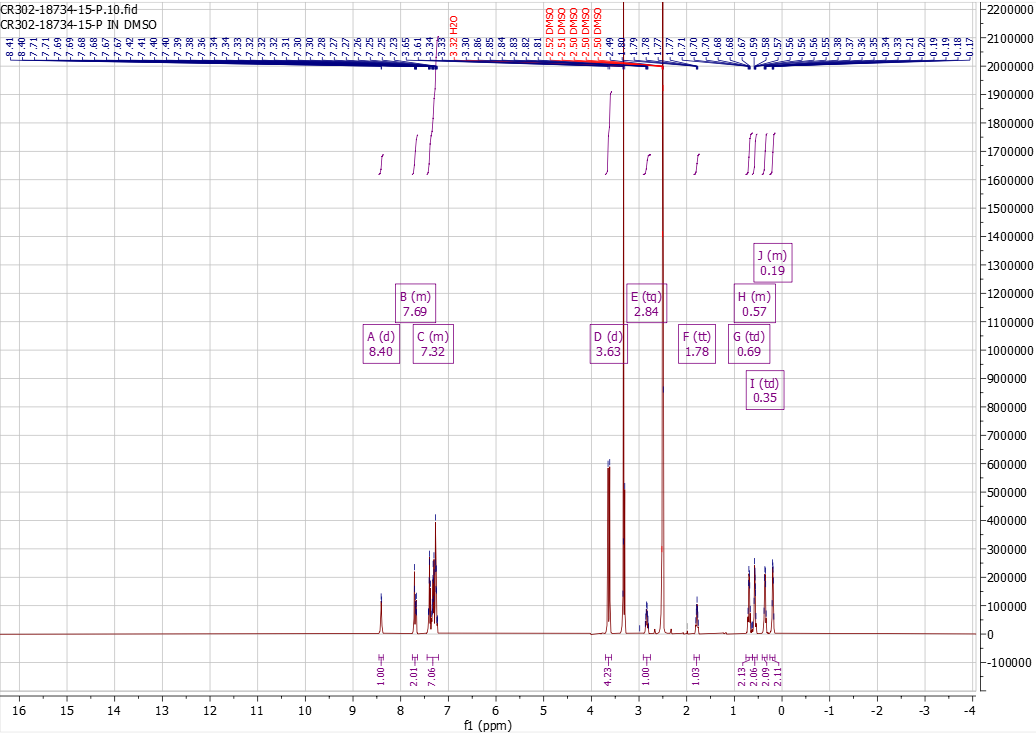


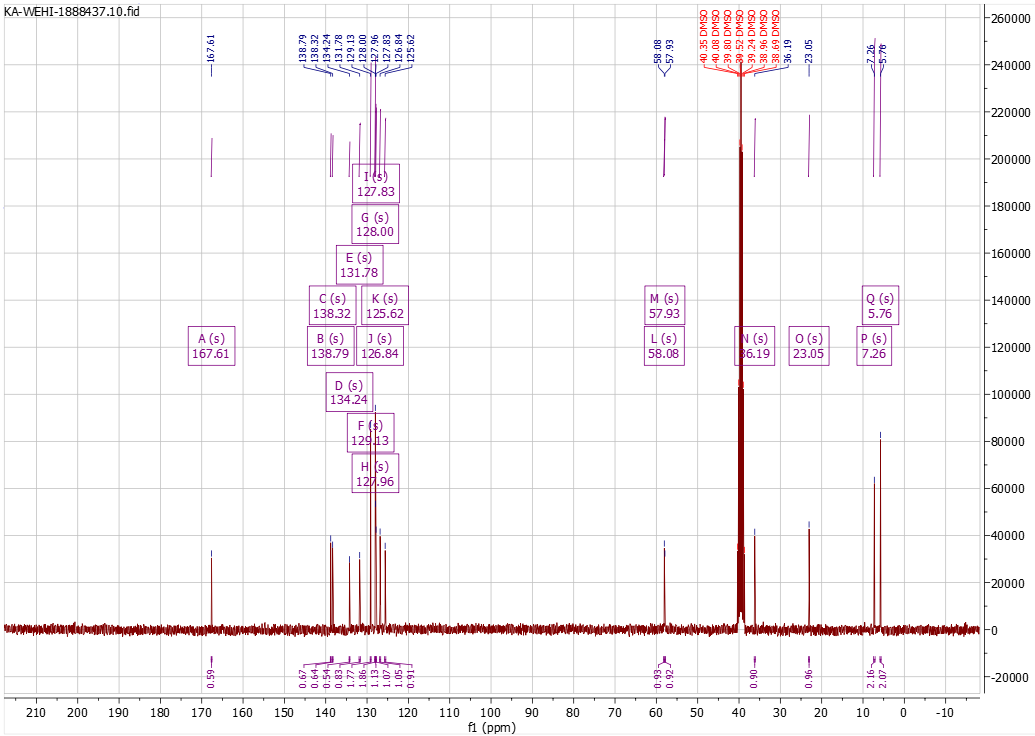


Compound **31**


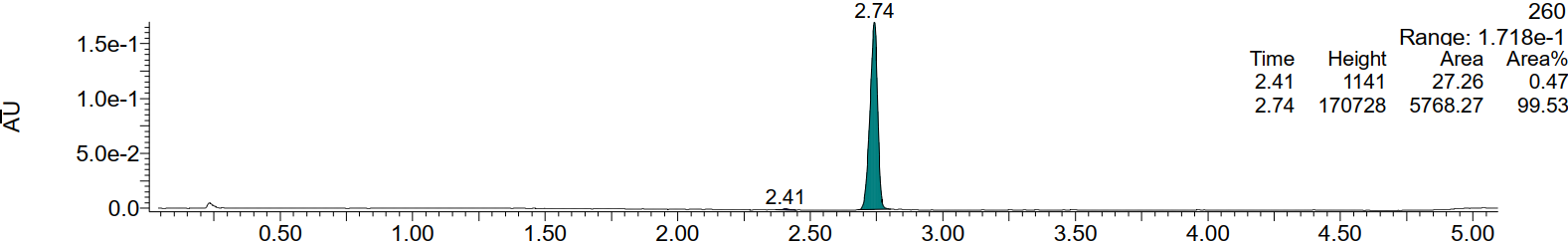


**
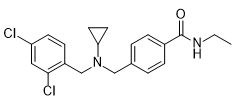

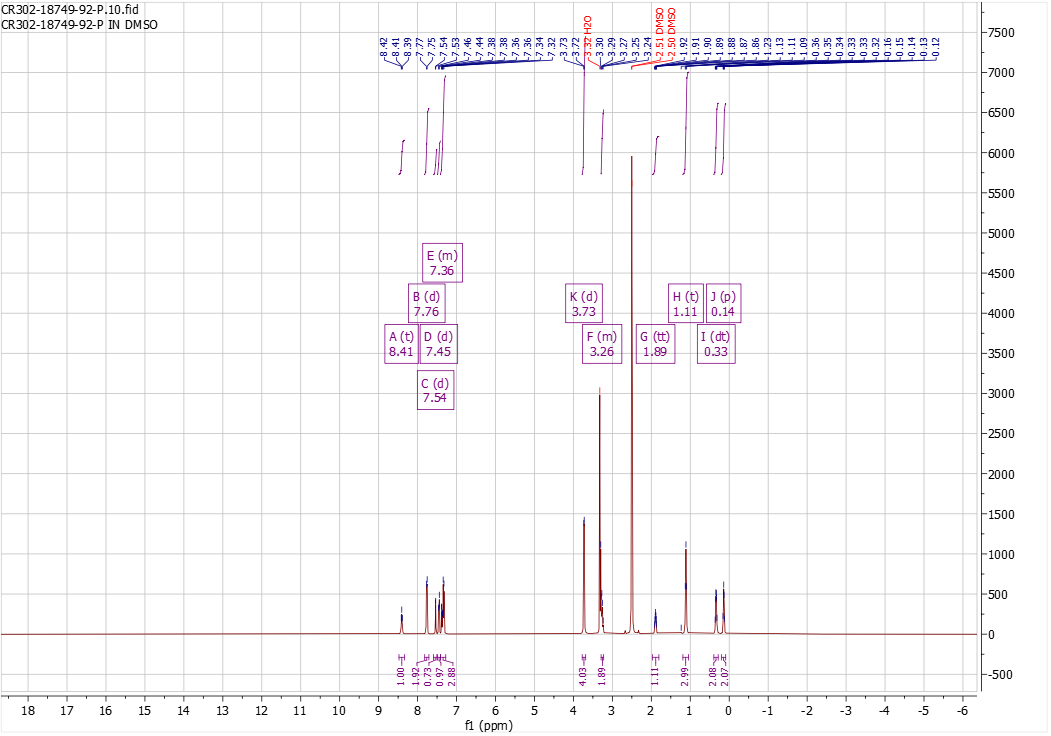
**

**
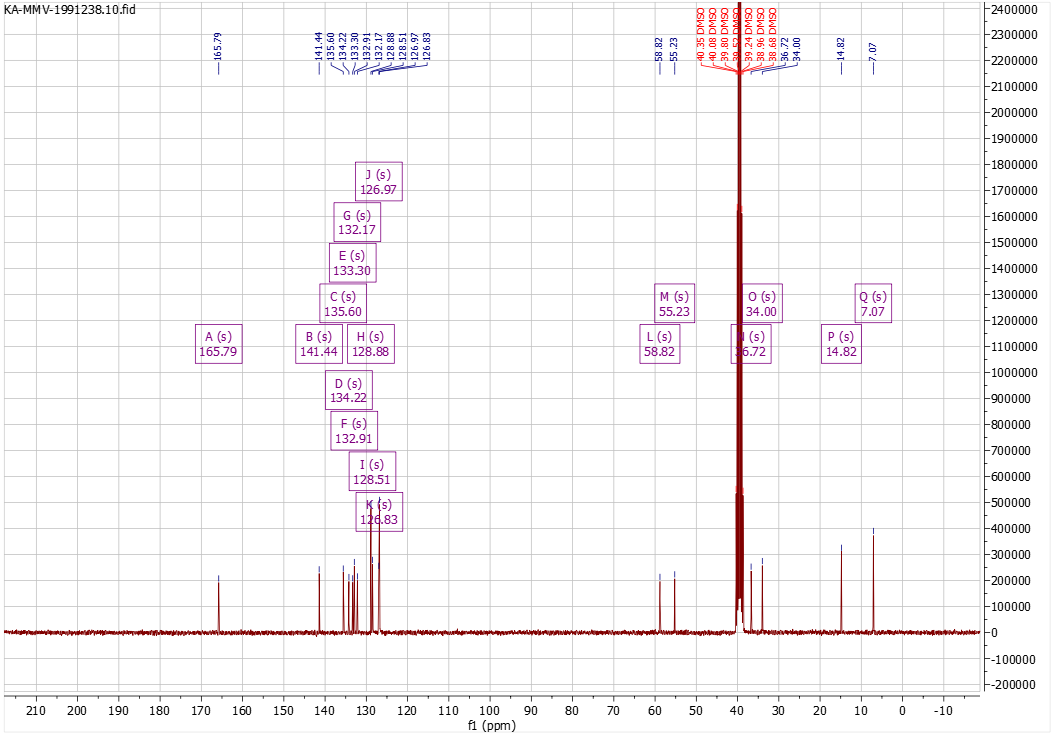
**

Compound **32**


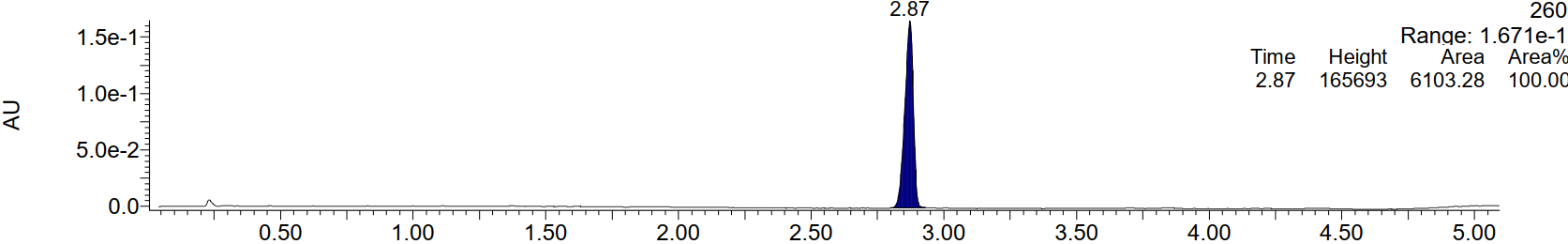


**
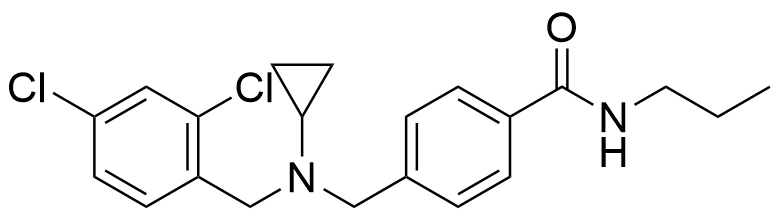
**
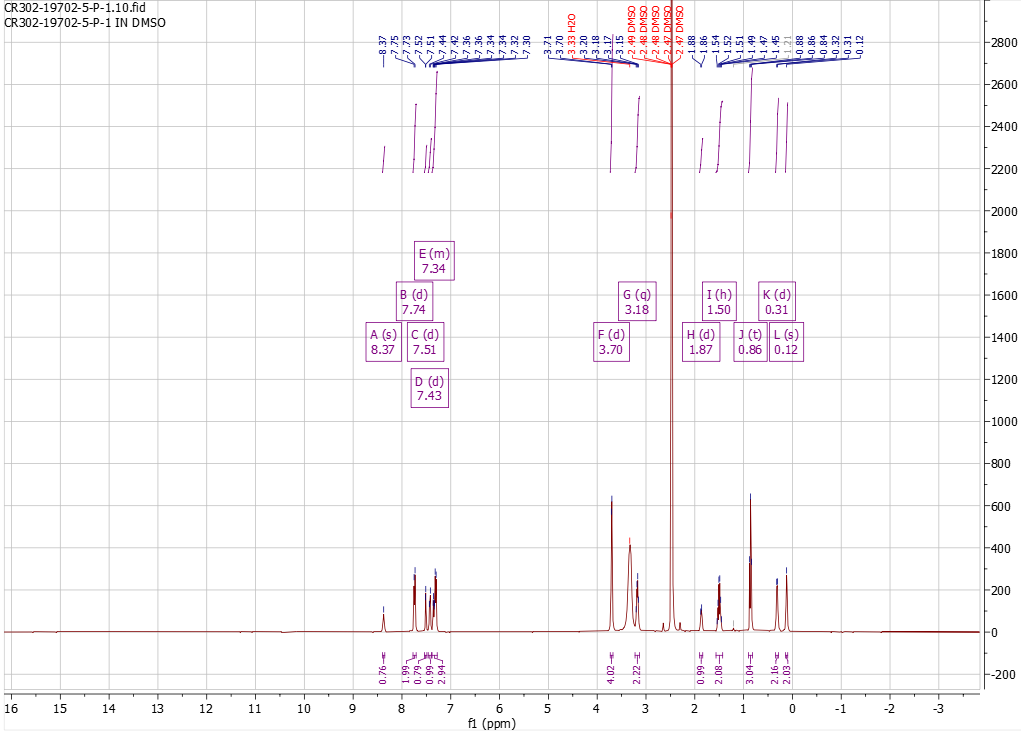


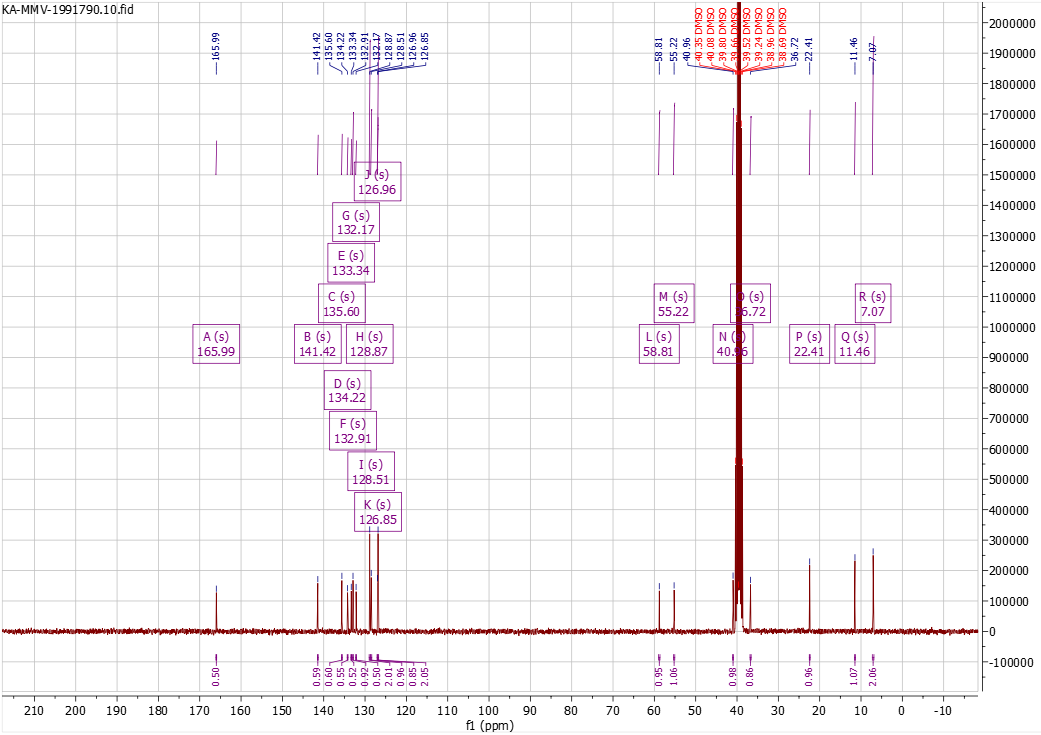


Compound **34**

**
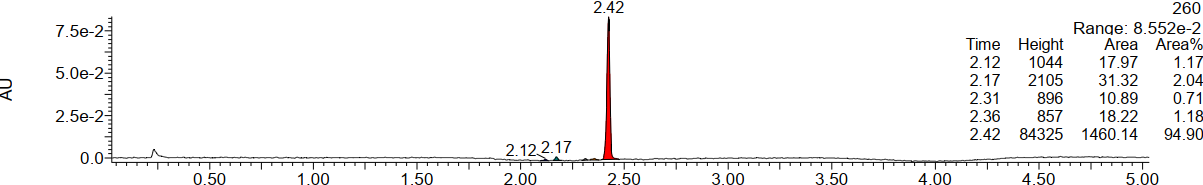
**

**
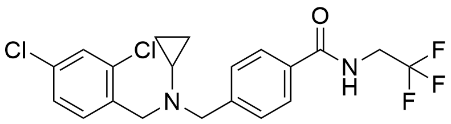
**
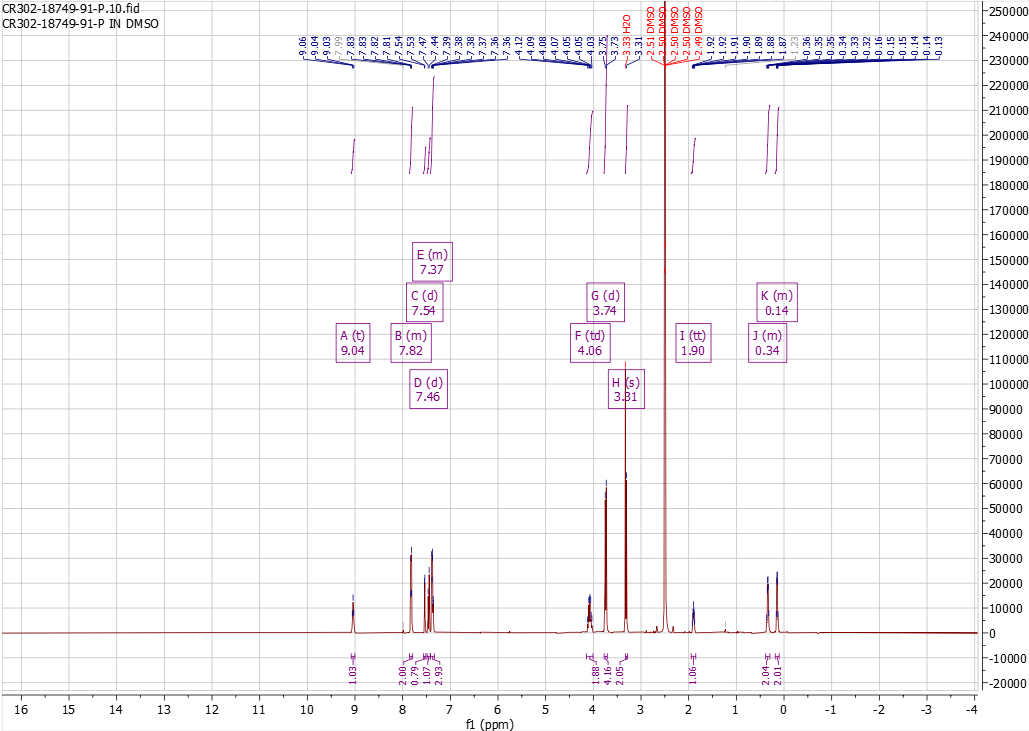


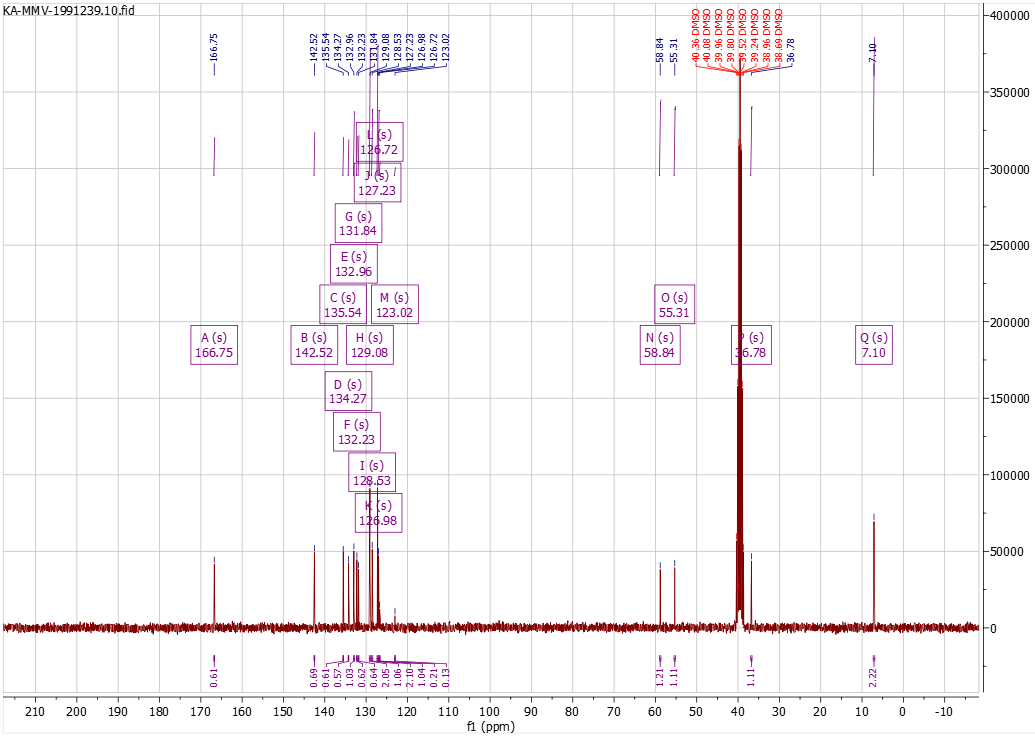


Compound **36**

**
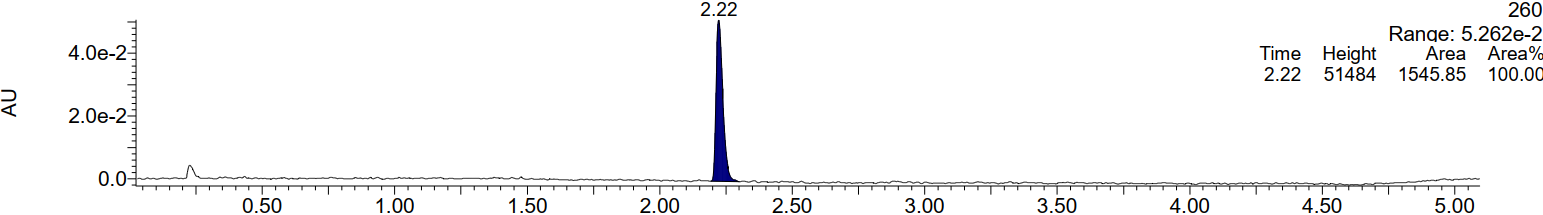
**

**
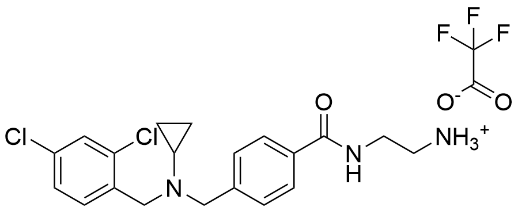

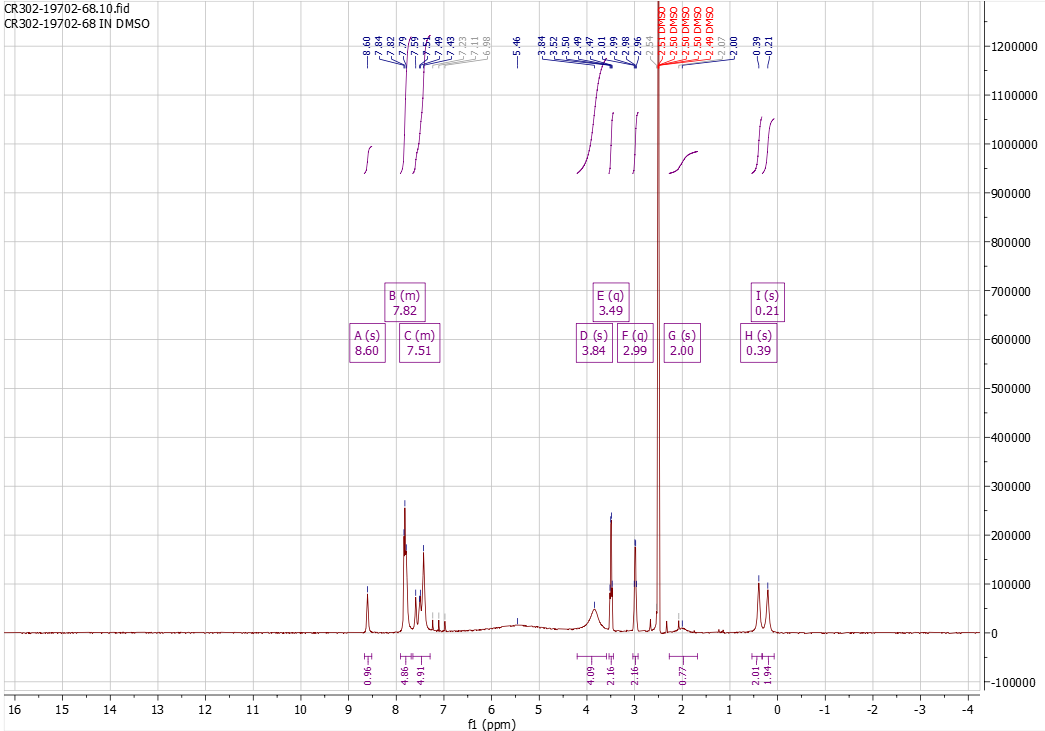
**


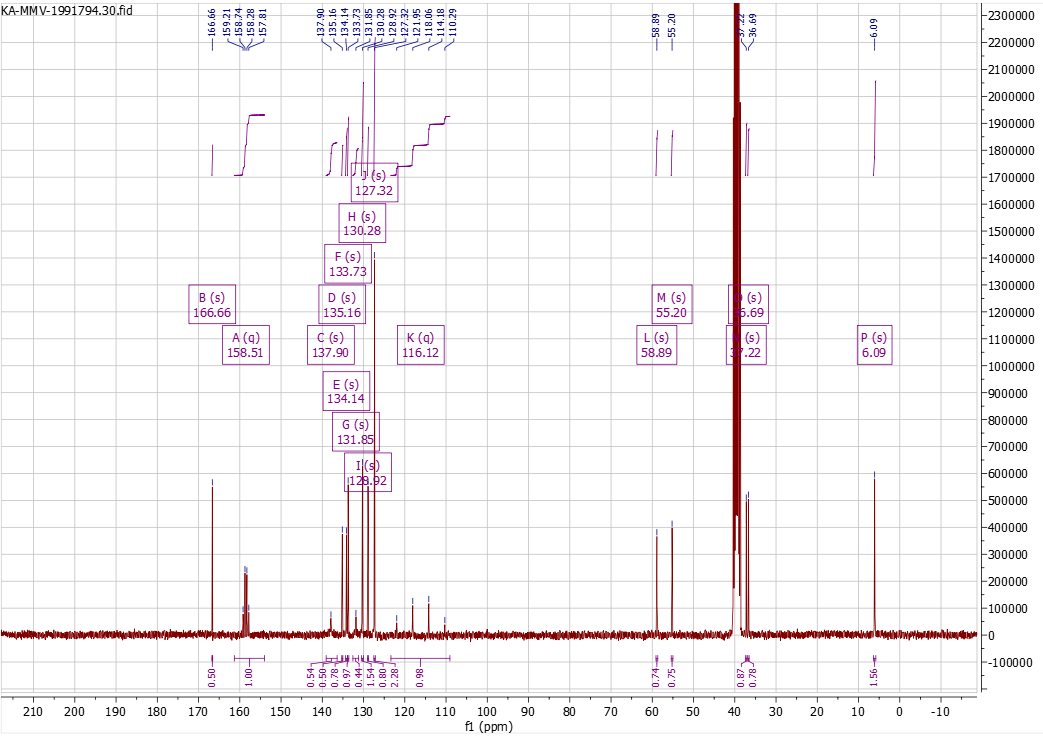


Compound **37**

**
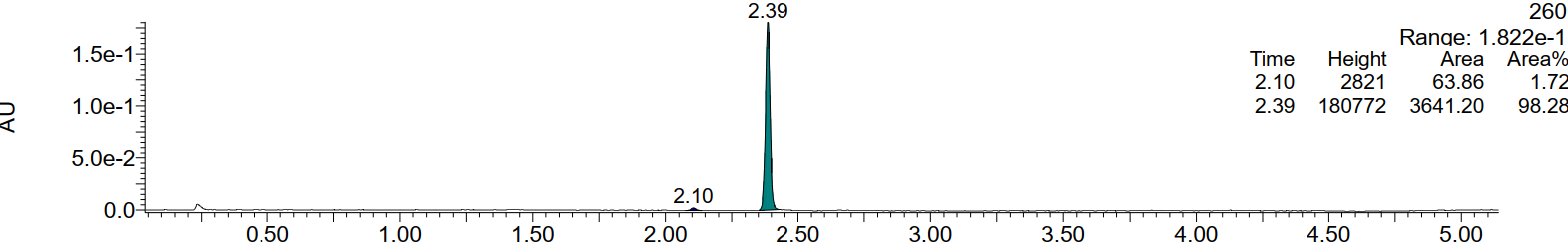
**

**
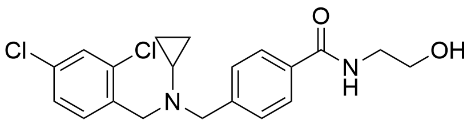
**
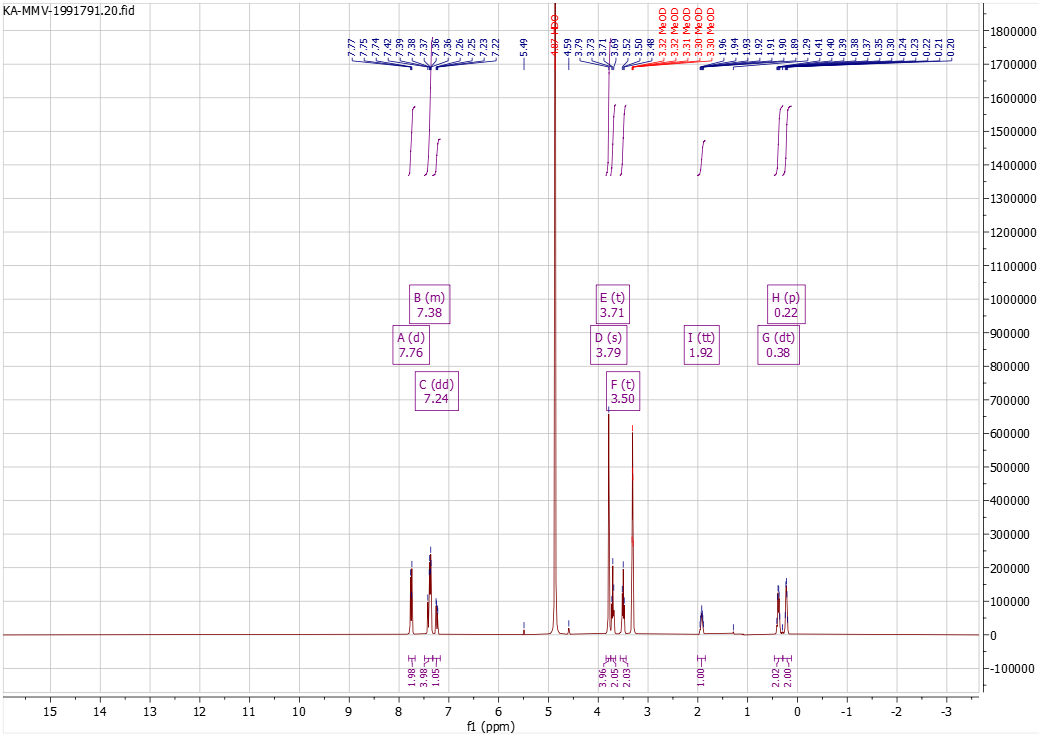


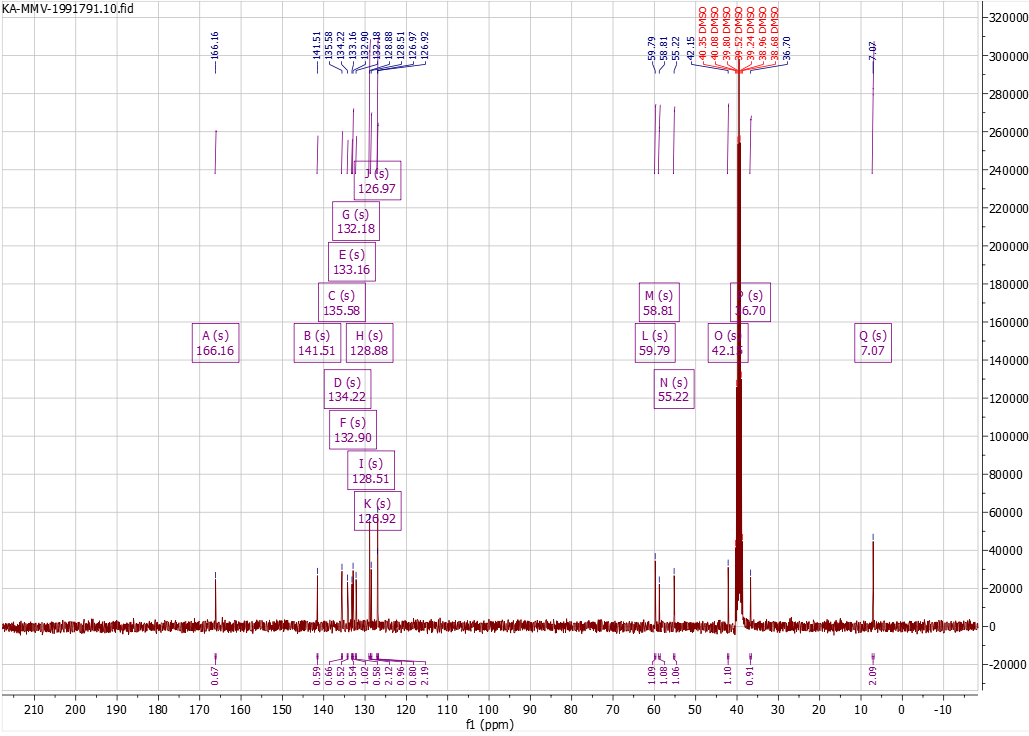


Compound **41**

**
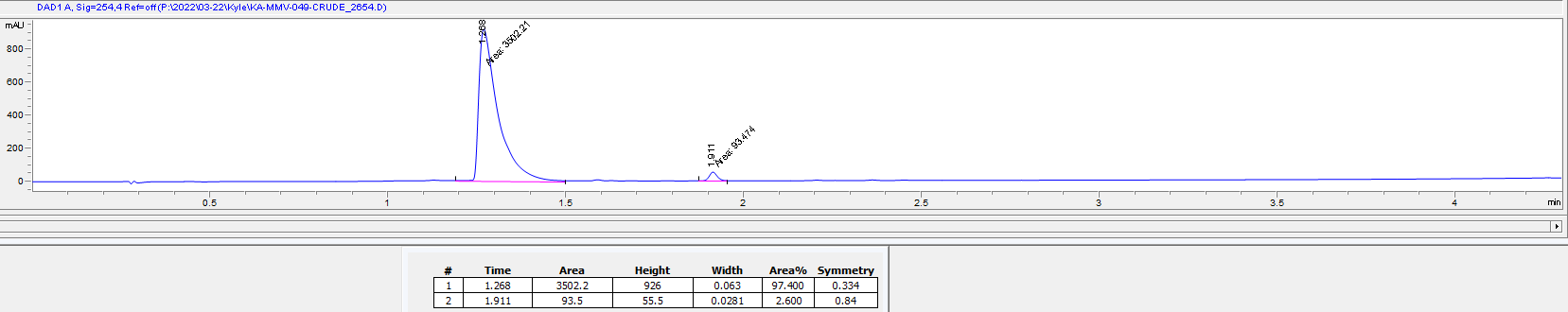
**

**
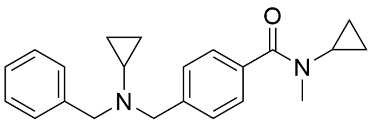
**
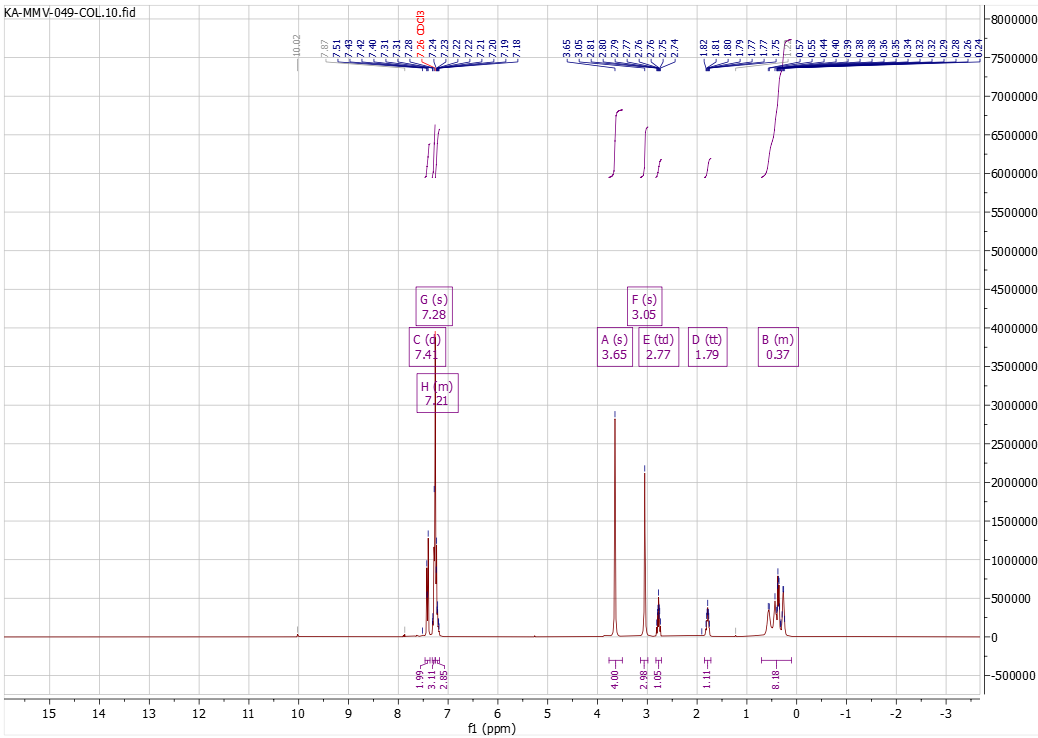


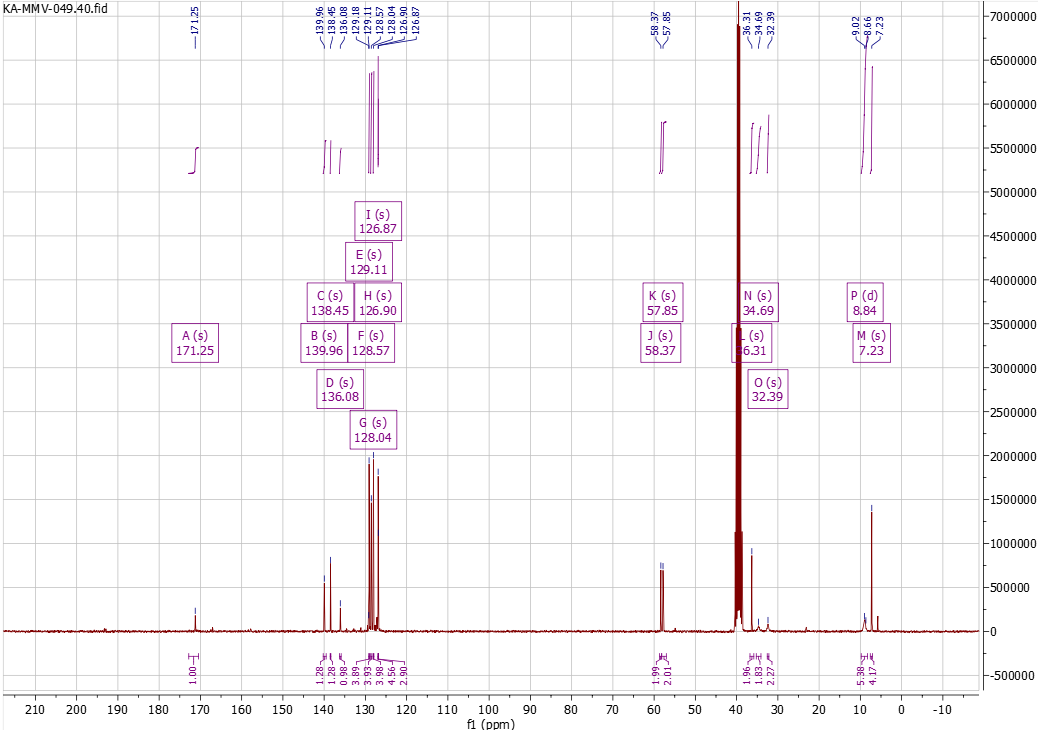


Compound **45**

**
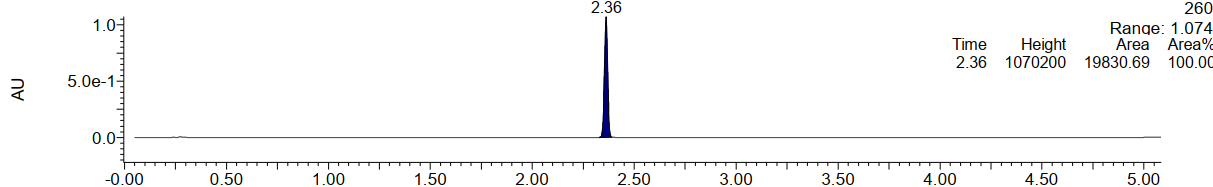
**

**
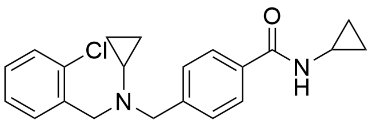
**
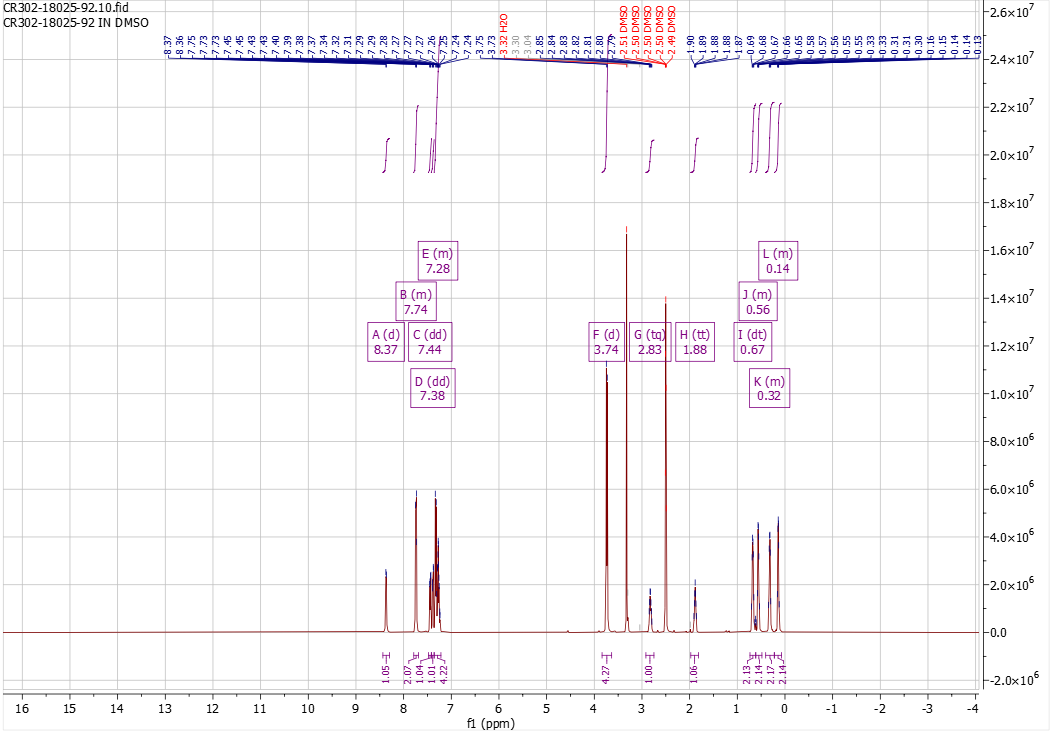


Compound **46**

Compound **47**

Compound **54**

Compound **55**

Compound **63**

Compound **64**

Compound **65**

Compound **66**

Compound **67**

Compound **70**

Compound **71**

Compound **72**

Compound **75**

Compound **78**

Compound **79**

Compound **80**

Compound **82**

Compound **83**

Compound **84**

Compound **88**

Compound **90**

Compound **91**

Compound **97**

Compound **98**

Compound **99**

Compound **102**

Compound **103**

Compound **104**

Compound **105**

Compound **106**

Compound **107**

Compound **108**

Compound **109**

Compound **110**
